# Integrative bulk and single-cell transcriptome analyses reveal RNA modification–related biomarkers of spinal cord injury

**DOI:** 10.4103/NRR.NRR-D-25-00080

**Published:** 2025-11-25

**Authors:** Shixue Huang, Kun Jiao, Keqing Li, Jiayan Yuan, Haoming Shu, Yinuo Zhang, Xin Zhou, Xuhui Zhou

**Affiliations:** 1 Department of Orthopedics, Changzheng Hospital, The Second Affiliated Hospital of Naval Medical University, Shanghai, China; 2 Department of Nursing, Changzheng Hospital, The Second Affiliated Hospital of Naval Medical University, Shanghai, China

**Keywords:** biomarkers, machine learning, microRNA–mRNA (miRNA–mRNA) network, RNA sequencing, RNA modification, single-cell sequencing analysis, spinal cord injury, transcription factor–mRNA network, weighted gene co-expression network analysis

## Abstract

Aberrant RNA modification has been linked to the pathogenesis of various diseases; however, its specific molecular mechanisms in spinal cord injury remain poorly understood. The objective of this study was to explore RNA modification–related biomarkers of spinal cord injury. The mRNA expression profiles of mice with spinal cord injury were retrieved from the Gene Expression Omnibus (GEO) database (GSE18179). We identified 185 differentially expressed genes using bioinformatics approaches. Functional enrichment analysis demonstrated aberrant activation or inhibition of common metabolism–related pathways, including sulfur metabolism and steroid biosynthesis, in mice with spinal cord injury. An integrated strategy comprising weighted gene co-expression network analysis, a random forest model, a support vector machine model, and a generalized linear model was employed to identify four genes whose aberrant RNA modification was linked to spinal cord injury: *Elovl6*, *Idi1*, *Sqle*, and *Stbd1*. We verified the expression levels and diagnostic performance of these four genes in the original training dataset and mouse samples via receiver operating characteristic curve analysis. Quantitative reverse transcription-polymerase chain reaction demonstrated variations in the mRNA levels of the four genes between the Sham and spinal cord injury groups at different time points following injury. We also constructed microRNA–mRNA and transcription factor–mRNA interaction networks using Cytoscape. Additionally, we evaluated the proportions of 22 types of immune cells in the spinal cords of mice using the CIBERSORT tool, revealing significant alterations in the numbers of memory B cells, resting dendritic cells, M0 macrophages, activated mast cells, resting mast cells, and CD8^+^ T cells in spinal cord injury mice compared with Sham controls. Microglia and T cells were identified as key cell types by single-cell sequencing analysis. These findings provide new directions for the development of RNA modification–related therapeutic strategies for spinal cord injury and suggest that *Elovl6*, *Idi1*, *Sqle*, and *Stbd1* are potential biomarkers of spinal cord injury.

## Graphical abstract

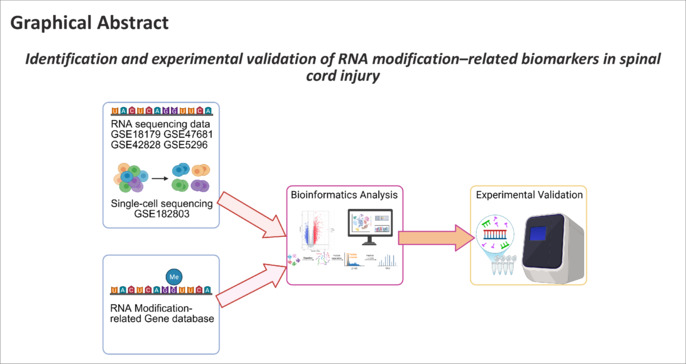

## Introduction

Spinal cord injury (SCI), a prevalent form of central nervous system (CNS) damage in modern society, imposes significant burdens on families, economies, and societies (Khorasanizadeh et al., 2019; Hu et al., 2023). Various therapeutic approaches for SCI have been explored. Commonly used clinical treatments include hormone pulse therapy and hyperbaric oxygen therapy (Liu et al., 2021). However, the complexity of SCI has hindered the development of universally effective treatments, and several studies have highlighted the importance of investigating potential therapeutic and diagnostic biomarkers (Chio et al., 2021; Jing et al., 2021; Saremi et al., 2022; Smith et al., 2022; Huang et al., 2024).

Epigenetic modifications, which are heritable changes in gene expression that do not alter the nucleotide sequence, encompass DNA methylation, histone modification, and RNA modification. Over 170 types of RNA modifications have been identified, establishing RNA modification as a burgeoning field of research (Barbieri and Kouzarides, 2020). Key RNA modifications include N^6^-methyladenosine (m^6^A), N^1^-methyladenosine (m^1^A), 5-methylcytosine (m^5^C), N^7^-methylguanosine (m^7^G), N^4^-acetylcytosine (ac^4^C), pseudouridine (Ψ), uridylation, adenosine-to-inosine (A-to-I) RNA editing, and modifications of the wobble uridine (U34) on tRNAs (Wang et al., 2023). These modifications are crucial for RNA stability and subsequent translation processes (Lin and Kuang, 2024). Aberrant RNA modifications are associated with various diseases and hold important potential in the fields of neurology and oncology (Gao et al., 2025). MicroRNAs (miRNAs), messenger RNAs (mRNAs), and transcription factors (TFs) play crucial and interrelated roles in the field of epigenetic regulation. miRNAs, a class of short non-coding RNAs, are vital for epigenetic regulation (Nikita et al., 2025). They inhibit mRNA translation or induce its degradation via complementary pairing with mRNAs, thereby regulating gene expression (Wang et al., 2025). Multiple miRNAs are involved in the regulation of cell apoptosis and inflammation in SCI (Shao et al., 2025). mRNA carries genetic information and can be regulated via epigenetic modification (Chen et al., 2025b). For instance, the m6A modification, which mainly occurs on mRNA, can influence mRNA stability, translation efficiency, and subcellular localization (Kapadia et al., 2025). In SCI, mRNA modification is closely related to nerve cell survival and axon regeneration (Hong et al., 2020). TFs, which regulate gene transcription initiation and transcription rate by binding to specific DNA regions, are key components of the epigenetic regulation network (Jiao et al., 2025). After SCI, changes in TF mRNA levels lead to alterations in their protein expression levels, thereby activating or inhibiting the expression of genes related to nerve repair and inflammation (Xie et al., 2023). RNA modification–related genes (RRGs) may play important roles in SCI, although the exact mechanisms remain unclear, and RNA modification-related biomarkers are potential targets for SCI diagnosis and treatment. Transcriptomic analysis reveals the global gene expression profiles of cells and tissues, offering insights into molecular disease mechanisms and serving as a valuable tool for early diagnosis and prognosis. However, traditional transcriptomic studies are limited by their inability to capture cell subset heterogeneity (Barbieri and Kouzarides, 2020). Recent advances in single-cell RNA sequencing (scRNA-seq) address these limitations by enabling precise characterization of gene expression at the level of individual cell subsets (Vanlandewijck et al., 2018; Zeisel et al., 2018; Li et al., 2022). Numerous studies have combined transcriptomics with single-cell omics to examine various aspects of SCI, including changes in cell types across different stages (Milich et al., 2021; Cao et al., 2022; Wei et al., 2023).

To investigate the molecular mechanisms regulated by RNA modification in SCI, in this study we identified and validated potential RNA modification–related biomarkers associated with SCI using transcriptomic and single-cell sequencing datasets from public repositories and explored their underlying regulatory mechanisms. These findings not only advance our understanding of epitranscriptomic mechanisms in SCI, but also offer novel therapeutic avenues and candidate targets for RNA modification–based interventions that could have profound translational potential.

## Methods

### Data acquisition

The SCI datasets used in this study were as follows: GSE18179 (platform: GPL81), GSE47681 (platform: GPL1261) (Wu et al., 2013), GSE42828 (platform: GPL1261), GSE5296 (platform: GPL1261), and GSE182803 (platform: GPL24247) (Wang et al., 2022). All of the datasets were retrieved from the Gene Expression Omnibus (GEO) database (https://www.ncbi.nlm.nih.gov/geo/). GSE18179, comprising 32 motoneuron cells from mice that underwent complete spinal cord transection (SCI group) and 30 motoneuron cells from sham-operated control mice (control group), was used as the training set. GSE47681 and GSE42828 were designated as validation set 1 and validation set 2, respectively; each contained 13 SCI samples (specifically, five samples from 1 day, four samples from 3 days, and four samples from 7 days post-SCI) along with four control samples from wild-type (WT) mice. Furthermore, GSE5296 included spinal cord sections from the impact site (I) and the regions above (A) and below (B) the impact site, collected at 0.5 hours, 4 hours, 24 hours, and 72 hours, as well as 7 days and 28 days, post-SCI (three samples per group), along with sham-injury controls (two samples per group). The GSE182803 scRNA-seq dataset included two samples of immune cells derived from injured spinal cords (SCI group) and two samples from healthy spinal cords of mice (control group). Additionally, RRGs (**Additional Table 1**) were sourced from the published literature (Luo et al., 2024), encompassing 10, 2, 13, 3, 22, 1, and 23 genes associated with m^1^A, m^3^C, m^5^C, hm^5^C, m^6^A, m^6^Am, m^7^G, respectively, and 3, 3, 12, 3, 7, 14, and 4 genes associated with RNA cap methylation, uridylation, pseudouracil, adenosine-to-inosine (A to I) RNA editing, mcm^5^s^2^U, APA, and RNA ribose methylation, respectively. Following deduplication, 114 RRGs remained.

**Table 1:** Marker genes of cell types

Cell types	Marker genes
Fibroblast	*Postn, Tmem176a, Tmem176b*
Monocyte	*Thbs1, Gpnmb, Ecm1*
Microglia	*Tmem119, Sall1, P2ry12*
Neutrophil	*S100a9, Mmp9, Cd177*
T-cell	*Cd3e, Cd3g, Trbc2*
B-cell	*Cd79a, Cd19, Top2a*
Dendritic cell	*Cd209a, Ccr7, Cd74*
Mast cell	*Prtn3, Plac8, Iqgap24*
Macrophage	*Mrc1, Ms4a7, Cd163*
Natural killer cell	*Klrb1c, Klre1, Ncr1*
Endotheliocyte	*Ptn, Fermt2, Gng11*

### Differential expression analysis

The limma package (v 3.54.0) (https://www.bioconductor.org/packages/release/bioc/html/limma.html) was used to identify genes that exhibited differential expression between the SCI and control groups in GSE18179 (|log2fold change (FC)| > 0.25, *P* < 0.05) (Ritchie et al., 2015). Moreover, the differentially expressed gene (DEG) volcano plot and heatmap were generated utilizing the ggplot2 package (v 3.4.1) (https://cran.r-project.org/web/packages/ggplot2/index.html) and pheatmap package (v 1.0.12) (https://CRAN.R-project.org/package=pheatmap), respectively (Gustavsson et al., 2022; Liu et al., 2023). The volcano plot shows the top 10 most significantly up- and down-regulated genes, ranked by log2FC values, while the heatmap illustrates their expression patterns.

### Functional analysis of differentially expressed genes

The biological functions of the DEGs were elucidated by using the clusterProfiler package (v 4.7.1.003) (https://www.bioconductor.org/packages/release/bioc/html/clusterProfiler.html) to perform Gene Ontology (GO) and Kyoto Encyclopedia of Genes and Genomes (KEGG) enrichment analyses (*P* < 0.05) (Yu et al., 2012). The GO analysis comprises three principal sections: biological process (BP), cellular component (CC), and molecular function (MF). The genes enriched in each GO entry were sorted in descending order of count, displaying the top three pathways with the most significant enrichment in BP, CC, and MF, respectively. Similarly, the top five most significantly enriched KEGG pathways are presented.

### Weighted gene co-expression network analysis

Single-sample gene set enrichment analysis was performed using the GSVA package (v 1.42.0) (https://www.bioconductor.org/packages/release/bioc/html/GSVA.html) to compute RRG scores across all samples in GSE18179 (Liu et al., 2024). Subsequently, the Wilcoxon test was applied using the rstatix package (v 0.7.2) (https://CRAN.R-project.org/package=rstatix) to ascertain whether a significant discrepancy existed between the RRG scores for the SCI and control groups (*P* < 0.05) (Rivas et al., 2024). Weighted gene co-expression network analysis (WGCNA) was conducted on all samples in GSE18179 utilizing the WGCNA package (v 1.72.5) (https://cran.r-project.org/web/packages/WGCNA/index.html) to identify modular genes associated with RRG phenotypes (Langfelder and Horvath, 2008). All samples were subjected to clustering analysis using the GoodSamplesGenes function to construct a sample clustering tree, and outlier samples were excluded. Next, to maximize the scale-free topological fit of the interactions between genes, a soft threshold (power) was selected for construction of a co-expression network, based on a scale-free fit index (signed *R*^2^) exceeding 0.85, with a mean connectivity approaching 0. A scale-free network was constructed based on the selected soft threshold (power), with the genes divided into multiple modules. Each module comprised a minimum of 100 genes, and the mergeCutHeight threshold was determined to be 0.25. Following this, co-expression modules were identified, resulting in the generation of a hierarchical clustering tree. The RRG scores were employed as phenotypic traits, and Pearson correlation analysis was conducted utilizing the Hmisc package (v 5.1.3) (https://CRAN.R-project.org/package=Hmisc) to generate a correlation matrix between RRG scores and co-expression modules (|correlation coefficient (cor)| > 0.30, *P* < 0.05) (André et al., 2024). The modules exhibiting the most robust positive and negative correlations with the RRGs score were identified and designated as the two key modules, and the key module genes were obtained from these two key modules.

### Identification and functional analysis of candidate genes

The overlap between the DEGs (associated with SCI) and key module genes (associated with RRGs) was assessed using the VennDiagram package (v 1.7.3) (https://CRAN.R-project.org/package=VennDiagram) (Chen and Boutros, 2011). The chromosomal distribution of the resulting candidate genes was visualized using the RCircos package (v 1.2.2) (https://cran.r-project.org/web/packages/RCircos/index.html) (Zhang et al., 2013). Furthermore, to investigate the interactions between candidate genes at the protein level, the Search Tool for the Retrieval of Interaction Gene/Proteins (STRING) database (https://www.string-db.org) (Szklarczyk et al., 2021) was used to construct a protein–protein interaction (PPI) network (confidence = 0.15). The PPI network was visualized utilizing Cytoscape software (v 3.9.1) (https://cytoscape.org/) (Shannon et al., 2003). Additionally, GO and KEGG pathway enrichment analyses of the candidate genes were performed using the clusterProfiler package (v 4.7.1.003), with a significance threshold of *P* < 0.05. The genes enriched for each GO category were ranked in descending order based on their counts, and the top 10 pathways exhibiting the most significant enrichment were highlighted. The top five KEGG pathways are presented.

### Identification of biomarkers through machine learning, expression validation, and receiver operating characteristic analysis

The caret package (v 6.0-94) (https://cran.r-project.org/web/packages/caret/index.html) was used to construct a random forest (RF) model, support vector machine (SVM) model, and generalized linear model (GLM) of the candidate genes identified based on GSE18179 (Nukui and Onogi, 2023). The DALEX package (v 2.4.3) (https://jmlr.org/papers/v19/18-416.html) is a dedicated model interpretation tool designed to facilitate comprehension of the relationships between input variables and model outputs (Qin et al., 2024). The DALEX package (v 2.4.3) was used to perform a residual-based assessment of model quality, in which smaller residuals indicated superior model performance. Furthermore, root mean square error was employed to evaluate the importance of variables. Upon completion of the modeling process, residual box plots for the three models were generated using the ggpubr package (v 0.6.0) (https://CRAN.R-project.org/package=ggpubr), and the importance of each gene within the models was then evaluated by root mean square error (Bian et al., 2023). Subsequently, receiver operating characteristic (ROC) curves were plotted utilizing the pROC package (v 1.18.5) (https://cran.r-project.org/web/packages/pROC/index.html), and area under the curve (AUC) values were calculated to evaluate the predictive efficacy of the models (Robin et al., 2011). An AUC value of more than 0.7 indicated favorable predictive ability. The optimal models exhibiting high predictive accuracy were identified based on model quality (assessed through residual size) and AUC value. Subsequently, the top 10 genes were selected from these models according to their importance scores. Intersections among these genes were identified using the VennDiagram package (v 1.7.3) (https://cran.r-project.org/web/packages/VennDiagram/index.html), and the overlapping genes were defined as feature genes (Chen and Boutros, 2011). Differences in feature gene expression between the SCI and control groups in GSE18179, GSE47681, and GSE42828 were analyzed by Wilcoxon test. Genes exhibiting consistent expression trends and significant inter-group expression differences (*P* < 0.05) across the aforementioned three datasets were identified as candidate biomarkers. Furthermore, ROC curves were generated in GSE18179 utilizing the pROC package (v 1.18.5) (Robin et al., 2011), and the diagnostic efficacy of the candidate biomarkers for SCI was evaluated by computing the AUC values. Candidate biomarkers were defined as those with AUC values exceeding 0.7.

### Nomogram establishment and assessment

Within the GSE18179, a nomogram was constructed using the rms package (v 6.5.0) (https://CRAN.R-project.org/package=rms) to assess the ability of biomarkers to predict SCI onset (Wang et al., 2024b). In the nomogram, each biomarker was assigned a point, and the total sum of the biomarker points was used to infer the incidence of SCI. A higher score was indicative of an increased probability of sustaining SCI. Moreover, a calibration curve was plotted using the rms package (v 6.5.0) to verify the accuracy of the nomogram. Concurrently, the ROC curve was plotted using the pROC package (v 1.18.5), and the diagnostic value of the nomogram model for SCI was assessed by calculating the AUC value. Furthermore, a decision curve analysis (DCA) was performed using the rmda package (v 1.6) (https://CRAN.R-project.org/package=rmda) to evaluate the clinical utility of the nomogram.

### Biomarker enrichment analyses

Gene set enrichment analysis (Subramanian et al., 2005) was conducted to elucidate the biological functions of the biomarkers throughout SCI progression. The Spearman correlation coefficients among each biomarker and all other genes across all samples in GSE18179 were calculated using the psych package (v 2.1.6) (https://CRAN.R-project.org/package=psych), and the results were listed in descending order (Kong et al., 2022). ‘’m2.cp.v2023.1.Mm.symbols.gmt” from the Molecular Signatures Database (MSigDB) (https://www.gsea-msigdb.org/) (Liberzon et al., 2015) was used as a reference gene set, and gene set enrichment analysis was performed utilizing the clusterProfiler package (v 4.7.1.003) (*P*_adj_ < 0.05). In addition, the KEGG mapper tool, accessible via the KEGG database (https://www.kegg.jp/), was employed to identify pathways containing the biomarkers.

### Immune microenvironment analysis

The immune microenvironment of the SCI and control groups in GSE18179 was subjected to further analysis. Initially, the genes within GSE18179 were transformed homozygously, after which the CIBERSORT algorithm was applied to calculate the abundance of 22 distinct types of infiltrating immune cells across all samples (Li et al., 2024). Subsequently, a stacked bar chart was generated using the ggplot2 package (v 3.4.1) to illustrate the distribution of the 22 distinct immune cell types. Furthermore, the rstatix package (v 0.7.2) was utilized to perform the Wilcoxon test to compare immune cell proportions between the SCI and control groups (*P* < 0.05). Spearman correlation analysis was conducted using the psych package (v 2.1.6) to evaluate the relationship between biomarkers and differentially abundant immune cells across all samples in GSE18179 (*P* < 0.05).

### Regulatory network construction

Molecular regulatory networks provided insight into the fundamental mechanisms of gene regulation in disease processes. The miRanda (http://mirtoolsgallery.tech/mirtoolsgallery/node/1055) and miRDB (http://mirdb.org/) databases (Chen and Wang, 2020), which are included in the multiMiR package (v 0.98.0.2) (https://www.bioconductor.org/packages/release/bioc/html/multiMiR.html), were used to predict miRNAs that regulate the biomarkers (Ru et al., 2014). Overlapping biomarker–miRNA pairs predicted by both databases were identified to determine the key miRNAs. Furthermore, TFs that interact with the biomarkers were predicted utilizing the miRNet database (https://www.mirnet.ca/) (Chang et al., 2020). The resulting miRNA–mRNA and TF–mRNA networks were visualized using Cytoscape software (v 3.9.1).

### Drug prediction and molecular docking

Drugs with the potential to target biomarkers involved in homologous conversion were identified through the Comparative Toxicogenomics Database (CTD) (http://ctdbase.org/) (Davis et al., 2023). The top 10 drugs for each predicted biomarker were downloaded, and a network map of the drugs and biomarkers was generated using Cytoscape software (v 3.9.1). Drugs that were predicted to target more than one of the biomarkers were defined as key drugs, and then subjected to molecular docking analysis bound to the biomarkers. Then, the key drugs were imported into the public chemistry database (PubChem) (https://pubchem.ncbi.nlm.nih.gov/) (Kim et al., 2021) to obtain their three-dimensional structures. Moreover, the protein crystal structures and ligand molecular structures of the biomarker target proteins were obtained from the Protein Data Bank (PDB) database (http://www.rcsb.org) (Burley et al., 2017). Next, the biomarkers were subjected to molecular docking analysis bound to the key drugs utilizing the CB-Dock2 online tools (https://pubchem.ncbi.nlm.nih.gov/) (Liu et al., 2022), and the binding free energies were calculated. Subsequently, the results were visualized using Pymol software (v 3.0.3) (http://www.pymol.org/pymol) (Rosignoli and Paiardini, 2022).

### Animals

Male and female C57/BL6 mice (specific-pathogen-free (SPF) grade) aged 8–9 weeks and weighing 20 g were procured from Shanghai Jihui Laboratory Animal Co., Ltd. (Shanghai, China) (license No. SCXK (Zhe) 2024-0002). The mice were housed three per cage under standard conditions with a 12/12‐hour light/dark cycle. All animal experiments were approved by the Animal Welfare and Research Ethics Committee of Shanghai Changzheng Hospital (Shanghai, China) on June 28, 2025 (approval No. 2025SLYS5) and were conducted in strict accordance with the National Institutes of Health’s Guide for the Care and Use of Laboratory Animals.

### Spinal cord injury model

A crush model of SCI was established, as previously reported (Gu et al., 2019). The animals were randomly assigned to one of three groups (*n* = 3/group): the sham group, SCI d7 group, and SCI d28 group. Anesthesia was induced using 3% inhaled isoflurane (Yaji Biological Technology, Shanghai, China) and maintained with 1.5% isoflurane. Subsequently, a laminectomy was performed at the T7–T9 level to expose the spinal cord. Then, the mice in the sham group underwent suturing of the incision. For the other two groups, spinal cord trauma was induced by laterally compressing the T8 spinal cord using a pair of forceps with a 0.40 mm spacer for 15 seconds. During the crushing process, excessive contraction of the hindlimb muscles resulted hindlimb rigidity. Upon recovery from anesthesia, the mice exhibited hindlimb paralysis, with an inability to voluntarily move the hindlimbs to perform actions such as standing or kicking. Following injury, a distinct lesion appeared in the mouse spinal cord, effectively disconnecting the upper and lower spinal segments. Immunofluorescence staining for neuronal nuclei and glial fibrillary acidic protein revealed a marked loss of neurons and an accumulation of astrocytes in the damaged region. The Basso Mouse Scale (BMS) was used to assess hindlimb locomotor performance in the mice by two trained observers who were unaware of the experimental group conditions. The BMS score, ranging from 0 to 9, was determined based on several locomotor parameters, including ankle movement, paw position, trunk stability, and stepping coordination in mice placed in an open field. Any discrepancy of more than two score points between the left and right hindlimbs resulted in the exclusion of the animal from the analysis. Our observations indicated that the crush model of SCI was successfully established (**Additional Figure 1**).

**Figure 1:**
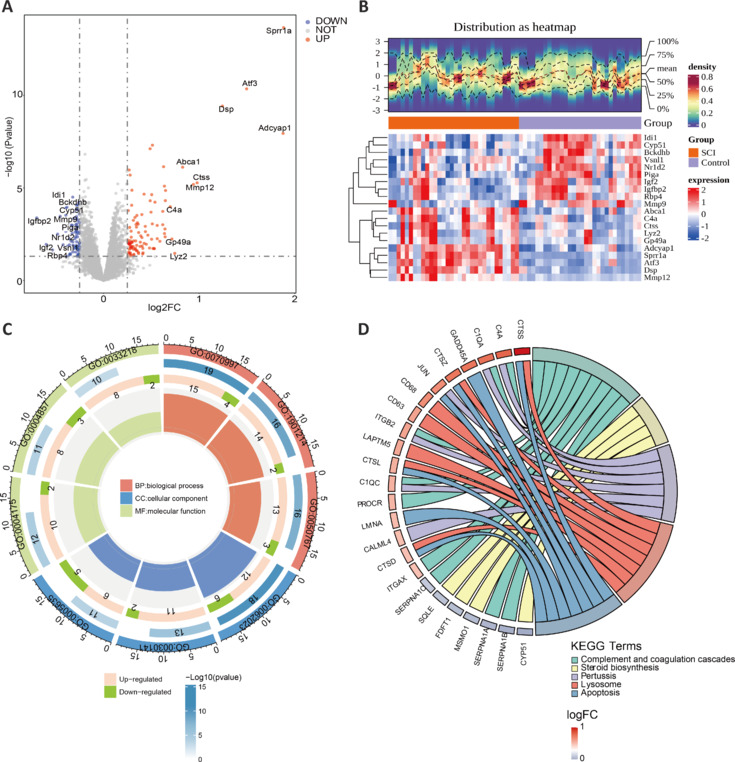
Identification of DEGs and exploration of their biological pathways. (A) Volcano plot of DEGs between the SCI and control groups in the GSE18179 dataset (moderated *t* test). (B) Heatmap of 185 DEGs between the SCI and control groups in the GSE18179 dataset. (C) GO size plot of DEGs (hypergeometric test and multiple hypothesis testing correction). (D) KEGG enrichment analysis of the DEGs (hypergeometric test and multiple hypothesis testing correction). In A and B, red and blue dots indicate genes whose expression is upregulated and downregulated, respectively, between the two groups. The gray dots indicate non-differentially expressed genes. In C, GO:0033218, GO:0004857, GO:0004175, GO:0005635, GO:0030141, GO:0062023, GO:0070997, GO:1901214, and GO:0050767 stand for “amide binding”, “enzyme inhibitor activity”, “endopeptidase activity”, “nuclear envelope”, “secretory granule”, “collagen-containing extracellular matrix”, “neuron death”, “regulation of neuron death”, and “regulation of neurogenesis”, respectively. DEG: Differentially expressed gene; GO: Gene Ontology; KEGG: Kyoto Encyclopedia of Genes and Genomes; SCI: spinal cord injury.

A heating pad was used postoperatively to aid in the recovery of the mice and improve their survival rate. Upon awakening, mice were housed in pairs in a well-ventilated environment with a temperature of 20–26°C and a humidity of 50%–60%, and were provided with sufficient rodent chow and clean drinking water. The bladders were manually massaged twice daily to promote urination and defecation until the mice regained their autonomous urinary function.

### Expression and validation of biomarkers

A *t*-test was conducted using the rstatix package (v 0.7.2) to evaluate the expression patterns of biomarkers from disparate regions (I, A, B) within GSE5296 at varying stages of SCI progression. The expression levels of the biomarkers were verified by quantitative reverse transcription-polymerase chain reaction (qRT-PCR). Spinal cord samples were obtained from three mice that had undergone a sham operation (sham group), three mice at 7 days post-SCI (SCI d7 group), and three mice at 28 days post-SCI (SCI d28 group). Total RNA was extracted from the frozen spinal cord tissue samples using a TRizol kit (15596-018CN, Ambion, Waltham, MA, USA) according to manufacturer’s instructions. The concentration of a 1 μL sample of the extracted RNA was measured using a NanoPhotometer N50 (Implen, Munich, Germany), and the recorded purity/concentration was used to calculate the amount of RNA required for subsequent reverse transcription steps. Subsequently, the RNA was reverse transcribed into cDNA using Servicebio’s SweScript First Strand cDNA Synthesis Kit (Servicebio, Wuhan, China), in accordance with the instructions provided. Next, the cDNA was diluted 5 to 20 times with ddH_2_O (without RNase/ARase) and mixed with 3 μL cDNA, 5 μL 2×Universal Blue SYBR Green qPCR Master Mix (Servicebio), 1 μL forward primer (10 µM), and 1 μL reverse primer (10 µM). A 40-cycle program (excluding the pre-denaturation steps) was carried out an a CFX Connect RT-qPCR instrument (BIO-RAD, XLFZ006, Hercules, CA, USA); detailed program information is provided in **Additional Table 2**. The primer sequences are presented in **Additional Table 3**. GAPDH was employed as the reference gene, and relative gene expression levels were determined utilizing the 2^–ΔΔCT^ method (Eker et al., 2022). Histograms were generated using GraphPad Prism 5 (GraphPad Software, San Diego, CA, USA, www.graphpad.com) to illustrate disparities in biomarker mRNA expression levels between the sham group, the SCI d7 group, and the SCI d28 group.

**Additional Table 2:** Procedure of quantitative reverse transcription-polymerase chain reaction

Procedures	Temperature (°C)	Time
Pre-denaturation	95	1 min
denaturation	95	20 s
anneal	55	20 s
extend	72	30 s

**Additional Table 3:** Primer sequences

Primer	Sequences (5'-3')	Product size (bp)
Elovl6	F: CAATGCTCAGCCCTGGATGT	20
	R: TTCGGAGTCGCTACGTGTTC	20
Stbd1	F: AAAGCACAAGAACGGGCTGA	20
	R: GAGTCACCAGACCAGTGCAA	20
Idi 1	F: ACATACAGCAGCAGCCTAGC	20
	R: TCTCGGAAAGTGGTGAAGGC	20
Sqle	F: GCCTGCCTTTCATTGGCTTC	20
	R: TTCCTTTTCTGCGCCTCCTG	20
GAPDH	F: CGAAGGTGGAGTCAACGGATTT	22
	R: ATGGGTGGAATCATATTGGAAC	22

### Single-cell RNA sequencing data processing

Quality control (QC) analysis was conducted for all samples (control: GSM5537262, GSM5537263; SCI: GSM5537264, GSM5537265) from GSE182803 using the PercentageFeatureSet function. The scRNA-seq data were processed using the Seurat package (v 4.1.0) (https://cran.r-project.org/web/packages/Seurat/index.html) (Hao et al., 2021). Genes whose expression was detected in fewer than three cells were excluded. Cells were excluded based on the following criteria: nFeature_RNA (number of detected genes) less than 200 or greater than 7000, nCount_RNA (total number of RNA counts per cell) less than 40,000, and percent.mt (percentage of mitochondrial gene expression) greater than 20%. Violin plots of the nFeature_RNA, nCount_RNA, and percent.mt before and after QC data were generated using the ggplot2 package (v 3.4.1). Next, the ControlizeData function was used to standardize the data, with the parameters set to ‘’LogControlize” and scale.factor = 10,000. A total of 2000 hypervariable genes were extracted utilizing the FindVariableFeatures function. The results were visualized utilizing the LabelPoints function, and the top 10 genes were labeled. Then, the data were normalized using the Scale Data function. Principal component analysis (PCA) was carried out to downscale the scRNA-seq data using the RunPCA function, based on the 2000 hypervariable genes. The top 30 most statistically significant principal components (PCs) (*P* < 0.05) were identified. Unsupervised clustering analysis was conducted using the FindNeighbors and FindClusters functions, based on the top 30 PCs, with the resolution set to 0.4, to determine the number of cell clusters. Uniform Manifold Approximation and Projection (UMAP) was used to visualize the cell clusters, which were annotated based on marker genes previously reported in the literature (Wang et al., 2022).

### Identification of key cells

Bar graphs were generated to illustrate the proportional distribution of annotated cell types between the SCI and control (sham) groups in GSE182803. Next, the *t*-test was employed to compare the percentage of annotated cells between the SCI and control groups utilizing the rstatix package (v 0.7.2). Cells exhibiting significant differences in abundance between the two groups (*P* < 0.05) were identified as differentially abundant cells. The ReactomeGSA package (v 1.16.1) (https://www.bioconductor.org/packages/release/bioc/html/ReactomeGSA.html) was used to identify biological pathways associated with the differentially abundant cells (Griss et al., 2020). Then, the ggplot2 package (v 3.4.1) was utilized to illustrate differences in biomarker expression in the differentially abundant cells between the SCI and control groups in GSE182803. Cells displaying significant variations in biomarker expression were identified as key cells (*P* < 0.05).

### Cellular communication and pseudo-temporal trajectory analyses

Dimensionality reduction and clustering analyses were conducted on the key cells using the RunPCA, FindNeighbours, and FindClusters functions of the Seurat package (v 4.1.0), resulting in re-clustering of the cells into distinct subpopulations. The CellChat package (v 1.5.0) (https://www.rdocumentation.org/packages/CellChat/versions/1.0.0) was employed to infer interaction dynamics among the annotated cells in the SCI and control groups, thereby elucidating the number of interactions and the interaction weight/strength between key cells and other cells in both groups (Jin et al., 2021b). Furthermore, heatmaps were generated to demonstrate the number of ligand–receptor interactions between key cells and other annotated cells in the SCI and control groups. Moreover, single-cell ligand–receptor relationships were analyzed utilizing the CellChat package (v 1.5.0). Additionally, the differentiation trajectories of all key cells were analyzed utilizing the monocle package (v 2.22.0) (https://www.bioconductor.org/packages/release/bioc/html/monocle.html) (Zhao et al., 2023). The key cells’ trajectories were segmented based on the trajectory nodes, thus enabling investigation of biomarker expression at each stage. Subsequently, the monocle package (v 2.22.0) was employed to delineate dynamic trends in biomarker expression throughout cell differentiation. To visualize the changes in the expression levels of key genes during single-cell differentiation, based on dataset GSE182803, we used the plot_cell_trajectory function of the R package “Monocle” (v 2.26.0) to generate a heatmap of gene expression dynamics (Trapnell et al., 2014). In addition, to understand changes in gene expression during cell state transition, we re-dimensionalized and clustered the microglia and performed a pseudotime analysis. Finally, the “ggplot2” package (v 3.4.1) was used to display the distribution of key genes in different microglia cell subtypes in the single-cell dataset GSE182803.

### Statistical analysis

No statistical methods were used to predetermine sample sizes; however, our sample sizes are similar to those reported in previous publications (Bhalala et al., 2012; Kwon et al., 2015; Wang et al., 2022). The statistical analyses were conducted utilizing R language (v 4.2.2) (https://www.r-project.org/), and significant differences between groups were identified by Wilcoxon test and independent samples *t*-test. For qRT-PCR, *C*_t_ values were compared by unpaired, independent-sample *t*-test using GraphPad Prism 5. *P* < 0.05 was defined as statistically significant. Cor values were calculated by Spearman’s correlation analysis.

## Results

### Identification of 185 differentially expressed genes and exploration of the biological pathways in which they are involved

To elucidate the molecular alterations that occur following SCI, we performed a differential expression analysis, which identified 185 DEGs between the SCI and control groups. Of these, 103 genes were up-regulated and 82 genes were down-regulated in the SCI group compared with the control group (**Figure 1A** and **B**). Subsequently, enrichment analyses were conducted to gain preliminary insight into the signaling pathways in which the DEGs are involved. The DEGs were significantly enriched in 1260 GO entries, comprising 1090 BPs, 77 CCs, and 93 MFs (*P* < 0.05; **Additional Table 4**). The top two BPs were “neuron death” and “regulation of neuron death” (**Figure 1C** and **Additional Table 4**). The top three CCs were “collagen-containing extracellular matrix” and “secretory granule” (**Figure 1C** and **Additional Table 4**). The top three MFs were “endopeptidase activity”’ and “enzyme inhibitor activity” (**Figure 1C** and **Additional Table 4**). These results indicate that DEGs played a pivotal role in regulating neuronal death, constructing the extracellular matrix, and facilitating protein degradation and regulation. Moreover, KEGG enrichment analysis of the DEGs revealed the enrichment of 25 pathways (*P* < 0.05), such as “complement and coagulation cascades” and “steroid biosynthesis” (**Figure 1D** and **Additional Table 5**), suggesting that the DEGs are critical for regulating immune responses, inflammatory processes, and blood clotting.

### Identification and functional analysis of 23 candidate genes

**Figure 2:**
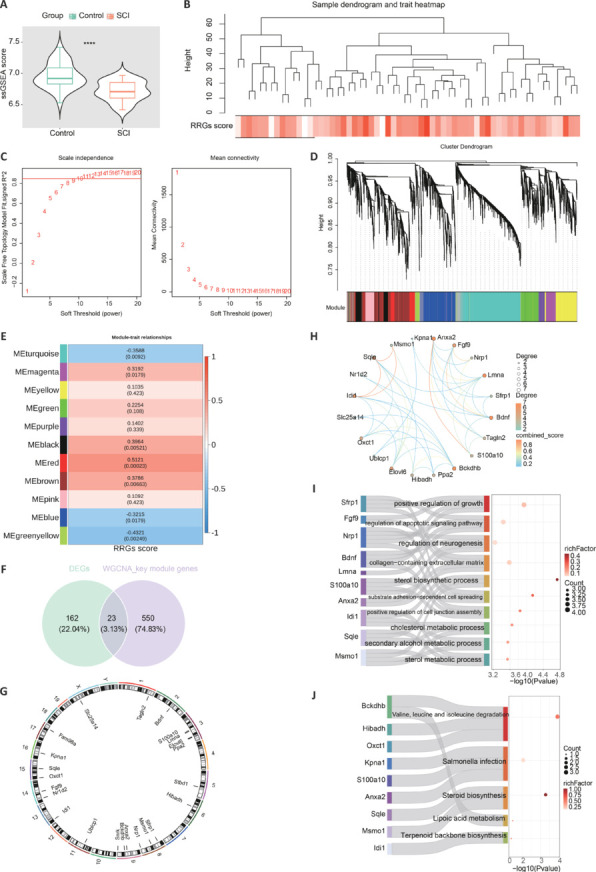
Weighted gene co-expression network analysis and enrichment analysis of 23 candidate genes in the Gene Expression Omnibus (GEO) database GSE18179. (A) Comparisons of RNA modification-related gene scores between the SCI group and the control group (Wilcoxon rank-sum test). ****P < 0.0001. (B) The sample clustering tree of GSE18179. (C) Soft threshold power screening and construction of a scale-free network (scale-free network fitting test). (D) Clustering dendrogram. The hierarchical clustering tree displays the network and the 11 identified modules. (E) Module‒feature associations. Correlation heatmap between module eigengenes and the RRG score (Spearman’s rank correlation test). (F) Venn diagram of the intersection of the DEGs and key module genes identified via WGCNA. (G) The locations of 23 candidate genes on chromosomes. (H) PPI network of the remaining 20 candidate genes. (I) Top ten significantly enriched pathways in the GO analysis of 20 candidate genes (hypergeometric test and multiple hypothesis testing correction). (J) Seven pathways in the KEGG analysis of 20 candidate genes (hypergeometric test and multiple hypothesis testing correction). DEG: Differentially expressed gene; GO: Gene Ontology; KEGG: Kyoto Encyclopedia of Genes and Genomes; PPI: protein–protein interaction; RRGs: RNA modification–related genes; SCI: spinal cord injury; WGCNA: weighted gene co-expression network analysis.

To identify pivotal genes underlying SCI pathogenesis, we performed transcriptomic analyses. The SCI group exhibited markedly lower RRG scores compared with the control group (**Figure 2A**). Subsequently, a WGCNA network was constructed utilizing data from all GSE18179 samples. The sample clustering tree showed no outliers, indicating that all samples formed a part of the WGCNA network (**Figure 2B**). A soft threshold (power) of 11 was identified, along with a signed *R*² value exceeding 0.85 and a mean connectivity of approximately 0 (**Figure 2C**). Hierarchical clustering identified 11 co-expression modules, excluding the gray module attributed to unclassifiable genes (**Figure 2D**). Furthermore, correlation analysis of the gene modules with RRG scores revealed that the MEred module exhibited the most positive Spearman correlation (*Cor* = 0.5121, *P* = 0.00023), whereas the MEgreenyellow module demonstrated the most negative Spearman correlation (*Cor* = –0.4321, *P* = 0.00249; **Figure 2E**). In detail, 447 genes were included in the MEred module and 126 genes in the MEgreenyellow module, resulting in a total of 573 key module genes. Subsequently, 185 DEGs and 573 key module genes were subjected to intersection analysis, resulting in the identification of 23 candidate genes (**Figure 2F**). With the exception of chromosomes 4, 7, 10, 12, 18, and 19 and the Y chromosome, all 23 candidate genes are distributed across the remaining 15 chromosomes (**Figure 2G**). This indicated that the candidate genes are distributed across the entire genome in a relatively dispersed manner. Discrete proteins encoded by three of the candidate genes were excluded, and a PPI network was constructed from the proteins encoded by the remaining 20 candidate genes, such as ELOVL family member 6 (*Elovl6*) and *Idi1* (**Figure 2H**). Moreover, the candidate genes were significantly enriched in 582 GO entries, including 518 BPs, 26 CCs, and 38 MFs (**Additional Table 6**). The top 10 most significantly enriched pathways (*P* < 0.05) included “sterol biosynthetic process” and “substrate adhesion-dependent cell spreading” (**Figure 2I**), which indicated that the candidate genes might play a crucial role in regulating cell growth and apoptosis. Additionally, KEGG analysis of the candidate genes showed enrichment of seven pathways (*P* < 0.05), such as “valine, leucine, and isoleucine degradation” (**Figure 2J** and **Additional Table 7**). Collectively, these analyses established the 23 candidate genes as key regulators of cell growth, apoptosis, and metabolism, providing mechanistic insight into SCI pathophysiology and potential targets for therapeutic intervention.

**Additional Table 7:** KEGG enrichment analysis of the candidate genes

	ID	Description	GeneRatio	BgRatio	P value	Padj	q value	geneID	Count
		V aline, leucine and							
mmu00280	mmu00280	isoleucine degradation - Mus musculus (house mouse) Steroid	3/17	57/9784	0.0001203157 75279044	0.0048126310 1116177	0.0041793900 8864049	Bckdhb/Hibadh/Oxct1	3
mmu00100	mmu00100	biosynthesis - Mus musculus (house mouse) Salmonella	2/17	20/9784	0.0005300806 03312825	0.0106016120 662565	0.0092066631 1017013	Sqle/Msmo1	2
mmu05132	mmu05132	infection - Mus musculus (house mouse) Lipoic acid	3/17	252/9784	0.0087871929 8296083	0.1171625731 06144	0.1017464450 65862	Kpna1/S100a10/Anxa2	3
mmu00785	mmu00785	metabolism - Mus musculus (house mouse) Terpenoid	1/17	19/9784	0.0325313478 480719	0.1911538711 98319	0.1660020460 40645	Bckdhb	1
mmu00900	mmu00900	backbone biosynthesis - Mus musculus (house mouse) Butanoate	1/17	23/9784	0.0392519138 231147	0.1911538711 98319	0.1660020460 40645	Idi1	1
mmu00650	mmu00650	metabolism - Mus musculus (house mouse) Fatty acid	1/17	27/9784	0.0459285232 625113	0.1911538711 98319	0.1660020460 40645	Oxct1	1
mmu00062	mmu00062	elongation - Mus musculus (house mouse)	1/17	29/9784	0.0492504286 053731	0.1911538711 98319	0.1660020460 40645	Elovl6	1

### Elovl6, Idi1, Sqle, and Stbd1 are potential biomarkers of spinal cord injury

**Figure 3:**
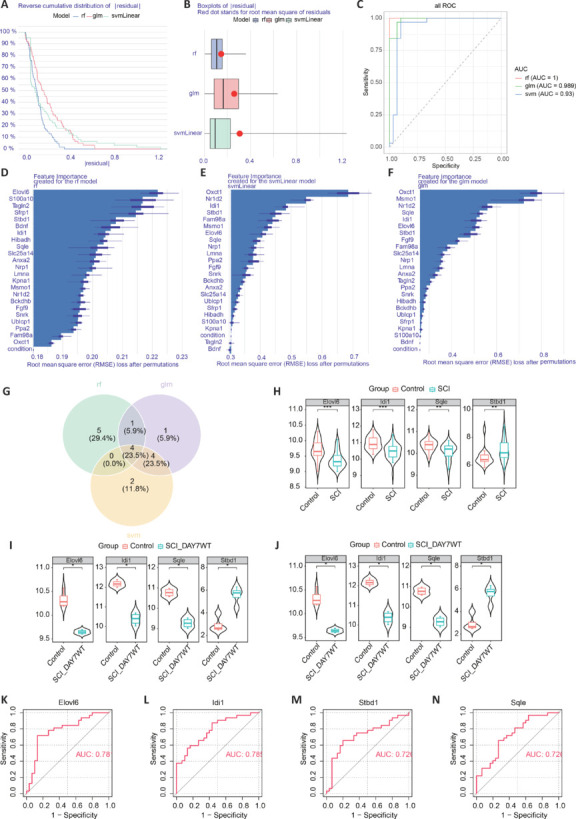
Identification of biomarkers for SCI by machine learning. (A) Reverse cumulative distributions of the absolute residuals for the RF, SVM, and GLM. (B) Box-line plots of residuals for RF, GLM, and SVM. The residuals reflect model prediction errors; boxplots summarize their spread (median, quartiles) and outliers. (C) ROC curves (sensitivity *versus* 1 - specificity) and AUC values quantify model performance in distinguishing SCI-relevant patterns. (D) Top 10 genes predicted by RF, ranked by importance scores from the RMSE. RMSE-derived scores prioritize genes critical for RF’s predictive power in SCI biomarker detection. (E) The top 10 genes predicted by SVM, selected via RMSE-based importance scores. (F) The top 10 genes predicted by the GLM, identified via RMSE-assigned importance. (G) Venn diagram of the top 10 gene intersections across RF, SVM, and GLM. (H) Gene expression of Elovl6, Idi1, Sqle, and Stbd1 in the GSE18179 dataset: SCI *vs.* control groups (Wilcoxon rank-sum test). **P < 0.01, ***P < 0.001 denote statistical significance. (I) Gene expression of Elovl6, Idi1, Sqle, and Stbd1 in GSE47681: SCI *vs*. control. *P < 0.05 indicates significant expression changes (Wilcoxon rank-sum test). (J) Gene expression of Elovl6, Idi1, Sqle, and Stbd1 in GSE42828: SCI *vs.* control (Wilcoxon rank-sum test); *P < 0.05. (K) ROC analysis of Elovl6 as an SCI biomarker in GSE18179. The AUC was used to quantify diagnostic accuracy. (L) ROC analysis of Idi1 as an SCI biomarker in GSE18179. Idi1’s diagnostic performance was evaluated via the AUC, which measures sensitivity/specificity tradeoffs. (M) ROC analysis of Sqle as an SCI biomarker in GSE18179. The AUC is used to gauge Sqle’s efficacy in identifying SCI-associated expression patterns. (N) ROC analysis of Stbd1 as an SCI biomarker in GSE18179. The diagnostic utility of Stbd1 via the AUC was assessed, and its potential as an SCI biomarker was quantified. AUC: Area under the curve; GLM: generalized linear model; RF: random forest; RMSE: root mean square error; ROC: receiver operating characteristic; SCI: spinal cord injury; SVM: support vector machine.

To identify robust biomarkers for SCI, we employed machine learning models to prioritize candidate genes based on their predictive power. The 23 candidate genes were incorporated into RF, SVM, and GLM models. Reverse cumulative distribution of |residual|, box-line plots of residuals, and ROC curve analysis (AUC > 0.9) all demonstrated that the RF, SVM, and GLM models exhibited similarly excellent performance (**Figure 3A–C**). It was therefore concluded that the RF, SVM, and GLM models were the optimal models. The top 10 genes, as predicted by the RF, SVM, and GLM models, were selected based on the importance scores assigned to the candidate genes by root mean square error (**Figure 3D–F**). Subsequently, four feature genes (*Elovl6*, *Idi1*, squalene epoxidase [*Sqle*], and starch-binding domain-containing protein 1 [*Stbd1*]) were identified by determining overlap among the top 10 genes designated by each of the three aforementioned models (**Figure 3G**). Notably, in GSE18179, *Elovl6*, *Idi1*, and *Sqle* exhibited a notable decrease in expression, whereas Stbd1 demonstrated a significant increase in expression, in the SCI group compared with the control group (*P* < 0.05; **Figure 3H**). The expression patterns of *Elovl6*, *Idi1*, *Sqle*, and *Stbd1* in GSE47681 and GSE42828 were consistent with those observed in GSE18179; thus, *Elovl6*, *Idi1*, *Sqle*, and *Stbd1* were defined as candidate biomarkers (**Figure 3I–J**). The AUC values for Elovl6, Idi1, Stbd1, and Sqle exceeded 0.7 (**Figure 3K–N**), indicating that these genes have favorable diagnostic value and might serve as biomarkers. Collectively, these findings establish Elovl6, Idi1, Sqle, and Stbd1 as robust SCI biomarkers, supported by their consistent predictive power across machine learning models, differential expression in multiple datasets, and strong diagnostic accuracy as evidenced by ROC analysis.

### A nomogram based on the identified biomarkers accurately diagnosed spinal cord injury

**Figure 4:**
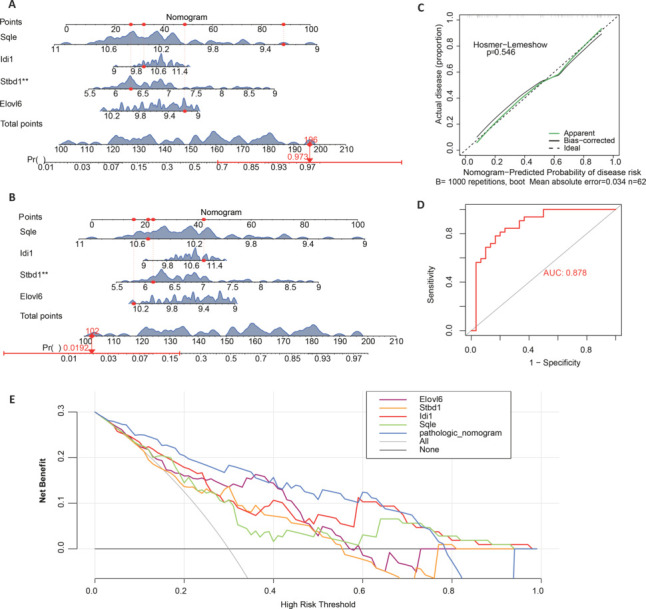
Performance of the nomogram in assessing the diagnosis of SCI. (A) Nomogram showing the maximum point scenario for the Elovl6, Idi1, Sqle, and Stbd1 genes, along with the total point calculation. It visualizes how individual gene–related point values sum to an overall score. (B) Nomogram presenting the minimum point situation for Elovl6, Idi1, Sqle, and Stbd1 plus the total points. The lower-end contributions of these genes to the overall score and the corresponding predicted SCI probability are shown. (C) The correlation between the nomogram-predicted probability of disease risk and actual disease risk. The Hosmer–Lemeshow test was used, with a *P* value of 0.546 (Hosmer–Lemeshow test). (D) ROC curve of the nomogram model for predicting SCI. (E) Decision curve analysis curve of Elovl6, Idi1, Sqle, Stbd1 and the pathologic nomogram. AUC: Area under the curve; ROC: receiver operating characteristic; SCI: spinal cord injury.

To translate the identified biomarkers into a clinical decision-making tool, we developed a nomogram and evaluated its utility for SCI diagnosis. A high nomogram score was associated with an enhanced probability of developing SCI (**Figure 4A** and **B**). Furthermore, the calibration curve exhibited a slope approaching 1 with a P-value of 0.546, indicating that the nomogram had a minimal diagnostic error rate (**Figure 4C**). Additionally, the AUC value of the nomogram was 0.878 (**Figure 4D**), indicating reasonable accuracy in predicting SCI. The DCA results indicated that the nomogram more accurately predicted SCI than any individual biomarker (**Figure 4E**), thereby suggesting its potential to enhance the accuracy of early SCI diagnosis.

### Functional analysis of Elovl6, Idi1, Sqle, and Stbd1

**Figure 5:**
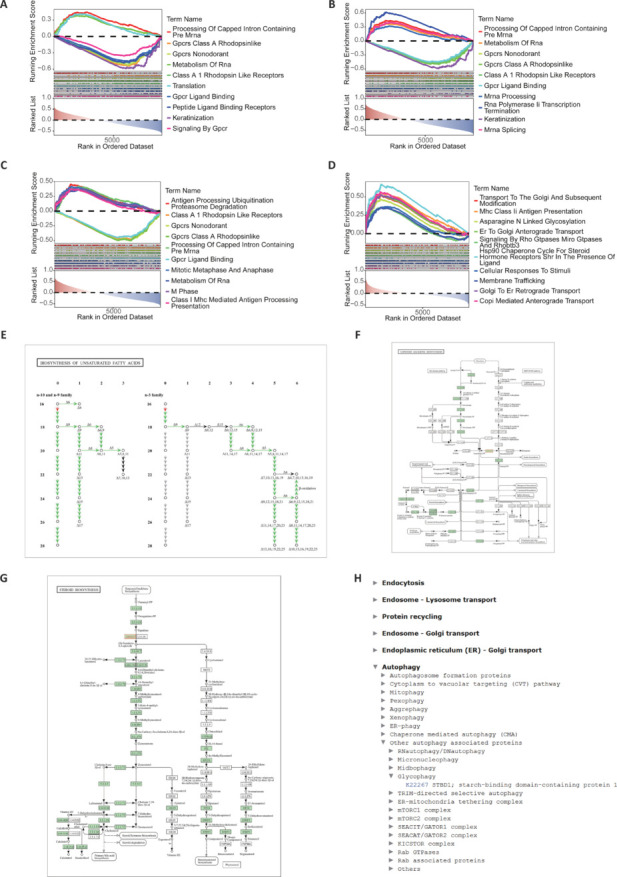
GSEA and KEGG reveal diverse Elovl6-, Idi1-, Sqle-, and Stbd1-related pathways. (A–D) Elovl6-, Idi1-, Sqle-, and Stbd1-related pathways identified via GSEA. (E) KEGG pathway analysis for Elovl6. Illustrates the specific KEGG pathways related to Elovl6 at the molecular interaction and pathway levels, mapping how Elovl6 participates in cellular/physiological processes. (F) KEGG pathways associated with Idi1. The KEGG-annotated pathways involving Idi1 were identified, and the network of molecular interactions and biological pathways centered on Idi1 was visualized. (G) KEGG pathway mapping for Sqle. Displays the KEGG pathways linked to Sqle, highlighting its role in metabolic or signaling pathways at the systems biology scale. (H) KEGG pathways related to Stbd1. The KEGG-defined pathways in which Stbd1 is involved are shown (hypergeometric test and multiple hypothesis testing correction). GSEA: Gene set enrichment analysis; KEGG: Kyoto Encyclopedia of Genes and Genomes.

To explore the mechanistic roles of the biomarkers that we identified for SCI, we performed in-depth functional annotation of Elovl6, Idi1, Sqle, and Stbd1. The functions associated with these biomarkers were then subjected to further investigation. The “G protein-coupled receptors (GPCRs) class A rhodopsinlike”, “GPCRs nonodorant”, “metabolism of RNA”, “class A 1 Rhodopsin like receptors”, and “GPCR ligand binding” pathways were co-enriched in Elovl6, Idi1, and Sqle (*P*_adj_ < 0.05; **Figure 5A–C**). These results suggested that these biomarkers may be involved in GPCR signaling, extracellular signal perception and transmission, and related physiological and pathological processes. Furthermore, Stbd1 was implicated in various biological pathways, such as “transport to the Golgi and subsequent modification,” as well as “MHC Class II antigen presentation” (**Figure 5D**). Additionally, KEGG enrichment analysis revealed that Elovl6 was involved in “biosynthesis of unsaturated fatty acids”, Idi1 in “terpenoid backbone biosynthesis”, and Sqle in “steroid biosynthesis”, and that Stbd1 played a crucial role in “glycophagy” (**Figure 5E–H**). Collectively, these functional analyses linked the four biomarkers to distinct yet interconnected biological processes—ranging from lipid metabolism and GPCR signaling to autophagic pathways and immune regulation—providing mechanistic insights into their roles in SCI and potential therapeutic intervention points.

### Immune cell distribution and correlation with Elovl6, Idi1, Sqle, and Stbd1

**Figure 6:**
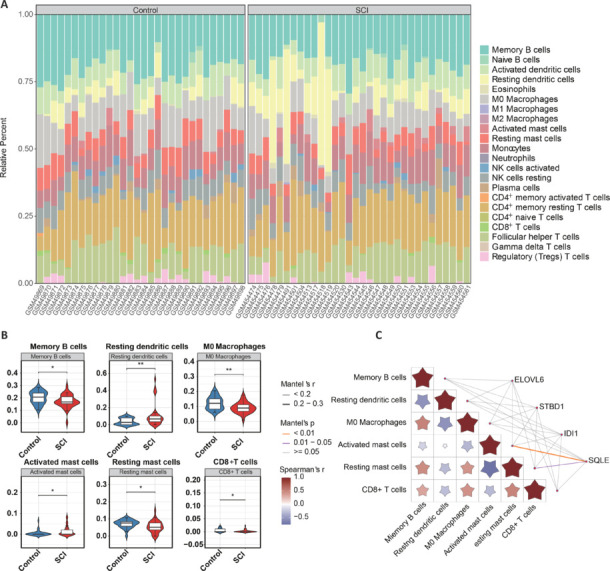
Immune cell distribution and correlation analysis of Elovl6, Idi1, Sqle and Stbd1 in the GSE18179 dataset. (A) A bar chart showing 22 distinct immune cells in the spinal cord before and after injury. (B) Six immune cells that were significantly different between the SCI and control groups (Wilcoxon rank-sum test). *P < 0.05, **P < 0.01. (C) Correlation analysis between biomarkers and differential immune cells (Spearman’s rank correlation test). SCI: Spinal cord injury.

To explore the immunological landscape of SCI and its association with the identified biomarkers, we analyzed immune cell composition and biomarker–immune cell correlations in the immune microenvironment in the SCI and control groups. A stacked bar chart was generated to illustrate the abundance of 22 distinct infiltrating immune cell types (**Figure 6A**). Six immune cell types exhibited significantly different abundance between the SCI and control groups: memory B cells, resting dendritic cells, M0 macrophages, activated mast cells, resting mast cells, and CD8^+^ T cells (*P* < 0.05; **Figure 6B**). The SCI group exhibited increased abundance of resting dendritic cells and activated mast cells and decreased abundance of memory B cells, M0 macrophages, resting mast cells, and CD8^+^ T cells. Notably, Sqle expression was significantly correlated with both activated (*r* = 0.2844, *P* = 0.007) and resting (*r* = 0.1775, *P* = 0.017) mast cells (**Figure 6C** and **Additional Table 8**). Collectively, these results suggest that SCI involves a shift in immune cell distribution and that there is a specific association between Sqle and mast cell activity, implicating lipid metabolic pathways (which Sqle is involved in) in the immunomodulatory processes underlying SCI pathogenesis.

**Additional Table 8:** Correlation analysis between biomarkers and differential immune cells

Spec	Cells	r	P value	rd	pd
ELOVL6	memory B cells	-0.053505258	0.854	< 0.2	≥0.05
ELOVL6	resting dendritic cells	-0.078149074	0.877	< 0.2	≥0.05
ELOVL6	M0 Macrophages	0.0305013419634614	0.275	< 0.2	≥0.05
ELOVL6	activated mast cells	0.0190369786656082	0.383	< 0.2	≥0.05
ELOVL6	resting mast cells	-0.023854499	0.655	< 0.2	≥ 0.05
ELOVL6	CD8 T cells	-0.05881344	0.858	< 0.2	≥0.05
STBD1	memory B cells	-0.018933572	0.552	< 0.2	≥ 0.05
STBD1	resting dendritic cells	0.0303211737572719	0.296	< 0.2	≥0.05
STBD1	M0 Macrophages	-0.02202463	0.616	< 0.2	≥0.05
STBD1	activated mast cells	-0.011017252	0.499	< 0.2	≥0.05
STBD1	resting mast cells	-0.062766864	0.822	< 0.2	≥0.05
STBD1	CD8 T cells	-0.051729568	0.746	< 0.2	≥0.05
IDI1	memory B cells	-0.0736142	0.906	< 0.2	≥0.05
IDI1	resting dendritic cells	-0.112459389	0.972	< 0.2	≥0.05
IDI1	M0 Macrophages	-0.129509942	0.997	< 0.2	≥0.05
IDI1	activated mast cells	0.0959016935129043	0.113	< 0.2	≥0.05
IDI1	resting mast cells	0.0479682734995395	0.222	< 0.2	≥0.05
IDI1	CD8 T cells	-0.104009594	0.955	< 0.2	≥0.05
SQLE	memory B cells	-0.041613989	0.711	< 0.2	≥0.05
SQLE	resting dendritic cells	-0.10085489	0.949	< 0.2	≥0.05
SQLE	M0 Macrophages	-0.089738389	0.931	< 0.2	≥ 0.05
SQLE	activated mast cells	0.284441101336461	0.007	0.2 - 0.3	< 0.01
SQLE	resting mast cells	0.177476689824929	0.017	< 0.2	0.01 - 0.05
SQLE	CD8 T cells	-0.044063358	0.673	< 0.2	≥0.05

### Elovl6, Idi1, Sqle, and Stbd1 regulatory mechanisms, potential targeting drugs, and molecular docking analysis

**Figure 7:**
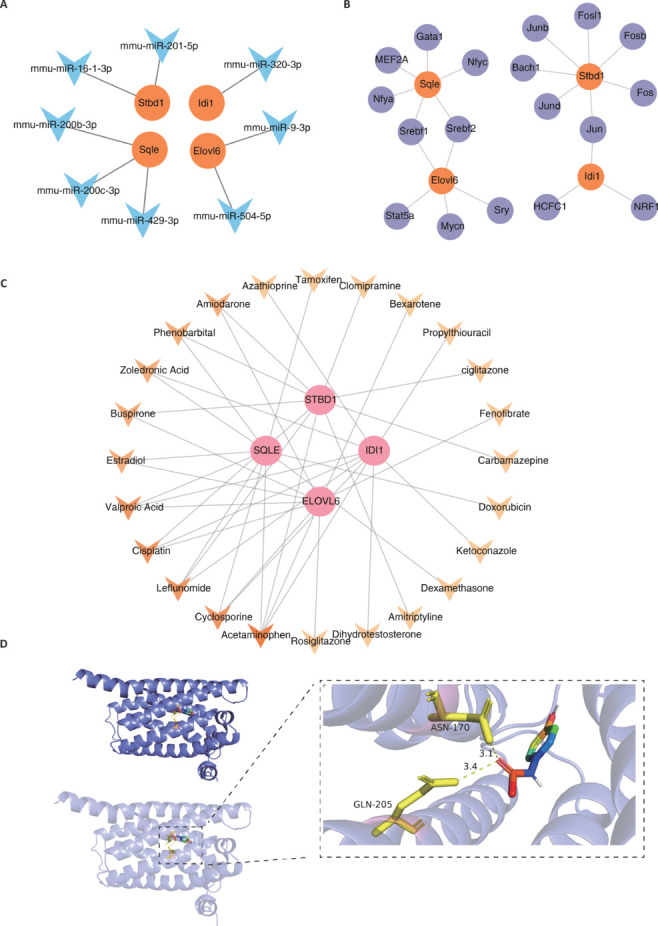
Analysis of the regulatory mechanisms and potential drug intervention targets related to Elovl6, Idi1, Sqle, and Stbd1.. (A) miRNA‒mRNA regulatory network of biomarkers. (B) TF‒mRNA regulatory network of biomarkers. (C) Drug prediction of Elovl6, Idi1, Sqle and Stbd1, with a total of 24 drugs and acetaminophen being a copredicted compound. (D) Molecular docking between Elovl6 and acetaminophen with two binding proteins: ASN-170 and GLN-205. miRNA: MicroRNA; TF: transcription factor.

To investigate the regulatory landscape of SCI biomarkers and explore therapeutic opportunities, we integrated molecular regulation, drug prediction, and binding kinetics analyses. To gain further insight into biomarker regulatory factors, a molecular regulatory network was constructed. The intersection of the predicted biomarker–miRNA relationship pairs from the miRanda and miRDB databases yielded eight key miRNAs, such as mmu-miR-201-5p (**Figure 7A**). Furthermore, Elovl6, Idi1, Sqle, and Stbd1 were predicted to be regulated by five, three, six, and seven TFs, respectively (**Figure 7B**). Notably, Elovl6 and Sqle were both predicted to be regulated by TFs Srebf1 and Srebf2, while Idi1 and Stbd1 were both predicted to be regulated by Jun.

We identified 24 potential drugs that might regulate Elovl6, Idi1, Sqle, and Stbd1, including acetaminophen, which was predicted to regulate all four (**Figure 7C**). To assess potential binding between the predicted drugs and key proteins, we performed molecular docking analyses. Acetaminophen was selected as the key drug. The free energies of acetaminophen binding to Elovl6, Idi1, Sqle, and Stbd1 were −6.3 kcal/mol, −6.1 kcal/mol, −6.1 kcal/mol, and −5.4 kcal/mol, respectively. A binding energy threshold of ≤ −5 kcal/mol indicated robust binding affinity between acetaminophen and Elovl6, Idi1, Sqle, and Stbd1. Elovl6 binding to acetaminophen exhibited the highest binding free energy, and thus this protein was selected for further investigation as a representative example. Molecular docking analysis of Elovl6 binding to acetaminophen identified two binding proteins: ASN-170 and GLN-205 (**Figure 7D**). Acetaminophen binding to Elovl6 was predominantly facilitated by hydrogen bond interactions. Collectively, these findings establish acetaminophen as a promising candidate for regulating SCI biomarker activity, supported by its robust binding affinity and specific interaction sites, suggesting a potential therapeutic role in modulating SCI-related molecular pathways.

### Elovl6, Idi1, Sqle, and Stbd1 expression during spinal cord injury progression

To experimentally validate our computational predictions from the scRNA-seq analysis, we experimentally characterized biomarker expression dynamics in a mouse model of SCI. The experiment included 20 mice, with a male-to-female ratio of 1:1. Three mice of each sex were assigned to the control group, and the remaining mice were subjected to SCI. One mouse of each sex died because of anesthesia-related complications, resulting in a survival rate of 85.71%. On the basis of macroscopic observation, BMS scores, and immunofluorescence staining, the remaining SCI mice were deemed suitable for subsequent use (**Additional Figure 1**).

**Figure 8:**
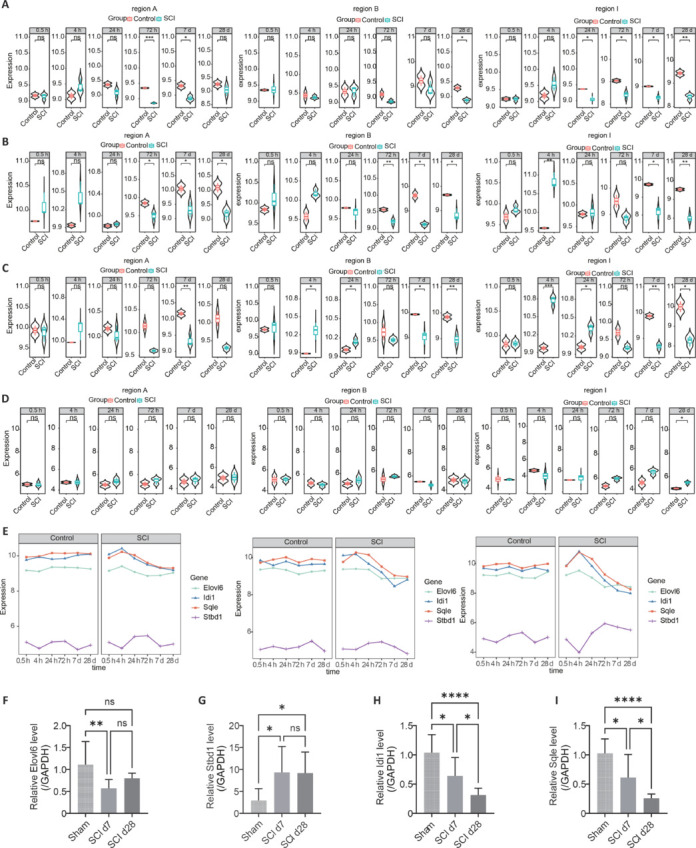
Spatiotemporal expression of Elovl6, Idi1, Sqle, and Stbd1 genes during the process of spinal cord injury (SCI). (A–D) Expression of Elovl6, Idi1, Sqle, and Stbd1 in three different regions between the SCI group and the control group (independent samples *t*-test). (E) Expression trends of Elovl6, Idi1, and Sqle over time in the SCI group ((independent samples *t*-test). (F) Bar graph of relative Elovl6 expression levels. Relative expression of Elovl6 normalized to that of GAPDH in different groups (sham, SCI_d7, SCI_d28). (G) Bar graph of the relative Stbd1 expression level. Relative expression of Stbd1 normalized to that of GAPDH across groups (sham, SCI_d7, SCI_d28). (H) Bar graph of the relative Idi1 expression level. The data are presented as the relative expression of Idi1 normalized to that of GAPDH in the different groups (sham, SCI_d7, SCI_d28). (I) Bar graph of the relative Sqle expression level. The relative expression of Sqle was normalized to that of GAPDH across groups (sham, SCI_d7, SCI_d28). *P < 0.05, **P < 0.01, ****P < 0.0001. GAPDH: Glyceraldehyde-3-phosphate dehydrogenase; ns: not significant; SCI: spinal cord injury.

Notable discrepancies in Elovl6 expression were observed between the SCI and sham-injury control groups at 72 hours and 7 days in region A, at 28 days in region B, and at 24 hours, 72 hours, 7 days, and 28 days in region I (*P* < 0.05; **Figure 8A**). Significant discrepancies in Idi1 expression were observed between the SCI and sham-injury control groups at 72 hours, 7 days, and 28 days in regions A and B, as well as at 4 hours, 7 days, and 28 days in region I (*P* < 0.05; **Figure 8B**). Furthermore, significant discrepancies in Sqle expression were observed between the SCI and sham-injury control groups at 7 days in region A and at 4 hours, 24 hours, 7 days, and 28 days in regions B and I (*P* < 0.05; **Figure 8C**). Stbd1 expression remained unaltered at all six time points in regions A and B; the only significant difference was observed at 28 days in region I (*P* < 0.05; **Figure 8D**). Elovl6, Idi1, and Sqle expression generally demonstrated a declining trend over time in regions A, B, and I in the SCI group (**Figure 8E**). A control experiment showed that all samples exhibited RNA concentrations within the standard range (**Additional Table 9**). Comparison of the sham group with the SCI 7 dpi group revealed a significant reduction in Elovl6 expression (*P* < 0.01; **Figure 8F**). Conversely, both the SCI d7 and SCI d28 groups exhibited a notable elevation in Stbd1 expression compared with the sham group (*P* < 0.05; **Figure 8G**). Additionally, the SCI 7 dpi and SCI 28 dpi groups demonstrated a pronounced decline in Idi1 and Sqle expression levels compared with the sham group (*P* < 0.05; **Figure 8H** and **I**). Idi1 and Sqle expression levels were significantly reduced in the SCI 28 dpi group in comparison to the SCI 7 dpi group (*P* < 0.05). Collectively, these findings validate the differential expression of Elovl6, Idi1, Sqle, and Stbd1 in SCI mouse tissues, in agreement with the bioinformatics predictions, and establish their temporal roles in SCI progression from the acute to the chronic stage.

**Additional Table 9:** The RNA concentration of samples

Number	Group	Concentration (ng/μL)	A260/A280	A260/A230
1	Sham (Male)	561.68	1.969	1.866
2	Sham (Male)	794.96	1.961	2.061
3	Sham (Male)	489.90	1.787	1.039
4	SCl d7 (Male)	376.08	2.017	1.962
5	SCl d7 (Male)	744.68	1.910	1.669
6	SCl d7 (Male)	326.32	1.874	1.349
7	SCI d28 (Male)	326.32	1.874	1.349
8	SCI d28 (Male)	470.04	1.654	0.532
9	SCI d28 (Male)	530.88	1.561	0.590
10	Sham (Female)	350.98	1.714	2.109
11	Sham (Female)	567.18	1.878	2.116
12	Sham (Female)	334.9	1.773	1.998
13	SCl d7 (Female)	778.14	2.09	2.111
14	SCl d7 (Female)	540.15	1.809	1.816
15	SCl d7 (Female)	480.26	1.706	2.001
16	SCI d28 (Female)	500.67	2.078	1.982
17	SCI d28 (Female)	517.24	1.992	2.789
18	SCI d28 (Female)	809.15	1.988	2.076

### Microglia and T cells are key cells in spinal cord injury progression

**Figure 9:**
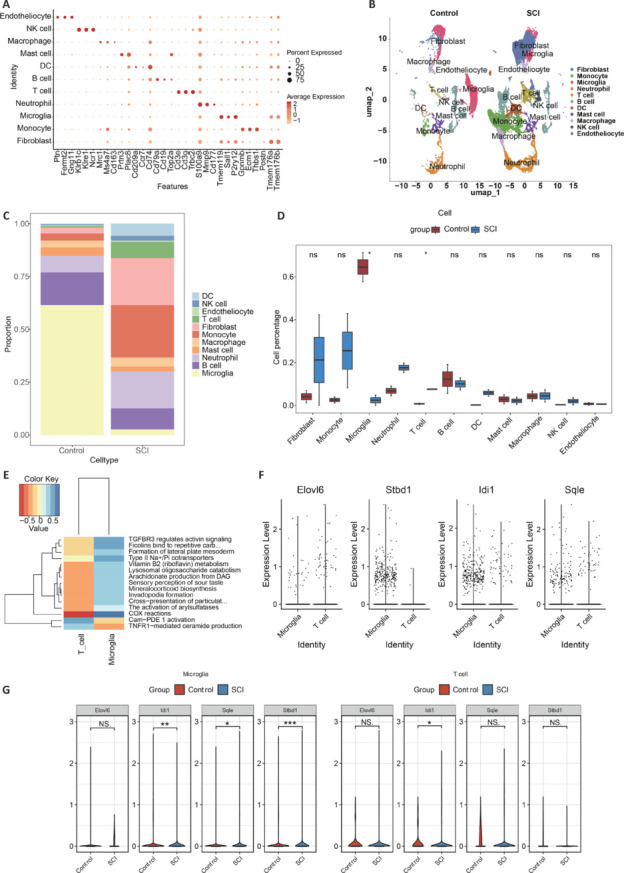
Microglia and T cells identified as key cells in the SCI and control groups. (A) Dot plot showing the DEGs in each cluster on the basis of adjusted *P* values. The dot color indicates the average scaled RNA expression of that gene in the cell type, and the dot size represents the percentage of cells in the cluster that express that gene. (B) UMAP plot showing eleven major cell types pooled from all groups. Each dot represents a single cell. (C) Stacked bar chart illustrating the fractional distribution of 11 cell subsets in the SCI *versus* control groups. Shows how the proportion of each cell type changes after injury, highlighting shifts in the cellular composition. (D) Box plot comparing the percentages of 11 cell subsets between the SCI and control groups. The variability and significance of cell type proportion changes were quantified (independent samples *t*-test). (E) Different pathways in which microglia and T cells might be enriched (hypergeometric test). (F) Expression of Elovl6, Stbd1, Idi1, and Sqle in microglia and T cells. (G) Expression of Elovl6, Idi1, Sqle and Stbd1 in microglia and T cells before and after SCI (Wilcoxon rank-sum test). *P < 0.05, **P < 0.01, ***P < 0.001. DEG: Differentially expressed gene; NS/ns: not significant; SCI: spinal cord injury; UAMP: uniform manifold approximation and projection.

To identify cell types driving SCI pathogenesis, we performed single-cell RNA-seq analysis to characterize cellular heterogeneity and differential abundance. Before the scRNA-seq data were subjected to QC analysis, the initial count comprised 31,622 cells and 19,282 genes. Following QC, the cell count was refined to 30,022, while the gene count remained constant at 19,282 (**Additional Figure 2A** and **B**). Standard data processing led to the identification of a subset of 2000 hypervariable genes (**Additional Figure 2C**). The top 30 PCs were selected for subsequent analysis, as illustrated in **Additional Figure 2D**. Most of the *P*-values associated with the top 30 genes were below 0.05 (**Additional Figure 2E**), indicating high statistical significance. UMAP cluster analysis delineated 25 different cell clusters (**Additional Figure 2F**). The marker genes expressed by each cell type are shown by bubble plot and heatmap (**Figure 9A** and **Additional Figure 2G**). The marker genes of the various cell types are listed in **Table 1**. A total of 11 cell types were identified by their marker genes, including fibroblasts, monocytes, microglia, neutrophils, T cells, B cells, dendritic cells, mast cells, macrophages, natural killer (NK) cells, and endotheliocytes (**Figure 9B**). The SCI group exhibited a higher percentage of neutrophils, monocytes, fibroblasts, T cells, and DCs than the control group, while the percentages of microglia and B cell were lower (**Figure 9C**). The most marked alterations in cell type abundance in the SCI group were the decreased microglia and increased T cells, establishing these two cell types as highly differentially abundant cells (**Figure 9D**). Moreover, microglia and T cells might be enriched in a number of different pathways affected by SCI; for example, TGFBR3 regulates activin signaling (**Figure 9E**). Further analysis demonstrated that Elovl6 was uniformly expressed in microglia and T cells, whereas Idi1, Sqle, and Stbd1 were predominantly expressed in microglia (**Figure 9F**). Changes in microglial Idi1, Sqle, and Stbd1 expression were observed between the SCI and control groups (**Figure 9G**). Additionally, there was a substantial difference in Idi1 expression in T cells between these two groups. Consequently, microglia and T cells were identified as key cells in SCI. Thus, scRNA-seq analysis identified microglia and T cells as key cellular players in SCI and linked their dysregulation to biomarker expression and signaling pathways, which might mediate their roles in SCI pathogenesis and progression.

### Analysis of microglia and T cell communication

**Figure 10:**
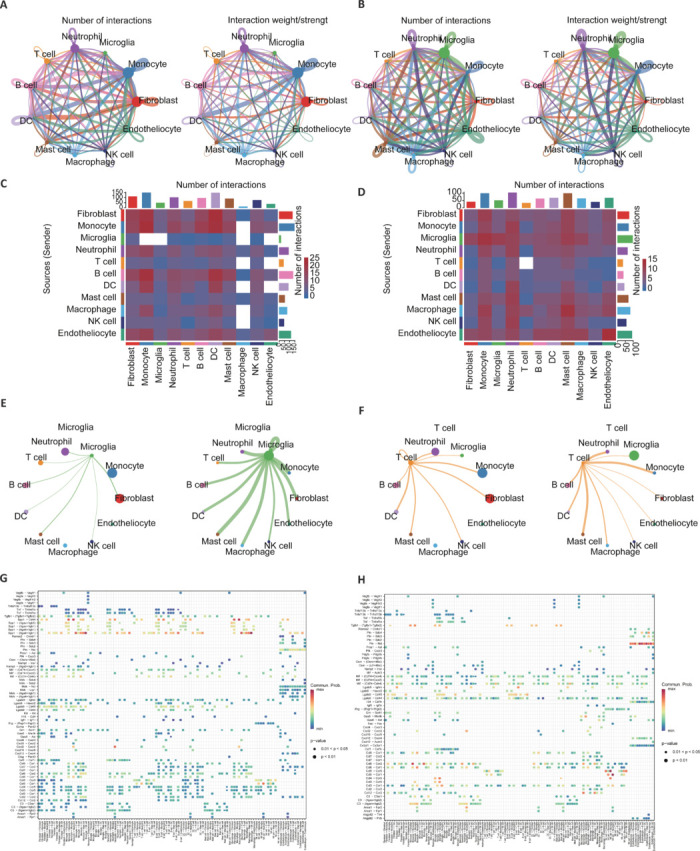
Cellular communication analysis of microglia and T cells before and after SCI. (A) Cellular communication network among 11 annotated cell types in the control group. Nodes represent cell types, and edges indicate interactions; visualizes the baseline (preinjury) communication patterns. (B) Cellular communication network among 11 annotated cell types in the SCI group. (C) Heatmap of ligand‒receptor and receptor‒ligand interactions among 11 cell types in the control group. The color intensity and values reflect the frequency/strength of these molecular interaction pairs at baseline. (D) Heatmap of ligand–receptor and receptor–ligand interactions among 11 cell types in the SCI group. (E) Communication strength analysis of microglia and T cells in the control group. The magnitude of interactions centered on these key cell types under normal conditions is quantified and visualized. (F) Communication strength analysis of microglia and T cells in the SCI group. Shows how spinal cord injury impacts the interaction strength of these cell types with other populations. (G) Heatmap “hot plot” of ligand‒receptor and receptor‒ligand interactions among 11 cell types in the control group. Highlights prominent interaction patterns and key molecular pairs in preinjury cellular communication (hypergeometric test). (H) Heatmap “hot pot” of ligand–receptor and receptor–ligand interactions among 11 cell types in the SCI group. Injury-associated changes in molecular interaction hotspots between cell types (hypergeometric test) were identified. DC: Dendritic cell; SCI: spinal cord injury.

Next, we sought to systematically elucidate how interactions between neural cells and immune cells are remodeled in the SCI microenvironment. Cellular communication analysis revealed complex interrelationships among the 11 annotated cell types. In the SCI group, T cells, neutrophils, monocytes, fibroblasts, and other cells exhibited a high frequency and intensity of interactions (**Figure 10A**). In contrast, the control group displayed a high frequency and intensity of interactions among microglia and other cells (**Figure 10B**). Additionally, the SCI group exhibited a diminished number of ligand–receptor and receptor–ligand interactions between microglia, macrophages, endothelial cells, and other cells in comparison to the control group (**Figure 10C** and **D**). Microglial communication strength was weaker in the SCI group than in the control group (**Figure 10E**). Conversely, T cells demonstrated stronger communication strength in the SCI group than in the control group (**Figure 10F**). In the SCI group, the probability of Spp1–Cd44 interactions was significantly higher in microglia than in other cell types, while that of Spp1–(Itga4+Itgb1) interactions was significantly higher in T cells (**Figure 10G**). In the control group, microglia demonstrated a markedly elevated probability of Ccl4–Ccr5, Ccl3–Ccr5, and Ccl3–Ccr1 interactions, whereas T cells exhibited a notably high probability of Ccl3–Ccr1 interaction (**Figure 10H**). These findings provide insight into how “neuro-immune synapses” are remodeled after SCI and present a theoretical foundation for developing spatiotemporal regulation strategies targeting cell–cell interactions.

### Pseudo-temporal trajectory analysis of microglia and T cells

**Figure 11:**
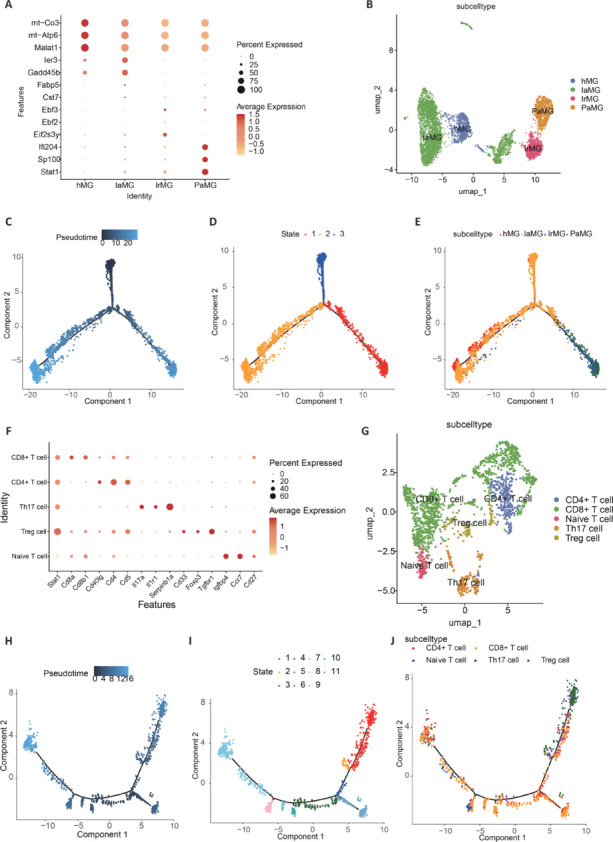
Pseudo-temporal trajectory analysis of microglia and T cells. (A) Dot plot of gene expression features in microglial subsets. (B) UMAP visualization of microglial subsets. Different colors represent microglial subsets, showing the distribution pattern of each subset in the low-dimensional space. (C) Pseudotemporal trajectory of microglia (identity dimension). Pseudotime is taken as the axis (the color gradient corresponds to “pseudotime”). The distribution of microglia along the differentiation trajectory reflects the direction of cell differentiation. (D) Pseudotemporal trajectory of microglia (state dimension). (E) Pseudotemporal trajectory of microglial subsets. On the basis of microglial subsets (hMGs, IaMGs, IrMGs, and PaMGs), a pseudotemporal trajectory is used to show the distribution and differentiation relationship of each subset along the differentiation path. (F) Dot plot of gene expression features in T-cell subsets. The percentage (dot size) and average expression (dot color) of genes across five T-cell subsets (e.g., CD8^+^ T cells and CD4^+^ T cells) reflect the gene expression characteristics of different T-cell subsets. (G) Different colors represent T-cell subsets (e.g., CD4^+^ T cells and CD8^+^ T cells), indicating the distribution of each subset in the low-dimensional space. (H) Pseudotemporal trajectory of T cells (pseudotemporal dimension). With pseudotime as the basis (the color gradient corresponds to “pseudotime”), the distribution of T cells along the differentiation trajectory is shown, demonstrating the differentiation path of T cells. (I) Pseudotemporal trajectory of T cells (state dimension). Pseudotemporal trajectory analysis of T cells according to cell state (States 1–11) was performed. Distribution and evolution of cells in different states during the differentiation process. (J) Pseudotemporal trajectory of T-cell subsets.

To elucidate the differentiation patterns of neuroimmune cells after SCI, we performed a comprehensive pseudo-temporal trajectory analysis of microglia and T cells. Microglia were categorized into six subpopulations and designated as one of four distinct cell subtypes: homeostatic microglia (hMG), interferon-responsive microglia (IrMG), injury-associated microglia (IaMG), and proliferation-associated microglia (PaMG) (**Additional Figure 3A** and **Figure 11A** and **B**). Microglial differentiation along the cell trajectory is illustrated in **Figure 11C**, which depicts the progressive differentiation of cells from left to right over time, with the darkest blue representing the earliest differentiated cells. Further analysis revealed that microglia differentiation occurs in three distinct stages (states 1, 2, and 3). Each stage is characterized by multiple cell subtypes, with stage 1 signifying the initial phase of differentiation (**Figure 11D** and **E**). Similarly, T cells were divided into seven subpopulations and classified into five cell subtypes: CD4^+^ T cells, CD8^+^ T cells, naive T cells, Th17 cells, and regulatory T (Treg) cells (**Additional Figure 3B** and **Figure 11F** and **G**). T cells progressed sequentially from their initial developmental stage into different branches, encompassing 11 distinct developmental states, with multiple subpopulations within each state (**Figure 11H–J**). The expression levels of the four biomarkers (Elovl6, Idi1, Sqle, and Stbd1) remained constant over time in both microglia and T cells (**Additional Figure 3C** and **D**). The expression levels of the four key genes changed during two key cell differentiation processes in the pseudotime analysis (**Additional Figure 4A** and **B**). Idi1 and Sqle expression levels first increased and then decreased during cell differentiation, while Elovl6 and Stbd1 expression increased, then decreased, and then increased again. The pseudotime analysis results were consistent with the time-series expression results from dataset GSE5296 and the qRT-PCR results (**Figure 8**), providing single-cell insight into the dynamic regulation of neuroimmune mechanisms.

To understand the gene expression changes experienced by each cell during the cell-state transition process in the dataset, we performed dimensionality reduction and clustering analysis of microglia. On the basis of this analysis, the microglia were re-clustered into six subgroups and annotated as one of two cell subtypes: M1_MGs and M2_MGs (**Additional Figure 5A** and **B**). Next, to understand the key gene expression changes experienced by each cell during the cell-state transition process in the scRNA-seq dataset, cell pseudotime trajectory analysis of these key cell clusters was conducted. The results showed that microglia differentiate via three different developmental stages. Multiple cell clusters were present during each developmental stage (**Additional Figure 5C–E**). In the early stage of cell differentiation, the proportion of M1_MGs was higher than that of M2_MGs (**Additional Figure 5F–I**). These results suggested that microglial polarization after SCI is time-dependent.

## Discussion

SCI is characterized by its high incidence and significant disability rates, posing substantial challenges to both diagnosis and treatment (Cao et al., 2025; Chen et al., 2025a). Despite advancements in SCI treatment and rehabilitation over the past decades, therapeutic outcomes remain unsatisfactory. RNA plays a pivotal role in biological processes and is essential for cellular functions. RNA modification, a key regulator of RNA biological activity, is closely associated with the pathogenesis of various diseases, including SCI.

Characterizing RNA modifications and exploring associated signaling pathways and biomarkers could thus provide promising strategies for SCI treatment and rehabilitation. In this study, public RNA-seq datasets were analyzed to identify four signature genes (*Elovl6*, *Idi1*, *Sqle*, and *Stbd1*) associated with RNA modification in SCI using WGCNA, machine learning algorithms, ROC analysis, and quantitative real-time PCR.

Elovl6, a member of the very-long-chain fatty acid gene family (ELOVLs), catalyzes the elongation of saturated and monounsaturated fatty acids and is a key component of fatty acid elongation and acyl CoA biosynthesis (Guo et al., 2024). Elovl6 is vital for fatty acid metabolism, oxidative stress, inflammatory responses, and other metabolic diseases (Matsuzaka and Shimano, 2009; Cui et al., 2024). Elovl6 upregulation has been linked to the synthesis of long-chain ceramides in plasma, which disrupt lipid metabolism in the CNS immune microenvironment, potentially inducing autoimmune encephalomyelitis (Schmitz et al., 2021). Furthermore, Elovl6 has been identified as a lipid metabolism biomarker in Parkinson’s disease (PD) (Wang et al., 2024a). In our study, we found that Elovl6 expression was significantly downregulated in the SCI group compared with controls. Although the precise role of reduced Elovl6 expression remains unclear, previous research suggests that its downregulation impairs macrophage lipid metabolism, contributing to atherosclerosis (Saito et al., 2011). On this basis, we hypothesized that decreased Elovl6 expression may affect the immune microenvironment, especially macrophage function, after SCI. This finding sheds new light on the immune response after SCI and suggests potential therapeutic intervention, which has important clinical implications.

The *IDI1* gene encodes a peroxisome-localizing enzyme with specific catalytic roles across various organisms. IDI1 facilitates the interconversion of isopentenyl diphosphate and its electrophilic isomer, dimethylallyl diphosphate, both of which serve as precursors for farnesyl diphosphate and cholesterol synthesis (Chen et al., 2015; Spann et al., 2017; Fan et al., 2024). Recent studies highlight the role of IDI1 in promoting myelin formation and outgrowth via the interaction of IDI1 with the YAP/TAZ-TEAD1 transcription complex, which is associated with enhanced cholesterol synthesis in Schwann cells (Grove et al., 2024). This relationship underscores the importance of IDI1 in facilitating myelination. Our observation that IDI1 expression is downregulated at 7 and 28 dpi after SCI suggests that diminished IDI1 activity may contribute to irreversible myelin sheath damage. These results provide new insights into the role of IDI1 in myelin repair and the implications of IDI1 for SCI pathophysiology.

Sqle is another critical metabolism-related protein identified in our study as playing a role in SCI pathogenesis. Sqle also plays a pivotal role in cholesterol and fatty acid biosynthesis. This enzyme catalyzes the conversion of squalene to lanosterol, a key intermediate in the cholesterol synthesis pathway (Chua et al., 2020). After SCI, Sqle downregulation and decreased cholesterol biosynthesis may signify acute loss of myelinated axons or an adaptive response to injury (Forston et al., 2023). This aligns with the findings from our study. Such metabolic reprogramming could also contribute to oligodendrocyte apoptosis in the subacute injury phase (Pukos et al., 2019; Duncan et al., 2020).

Stbd1 is defined by its starch-binding activity, but its biological function has not been clearly characterized. It is predominantly involved in glycophagy and intracellular transport (Kyriakoudi et al., 2022; Tang et al., 2023). Glycophagy, a glycogen-selective autophagy process, is increasingly recognized as a crucial metabolic pathway for the transport and delivery of glycolytic substrates. Dysregulated glycogen metabolism is closely associated with the pathophysiology of various metabolic diseases in tissues such as the liver, skeletal muscle, myocardium, and brain (Koutsifeli et al., 2022). In neurons, impaired glucose metabolism following SCI may trigger glycophagy (Li et al., 2023). Our analysis indicates that Stbd1 expression is significantly upregulated after SCI, likely correlating with enhanced neuronal glycogenesis.

Metabolic reprogramming of CNS cells and components after SCI is a well-recognized phenomenon (Li et al., 2020; Hakim et al., 2021; Tan et al., 2022). Elovl6, Idi1, Sqle, and Stbd1 are mainly involved in lipid metabolism and glucose metabolism, affecting many signaling pathways. Previous studies have demonstrated that dysregulation of lipid metabolism can modulate the release of inflammatory mediators, thereby influencing microglia and macrophage polarization and subsequently regulating neuroinflammatory responses (Du et al., 2025). Additionally, pathways such as the steroid biosynthesis and fatty acid elongation pathways may indirectly participate in axon regeneration and neuronal survival by affecting cell membrane structure and signal transduction (Xing et al., 2011). Although KEGG pathway analysis did not reveal a direct association of these genes with inflammation or nerve regeneration, previous studies have supported the multifaceted roles of metabolism-related genes in neurological diseases. For instance, Elovl6 has been identified as a lipid metabolism biomarker in Parkinson’s disease, and changes in its expression are closely related to neuroinflammation and oxidative stress (Wang et al., 2024a). Idi1, by regulating cholesterol synthesis, promotes myelination and axon regeneration (Grove et al., 2024). These studies suggest that metabolism-related genes may indirectly influence the core pathological mechanisms of SCI by modulating the microenvironment.

In the present study, Elovl6, Idi1, and Sqle were all enriched in pathways such as “GPCRs, Class A (Rhodopsin-like)”, “GPCRs, Class A (Rhodopsin-like), “GPCRs nonodorant”, “RNA metabolism”, “Class A1 (Rhodopsin-like) receptors”, and “GPCR ligand binding”. GPCRs are widely distributed in the cytoplasm, nucleus, and organelles (Poniatowski et al., 2017). As the largest and most diverse family of cell surface receptors, GPCRs provide versatile scaffolds for programming cellular responses, including through synthetic receptors. These seven-transmembrane domain proteins mediate responses to a variety of extracellular signals, such as hormones, neurotransmitters, peptides, light, mechanical forces, and odors. Ligand binding induces conformational changes in GPCRs, activating heterotrimeric G proteins and initiating downstream intracellular signaling cascades (Kalogriopoulos et al., 2025). The role of GPCR signaling in SCI is well established and extensively studied. For instance, GPCR kinase 2-interacting protein-1 has been shown to protect against spinal cord ischemia-reperfusion injury by modulating the ASK1/JNK/p38 signaling pathway (Chen et al., 2018). Additionally, GPCR kinase 2-interacting protein-1 exerts neuroprotective effects by promoting Beclin1-Parkin–induced mitophagy during the early stages of spinal cord ischemia-reperfusion injury (Huang et al., 2020). Additionally, GPCR signaling significantly contributes to neuropathic pain management following SCI. For example, expression of the orphan GPCR-Gpr160 is upregulated in the dorsal horn of the rodent spinal cord after traumatic nerve injury (Yosten et al., 2020). Similarly, deletion of the GPR34 gene attenuates nerve injury–induced neuropathic pain by suppressing pro-inflammatory microglial responses, without altering their morphology (Sayo et al., 2019). In conclusion, Elovl6, Idi1, Sqle, and Stbd1 may play significant roles in SCI pathogenesis by acting on the GPCR signaling pathway.

RNA metabolism is a comprehensive process encompassing RNA processing, RNA decay, and RNA stability (Houseley and Tollervey, 2009; Matsui et al., 2019). RNA modifications significantly influence RNA metabolism and have been shown to regulate a variety of diseases, including SCI. Injury is often accompanied by persistent local inflammation, resulting in severe secondary damage, including neuronal apoptosis and spinal cord demyelination (David and Kroner, 2011). Importantly, RNA modifications play a crucial role in modulating the inflammation associated with SCI. m^6^A methylation of miRNA and long non-coding RNAs is vital for the regulation of neuronal apoptosis and myelin regeneration (Guo et al., 2023). Our study identified eight miRNAs associated with Elovl6, Idi1, Sqle, and Stbd1, revealing potential links between RNA modification and SCI outcomes. miR-201-5p is a key differentially expressed miRNA in spinal cord ischemia-reperfusion injury (Li et al., 2016). Fat mass and obesity-associated protein inhibits ferroptosis through the miR-320-3p/SLC7A11 axis in an m6A-dependent manner, thus protecting against cerebral ischemia-reperfusion injury (Peng et al., 2024). miR-200b-3p expression is increased in primary hippocampal neurons after oxygen-glucose deprivation *in vitro*. Inhibition of miR-200b-3p can prevent neuronal apoptosis and promote neuronal viability (Zhang et al., 2023). miR-9-3p, which is expressed in the hippocampus, promotes neuronal function, thereby enhancing learning and memory (Sim et al., 2016). No studies have yet explored the role of miR-504-5p, miR-200c-3p, and miR-16-1-3p in CNS diseases. However, the potential regulatory effects of RNA modifications in the CNS underscore the need for further investigation. Additional experimental research is needed to elucidate the precise mechanisms and therapeutic applications of RNA modifications in SCI and related conditions.

Recently, it has been discovered that a variety of small-molecule drugs can influence RNA modification status by regulating RNA modification–related enzymes (such as methyltransferases and demethylases), thereby participating in epigenetic regulation (Dai et al., 2024). For example, fat mass and obesity-associated protein inhibitors (such as MA2) can increase the level of m^6^A modification, thereby inhibiting tumor cell proliferation and migration (Wen et al., 2020). Additionally, small-molecule inhibitors of the METTL3/METTL14 complex (e.g., STM2457) can reduce the level of m^6^A modification, affecting mRNA stability and translation efficiency (Su et al., 2024). These studies indicate that drugs targeting RNA modifications play an important role in epigenetic regulation. In the present study, we identified small-molecule drugs predicted to regulate Elovl6, Idi1, Sqle, and Stbd1 and found that acetaminophen was predicted to regulate all four. Previous studies have shown that acetaminophen can affect gene expression by regulating inflammation-related pathways (e.g., the nuclear factor kappa B pathway) (Chen et al., 2024), but whether it exerts its effects through RNA modification needs to be further verified. In combination with the reported RNA modification–related drugs in the literature, future research can further explore the potential of these drugs to regulate RNA modifications in SCI, providing a theoretical basis for the development of new therapeutic strategies.

SCI is accompanied by destruction of the blood–spinal cord barrier, allowing infiltration of peripheral immune cells and blood cells into the CNS (Jin et al., 2021a). These peripheral immune cells play a crucial role in both neuroinflammation and the reconstruction of neural function following SCI. As early responders to injury, activated neutrophils clear cellular and tissue debris through phagocytosis while releasing cytokines that recruit additional neutrophils, monocytes, and macrophages (Zivkovic et al., 2021). Recently, T cells have also gained significant attention for their roles in the CNS. Tregs facilitate spinal cord repair by influencing macrophage and microglia polarization, activating reactive astrocytes, and promoting axonal formation by oligodendrocytes (Chen et al., 2023). Dendritic cells, the most potent antigen-presenting cells in the CNS, play a beneficial role in SCI. They initiate T cell differentiation and activate microglia and astrocytes, which in turn secrete neurotrophic factors and modulate the microenvironment, producing anti-inflammatory and pro-repair effects at the injury site (Han et al., 2024). Our analysis revealed significant changes in the abundance of memory B cells, resting dendritic cells, M0 macrophages, activated mast cells, resting mast cells, and CD8^+^ T cells after SCI, highlighting extensive immune infiltration at the injury site. Notably, Sqle expression exhibited a significant correlation with the abundance of both activated and resting mast cells. Mast cells are particularly intriguing in SCI due to their distinct morphology, which is characterized by abundant electron-dense lysosomal-like secretory granules that occupy much of their cytoplasm. These granules store preformed and preactivated mediators, including interleukin-4, nerve growth factor, and mast cell–specific proteases such as tryptase, rennin, and carboxypeptidase A (Pejler et al., 2010; Lundequist and Pejler, 2011). Mast cells exert protective effects on the CNS after trauma through various mechanisms. For instance, mouse mast cell protease 4 cleaves pro-inflammatory mediators, thereby suppressing harmful inflammation, while mouse mast cell protease 6 reduces scar tissue formation (Vangansewinkel et al., 2023). Further investigation is required to elucidate the specific regulatory mechanisms underlying Sqle expression in mast cells and its functional implications for SCI.

After SCI, the cellular microenvironment at the injury site undergoes dynamic and complex changes, which are both cell-specific and spatiotemporally regulated. To further investigate these cellular changes, we analyzed single-cell sequencing data from public databases. Among the cell types affected, microglia and T cells emerged as two key players, exhibiting distinct and potentially irreplaceable roles in SCI pathology. The expression patterns of IDI1, Elovl6, Stbd1, and Sqle in microglia and T cells were variable. ELOVL6 expression in both cell types appeared unaffected by injury, while IDI1, Sqle, and Stbd1 demonstrated significant changes in microglial expression post-injury. These findings suggest that RNA modifications in microglia are intricately linked to fatty acid metabolic reprogramming and glycophagy. Ferroptosis, a form of programmed cell death regulated by lipid metabolism, plays a significant role in microglial function. The redox activity of 15-lipoxygenase during the generation of pro-ferroptotic signals, such as 15-hydroperoxy-eicosa-tetra-enoyl-phosphatidylethanolamine, modulates ferroptotic resilience in microglia (Kapralov et al., 2020). These metabolic shifts influence microglial M1/M2 polarization, thereby regulating inflammation. On the basis of our findings, we speculate that post-SCI metabolic alterations in microglia promote a shift toward the pro-inflammatory M1 phenotype, exacerbating neuroinflammation. Interestingly, among the four biomarkers analyzed, IDI1 was the only gene whose expression notably changed in T cells following injury. Abnormal cellular metabolism, particularly fatty acid metabolism, is a primary driver of T cell dysfunction (Shi et al., 2020; Yu et al., 2021). Fatty acid metabolism is critical for T cell survival and function, influencing processes such as senescence, ferroptosis, and their antitumor response (Ping et al., 2022). CD4^+^ T cells exert neuroprotective effects (Gao et al., 2024), and Tregs play essential roles in regulating inflammation (Chen et al., 2023). These findings underscore the importance of focusing on RNA modifications and metabolic reprogramming in microglia and T cells after SCI. Investigating these processes offers a promising avenue for understanding the mechanisms underlying neuroinflammation and for developing therapeutic strategies to promote neurological recovery.

Despite these positive findings, the present study also had several limitations. First, the use of isoflurane for anesthesia may have interfered with the cellular transcriptome. Future research could address this by comparing different anesthesia methods, establishing anesthesia control groups, or employing bioinformatics approaches to correct the data, thereby excluding the influence of anesthesia. Additionally, we did not validate our findings in human samples. Future work should incorporate human SCI datasets (such as those from GEO or TCGA) and clinical samples (such as blood or cerebrospinal fluid) to validate the diagnostic and therapeutic potential of biomarkers like Elovl6, Idi1, Sqle, and Stbd1. Lastly, we did not verify the expression and function of key molecular targets at the protein level. Future research should employ functional experiments, such as immunofluorescence staining, gene knockout, or overexpression studies, to further elucidate the specific mechanisms of these molecules in SCI. These improvements will enhance the generalizability and clinical translational value of our study’s conclusions.

In conclusion, we performed bioinformatics analysis to identify RNA modification–related biomarkers (Elovl6, Idi1, Sqle, and Stbd1) associated with SCI. Additionally, we identified two key cellular players in SCI, T cells and microglia, and examined the expression patterns of the biomarkers in immune-infiltrating mast cells, T cells, and microglia. Functional enrichment analysis revealed pathways related to Elovl6, Idi1, Sqle, and Stbd1, highlighting their potential roles in SCI pathophysiology. These findings suggest that these biomarkers hold promise for therapeutic applications in SCI.

## Additional files:

***Additional Table 1:*** *RNA modification-related genes (RRGs) sourced from the published literature.*

Additional Table 1 RNA modification-related genes sourced from the published literature

***Additional Table 2:*** *Procedure of quantitative reverse transcription-polymerase chain reaction.*

***Additional Table 3:*** *Information on primer sequences.*

***Additional Table 4:*** *Gene Ontology (GO) enrichment analysis of differentially expressed genes (DEGs).*

Additional Table 4 Gene Ontology (GO) enrichment analysis of differentially expressed genes (DEGs)

***Additional Table 5:*** *Kyoto Encyclopedia of Genes and Genomes (KEGG) enrichment analysis of DEGs.*

Additional Table 5 Kyoto Encyclopedia of Genes and Genomes (KEGG) enrichment analysis of DEGs

***Additional Table 6:*** *GO enrichment analysis of the candidate genes.*

Additional Table 6 GO enrichment analysis of the candidate genes

***Additional Table 7:*** *KEGG enrichment analysis of the candidate genes.*

***Additional Table 8:*** *Correlation analysis between biomarkers and differential immune cells.*

***Additional Table 9:*** *The RNA concentration of samples.*

***Additional Figure 1:*** *Indicators for the successful establishment of an SCI model.*

Additional Figure 1 Indicators for the successful establishment of an SCI model.
(A-B) Macroscopic appearance of the spinal cord before (A) and after SCI (B). (C) After SCI, the mice exhibited hind limb paralysis and were unable to move voluntarily. (D) BMS scores of the Sham and SCI groups at different times. (E) Immunofluorescence results revealed that the injured area was characterized by neuronal loss (red) and an accumulation of astrocytes (green). BMS: Basso Mouse Scale; GFAP: glial fibrillary acidic protein; NeuN: neuronal nuclei; SCI: spinal cord injury.

***Additional Figure 2:*** *Quality control-related results of single-cell analysis.*

Additional Figure 2 Quality control-related results of single-cell analysis.
(A) Violin plots of single-cell quality control metrics before filtering. Distribution of three quality control metrics. (B) Violin plots of single-cell quality control metrics after filtering. (C) 2000 hypervariable genes after standard data processing. (D) Top 30 PCs selected for single-cell analysis. (E) P values associated with each gene across the top 30 PCs. (F) UMAP of 25 different cell clusters. (G) Heatmap showing the expression of marker genes for each cell type. DC: Dendritic cell; NK: natural killer cell; PCs: principal components.

***Additional Figure 3:*** *UMAP and expression of biomarkers of microglia and T-cell subsets.*

Additional Figure 3 UMAP and expression of biomarkers of microglia and T-cell subsets.
(A) UMAP plot of microglial subsets. Six distinct cell clusters (color coded 0–5) within microglia were visualized in a 2D UMAP space. The x-axis represents umap_1, and the y-axis represents umap_2, indicating the distribution and clustering of the microglial subpopulations. (B) UMAP plot of T-cell subsets. 7 distinct cell clusters (color - coded 0–6) within T cells projected in a 2D UMAP space. where umap_1 is on the x-axis and umap_2 is on the y-axis, illustrating the separation of T-cell subpopulations. (C) Pseudotemporal expression of biomarkers in microglial subsets. Plots of the relative expression of biomarkers (Elovl6, Idi1, Sqle, Stbd1) over pseudotime for microglial subpopulations. Each dot represents a cell, color-coded by subcell type, showing how biomarker expression changes along the pseudotemporal trajectory (likelihood ratio test). (D) Pseudotemporal expression of biomarkers in T-cell subsets. Displays the relative expression of biomarkers (Elovl6, Idi1, Sqle, Stbd1) over pseudotime for T-cell subpopulations (CD4+ T cells, CD8+ T cells, naive T cells, Th17 cells, and Treg cells). Dots represent individual cells, color-coded by subcell type, depicting biomarker expression dynamics during T-cell pseudotemporal progression (likelihood ratio test). UMAP: Uniform Manifold Approximation and Projection.

***Additional Figure 4:*** *Validation of the expression analysis of key genes in spinal cord injury-related cells over time.*

Additional Figure 4 Validation of the expression analysis of key genes in spinal cord injury-related cells over time.
(A) The expression of prognostic genes in microglia over pseudotime. (B) The expression of prognostic genes in T cells over pseudotime. The horizontal axis represents pseudotime, and the vertical axis represents prognostic genes. The color of the heatmap indicates the gene expression level, where a redder color corresponds to a higher gene expression level.

***Additional Figure 5:*** *Results of the analysis of gene expression and cell subtypes related to microglia.*

Additional Figure 5 Results of the analysis of gene expression and cell subtypes related to microglia.
(A) Heatmap of marker gene expression for microglial cell subtypes. The horizontal axis represents marker genes, and the vertical axis represents different cell types. The size of the circles reflects the content of genes in the cells; the larger the circle is, the greater the content. The shade of color represents the gene expression level; the darker the color is, the higher the expression level. (B) UMAP plot of the cell annotations. Different colors correspond to different cell types. (C) Pseudotime trajectory plot of microglia. The labels indicate pseudotime, and the dark-colored part is the starting position of development. (D) Gene kinetics during the cell differentiation process. The labels represent different developmental stages. (E) Trajectory plots of different cell subgroups. The labels correspond to different cell subgroups. (F-I) Distribution plots of key genes in different microglial cell subtypes, showing the distribution of the expression levels of four key genes, Elovl6, Stbd1, Idi1, and Sqle, in the M1_MGs and M2_MGs cell subtypes.

## Data Availability

*All relevant data are within the paper and its Additional files*.
