# Supplementary material for: Integrative bulk and single-cell transcriptome analyses reveal RNA modification–related biomarkers of spinal cord injury
Source: Neural Regen Res. 2025 Nov 25;21(7):3249–66. doi: 10.4103/NRR.NRR-D-25-00080 (PMC13379046; doi:10.4103/NRR.NRR-D-25-00080)
Supplement: Supplementary file 1 [file NRR-21-3249_Suppl1.pdf]

Additional Table 1 RNA modification-related genes sourced from the published literature

Symbol	RNA modifications type
DIS3L2	Uridylation[1]
MTPAP	Uridylation
TUT1	Uridylation
FTSJ1	RNA ribose methylation[2]
FTSJ3	RNA ribose methylation
MRM1	RNA ribose methylation
TARBP1	RNA ribose methylation
BCDIN3D	RNA cap methylations[3]
MEPCE	RNA cap methylations
RNGTT	RNA cap methylations
DKC1	Pseudouracil[4]
GAR1	Pseudouracil
NHP2	Pseudouracil
NOP10	Pseudouracil
PUS1	Pseudouracil
PUS10	Pseudouracil
PUS3	Pseudouracil
PUS7	Pseudouracil
RPUSD1	Pseudouracil
RPUSD2	Pseudouracil
RPUSD3	Pseudouracil
RPUSD4	Pseudouracil
ALKBH8	mcm5s2U[5]
CTU1	mcm5s2U
CTU2	mcm5s2U
ELP2	mcm5s2U
ELP3	mcm5s2U
ELP4	mcm5s2U
URM1	mcm5s2U
CYFIP1	m7G[6]
DCP2	m7G
DCPS	m7G
EIF3D	m7G
EIF4A1	m7G
EIF4E	m7G
EIF4E1B	m7G
EIF4E2	m7G

EIF4E3	m7G
EIF4G3	m7G
GEMIN5	m7G
LARP1	m7G
LSM1	m7G
METTL1	m7G
NCBP1	m7G
NCBP2	m7G
NUDT11	m7G
NUDT16	m7G
NUDT3	m7G
NUDT4	m7G
SNUPN	m7G
WDR4	m7G
PCIF1	m6Am[7]
ALKBH5	m6A[8]
HNRNPA2B1	m6A
IGF2BP1	m6A
IGF2BP2	m6A
IGF2BP3	m6A
LRPPRC	m6A
METTL14	m6A
METTL16	m6A
METTL3	m6A
NPLOC4	m6A
RBM15	m6A
RBM15B	m6A
RBMX	m6A
RBMY1A1	m6A
SNRPC	m6A
WTAP	m6A
YTHDC2	m6A
ZC3H13	m6A
NSUN2	m5C[9]/m7G
TET2	m5C/hm5C
DNMT1	m5C
DNMT3A	m5C
DNMT3B	m5C
NOP2	m5C
NSUN3	m5C

NSUN4	m5C
NSUN5	m5C
NSUN6	m5C
NSUN7	m5C
TRDMT1	m5C
YBX1	m5C
METTL2A	m3C[10]
METTL6	m3C
YTHDC1	m1A[11]/m6A
YTHDF1	m1A/m6A
YTHDF2	m1A/m6A
YTHDF3	m1A/m6A
ALKBH1	m1A
ALKBH3	m1A
RRP8	m1A
TRMT6	m1A
TRMT61A	m1A
TRMT61B	m1A
TET1	hm5C[12]
TET3	hm5C
ADAD1	A-to-I RNA editing[13]
ADAD2	A-to-I RNA editing
ADAT2	A-to-I RNA editing
CPSF6	APA[14]
CPSF7	APA
CSTF2	APA
CSTF2T	APA
FUS	APA
NOVA2	APA
NUDT21	APA
PABPN1	APA
PCF11	APA
RBBP6	APA
SF3B1	APA
SRSF3	APA
SRSF7	APA
U2AF2	APA

[1]Uridylation: Uridylate residues were added to the 3' end of RNA, which affected the stability, processing, and degradation of RNA and participated in the metabolic regulation of various RNAs such as miRNA and mRNA.

[2]RNA ribose methylation: It occurred in the ribose part of RNA, was commonly found in rRNA and tRNA, affected the structure and

function of RNA, and was crucial for the assembly of ribosomes, the stability of tRNA, and the recognition of amino acids.

[3]RNA cap methylation: In addition to the m7G modification in the 5' cap of mRNA, there might have been other modifications such as 2'-O-methylation, which synergistically regulated the metabolic functions of mRNA.

[4]Pseudouridine (Ψ): It was an isomeric form of uracil, formed by the connection between the C5 position of uracil and the C1' position of ribose. It was widely present in RNA, affected the structure and function of RNA, and participated in the regulation of translation and RNA-protein interactions.

[5]mcm5s2U: It was a commonly modified nucleotide in tRNA, located near the anticodon loop of tRNA, and was important for the structure of tRNA and the recognition of codons.

[6]m7G: It was commonly found in the 5' cap of mRNA, was crucial for the stability, transportation, and initiation of translation of mRNA. It could protect mRNA from degradation by nucleases and provided binding sites for translation initiation factors.

[7]m6Am: It was the modification of the first adenosine downstream of the 5' cap of mRNA, possessing both N6 methylation and 2'-O-methylation, and might have affected the stability and translation efficiency of mRNA.

[8]m6A: It was the most common internal modification in eukaryotic mRNA, catalyzed by a methyltransferase complex, could be recognized by "reader" proteins, participated in the regulation of multiple processes of mRNA, and played a key role in cellular physiology and diseases.

[9]m5C: It was the methylation modification of the 5th carbon atom of cytidine, existed in tRNA, rRNA, mRNA, etc., affected the structure and function of RNA, and participated in the regulation of gene expression.

[10]m3C: It was the methylation modification of the 3rd nitrogen atom of cytidine, and might have affected the structure and function of RNA.

[11]m1A: It was the methylation modification of the 1st nitrogen atom of adenosine, affected the structure, stability of RNA, and its interaction with proteins, and participated in the initiation of translation and RNA metabolism.

[12]hm5C: It was the oxidized modification form of m5C, and might have played an important role in the regulation of RNA.

[13]A-to-I RNA editing: Adenosine (A) in specific double-stranded RNA regions was deaminated to form inosine (I). Since inosine was similar to guanosine (G) in base pairing, it could change the information, structure, and function of RNA, and was important for gene expression and protein diversity.

[14]APA: Different polyadenylation sites were used during gene transcription, generating mRNA transcripts with different lengths of the 3'-UTR, which affected the stability, localization, and translation efficiency of mRNA and regulated gene expression.
