# Supplementary figures and images for: Integrative bulk and single-cell transcriptome analyses reveal RNA modification–related biomarkers of spinal cord injury
Source: Neural Regen Res. 2025 Nov 25;21(7):3249–66. doi: 10.4103/NRR.NRR-D-25-00080 (PMC13379046; doi:10.4103/NRR.NRR-D-25-00080)

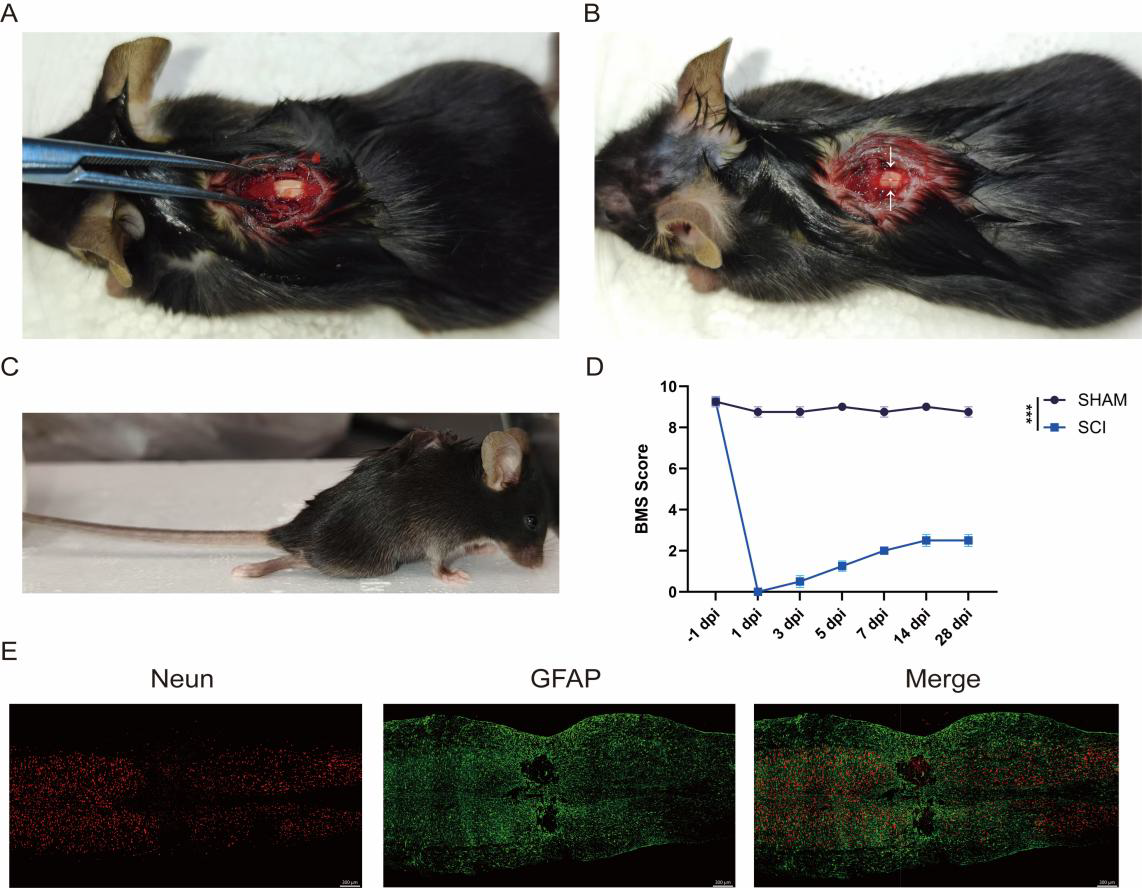

Supplement: Supplementary file 2 [file NRR-21-3249_Suppl1.tif]

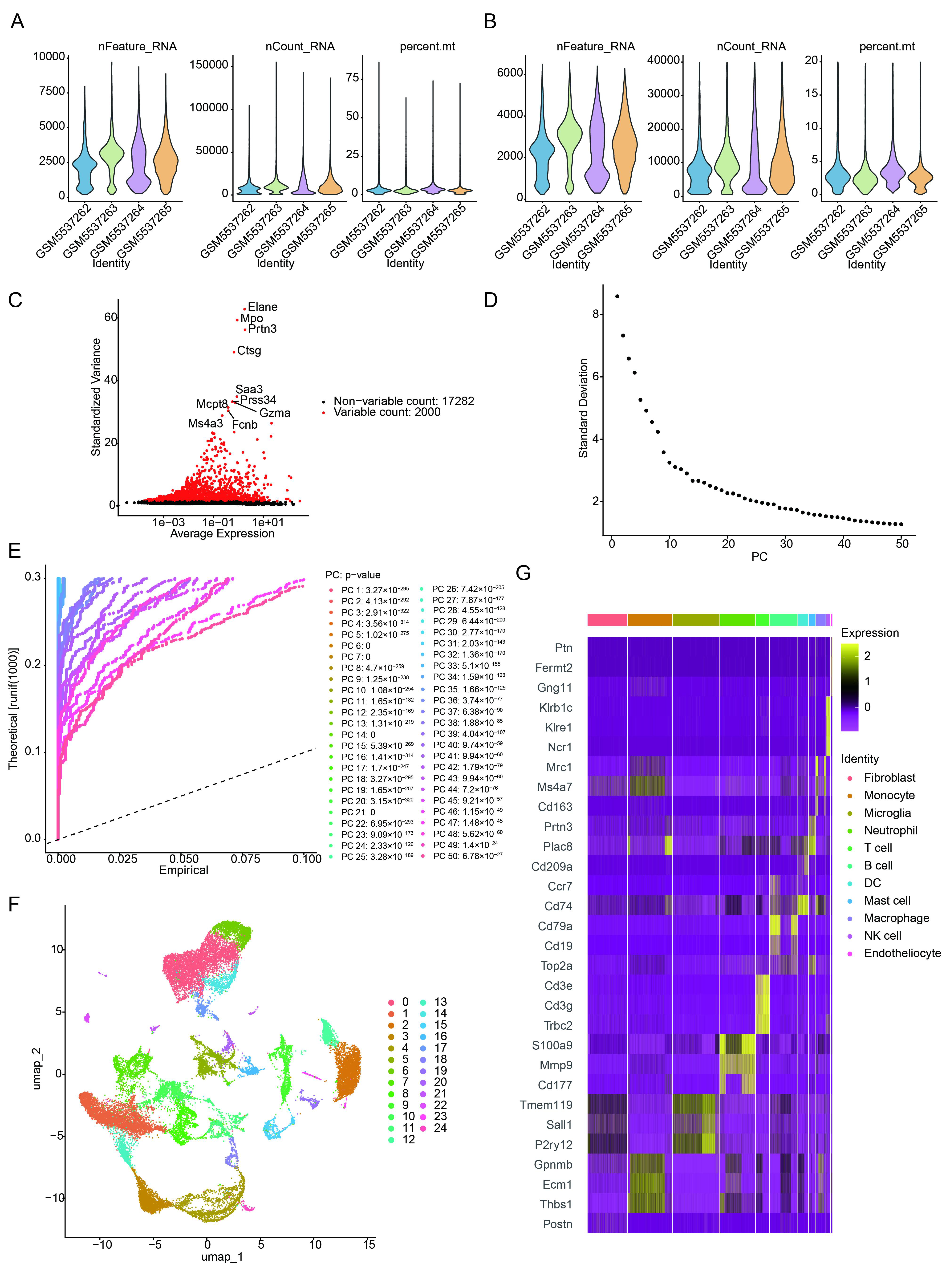

Supplement: Supplementary file 6 [file NRR-21-3249_Suppl2.tif]

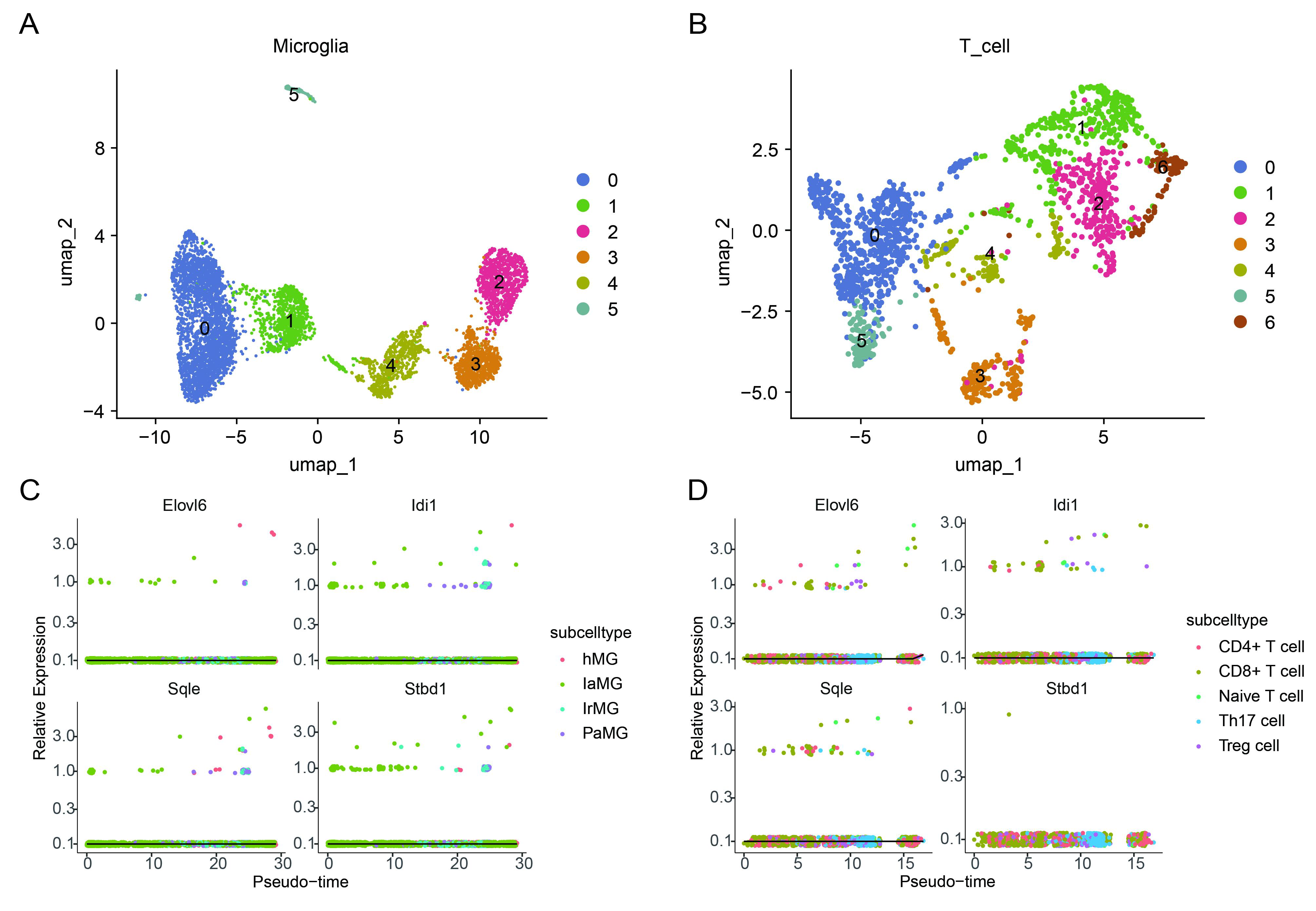

Supplement: Supplementary file 7 [file NRR-21-3249_Suppl3.tif]

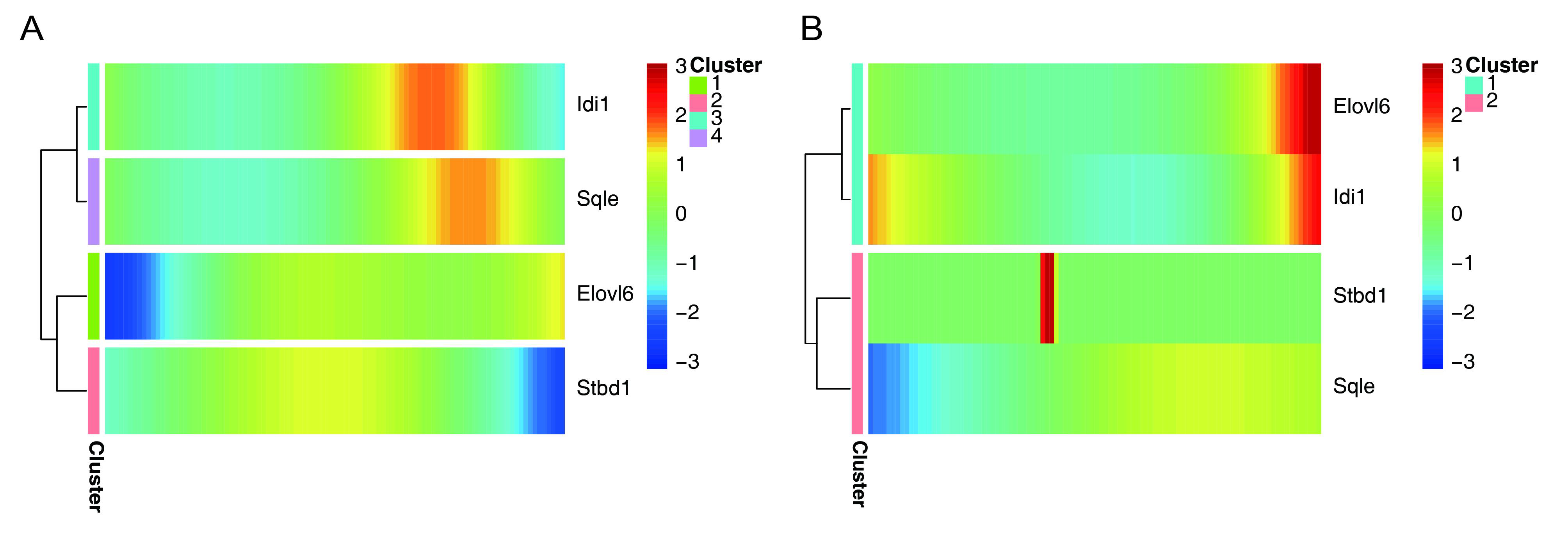

Supplement: Supplementary file 8 [file NRR-21-3249_Suppl4.tif]

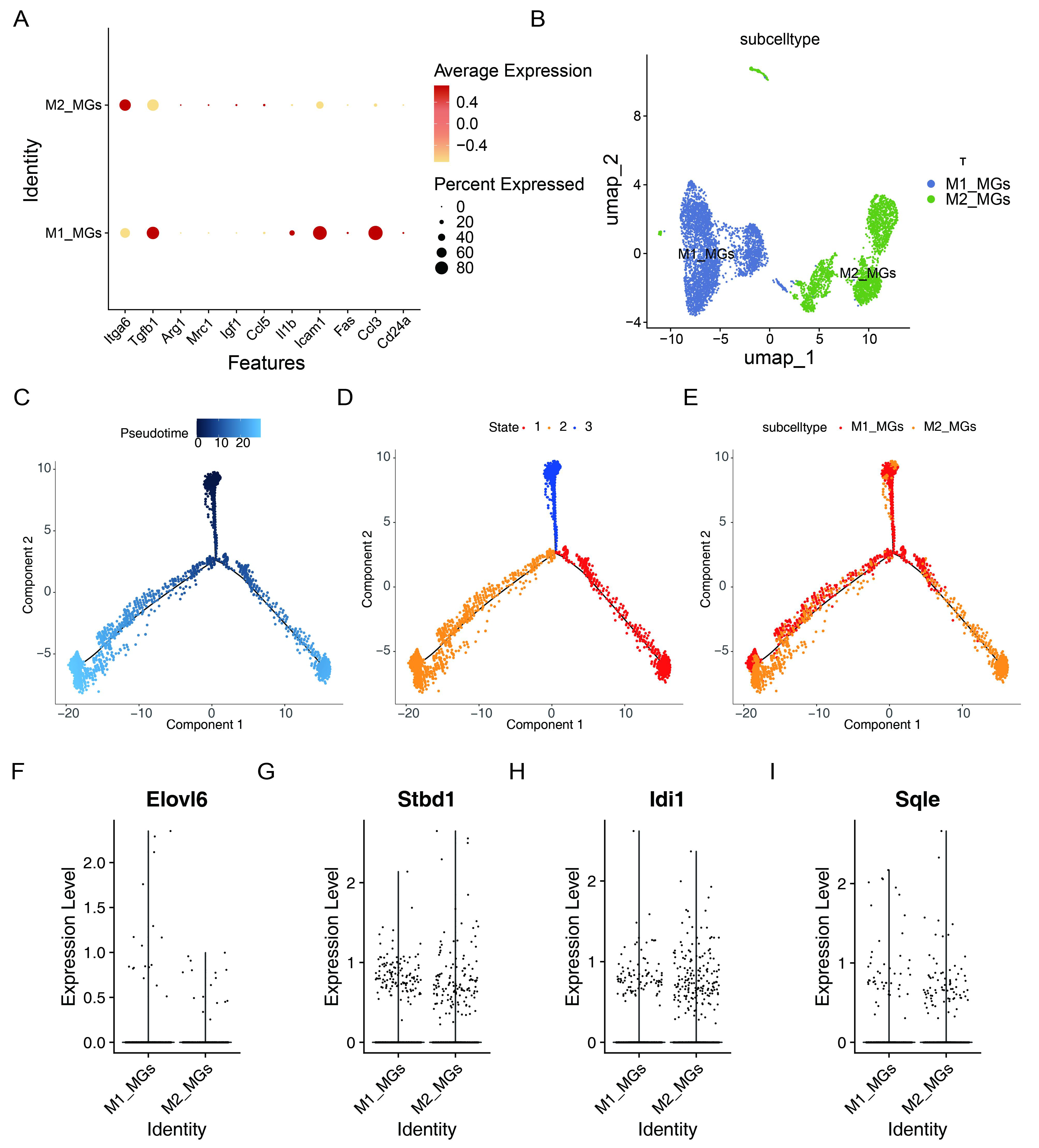

Supplement: Supplementary file 9 [file NRR-21-3249_Suppl5.tif]
