# Supplementary material for: Integrative bulk and single-cell transcriptome analyses reveal RNA modification–related biomarkers of spinal cord injury
Source: Neural Regen Res. 2025 Nov 25;21(7):3249–66. doi: 10.4103/NRR.NRR-D-25-00080 (PMC13379046; doi:10.4103/NRR.NRR-D-25-00080)
Supplement: Supplementary file 3 [file NRR-21-3249_Suppl2.pdf]

Additional Table 4 Gene Ontology (GO) enrichment analysis of differentially expressed genes (DEGs)

	ONTOLOGY	ID	Description	GeneRatio	BgRatio	P-value	Padj	q value	geneID	Count	richFactor
GO:0070997	BP	GO:0070997	neuron death	19/180	450/28943	6.57838288652996e-11	2.14257930614281e-07	1.50056375948531e-07	Jun/Adam8/Ccl2/C1qa/Npy/Ctsz/Tyrobp/Tnfrsf1b/Adarb1/Nes/Nrp1/Mt1/Bdnf/Ddit4/Mapt/Apoe/Ccr5/Agtr2/Efnb2	19	0.422222222222222
GO:1901214	BP	GO:1901214	regulation of neuron death	16/180	399/28943	4.71847715126122e-09	5.97651511468367e-06	4.18567563095503e-06	Jun/Adam8/Ccl2/C1qa/Npy/Ctsz/Tyrobp/Tnfrsf1b/Nes/Nrp1/Mt1/Bdnf/Ddit4/Mapt/Apoe/Efnb2	16	0.401002506265664
GO:0010977	BP	GO:0010977	negative regulation of neuron projection development	11/180	160/28943	5.5049264181919e-09	5.97651511468367e-06	4.18567563095503e-06	Ctsz/B2m/Sema7a/Rit2/Nrp1/Apoe/Gfap/Lgals1/Lpar1/Ccr5/Efnb2	11	0.6875
GO:0050767	BP	GO:0050767	regulation of neurogenesis	16/180	463/28943	3.77244560569908e-08	2.40590043878209e-05	1.6849817400064e-05	Adcyap1/Sox11/Tnfrsf1b/S100a10/Enpp2/B2m/Sema7a/Sgk1/Nrp1/Bdnf/Mapt/Apoe/Anxa2/Gfap/Egr2/Ccr5	16	0.345572354211663
GO:0002683	BP	GO:0002683	negative regulation of immune system process	16/180	465/28943	4.00312575939912e-08	2.40590043878209e-05	1.6849817400064e-05	Adcyap1/Cst7/Mmp12/Ccl2/Hoxa7/Sox11/Npy/Sfrp1/C1qc/Cd68/Tyrobp/Lgals3/Tmem176a/Laptm5/Igf2/Tmem176b	16	0.344086021505376
GO:0032102	BP	GO:0032102	negative regulation of response to external stimulus	15/180	407/28943	4.43211625197806e-08	2.40590043878209e-05	1.6849817400064e-05	Adcyap1/Cst7/Mmp12/Ccl2/Npy/Tnfrsf1b/Nr1d2/Ctla2a/Sema7a/Igf2/Nrp1/Nucb2/Apoe/Anxa2/Htra1	15	0.368550368550369
GO:0031345	BP	GO:0031345	negative regulation of cell projection organization	11/180	213/28943	1.05402328009844e-07	4.90421974754375e-05	3.43468939544861e-05	Ctsz/B2m/Sema7a/Rit2/Nrp1/Apoe/Gfap/Lgals1/Lpar1/Ccr5/Efnb2	11	0.516431924882629
GO:0010720	BP	GO:0010720	positive regulation of cell development	14/180	384/28943	1.44711571715006e-07	5.89156986344719e-05	4.12618389350551e-05	Sox11/Cyp51/Tyrobp/Tnfrsf1b/S100a10/Enpp2/Sema7a/Sgk1/Nrp1/Bdnf/Mapt/Apoe/Gfap/Egr2	14	0.364583333333333
GO:1901652	BP	GO:1901652	response to peptide	14/180	430/28943	5.65206756516711e-07	0.0001783627824311	0.000124917069155728	Mmp9/Sgk1/Car2/Serpina3n/Igf2/Bglap/Serpina1b/Nucb2/Rbp4/Inhbb/Pdk4/Serpina1a/Egr2/Serpina1c	14	0.325581395348837
GO:0050727	BP	GO:0050727	regulation of inflammatory response	13/180	367/28943	5.66699306716535e-07	0.0001783627824311	0.000124917069155728	Adcyap1/Cst7/Ctss/Adam8/Npy/Tnfrsf1b/Nr1d2/Ctla2a/Sema7a/Apoe/Lgals1/Ccr5/Pla2g4a	13	0.354223433242507
GO:0043434	BP	GO:0043434	response to peptide hormone	13/180	369/28943	6.0239195785757e-07	0.0001783627824311	0.000124917069155728	Sgk1/Car2/Serpina3n/Igf2/Bglap/Serpina1b/Nucb2/Rbp4/Inhbb/Pdk4/Serpina1a/Egr2/Serpina1c	13	0.35230352303523
GO:0010631	BP	GO:0010631	epithelial cell migration	12/180	328/28943	1.11542623882008e-06	0.000274847970824579	0.000192490846525496	Jun/Gadd45a/Adam8/Mmp9/Enpp2/Itgb2/Igf2/Nrp1/Apoe/Cd63/Nus1/Efnb2	12	0.365853658536585

GO:0051402	BP	GO:0051402	neuron apoptotic process	12/180	329/28943	1.15145385051339e-06	0.000274847970824579	0.000192490846525496	Jun/Adam8/Ccl2/Ctsz/Tyrobp/Adarb1/Nes/Nrp1/Mt1/Bdnf/Apoe/Agtr2	12	0.364741641337386
GO:0090132	BP	GO:0090132	epithelium migration	12/180	330/28943	1.1885088561878e-06	0.000274847970824579	0.000192490846525496	Jun/Gadd45a/Adam8/Mmp9/Enpp2/Itgb2/Igf2/Nrp1/Apoe/Cd63/Nus1/Efnb2	12	0.363636363636364
GO:0090130	BP	GO:0090130	tissue migration	12/180	332/28943	1.26580275172511e-06	0.000274847970824579	0.000192490846525496	Jun/Gadd45a/Adam8/Mmp9/Enpp2/Itgb2/Igf2/Nrp1/Apoe/Cd63/Nus1/Efnb2	12	0.36144578313253
GO:0001667	BP	GO:0001667	ameboidal-type cell migration	14/180	471/28943	1.65314520069172e-06	0.000336518369915808	0.000235681950651247	Jun/Gadd45a/Mmp12/Adam8/Mmp9/Enpp2/Itgb2/Sema7a/Igf2/Nrp1/Apoe/Cd63/Nus1/Efnb2	14	0.29723991507431
GO:0032488	BP	GO:0032488	Cdc42 protein signal transduction	4/180	15/28943	1.87192260880725e-06	0.000350309098886347	0.000245340341365848	Abca1/Rit2/Nrp1/Apoe	4	2.66666666666667
GO:1901216	BP	GO:1901216	positive regulation of neuron death	8/180	133/28943	1.93600361668482e-06	0.000350309098886347	0.000245340341365848	Jun/C1qa/Ctsz/Tyrobp/Ddit4/Mapt/Apoe/Efnb2	8	0.601503759398496
GO:0008299	BP	GO:0008299	isoprenoid biosynthetic process	5/180	35/28943	2.4555108520551e-06	0.000412002840831261	0.000288547793766088	Idi1/Fdft1/Fdps/Aldh1a1/Nus1	5	1.42857142857143
GO:0051047	BP	GO:0051047	positive regulation of secretion	13/180	420/28943	2.5299529679537e-06	0.000412002840831261	0.000288547793766088	Adcyap1/Sox11/Lgals3/S100a10/Itgb2/Vsnl1/Oxct1/Nucb2/Anxa2/Rbp4/Inhbb/Agtr2/Pla2g4a	13	0.30952380952381
GO:0016126	BP	GO:0016126	sterol biosynthetic process	6/180	63/28943	2.6950311349722e-06	0.000417986495552592	0.000292738469648358	Cyp51/Fdft1/Sqle/Fdps/Msmo1/Apoe	6	0.952380952380952
GO:0060537	BP	GO:0060537	muscle tissue development	14/180	494/28943	2.87368831862991e-06	0.000425436493353529	0.000297956104615838	Atf3/Dsp/Sox11/Fgf9/Fdps/Nr1d2/Lmna/Col14a1/Igf2/Bdnf/Rbp4/Egr2/Agtr2/Efnb2	14	0.283400809716599
GO:0031348	BP	GO:0031348	negative regulation of defense response	10/180	244/28943	3.28974280499573e-06	0.000451153607104676	0.000315967185364586	Adcyap1/Cst7/Mmp12/Npy/Tnfrsf1b/Nr1d2/Ctla2a/Igf2/Apoe/Htra1	10	0.40983606557377
GO:1903532	BP	GO:1903532	positive regulation of secretion by cell	12/180	365/28943	3.36743298036349e-06	0.000451153607104676	0.000315967185364586	Adcyap1/Sox11/Lgals3/S100a10/Itgb2/Vsnl1/Oxct1/Nucb2/Anxa2/Rbp4/Inhbb/Pla2g4a	12	0.328767123287671

GO:0051962	BP	GO:0051962	positive regulation of nervous system development	12/180	366/28943	3.46295369285137e-06	0.000451153607104676	0.000315967185364586	Sox11/Tnfrsf1b/Enpp2/Sema7a/Sgk1/Nrp1/Bdnf/Mapt/Apoe/Cbln1/Gfap/Egr2	12	0.327868852459016
GO:0050769	BP	GO:0050769	positive regulation of neurogenesis	11/180	305/28943	3.62068319804415e-06	0.000453560199078069	0.000317652651423549	Sox11/Tnfrsf1b/Enpp2/Sema7a/Sgk1/Nrp1/Bdnf/Mapt/Apoe/Gfap/Egr2	11	0.360655737704918
GO:0031589	BP	GO:0031589	cell-substrate adhesion	12/180	371/28943	3.97730041185069e-06	0.000479780275607322	0.000336015984112298	Mmp12/Adam8/Hoxa7/Npy/Sfrp1/Itgax/S100a10/Enpp2/Itgb2/Nrp1/Lgals1/Cd63	12	0.32345013477089
GO:0051000	BP	GO:0051000	positive regulation of nitric-oxide synthase activity	4/180	18/28943	4.13557224306222e-06	0.000481055671273344	0.000336909212432926	Apoe/Npr3/Nus1/Agtr2	4	2.22222222222222
GO:0050678	BP	GO:0050678	regulation of epithelial cell proliferation	13/180	442/28943	4.40664435210543e-06	0.000494911746717496	0.000346613368820779	Jun/Mmp12/Ccl2/Sox11/Sfrp1/Scg2/Fgf9/B2m/Igf2/Apoe/Htra1/Ctsl/Efnb2	13	0.294117647058824
GO:0032103	BP	GO:0032103	positive regulation of response to external stimulus	13/180	459/28943	6.61306865482888e-06	0.000717958820292589	0.000502825255263656	Ctss/Mmp12/Adam8/Ccl2/Npy/Scg2/Tyrobp/Ankrd17/Nrp1/Lgals1/Lpar1/Ccr5/Pla2g4a	13	0.28322440087146
GO:1901215	BP	GO:1901215	negative regulation of neuron death	10/180	267/28943	7.28106182447366e-06	0.000764981237493894	0.00053575758823886	Jun/Adam8/Ccl2/Npy/Tnfrsf1b/Nes/Nrp1/Mt1/Bdnf/Apoe	10	0.374531835205993
GO:0048638	BP	GO:0048638	regulation of developmental growth	12/180	402/28943	8.94698405417648e-06	0.000900748705471694	0.000630842863066484	Fgf9/Fdps/H19/Sema7a/Col14a1/Igf2/Nrp1/Bdnf/Mapt/Apoe/Rbp4/Ccr5	12	0.298507462686567
GO:0006898	BP	GO:0006898	receptor-mediated endocytosis	10/180	274/28943	9.12640690223085e-06	0.000900748705471694	0.000630842863066484	Adm/Msr1/B2m/Itgb2/Sfrp4/Apoe/Anxa2/Ctsl/Cd63/Efnb2	10	0.364963503649635
GO:0051098	BP	GO:0051098	regulation of binding	12/180	408/28943	1.03748173577724e-05	0.000956611576157259	0.000669966642060915	Jun/Sox11/Ctsz/Mmp9/S100a10/B2m/Nes/Ckmt1/Bdnf/Mark2/Apoe/Anxa2	12	0.294117647058824
GO:0043393	BP	GO:0043393	regulation of protein binding	9/180	221/28943	1.0821247040174e-05	0.000956611576157259	0.000669966642060915	Ctsz/Mmp9/B2m/Nes/Ckmt1/Bdnf/Mark2/Apoe/Anxa2	9	0.407239819004525
GO:0006694	BP	GO:0006694	steroid biosynthetic process	8/180	169/28943	1.14021996722162e-05	0.000956611576157259	0.000669966642060915	Cyp51/Fdft1/Slc27a2/Sqle/Fdps/Igf2/Msmo1/Apoe	8	0.473372781065089

GO:0031643	BP	GO:0031643	positive regulation of myelination	4/180	23/28943	1.16799746399244e-05	0.000956611576157259	0.000669966642060915	Cst7/Itgax/Tnfrsf1b/Egr2	4	1.73913043478261
GO:0072677	BP	GO:0072677	eosinophil migration	4/180	23/28943	1.16799746399244e-05	0.000956611576157259	0.000669966642060915	Adam8/Ccl2/Scg2/Lgals3	4	1.73913043478261
GO:1900120	BP	GO:1900120	regulation of receptor binding	4/180	23/28943	1.16799746399244e-05	0.000956611576157259	0.000669966642060915	Mmp9/B2m/Bdnf/Anxa2	4	1.73913043478261
GO:0043534	BP	GO:0043534	blood vessel endothelial cell migration	7/180	122/28943	1.1748376741262e-05	0.000956611576157259	0.000669966642060915	Gadd45a/Adam8/Igf2/Nrp1/Apoe/Nus1/Efnb2	7	0.573770491803279
GO:0051961	BP	GO:0051961	negative regulation of nervous system development	8/180	172/28943	1.29586269723937e-05	0.00102942068412405	0.000720958784317768	Adcyap1/Sox11/B2m/Sema7a/Nrp1/Bdnf/Cbln1/Ccr5	8	0.465116279069767
GO:0009991	BP	GO:0009991	response to extracellular stimulus	12/180	420/28943	1.38404286434149e-05	0.00107329228789529	0.000751684432839098	Atf3/Jun/Npy/Bckdhb/Mmp9/Cd68/Bglap/Nucb2/Mapt/Apoe/Inhbb/Pdk4	12	0.285714285714286
GO:0008202	BP	GO:0008202	steroid metabolic process	11/180	354/28943	1.47151157787032e-05	0.00109778443749428	0.000768837605174317	Abca1/Adm/Cyp51/Fdft1/Slc27a2/Sqle/Fdps/Ugt1a1/Igf2/Msmo1/Apoe	11	0.310734463276836
GO:0043523	BP	GO:0043523	regulation of neuron apoptotic process	10/180	290/28943	1.49202410623055e-05	0.00109778443749428	0.000768837605174317	Jun/Adam8/Ccl2/Ctsz/Tyrobp/Nes/Nrp1/Mt1/Bdnf/Apoe	10	0.344827586206897
GO:0050679	BP	GO:0050679	positive regulation of epithelial cell proliferation	9/180	232/28943	1.59315955825418e-05	0.00109778443749428	0.000768837605174317	Jun/Mmp12/Ccl2/Sox11/Sfrp1/Scg2/Fgf9/Igf2/Htra1	9	0.387931034482759
GO:1903531	BP	GO:1903531	negative regulation of secretion by cell	8/180	177/28943	1.59495424021133e-05	0.00109778443749428	0.000768837605174317	Sfrp1/Cyp51/Tnfrsf1b/Vsnl1/Nucb2/Apoe/Inhbb/Agtr2	8	0.451977401129944
GO:0150063	BP	GO:0150063	visual system development	12/180	427/28943	1.62979254716592e-05	0.00109778443749428	0.000768837605174317	Jun/C1qa/Sox11/Atf6/Fgf9/Nrp1/Bdnf/Pbx3/Mab21l2/Rbp4/Inhbb/Aldh1a1	12	0.281030444964871
GO:0007160	BP	GO:0007160	cell-matrix adhesion	9/180	233/28943	1.64841225534788e-05	0.00109778443749428	0.000768837605174317	Mmp12/Adam8/Hoxa7/Itgax/S100a10/Enpp2/Itgb2/Nrp1/Cd63	9	0.386266094420601

GO:0006066	BP	GO:0006066	alcohol metabolic process	11/180	359/28943	1.67538438787254e-05	0.00109778443749428	0.000768837605174317	Abca1/Cyp51/Fdft1/Sqle/Fdps/Msmo1/Apoe/Rbp4/Aldh1a1/Nus1/Pla2g4a	11	0.306406685236769
GO:1901617	BP	GO:1901617	organic hydroxy compound biosynthetic process	9/180	234/28943	1.70528870045941e-05	0.00109778443749428	0.000768837605174317	Cyp51/Fdft1/Slc27a2/Sqle/Fdps/Msmo1/Apoe/Nus1/Agtr2	9	0.384615384615385
GO:0040013	BP	GO:0040013	negative regulation of locomotion	11/180	360/28943	1.71897471023053e-05	0.00109778443749428	0.000768837605174317	Gadd45a/Ccl2/Hoxa7/Sfrp1/Acan/Adarb1/Sema7a/Nrp1/Apoe/Cd63/Ccr5	11	0.305555555555556
GO:0010038	BP	GO:0010038	response to metal ion	10/180	296/28943	1.77921770566314e-05	0.00109796338519645	0.000768962931894289	Jun/Mt2/Mmp9/B2m/Mt1/Serpina1b/Mapt/Ank3/Pla2g4a/Serpina1c	10	0.337837837837838
GO:0048880	BP	GO:0048880	sensory system development	12/180	431/28943	1.78667667839767e-05	0.00109796338519645	0.000768962931894289	Jun/C1qa/Sox11/Atf6/Fgf9/Nrp1/Bdnf/Pbx3/Mab21l2/Rbp4/Inhbb/Aldh1a1	12	0.278422273781903
GO:0071276	BP	GO:0071276	cellular response to cadmium ion	4/180	26/28943	1.94340171455829e-05	0.00117215914524377	0.000820926221334856	Jun/Mt2/Mmp9/Mt1	4	1.53846153846154
GO:0035023	BP	GO:0035023	regulation of Rho protein signal transduction	6/180	91/28943	2.28966732585885e-05	0.0013558993600586	0.000949609396198303	Abca1/Arhgef3/Rit2/Nrp1/Apoe/Lpar1	6	0.659340659340659
GO:1905952	BP	GO:1905952	regulation of lipid localization	8/180	187/28943	2.36935470751412e-05	0.00135779026732764	0.000950933700466688	Abca1/Msr1/Nucb2/Apoe/Anxa2/Nus1/Agtr2/Pla2g4a	8	0.427807486631016
GO:0072593	BP	GO:0072593	reactive oxygen species metabolic process	9/180	245/28943	2.44970809263169e-05	0.00135779026732764	0.000950933700466688	Gadd45a/Tyrobp/Abcb7/Itgb2/Gpx3/Ddit4/Mapt/Prdx6/Pdk4	9	0.36734693877551
GO:0010951	BP	GO:0010951	negative regulation of endopeptidase activity	8/180	188/28943	2.46168078219945e-05	0.00135779026732764	0.000950933700466688	Cst7/Mmp9/Cst3/Serpina3n/Serpinb6a/Serpina1b/Serpina1a/Serpina1c	8	0.425531914893617
GO:0045765	BP	GO:0045765	regulation of angiogenesis	10/180	308/28943	2.49857885435727e-05	0.00135779026732764	0.000950933700466688	Gadd45a/Ccl2/Adm/Mmp9/Lgals3/Itgax/Enpp2/Itgb2/Igf2/Nrp1	10	0.324675324675325
GO:0046879	BP	GO:0046879	hormone secretion	11/180	375/28943	2.50130230395022e-05	0.00135779026732764	0.000950933700466688	Adcyap1/Sox11/Adm/Sfrp1/Vsnl1/Oxct1/Nucb2/Rbp4/Inhbb/Pclo/Agtr2	11	0.293333333333333

GO:0019932	BP	GO:0019932	second-messenger-mediated signaling	10/180	309/28943	2.56843122996612e-05	0.00137137385508191	0.000960447019039964	Adcyap1/Mt2/Lat2/Rit2/Mt1/Nucb2/Mapt/Apoe/Lpar1/Ccr5	10	0.323624595469256
GO:0050863	BP	GO:0050863	regulation of T cell activation	11/180	378/28943	2.69019207623275e-05	0.00139332213731474	0.000975818583960391	Adam8/Ccl2/Igfbp2/Lgals3/Tnfrsf1b/B2m/Ctla2a/Laptm5/Igf2/Lgals1/Efnb2	11	0.291005291005291
GO:0010632	BP	GO:0010632	regulation of epithelial cell migration	9/180	248/28943	2.69509655053204e-05	0.00139332213731474	0.000975818583960391	Jun/Gadd45a/Mmp9/Enpp2/Igf2/Nrp1/Apoe/Cd63/Nus1	9	0.362903225806452
GO:1901342	BP	GO:1901342	regulation of vasculature development	10/180	312/28943	2.78813034264689e-05	0.00139926201434941	0.000979978599969354	Gadd45a/Ccl2/Adm/Mmp9/Lgals3/Itgax/Enpp2/Itgb2/Igf2/Nrp1	10	0.32051282051282
GO:0006695	BP	GO:0006695	cholesterol biosynthetic process	5/180	57/28943	2.83547107605346e-05	0.00139926201434941	0.000979978599969354	Cyp51/Fdft1/Fdps/Msmo1/Apoe	5	0.87719298245614
GO:1902653	BP	GO:1902653	secondary alcohol biosynthetic process	5/180	57/28943	2.83547107605346e-05	0.00139926201434941	0.000979978599969354	Cyp51/Fdft1/Fdps/Msmo1/Apoe	5	0.87719298245614
GO:0009914	BP	GO:0009914	hormone transport	11/180	383/28943	3.03245878670179e-05	0.00145741632560449	0.00102070719828868	Adcyap1/Sox11/Adm/Sfrp1/Vsnl1/Oxct1/Nucb2/Rbp4/Inhbb/Pclo/Agtr2	11	0.287206266318538
GO:0032770	BP	GO:0032770	positive regulation of monooxygenase activity	4/180	29/28943	3.04280964510608e-05	0.00145741632560449	0.00102070719828868	Apoe/Npr3/Nus1/Agtr2	4	1.37931034482759
GO:0043524	BP	GO:0043524	negative regulation of neuron apoptotic process	8/180	195/28943	3.1962998895017e-05	0.00150874619421841	0.00105665627163237	Jun/Adam8/Ccl2/Nes/Nrp1/Mt1/Bdnf/Apoe	8	0.41025641025641
GO:0002761	BP	GO:0002761	regulation of myeloid leukocyte differentiation	7/180	143/28943	3.28608405676304e-05	0.00152883772698216	0.00107072745483262	Jun/Adam8/Hoxa7/Sfrp1/C1qc/Tyrobp/Car2	7	0.48951048951049
GO:0031667	BP	GO:0031667	response to nutrient levels	11/180	387/28943	3.33274420066728e-05	0.00152883772698216	0.00107072745483262	Atf3/Jun/Npy/Bckdhb/Cd68/Bglap/Nucb2/Mapt/Apoe/Inhbb/Pdk4	11	0.284237726098191
GO:0015833	BP	GO:0015833	peptide transport	10/180	320/28943	3.45437510891512e-05	0.00155633260431182	0.00108998359922554	Adcyap1/Abca1/Sfrp1/Car2/Vsnl1/Oxct1/Nucb2/Rbp4/Inhbb/Pclo	10	0.3125

GO:0006720	BP	GO:0006720	isoprenoid metabolic process	6/180	98/28943	3.4882493127038e-05	0.00155633260431182	0.00108998359922554	Idi1/Fdft1/Fdps/Rbp4/Aldh1a1/Nus1	6	0.612244897959184
GO:0008203	BP	GO:0008203	cholesterol metabolic process	7/180	145/28943	3.59162053254673e-05	0.00158079838844658	0.00110711830640523	Abca1/Cyp51/Fdft1/Sqle/Fdps/Msmo1/Apoe	7	0.482758620689655
GO:1904950	BP	GO:1904950	negative regulation of establishment of protein localization	7/180	146/28943	3.75291902779878e-05	0.00162244422379845	0.00113628512934772	Sfrp1/Cyp51/Vsnl1/Mapt/Apoe/Inhbb/Mdfic	7	0.479452054794521
GO:0022409	BP	GO:0022409	positive regulation of cell-cell adhesion	10/180	324/28943	3.83568330465092e-05	0.00162244422379845	0.00113628512934772	Adam8/Ccl2/Igfbp2/B2m/Itgb2/Igf2/Ank3/Lgals1/Ccr5/Efnb2	10	0.308641975308642
GO:0045927	BP	GO:0045927	positive regulation of growth	10/180	324/28943	3.83568330465092e-05	0.00162244422379845	0.00113628512934772	Sfrp1/Fgf9/Fdps/Sema7a/Sgk1/Igf2/Nrp1/Bdnf/Mapt/Apoe	10	0.308641975308642
GO:0050728	BP	GO:0050728	negative regulation of inflammatory response	7/180	147/28943	3.92011977457839e-05	0.00163690129561562	0.00114641019588548	Adcyap1/Cst7/Npy/Tnfrsf1b/Nr1d2/Ctla2a/Apoe	7	0.476190476190476
GO:0042391	BP	GO:0042391	regulation of membrane potential	12/180	469/28943	4.06838626340954e-05	0.00167730810885125	0.00117470926486455	Dsp/Adcyap1/Jun/B2m/Gna14/Clic1/Bdnf/Mapt/Cbln1/Ank3/Ppa2/Pclo	12	0.255863539445629
GO:0031102	BP	GO:0031102	neuron projection regeneration	5/180	62/28943	4.27349331994744e-05	0.0017398459678836	0.00121850789793764	Jun/Adm/Gap43/Nrep/Gfap	5	0.806451612903226
GO:0051048	BP	GO:0051048	negative regulation of secretion	8/180	206/28943	4.71649396739332e-05	0.0018964964014568	0.00132821863903071	Sfrp1/Cyp51/Tnfrsf1b/Vsnl1/Nucb2/Apoe/Inhbb/Agtr2	8	0.388349514563107
GO:0007229	BP	GO:0007229	integrin-mediated signaling pathway	6/180	104/28943	4.87610297272641e-05	0.00190253093734409	0.00133244494973567	Tyrobp/Itgax/Itgb2/Sema7a/Nrp1/Cd63	6	0.576923076923077
GO:0009306	BP	GO:0009306	protein secretion	11/180	404/28943	4.91330352156778e-05	0.00190253093734409	0.00133244494973567	Adcyap1/Abca1/Sfrp1/Cyp51/Vsnl1/Oxct1/Apoe/Cbln1/Rbp4/Inhbb/Pclo	11	0.272277227722772
GO:0021561	BP	GO:0021561	facial nerve development	3/180	12/28943	4.99381305720108e-05	0.00190253093734409	0.00133244494973567	Adarb1/Nrp1/Egr2	3	2.5

GO:0021610	BP	GO:0021610	facial nerve morphogenesis	3/180	12/28943	4.99381305720108e-05	0.00190253093734409	0.00133244494973567	Adarb1/Nrp1/Egr2	3	2.5
GO:0035592	BP	GO:0035592	establishment of protein localization to extracellular region	11/180	405/28943	5.02356956130155e-05	0.00190253093734409	0.00133244494973567	Adcyap1/Abca1/Sfrp1/Cyp51/Vsnl1/Oxct1/Apoe/Cbln1/Rbp4/Inhbb/Pclo	11	0.271604938271605
GO:0050999	BP	GO:0050999	regulation of nitric-oxide synthase activity	4/180	33/28943	5.14157151589901e-05	0.00192483889968771	0.00134806841802216	Apoe/Npr3/Nus1/Agtr2	4	1.21212121212121
GO:0016125	BP	GO:0016125	sterol metabolic process	7/180	154/28943	5.27008007064549e-05	0.00195052849887413	0.00136606022883837	Abca1/Cyp51/Fdft1/Sqle/Fdps/Msmo1/Apoe	7	0.454545454545455
GO:0031099	BP	GO:0031099	regeneration	7/180	155/28943	5.49071939309479e-05	0.00200705489457457	0.0014056487101605	Jun/Adm/Kpna1/Gap43/Nrep/Bdnf/Gfap	7	0.451612903225806
GO:0030307	BP	GO:0030307	positive regulation of cell growth	8/180	211/28943	5.5842890055188e-05	0.00200705489457457	0.0014056487101605	Sfrp1/Fdps/Sema7a/Sgk1/Nrp1/Bdnf/Mapt/Apoe	8	0.37914691943128
GO:0048588	BP	GO:0048588	developmental cell growth	9/180	273/28943	5.680681953761e-05	0.00200705489457457	0.0014056487101605	Fdps/Sema7a/Col14a1/Nrp1/Bdnf/Mapt/Apoe/Ccr5/Agtr2	9	0.32967032967033
GO:0007159	BP	GO:0007159	leukocyte cell-cell adhesion	11/180	411/28943	5.73092125254637e-05	0.00200705489457457	0.0014056487101605	Adam8/Ccl2/Igfbp2/Lgals3/Msn/B2m/Itgb2/Laptm5/Igf2/Lgals1/Efnb2	11	0.267639902676399
GO:0071692	BP	GO:0071692	protein localization to extracellular region	11/180	411/28943	5.73092125254637e-05	0.00200705489457457	0.0014056487101605	Adcyap1/Abca1/Sfrp1/Cyp51/Vsnl1/Oxct1/Apoe/Cbln1/Rbp4/Inhbb/Pclo	11	0.267639902676399
GO:0032368	BP	GO:0032368	regulation of lipid transport	7/180	157/28943	5.95470889862898e-05	0.00206324328540793	0.00144500046845789	Abca1/Nucb2/Apoe/Anxa2/Nus1/Agtr2/Pla2g4a	7	0.445859872611465
GO:1902652	BP	GO:1902652	secondary alcohol metabolic process	7/180	158/28943	6.1984511871247e-05	0.00212509005436475	0.00148831509390573	Abca1/Cyp51/Fdft1/Sqle/Fdps/Msmo1/Apoe	7	0.443037974683544
GO:0030516	BP	GO:0030516	regulation of axon extension	6/180	109/28943	6.3428080931249e-05	0.0021519297874279	0.00150711240546071	Sema7a/Nrp1/Bdnf/Mapt/Apoe/Ccr5	6	0.55045871559633

GO:0010288	BP	GO:0010288	response to lead ion	3/180	13/28943	6.46225318972019e-05	0.00216985140607409	0.00151966388085986	Serpina1b/Mapt/Serpina1c	3	2.30769230769231
GO:0007409	BP	GO:0007409	axonogenesis	12/180	493/28943	6.55683811089356e-05	0.00217914507420208	0.00152617273751948	Gap43/Adarb1/Sema7a/Nrp1/Bdnf/Mapt/Mark2/Apoe/Ank3/Egr2/Ccr5/Efnb2	12	0.243407707910751
GO:0048639	BP	GO:0048639	positive regulation of developmental growth	8/180	218/28943	7.01924988309307e-05	0.00230028731718621	0.00161101517907746	Fgf9/Fdps/Sema7a/Igf2/Nrp1/Bdnf/Mapt/Apoe	8	0.36697247706422
GO:0022407	BP	GO:0022407	regulation of cell-cell adhesion	12/180	497/28943	7.07911043815616e-05	0.00230028731718621	0.00161101517907746	Adam8/Ccl2/Igfbp2/Lgals3/B2m/Itgb2/Laptm5/Igf2/Ank3/Lgals1/Ccr5/Efnb2	12	0.241448692152917
GO:1902105	BP	GO:1902105	regulation of leukocyte differentiation	10/180	349/28943	7.1332213397546e-05	0.00230028731718621	0.00161101517907746	Jun/Adam8/Hoxa7/Sfrp1/C1qc/Tyrobp/Tmem176a/Ctla2a/Car2/Tmem176b	10	0.286532951289398
GO:0097242	BP	GO:0097242	amyloid-beta clearance	4/180	36/28943	7.29444066340901e-05	0.00232921502360031	0.00163127481089859	Cyp51/Msr1/Itgb2/Apoe	4	1.11111111111111
GO:0002526	BP	GO:0002526	acute inflammatory response	6/180	112/28943	7.37926794791589e-05	0.00233342482586039	0.00163422316230289	Adcyap1/Adam8/Npy/Serpina3n/Serpina1b/Ccr5	6	0.535714285714286
GO:0001654	BP	GO:0001654	eye development	11/180	424/28943	7.56222923982708e-05	0.00236828659943431	0.0016586387411645	Jun/Sox11/Atf6/Fgf9/Nrp1/Bdnf/Pbx3/Mab21l2/Rbp4/Inhbb/Aldh1a1	11	0.259433962264151
GO:0150076	BP	GO:0150076	neuroinflammatory response	5/180	70/28943	7.67855831541436e-05	0.00237769431426818	0.00166522747087864	Jun/Cst7/C1qa/Tyrobp/Tnfrsf1b	5	0.714285714285714
GO:0042886	BP	GO:0042886	amide transport	10/180	353/28943	7.8376549074448e-05	0.00237769431426818	0.00166522747087864	Adcyap1/Abca1/Sfrp1/Car2/Vsnl1/Oxct1/Nucb2/Rbp4/Inhbb/Pclo	10	0.28328611898017
GO:2001236	BP	GO:2001236	regulation of extrinsic apoptotic signaling pathway	7/180	164/28943	7.8382521339943e-05	0.00237769431426818	0.00166522747087864	Atf3/Sfrp1/Scg2/Lgals3/Lmna/Nrp1/Agtr2	7	0.426829268292683
GO:0001503	BP	GO:0001503	ossification	11/180	426/28943	7.88427958062521e-05	0.00237769431426818	0.00166522747087864	Cebpd/Sox11/Sfrp1/Mmp9/Fgf9/Clec3b/Lmna/Clic1/Igf2/Bglap/Egr2	11	0.258215962441315

GO:2000644	BP	GO:2000644	regulation of receptor catabolic process	3/180	14/28943	8.18706470558666e-05	0.00244635502257759	0.00171331426528308	Laptm5/Apoe/Anxa2	3	2.14285714285714
GO:0001649	BP	GO:0001649	osteoblast differentiation	8/180	225/28943	8.74814325301236e-05	0.00255259471893198	0.00178771965028379	Cebpd/Sox11/Sfrp1/Fgf9/Lmna/Clic1/Igf2/Bglap	8	0.355555555555556
GO:0051607	BP	GO:0051607	defense response to virus	9/180	289/28943	8.78007447751806e-05	0.00255259471893198	0.00178771965028379	Mmp12/Ankrd17/Ifit1/Itgax/Il10rb/Ddit4/Htra1/Ifih1/Trim25	9	0.311418685121107
GO:0070613	BP	GO:0070613	regulation of protein processing	5/180	72/28943	8.78816546079529e-05	0.00255259471893198	0.00178771965028379	Cst7/Ctsz/S100a10/Clec3b/Ctla2a	5	0.694444444444444
GO:0140546	BP	GO:0140546	defense response to symbiont	9/180	290/28943	9.01349957837779e-05	0.00255259471893198	0.00178771965028379	Mmp12/Ankrd17/Ifit1/Itgax/Il10rb/Ddit4/Htra1/Ifih1/Trim25	9	0.310344827586207
GO:0045637	BP	GO:0045637	regulation of myeloid cell differentiation	8/180	226/28943	9.0217024577171e-05	0.00255259471893198	0.00178771965028379	Jun/Adam8/Hoxa7/Sfrp1/C1qc/Tyrobp/B2m/Car2	8	0.353982300884956
GO:0046686	BP	GO:0046686	response to cadmium ion	4/180	38/28943	9.05258857890723e-05	0.00255259471893198	0.00178771965028379	Jun/Mt2/Mmp9/Mt1	4	1.05263157894737
GO:0050768	BP	GO:0050768	negative regulation of neurogenesis	7/180	168/28943	9.11616583433834e-05	0.00255259471893198	0.00178771965028379	Adcyap1/Sox11/B2m/Sema7a/Nrp1/Bdnf/Ccr5	7	0.416666666666667
GO:0014706	BP	GO:0014706	striated muscle tissue development	9/180	291/28943	9.25211407613307e-05	0.00255259471893198	0.00178771965028379	Dsp/Fgf9/Fdps/Lmna/Col14a1/Bdnf/Rbp4/Agtr2/Efnb2	9	0.309278350515464
GO:0045861	BP	GO:0045861	negative regulation of proteolysis	10/180	361/28943	9.42510806130743e-05	0.00255259471893198	0.00178771965028379	Cst7/Ctsz/Mmp9/Cst3/Ctla2a/Serpina3n/Serpinb6a/Serpina1b/Serpina1a/Serpina1c	10	0.277008310249307
GO:0052548	BP	GO:0052548	regulation of endopeptidase activity	10/180	361/28943	9.42510806130743e-05	0.00255259471893198	0.00178771965028379	Cst7/Mmp9/Cst3/Ctsd/Laptm5/Serpina3n/Serpinb6a/Serpina1b/Serpina1a/Serpina1c	10	0.277008310249307
GO:0046887	BP	GO:0046887	positive regulation of hormone secretion	7/180	169/28943	9.46080547057178e-05	0.00255259471893198	0.00178771965028379	Adcyap1/Sox11/Vsnl1/Oxct1/Nucb2/Rbp4/Inhbb	7	0.414201183431953

GO:1903706	BP	GO:1903706	regulation of hemopoiesis	11/180	435/28943	9.48308139363737e-05	0.00255259471893198	0.00178771965028379	Jun/Adam8/Hoxa7/Sfrp1/C1qc/Tyrobp/Tmem176a/B2m/Ctla2a/Car2/Tmem176b	11	0.252873563218391
GO:0015850	BP	GO:0015850	organic hydroxy compound transport	9/180	294/28943	0.000100000151199408	0.00266967616767599	0.00186971809878444	Abca1/Msr1/Lgals3/Nucb2/Apoe/Anxa2/Rbp4/Nus1/Agtr2	9	0.306122448979592
GO:0007188	BP	GO:0007188	adenylate cyclase-modulating G protein-coupled receptor signaling pathway	8/180	231/28943	0.000104981485749348	0.00277987560232215	0.00194689670191559	Adcyap1/Abca1/Adm/Adcy9/Rit2/Gna14/Npr3/Lpar1	8	0.346320346320346
GO:0031346	BP	GO:0031346	positive regulation of cell projection organization	11/180	441/28943	0.000106964990954527	0.00280955625434591	0.00196768366212614	Adcyap1/Enpp2/Sema7a/Sgk1/Rit2/Nrp1/Bdnf/Mapt/Mark2/Apoe/Fam98a	11	0.249433106575964
GO:0050830	BP	GO:0050830	defense response to Gram-positive bacterium	7/180	174/28943	0.000113469816819292	0.00293623121470255	0.00205640096383835	Adm/Npy/Mpeg1/B2m/Lyz1/Gbp3/Lyz2	7	0.402298850574713
GO:0002790	BP	GO:0002790	peptide secretion	9/180	299/28943	0.000113590768514744	0.00293623121470255	0.00205640096383835	Adcyap1/Abca1/Sfrp1/Vsnl1/Oxct1/Nucb2/Rbp4/Inhbb/Pclo	9	0.301003344481605
GO:0043542	BP	GO:0043542	endothelial cell migration	8/180	234/28943	0.000114761972670042	0.00294314759831754	0.002061244880033	Gadd45a/Adam8/Itgb2/Igf2/Nrp1/Apoe/Nus1/Efnb2	8	0.341880341880342
GO:1903037	BP	GO:1903037	regulation of leukocyte cell-cell adhesion	10/180	371/28943	0.000117856156977322	0.00299888674433701	0.00210028200797579	Adam8/Ccl2/Igfbp2/Lgals3/B2m/Itgb2/Laptm5/Igf2/Lgals1/Efnb2	10	0.269541778975741
GO:1903317	BP	GO:1903317	regulation of protein maturation	5/180	77/28943	0.000121029838109019	0.00304884378570811	0.00213526961641468	Cst7/Ctsz/S100a10/Clec3b/Ctla2a	5	0.649350649350649
GO:0030100	BP	GO:0030100	regulation of endocytosis	8/180	236/28943	0.000121691646343891	0.00304884378570811	0.00213526961641468	Lgals3/B2m/Sfrp4/Rit2/Apoe/Anxa2/Cd63/Efnb2	8	0.338983050847458
GO:0051044	BP	GO:0051044	positive regulation of membrane protein ectodomain proteolysis	3/180	16/28943	0.000124805686177612	0.00308892893340206	0.00216334340611239	Adam8/Tnfrsf1b/Apoe	3	1.875
GO:0042692	BP	GO:0042692	muscle cell differentiation	11/180	449/28943	0.000125188400150666	0.00308892893340206	0.00216334340611239	Adm/Ankrd17/Fgf9/Fdps/Lmna/Col14a1/Igf2/Bdnf/Cd53/Agtr2/Efnb2	11	0.244988864142539

GO:0097191	BP	GO:0097191	extrinsic apoptotic signaling pathway	8/180	238/28943	0.000128963506578998	0.0031250311782542	0.00218862775343046	Atf3/Sfrp1/Scg2/Lgals3/Tnfrsf1b/Lmna/Nrp1/Agtr2	8	0.336134453781513
GO:0061387	BP	GO:0061387	regulation of extent of cell growth	6/180	124/28943	0.000129529999712716	0.0031250311782542	0.00218862775343046	Sema7a/Nrp1/Bdnf/Mapt/Apoe/Ccr5	6	0.483870967741935
GO:1905954	BP	GO:1905954	positive regulation of lipid localization	6/180	124/28943	0.000129529999712716	0.0031250311782542	0.00218862775343046	Abca1/Msr1/Nucb2/Apoe/Anxa2/Pla2g4a	6	0.483870967741935
GO:0001774	BP	GO:0001774	microglial cell activation	4/180	42/28943	0.000134634380770097	0.0032242954277074	0.00225814785703406	Jun/Cst7/C1qa/Tyrobp	4	0.952380952380952
GO:0046883	BP	GO:0046883	regulation of hormone secretion	9/180	308/28943	0.000141957880681564	0.00337486728014492	0.00236360144016096	Adcyap1/Sox11/Sfrp1/Vsnl1/Oxct1/Nucb2/Rbp4/Inhbb/Agtr2	9	0.292207792207792
GO:0090087	BP	GO:0090087	regulation of peptide transport	8/180	242/28943	0.000144586003960792	0.00339536051956153	0.00237795396017964	Adcyap1/Sfrp1/Car2/Vsnl1/Oxct1/Nucb2/Rbp4/Inhbb	8	0.330578512396694
GO:0050770	BP	GO:0050770	regulation of axonogenesis	7/180	181/28943	0.000144904854841588	0.00339536051956153	0.00237795396017964	Sema7a/Nrp1/Bdnf/Mapt/Mark2/Apoe/Ccr5	7	0.386740331491713
GO:0045766	BP	GO:0045766	positive regulation of angiogenesis	7/180	182/28943	0.000149921017759022	0.00346306918327046	0.00242537398644121	Adm/Mmp9/Lgals3/Itgax/Itgb2/Igf2/Nrp1	7	0.384615384615385
GO:1904018	BP	GO:1904018	positive regulation of vasculature development	7/180	182/28943	0.000149921017759022	0.00346306918327046	0.00242537398644121	Adm/Mmp9/Lgals3/Itgax/Itgb2/Igf2/Nrp1	7	0.384615384615385
GO:0070661	BP	GO:0070661	leukocyte proliferation	10/180	384/28943	0.000155861157419325	0.00356090898318746	0.00249389647126585	Sox11/Igfbp2/Tyrobp/Lgals3/Msn/Tnfrsf1b/Itgb2/Laptm5/Igf2/Npr3	10	0.260416666666667
GO:0030336	BP	GO:0030336	negative regulation of cell migration	9/180	312/28943	0.000156343255939762	0.00356090898318746	0.00249389647126585	Gadd45a/Ccl2/Hoxa7/Sfrp1/Acan/Adarb1/Apoe/Cd63/Ccr5	9	0.288461538461538
GO:0050709	BP	GO:0050709	negative regulation of protein secretion	5/180	82/28943	0.000163008708122609	0.00368333262664005	0.00257963634663122	Sfrp1/Cyp51/Vsnl1/Apoe/Inhbb	5	0.609756097560976

GO:0060326	BP	GO:0060326	cell chemotaxis	9/180	314/28943	0.000163980113866383	0.00368333262664005	0.00257963634663122	Adam8/Ccl2/Scg2/Lgals3/Enpp2/Itgb2/Nrp1/Lpar1/Ccr5	9	0.286624203821656
GO:0002697	BP	GO:0002697	regulation of immune effector process	11/180	464/28943	0.000166547933271538	0.00371538779907807	0.00260208631145942	Ccl2/Tyrobp/Lgals3/Ankrd17/Tnfrsf1b/B2m/Itgb2/Sema7a/Laptm5/Igf2/Rbp4	11	0.237068965517241
GO:0050829	BP	GO:0050829	defense response to Gram-negative bacterium	6/180	131/28943	0.000174908902186194	0.00387536254707778	0.00271412524910478	Adm/Npy/Mpeg1/B2m/Lyz1/Lyz2	6	0.458015267175573
GO:0090287	BP	GO:0090287	regulation of cellular response to growth factor stimulus	9/180	318/28943	0.000180189830004137	0.00391991261775568	0.0027453260645659	Sox11/Sfrp1/Fgf9/Nrep/Sfrp4/Nrp1/Htra1/Cd63/Agtr2	9	0.283018867924528
GO:0010755	BP	GO:0010755	regulation of plasminogen activation	3/180	18/28943	0.000180201450643104	0.00391991261775568	0.0027453260645659	Ctsz/S100a10/Clec3b	3	1.66666666666667
GO:0032868	BP	GO:0032868	response to insulin	8/180	250/28943	0.000180530209598819	0.00391991261775568	0.0027453260645659	Sgk1/Igf2/Bglap/Nucb2/Rbp4/Inhbb/Pdk4/Egr2	8	0.32
GO:0010876	BP	GO:0010876	lipid localization	11/180	470/28943	0.000186073596285693	0.00401352121259935	0.00281088520844264	Abca1/Slc27a2/Msr1/Sqle/Nucb2/Apoe/Anxa2/Rbp4/Nus1/Agtr2/Pla2g4a	11	0.234042553191489
GO:0002269	BP	GO:0002269	leukocyte activation involved in inflammatory response	4/180	46/28943	0.000192520630543096	0.00409829865149584	0.00287025941786645	Jun/Cst7/C1qa/Tyrobp	4	0.869565217391304
GO:0032768	BP	GO:0032768	regulation of monooxygenase activity	4/180	46/28943	0.000192520630543096	0.00409829865149584	0.00287025941786645	Apoe/Npr3/Nus1/Agtr2	4	0.869565217391304
GO:0030198	BP	GO:0030198	extracellular matrix organization	9/180	322/28943	0.000197713542583284	0.00418151304021919	0.00292853893901556	Ctss/Mmp12/Acan/Mmp9/Lgals3/Tnfrsf1b/Col14a1/Anxa2/Gfap	9	0.279503105590062
GO:0043062	BP	GO:0043062	extracellular structure organization	9/180	323/28943	0.000202309337782257	0.00425110653649555	0.00297727901510459	Ctss/Mmp12/Acan/Mmp9/Lgals3/Tnfrsf1b/Col14a1/Anxa2/Gfap	9	0.278637770897833
GO:0014013	BP	GO:0014013	regulation of gliogenesis	6/180	135/28943	0.000205978940150627	0.00429413034911969	0.00300741090979506	Adcyap1/Sox11/Tnfrsf1b/Enpp2/Gfap/Egr2	6	0.444444444444444

GO:0045229	BP	GO:0045229	external encapsulating structure organization	9/180	324/28943	0.000206993695060421	0.00429413034911969	0.00300741090979506	Ctss/Mmp12/Acan/Mmp9/Lgals3/Tnfrsf1b/Col14a1/Anxa2/Gfap	9	0.277777777777778
GO:2000146	BP	GO:2000146	negative regulation of cell motility	9/180	325/28943	0.000211767945796648	0.00433612127492298	0.00303681941817885	Gadd45a/Ccl2/Hoxa7/Sfrp1/Acan/Adarb1/Apoe/Cd63/Ccr5	9	0.276923076923077
GO:0021783	BP	GO:0021783	preganglionic parasympathetic fiber development	3/180	19/28943	0.000213011791215129	0.00433612127492298	0.00303681941817885	Adarb1/Nrp1/Egr2	3	1.57894736842105
GO:0048245	BP	GO:0048245	eosinophil chemotaxis	3/180	19/28943	0.000213011791215129	0.00433612127492298	0.00303681941817885	Ccl2/Scg2/Lgals3	3	1.57894736842105
GO:2001233	BP	GO:2001233	regulation of apoptotic signaling pathway	10/180	401/28943	0.00022072338373978	0.00446085512156525	0.00312417725334321	Atf3/Sfrp1/Scg2/Mmp9/Lgals3/Lmna/Nrp1/Bdnf/Inhbb/Agtr2	10	0.249376558603491
GO:0031644	BP	GO:0031644	regulation of nervous system process	7/180	194/28943	0.00022187857835234	0.00446085512156525	0.00312417725334321	Cst7/Ctss/Itgax/Tnfrsf1b/Cbln1/Egr2/Pclo	7	0.360824742268041
GO:0048675	BP	GO:0048675	axon extension	6/180	138/28943	0.000232014729114854	0.00463602437255877	0.00324685772032217	Sema7a/Nrp1/Bdnf/Mapt/Apoe/Ccr5	6	0.434782608695652
GO:0007266	BP	GO:0007266	Rho protein signal transduction	6/180	139/28943	0.000241245965023193	0.00479108602488134	0.00335545575228023	Abca1/Arhgef3/Rit2/Nrp1/Apoe/Lpar1	6	0.431654676258993
GO:0061900	BP	GO:0061900	glial cell activation	4/180	49/28943	0.000246361932717539	0.00486303524158196	0.00340584566634071	Jun/Cst7/C1qa/Tyrobp	4	0.816326530612245
GO:0010466	BP	GO:0010466	negative regulation of peptidase activity	8/180	262/28943	0.000247973281146447	0.00486535528128903	0.00340747051518295	Cst7/Mmp9/Cst3/Serpina3n/Serpinb6a/Serpina1b/Serpina1a/Serpina1c	8	0.305343511450382
GO:0046165	BP	GO:0046165	alcohol biosynthetic process	6/180	141/28943	0.00026057453033806	0.00508198350485665	0.00355918693503041	Cyp51/Fdft1/Fdps/Msmo1/Apoe/Nus1	6	0.425531914893617
GO:0030574	BP	GO:0030574	collagen catabolic process	4/180	50/28943	0.000266492400673868	0.00516646279163565	0.00361835233245785	Ctss/Mmp12/Mmp9/Ctsl	4	0.8

GO:0051224	BP	GO:0051224	negative regulation of protein transport	6/180	142/28943	0.000270684496114594	0.00521668286298955	0.00365352415496933	Sfrp1/Cyp51/Vsnl1/Apoe/Inhbb/Mdfic	6	0.422535211267606
GO:0022604	BP	GO:0022604	regulation of cell morphogenesis	9/180	338/28943	0.000282644384212512	0.00541513387870677	0.0037925100965233	Sprr1b/Ccl2/Msn/S100a10/Enpp2/Sgk1/Nrp1/Mark2/Lpar1	9	0.266272189349112
GO:0050808	BP	GO:0050808	synapse organization	11/180	496/28943	0.000294712683457476	0.0056133287135731	0.00393131662082087	C1qa/C1qc/Gap43/Nrp1/Bdnf/Mapt/Apoe/Cbln1/Ank3/Pclo/Efnb2	11	0.221774193548387
GO:0048144	BP	GO:0048144	fibroblast proliferation	6/180	145/28943	0.00030287448499606	0.00573524533507075	0.00401670140138594	Jun/Sfrp1/Mmp9/Cks2/Anxa2/Agtr2	6	0.413793103448276
GO:0030003	BP	GO:0030003	cellular cation homeostasis	11/180	498/28943	0.000304922391940904	0.0057406487315117	0.00402048569112223	Ccl2/Mt2/Abcb7/Sgk1/Car2/Mt1/Nucb2/Apoe/Ccr5/Agtr2/Slc4a3	11	0.220883534136546
GO:0045773	BP	GO:0045773	positive regulation of axon extension	4/180	52/28943	0.000310254826959895	0.00578734303467819	0.0040531882281556	Sema7a/Nrp1/Mapt/Apoe	4	0.769230769230769
GO:0048738	BP	GO:0048738	cardiac muscle tissue development	8/180	271/28943	0.000311064863761837	0.00578734303467819	0.0040531882281556	Dsp/Fgf9/Fdps/Lmna/Col14a1/Rbp4/Agtr2/Efnb2	8	0.29520295202952
GO:0006869	BP	GO:0006869	lipid transport	10/180	419/28943	0.000312733304913528	0.00578734303467819	0.0040531882281556	Abca1/Slc27a2/Msr1/Nucb2/Apoe/Anxa2/Rbp4/Nus1/Agtr2/Pla2g4a	10	0.238663484486873
GO:0002274	BP	GO:0002274	myeloid leukocyte activation	8/180	272/28943	0.000318817571567316	0.00583364511570084	0.00408561607088335	Jun/Cst7/C1qa/Npy/Tyrobp/Itgb2/Lat2/Pla2g4a	8	0.294117647058824
GO:1903039	BP	GO:1903039	positive regulation of leukocyte cell-cell adhesion	8/180	272/28943	0.000318817571567316	0.00583364511570084	0.00408561607088335	Adam8/Ccl2/Igfbp2/B2m/Itgb2/Igf2/Lgals1/Efnb2	8	0.294117647058824
GO:0046651	BP	GO:0046651	lymphocyte proliferation	9/180	344/28943	0.000321430521552864	0.00584859893127194	0.00409608903384332	Sox11/Igfbp2/Tyrobp/Lgals3/Msn/Tnfrsf1b/Itgb2/Laptm5/Igf2	9	0.261627906976744
GO:0043535	BP	GO:0043535	regulation of blood vessel endothelial cell migration	5/180	95/28943	0.000324361817643897	0.00586914688925652	0.00411047987622412	Gadd45a/Igf2/Nrp1/Apoe/Nus1	5	0.526315789473684

GO:0048486	BP	GO:0048486	parasympathetic nervous system development	3/180	22/28943	0.000333917547803587	0.00597647280461313	0.00418564599893239	Adarb1/Nrp1/Egr2	3	1.36363636363636
GO:0050798	BP	GO:0050798	activated T cell proliferation	4/180	53/28943	0.000333963171765303	0.00597647280461313	0.00418564599893239	Igfbp2/Itgb2/Laptm5/Igf2	4	0.754716981132075
GO:0009615	BP	GO:0009615	response to virus	9/180	347/28943	0.000342414815786253	0.00609423527331053	0.00426812140240903	Mmp12/Ankrd17/Ifit1/Itgax/Il10rb/Ddit4/Htra1/Ifih1/Trim25	9	0.259365994236311
GO:0002673	BP	GO:0002673	regulation of acute inflammatory response	4/180	54/28943	0.000358940918304139	0.00631930038333288	0.00442574662853525	Adcyap1/Adam8/Npy/Ccr5	4	0.740740740740741
GO:0048483	BP	GO:0048483	autonomic nervous system development	4/180	54/28943	0.000358940918304139	0.00631930038333288	0.00442574662853525	Sox11/Adarb1/Nrp1/Egr2	4	0.740740740740741
GO:0010721	BP	GO:0010721	negative regulation of cell development	7/180	211/28943	0.000369058334345764	0.00646248922023738	0.00452602948798681	Adcyap1/Sox11/B2m/Sema7a/Nrp1/Bdnf/Ccr5	7	0.33175355450237
GO:0032943	BP	GO:0032943	mononuclear cell proliferation	9/180	351/28943	0.000372143298415894	0.00648166162000303	0.00453945695281307	Sox11/Igfbp2/Tyrobp/Lgals3/Msn/Tnfrsf1b/Itgb2/Laptm5/Igf2	9	0.256410256410256
GO:0050772	BP	GO:0050772	positive regulation of axonogenesis	5/180	98/28943	0.000374522481006698	0.0064884027693554	0.0045441781430096	Sema7a/Nrp1/Bdnf/Mapt/Apoe	5	0.510204081632653
GO:0051043	BP	GO:0051043	regulation of membrane protein ectodomain proteolysis	3/180	23/28943	0.000382253183778136	0.00658729428341476	0.00461343719992883	Adam8/Tnfrsf1b/Apoe	3	1.30434782608696
GO:0007189	BP	GO:0007189	adenylate cyclase-activating G protein-coupled receptor signaling pathway	6/180	153/28943	0.000403534450203011	0.00688865367983312	0.00482449542659483	Adcyap1/Abca1/Adm/Adcy9/Rit2/Lpar1	6	0.392156862745098
GO:0016032	BP	GO:0016032	viral process	9/180	355/28943	0.000403970786873849	0.00688865367983312	0.00482449542659483	Jun/Adarb1/Nrp1/Apoe/Ctsl/Ifih1/Lgals1/Mdfic/Trim25	9	0.253521126760563
GO:0001662	BP	GO:0001662	behavioral fear response	4/180	56/28943	0.000412859832630514	0.00700356497332074	0.00490497399841186	Adcyap1/Bdnf/Apoe/Penk	4	0.714285714285714

GO:0032092	BP	GO:0032092	positive regulation of protein binding	5/180	101/28943	0.000430333996720396	0.00722473106865118	0.00505986853441724	Mmp9/B2m/Bdnf/Apoe/Anxa2	5	0.495049504950495
GO:0032370	BP	GO:0032370	positive regulation of lipid transport	5/180	101/28943	0.000430333996720396	0.00722473106865118	0.00505986853441724	Abca1/Nucb2/Apoe/Anxa2/Pla2g4a	5	0.495049504950495
GO:0032930	BP	GO:0032930	positive regulation of superoxide anion generation	3/180	24/28943	0.000434868209219055	0.00722635590523705	0.00506100649504668	Tyrobp/Itgb2/Mapt	3	1.25
GO:1990000	BP	GO:1990000	amyloid fibril formation	3/180	24/28943	0.000434868209219055	0.00722635590523705	0.00506100649504668	B2m/Mapt/Apoe	3	1.25
GO:0002209	BP	GO:0002209	behavioral defense response	4/180	57/28943	0.000441879087111268	0.00723216174231859	0.00506507263565256	Adcyap1/Bdnf/Apoe/Penk	4	0.701754385964912
GO:0031646	BP	GO:0031646	positive regulation of nervous system process	4/180	57/28943	0.000441879087111268	0.00723216174231859	0.00506507263565256	Cst7/Itgax/Tnfrsf1b/Egr2	4	0.701754385964912
GO:0051353	BP	GO:0051353	positive regulation of oxidoreductase activity	4/180	57/28943	0.000441879087111268	0.00723216174231859	0.00506507263565256	Apoe/Npr3/Nus1/Agtr2	4	0.701754385964912
GO:0001894	BP	GO:0001894	tissue homeostasis	8/180	286/28943	0.000444968877585291	0.00724631817147646	0.00507498714593329	Ctss/Adam8/Car2/Col14a1/Rbp4/Pdk4/Aldh1a1/Pla2g4a	8	0.27972027972028
GO:0051147	BP	GO:0051147	regulation of muscle cell differentiation	6/180	157/28943	0.000462786486494206	0.0074989830174708	0.00525194195461087	Ankrd17/Fgf9/Fdps/Igf2/Bdnf/Efnb2	6	0.382165605095541
GO:0042311	BP	GO:0042311	vasodilation	4/180	58/28943	0.000472323887604212	0.00757373534393974	0.00530429503751189	Adcyap1/Apoe/Npr3/Agtr2	4	0.689655172413793
GO:0098754	BP	GO:0098754	detoxification	4/180	58/28943	0.000472323887604212	0.00757373534393974	0.00530429503751189	Mt2/Mt1/Prdx6/Aldh1a1	4	0.689655172413793
GO:0042063	BP	GO:0042063	gliogenesis	9/180	363/28943	0.000474375809076975	0.00757373534393974	0.00530429503751189	Adcyap1/Ccl2/C1qa/Sox11/Gap43/Tnfrsf1b/Enpp2/Gfap/Egr2	9	0.247933884297521

GO:1903828	BP	GO:1903828	negative regulation of protein localization	7/180	221/28943	0.000486831121282494	0.00773467786349797	0.0054170117577364	Sfrp1/Cyp51/Vsnl1/Mapt/Apoe/Inhbb/Mdfic	7	0.316742081447964
GO:0007263	BP	GO:0007263	nitric oxide mediated signal transduction	3/180	25/28943	0.00049191511054745	0.00774439148060312	0.0054238147272973	Mt2/Mt1/Apoe	3	1.2
GO:0042116	BP	GO:0042116	macrophage activation	5/180	104/28943	0.000492198046203514	0.00774439148060312	0.0054238147272973	Jun/Cst7/C1qa/Tyrobp/Pla2g4a	5	0.480769230769231
GO:0030072	BP	GO:0030072	peptide hormone secretion	8/180	291/28943	0.000498805559689605	0.00780144446100651	0.00546377200426647	Adcyap1/Sfrp1/Vsnl1/Oxct1/Nucb2/Rbp4/Inhbb/Pclo	8	0.274914089347079
GO:0052547	BP	GO:0052547	regulation of peptidase activity	10/180	445/28943	0.000500614643030507	0.00780144446100651	0.00546377200426647	Cst7/Mmp9/Cst3/Ctsd/Laptm5/Serpina3n/Serpinb6a/Serpina1b/Serpina1a/Serpina1c	10	0.224719101123595
GO:0051346	BP	GO:0051346	negative regulation of hydrolase activity	9/180	366/28943	0.000503249911569889	0.00780516648563394	0.00546637873870651	Adcyap1/Cst7/Mmp9/Cst3/Serpina3n/Serpinb6a/Serpina1b/Serpina1a/Serpina1c	9	0.245901639344262
GO:0010634	BP	GO:0010634	positive regulation of epithelial cell migration	6/180	160/28943	0.000511512315130383	0.00786321384688465	0.00550703243417386	Jun/Mmp9/Enpp2/Igf2/Nrp1/Nus1	6	0.375
GO:0006644	BP	GO:0006644	phospholipid metabolic process	9/180	367/28943	0.000513189489160293	0.00786321384688465	0.00550703243417386	Idi1/Fdft1/Piga/Enpp2/Fdps/Sacm1l/Prdx6/Nus1/Pla2g4a	9	0.245231607629428
GO:0046849	BP	GO:0046849	bone remodeling	5/180	105/28943	0.000514235354432432	0.00786321384688465	0.00550703243417386	Ctss/Adam8/Sfrp1/Car2/Pdk4	5	0.476190476190476
GO:0031641	BP	GO:0031641	regulation of myelination	4/180	60/28943	0.000537648094850255	0.00816647080134869	0.00571941962300555	Cst7/Itgax/Tnfrsf1b/Egr2	4	0.666666666666667
GO:0010810	BP	GO:0010810	regulation of cell-substrate adhesion	7/180	225/28943	0.000541589712339981	0.00816647080134869	0.00571941962300555	Mmp12/Hoxa7/Npy/S100a10/Enpp2/Nrp1/Lgals1	7	0.311111111111111
GO:0050866	BP	GO:0050866	negative regulation of cell activation	7/180	225/28943	0.000541589712339981	0.00816647080134869	0.00571941962300555	Cst7/Sox11/Sfrp1/Tyrobp/Lgals3/Laptm5/Apoe	7	0.311111111111111

GO:0002523	BP	GO:0002523	leukocyte migration involved in inflammatory response	3/180	26/28943	0.000553543079252042	0.00826079572783358	0.00578548045253636	Adam8/Ccl2/Itgb2	3	1.15384615384615
GO:0007517	BP	GO:0007517	muscle organ development	9/180	371/28943	0.000554574523026541	0.00826079572783358	0.00578548045253636	Atf3/Dsp/Sox11/Nr1d2/Adarb1/Lmna/Igf2/Bdnf/Egr2	9	0.242587601078167
GO:0033673	BP	GO:0033673	negative regulation of kinase activity	7/180	226/28943	0.000556017149848381	0.00826079572783358	0.00578548045253636	Gadd45a/Sfrp1/Pkib/Adarb1/Mapt/Apoe/Chordc1	7	0.309734513274336
GO:0003300	BP	GO:0003300	cardiac muscle hypertrophy	5/180	107/28943	0.000560526820955241	0.00826079572783358	0.00578548045253636	Tnfrsf1b/Fdps/Lmna/Col14a1/Agtr2	5	0.467289719626168
GO:0071901	BP	GO:0071901	negative regulation of protein serine/threonine kinase activity	5/180	107/28943	0.000560526820955241	0.00826079572783358	0.00578548045253636	Gadd45a/Sfrp1/Pkib/Apoe/Chordc1	5	0.467289719626168
GO:0010976	BP	GO:0010976	positive regulation of neuron projection development	7/180	227/28943	0.000570748777207244	0.00832580545246977	0.00583101026630965	Adcyap1/Rit2/Nrp1/Bdnf/Mapt/Mark2/Apoe	7	0.308370044052863
GO:0070374	BP	GO:0070374	positive regulation of ERK1 and ERK2 cascade	7/180	227/28943	0.000570748777207244	0.00832580545246977	0.00583101026630965	Adcyap1/Jun/Ccl2/Npy/Sema7a/Nrp1/Apoe	7	0.308370044052863
GO:0010812	BP	GO:0010812	negative regulation of cell-substrate adhesion	4/180	61/28943	0.000572606822644528	0.00832580545246977	0.00583101026630965	Mmp12/Hoxa7/Enpp2/Lgals1	4	0.655737704918033
GO:0032755	BP	GO:0032755	positive regulation of interleukin-6 production	5/180	108/28943	0.000584812371907499	0.00846548397912322	0.00592883466630901	Adcyap1/Tyrobp/Laptm5/Ifih1/Ccr5	5	0.462962962962963
GO:0042596	BP	GO:0042596	fear response	4/180	62/28943	0.000609149711571774	0.00874009079554744	0.00612115661941124	Adcyap1/Bdnf/Apoe/Penk	4	0.645161290322581
GO:0048260	BP	GO:0048260	positive regulation of receptor-mediated endocytosis	4/180	62/28943	0.000609149711571774	0.00874009079554744	0.00612115661941124	B2m/Sfrp4/Anxa2/Cd63	4	0.645161290322581
GO:0032367	BP	GO:0032367	intracellular cholesterol transport	3/180	27/28943	0.000619898054028729	0.00885529807882268	0.00620184248882852	Abca1/Anxa2/Nus1	3	1.11111111111111

GO:0001558	BP	GO:0001558	regulation of cell growth	10/180	459/28943	0.000635564271381744	0.00903944468074384	0.0063308102784842	Sfrp1/Fdps/Sema7a/Sgk1/Col14a1/Nrp1/Bdnf/Mapt/Apoe/Ccr5	10	0.217864923747277
GO:0046850	BP	GO:0046850	regulation of bone remodeling	4/180	63/28943	0.000647316698871914	0.00916656734011228	0.00641984112794251	Adam8/Sfrp1/Car2/Pdk4	4	0.634920634920635
GO:0042060	BP	GO:0042060	wound healing	9/180	380/28943	0.000657760820020602	0.00927414281734675	0.00649518203228363	Dsp/Mmp12/Ccl2/Procr/S100a10/Paps s2/Apoe/Anxa2/Pla2g4a	9	0.236842105263158
GO:0014897	BP	GO:0014897	striated muscle hypertrophy	5/180	111/28943	0.000662419083469022	0.00929956446059743	0.00651298617911692	Tnfrsf1b/Fdps/Lmna/Col14a1/Agtr2	5	0.45045045045045
GO:0031349	BP	GO:0031349	positive regulation of defense response	8/180	305/28943	0.000678193557631053	0.0094579278882899	0.00662389662231121	Ctss/Mmp12/Adam8/Tyrobp/Penk/Lgals1/Ccr5/Pla2g4a	8	0.262295081967213
GO:0090278	BP	GO:0090278	negative regulation of peptide hormone secretion	4/180	64/28943	0.000687147809108633	0.0094579278882899	0.00662389662231121	Sfrp1/Vsnl1/Nucb2/Inhbb	4	0.625
GO:0030301	BP	GO:0030301	cholesterol transport	5/180	112/28943	0.00068992499019752	0.0094579278882899	0.00662389662231121	Abca1/Msr1/Apoe/Anxa2/Nus1	5	0.446428571428571
GO:0031639	BP	GO:0031639	plasminogen activation	3/180	28/28943	0.000691122762484801	0.0094579278882899	0.00662389662231121	Ctsz/S100a10/Clec3b	3	1.07142857142857
GO:0032928	BP	GO:0032928	regulation of superoxide anion generation	3/180	28/28943	0.000691122762484801	0.0094579278882899	0.00662389662231121	Tyrobp/Itgb2/Mapt	3	1.07142857142857
GO:0051894	BP	GO:0051894	positive regulation of focal adhesion assembly	3/180	28/28943	0.000691122762484801	0.0094579278882899	0.00662389662231121	S100a10/Enpp2/Nrp1	3	1.07142857142857
GO:0030073	BP	GO:0030073	insulin secretion	7/180	235/28943	0.000700112945866013	0.00950111610285669	0.00665414365654232	Adcyap1/Sfrp1/Vsnl1/Oxct1/Rbp4/Inhbb/Pclo	7	0.297872340425532
GO:0090276	BP	GO:0090276	regulation of peptide hormone secretion	7/180	235/28943	0.000700112945866013	0.00950111610285669	0.00665414365654232	Adcyap1/Sfrp1/Vsnl1/Oxct1/Nucb2/Rbp4/Inhbb	7	0.297872340425532

GO:0071248	BP	GO:0071248	cellular response to metal ion	6/180	170/28943	0.000703315573165366	0.00950497436431368	0.00665684580497641	Jun/Mt2/Mmp9/B2m/Mt1/Ank3	6	0.352941176470588
GO:0014896	BP	GO:0014896	muscle hypertrophy	5/180	113/28943	0.000718276493748778	0.00954867975567253	0.00668745504598755	Tnfrsf1b/Fdps/Lmna/Col14a1/Agtr2	5	0.442477876106195
GO:0055017	BP	GO:0055017	cardiac muscle tissue growth	5/180	113/28943	0.000718276493748778	0.00954867975567253	0.00668745504598755	Fgf9/Fdps/Col14a1/Rbp4/Agtr2	5	0.442477876106195
GO:1902106	BP	GO:1902106	negative regulation of leukocyte differentiation	5/180	113/28943	0.000718276493748778	0.00954867975567253	0.00668745504598755	Hoxa7/Sfrp1/C1qc/Tmem176a/Tmem176b	5	0.442477876106195
GO:1903557	BP	GO:1903557	positive regulation of tumor necrosis factor superfamily cytokine production	5/180	113/28943	0.000718276493748778	0.00954867975567253	0.00668745504598755	Adam8/Ccl2/Tyrobp/Ifih1/Ccr5	5	0.442477876106195
GO:0042098	BP	GO:0042098	T cell proliferation	7/180	237/28943	0.000735826857695279	0.00974222794924197	0.00682300727696051	Igfbp2/Lgals3/Msn/Tnfrsf1b/Itgb2/Laptm5/Igf2	7	0.29535864978903
GO:0002792	BP	GO:0002792	negative regulation of peptide secretion	4/180	66/28943	0.000771962857987051	0.0101106744205383	0.00708105019773006	Sfrp1/Vsnl1/Nucb2/Inhbb	4	0.606060606060606
GO:0051148	BP	GO:0051148	negative regulation of muscle cell differentiation	4/180	66/28943	0.000771962857987051	0.0101106744205383	0.00708105019773006	Ankrd17/Fgf9/Igf2/Bdnf	4	0.606060606060606
GO:0042445	BP	GO:0042445	hormone metabolic process	7/180	239/28943	0.000772968354533016	0.0101106744205383	0.00708105019773006	Adm/Ugt1a1/Papss2/Igf2/Rbp4/Ctsl/Aldh1a1	7	0.292887029288703
GO:0002791	BP	GO:0002791	regulation of peptide secretion	7/180	240/28943	0.000792087001757364	0.0103193094588949	0.00722716855919245	Adcyap1/Sfrp1/Vsnl1/Oxct1/Nucb2/Rbp4/Inhbb	7	0.291666666666667
GO:0015918	BP	GO:0015918	sterol transport	5/180	116/28943	0.000808569233807842	0.0104893195014622	0.00734623575446198	Abca1/Msr1/Apoe/Anxa2/Nus1	5	0.431034482758621
GO:0008406	BP	GO:0008406	gonad development	7/180	241/28943	0.000811577683257129	0.0104893195014622	0.00734623575446198	Adcyap1/Sfrp1/Fgf9/Serpinb6a/Fndc3a/Rbp4/Pla2g4a	7	0.29045643153527

GO:1903707	BP	GO:1903707	negative regulation of hemopoiesis	5/180	117/28943	0.000840468270268731	0.0107188357287872	0.00750697833792214	Hoxa7/Sfrp1/C1qc/Tmem176a/Tmem176b	5	0.427350427350427
GO:0010955	BP	GO:0010955	negative regulation of protein processing	3/180	30/28943	0.000848736482630784	0.0107188357287872	0.00750697833792214	Cst7/Ctsz/Ctla2a	3	1
GO:0021602	BP	GO:0021602	cranial nerve morphogenesis	3/180	30/28943	0.000848736482630784	0.0107188357287872	0.00750697833792214	Adarb1/Nrp1/Egr2	3	1
GO:0032366	BP	GO:0032366	intracellular sterol transport	3/180	30/28943	0.000848736482630784	0.0107188357287872	0.00750697833792214	Abca1/Anxa2/Nus1	3	1
GO:1903318	BP	GO:1903318	negative regulation of protein maturation	3/180	30/28943	0.000848736482630784	0.0107188357287872	0.00750697833792214	Cst7/Ctsz/Ctla2a	3	1
GO:0042326	BP	GO:0042326	negative regulation of phosphorylation	9/180	394/28943	0.000849081860002176	0.0107188357287872	0.00750697833792214	Jun/Gadd45a/Sfrp1/Pkib/Adarb1/Ddit4/Mapt/Apoe/Chordc1	9	0.228426395939086
GO:0097006	BP	GO:0097006	regulation of plasma lipoprotein particle levels	4/180	68/28943	0.00086391632902087	0.0108279111063103	0.00758336970327047	Abca1/Msr1/Apoe/Anxa2	4	0.588235294117647
GO:0006606	BP	GO:0006606	protein import into nucleus	6/180	177/28943	0.00086769567047804	0.0108279111063103	0.00758336970327047	Mmp12/Kpna1/Fgf9/Lmna/Mdfic/Agtr2	6	0.338983050847458
GO:0051100	BP	GO:0051100	negative regulation of binding	6/180	177/28943	0.00086769567047804	0.0108279111063103	0.00758336970327047	Jun/Sox11/Ctsz/B2m/Nes/Ckmt1	6	0.338983050847458
GO:0045137	BP	GO:0045137	development of primary sexual characteristics	7/180	245/28943	0.000893363719904779	0.0111056703653812	0.00777789948185478	Adcyap1/Sfrp1/Fgf9/Serpinb6a/Fndc3a/Rbp4/Pla2g4a	7	0.285714285714286
GO:0002573	BP	GO:0002573	myeloid leukocyte differentiation	7/180	246/28943	0.000914792111540846	0.0112859011639717	0.00790412482340117	Jun/Adam8/Hoxa7/Sfrp1/C1qc/Tyrobp/Car2	7	0.284552845528455
GO:0050870	BP	GO:0050870	positive regulation of T cell activation	7/180	246/28943	0.000914792111540846	0.0112859011639717	0.00790412482340117	Adam8/Ccl2/Igfbp2/B2m/Igf2/Lgals1/Efnb2	7	0.284552845528455

GO:0002699	BP	GO:0002699	positive regulation of immune effector process	8/180	320/28943	0.000924761388271578	0.0113658409117001	0.00796011093697918	Ccl2/Tyrobp/Ankrd17/B2m/Itgb2/Sema7a/Laptm5/Rbp4	8	0.25
GO:0032801	BP	GO:0032801	receptor catabolic process	3/180	31/28943	0.000935395263414522	0.0114533171915079	0.00802137529014353	Laptm5/Apoe/Anxa2	3	0.967741935483871
GO:0007611	BP	GO:0007611	learning or memory	8/180	321/28943	0.000943459315340756	0.011508790224962	0.008060226045115	Jun/Specc1/B2m/Sgk1/Igf2/Bdnf/Mapt/Apoe	8	0.249221183800623
GO:0051170	BP	GO:0051170	import into nucleus	6/180	181/28943	0.000974070819388855	0.0118378681296623	0.00829069703698212	Mmp12/Kpna1/Fgf9/Lmna/Mdfic/Agtr2	6	0.331491712707182
GO:0070372	BP	GO:0070372	regulation of ERK1 and ERK2 cascade	8/180	326/28943	0.00104154948741372	0.012610879853184	0.00883207880737836	Atf3/Adcyap1/Jun/Ccl2/Npy/Sema7a/Nrp1/Apoe	8	0.245398773006135
GO:0060419	BP	GO:0060419	heart growth	5/180	123/28943	0.00105195876973809	0.0126897396779146	0.00888730859268005	Fgf9/Fdps/Col14a1/Rbp4/Agtr2	5	0.40650406504065
GO:0007178	BP	GO:0007178	transmembrane receptor protein serine/threonine kinase signaling pathway	9/180	407/28943	0.00106514541671779	0.0127252076196408	0.00891214870376733	Jun/Ccl2/Sox11/Sfrp1/Fgf9/Nrep/Sfrp4/Htra1/Inhbb	9	0.221130221130221
GO:0032371	BP	GO:0032371	regulation of sterol transport	4/180	72/28943	0.00107052713778986	0.0127252076196408	0.00891214870376733	Abca1/Apoe/Anxa2/Nus1	4	0.555555555555556
GO:0032374	BP	GO:0032374	regulation of cholesterol transport	4/180	72/28943	0.00107052713778986	0.0127252076196408	0.00891214870376733	Abca1/Apoe/Anxa2/Nus1	4	0.555555555555556
GO:0048678	BP	GO:0048678	response to axon injury	4/180	72/28943	0.00107052713778986	0.0127252076196408	0.00891214870376733	Jun/Tyrobp/Gap43/Nrep	4	0.555555555555556
GO:0050670	BP	GO:0050670	regulation of lymphocyte proliferation	7/180	253/28943	0.00107638440191971	0.0127483054438272	0.00892832535487082	Sox11/Igfbp2/Tyrobp/Lgals3/Tnfrsf1b/Laptm5/Igf2	7	0.276679841897233
GO:0032963	BP	GO:0032963	collagen metabolic process	5/180	124/28943	0.00109071628108369	0.0127786436240632	0.008949572817525	Ctss/Mmp12/Ccl2/Mmp9/Ctsl	5	0.403225806451613

GO:0046620	BP	GO:0046620	regulation of organ growth	5/180	124/28943	0.0010907162 8108369	0.0127786436 240632	0.008949572817525	Fgf9/Fdps/Col14a1/Igf2/Rbp4	5	0.403225806451613
GO:0048145	BP	GO:0048145	regulation of fibroblast proliferation	5/180	124/28943	0.0010907162 8108369	0.0127786436 240632	0.008949572817525	Jun/Sfrp1/Mmp9/Anxa2/Agtr2	5	0.403225806451613
GO:0150117	BP	GO:0150117	positive regulation of cell-substrate junction organization	3/180	33/28943	0.0011250681 6462254	0.0130571341 423522	0.00914461473634998	S100a10/Enpp2/Nrp1	3	0.909090909090909
GO:0048259	BP	GO:0048259	regulation of receptor-mediated endocytosis	5/180	125/28943	0.0011305225 1401392	0.0130571341 423522	0.00914461473634998	B2m/Sfrp4/Anxa2/Cd63/Efnb2	5	0.4
GO:1901890	BP	GO:1901890	positive regulation of cell junction assembly	5/180	125/28943	0.0011305225 1401392	0.0130571341 423522	0.00914461473634998	S100a10/Enpp2/Nrp1/Bdnf/Cbln1	5	0.4
GO:2000177	BP	GO:2000177	regulation of neural precursor cell proliferation	5/180	125/28943	0.0011305225 1401392	0.0130571341 423522	0.00914461473634998	Adcyap1/Ctsz/Nes/Bdnf/Ccr5	5	0.4
GO:0048640	BP	GO:0048640	negative regulation of developmental growth	5/180	126/28943	0.0011713949 3742111	0.0134813897 921574	0.00944174383258897	H19/Sema7a/Nrp1/Rbp4/Ccr5	5	0.396825396825397
GO:0032944	BP	GO:0032944	regulation of mononuclear cell proliferation	7/180	258/28943	0.0012049405 6734505	0.0137919854 282608	0.00965927069568091	Sox11/Igfbp2/Tyrobp/Lgals3/Tnfrsf1b/Laptm5/Igf2	7	0.271317829457364
GO:0006820	BP	GO:0006820	anion transport	10/180	500/28943	0.0012133317 2128703	0.0137919854 282608	0.00965927069568091	Slc25a14/Car2/Sfrp4/Clic1/Bdnf/Gfap/Clca1/Agtr2/Slc4a3/Pla2g4a	10	0.2
GO:0045807	BP	GO:0045807	positive regulation of endocytosis	5/180	127/28943	0.0012133510 9201759	0.0137919854 282608	0.00965927069568091	B2m/Sfrp4/Apoe/Anxa2/Cd63	5	0.393700787401575
GO:0051146	BP	GO:0051146	striated muscle cell differentiation	8/180	334/28943	0.0012153207 9149857	0.0137919854 282608	0.00965927069568091	Fdps/Lmna/Col14a1/Igf2/Bdnf/Cd53/Agtr2/Efnb2	8	0.239520958083832
GO:0055094	BP	GO:0055094	response to lipoprotein particle	3/180	34/28943	0.0012283338 8079544	0.0138370589 754983	0.00969083812998879	Cd68/Itgb2/Apoe	3	0.882352941176471

GO:0090322	BP	GO:0090322	regulation of superoxide metabolic process	3/180	34/28943	0.00122833388079544	0.0138370589754983	0.00969083812998879	Tyrobp/Itgb2/Mapt	3		0.882352941176471
GO:0007411	BP	GO:0007411	axon guidance	7/180	259/28943	0.00123203779640605	0.0138370589754983	0.00969083812998879	Gap43/Sema7a/Nrp1/Bdnf/Ank3/Egr2/Efnb2	7		0.27027027027027
GO:0050680	BP	GO:0050680	negative regulation of epithelial cell proliferation	6/180	190/28943	0.00124998600401309	0.0139903931789369	0.00979822633639487	Ccl2/Sfrp1/B2m/Apoe/Ctsl/Efnb2	6		0.315789473684211
GO:0001952	BP	GO:0001952	regulation of cell-matrix adhesion	5/180	128/28943	0.00125640858785954	0.01400188014854	0.00980627128028252	Mmp12/Hoxa7/S100a10/Enpp2/Nrp1	5		0.390625
GO:0097485	BP	GO:0097485	neuron projection guidance	7/180	260/28943	0.00125961034188585	0.01400188014854	0.00980627128028252	Gap43/Sema7a/Nrp1/Bdnf/Ank3/Egr2/Efnb2	7		0.269230769230769
GO:0010001	BP	GO:0010001	glial cell differentiation	7/180	261/28943	0.00128766397658341	0.0142166832940073	0.00995670949957625	C1qa/Sox11/Gap43/Tnfrsf1b/Enpp2/Gfap/Egr2	7		0.268199233716475
GO:0019058	BP	GO:0019058	viral life cycle	7/180	261/28943	0.00128766397658341	0.0142166832940073	0.00995670949957625	Adarb1/Nrp1/Apoe/Ctsl/Ifih1/Lgals1/Trim25	7		0.268199233716475
GO:0051348	BP	GO:0051348	negative regulation of transferase activity	7/180	262/28943	0.00131620450547776	0.0144826961970982	0.0101430126720139	Gadd45a/Sfrp1/Pkib/Adarb1/Mapt/Apoe/Chordc1	7		0.267175572519084
GO:0032386	BP	GO:0032386	regulation of intracellular transport	8/180	339/28943	0.00133517125123406	0.0146169561615618	0.0102370421608856	Ccl2/Msn/Mapt/Anxa2/Ank3/Mdfic/Nus1/Agtr2	8		0.23598820058997
GO:0046475	BP	GO:0046475	glycerophospholipid catabolic process	3/180	35/28943	0.00133738192697126	0.0146169561615618	0.0102370421608856	Enpp2/Prdx6/Pla2g4a	3		0.857142857142857
GO:0007416	BP	GO:0007416	synapse assembly	6/180	194/28943	0.00139023211701491	0.0150932866837253	0.0105706420967415	Gap43/Bdnf/Mapt/Cbln1/Pclo/Efnb2	6		0.309278350515464
GO:0051099	BP	GO:0051099	positive regulation of binding	6/180	194/28943	0.00139023211701491	0.0150932866837253	0.0105706420967415	Mmp9/S100a10/B2m/Bdnf/Apoe/Anxa2	6		0.309278350515464

GO:0033002	BP	GO:0033002	muscle cell proliferation	7/180	266/28943	0.0014353527 7561228	0.0154496918 29342	0.0108202518281868	Jun/Mmp9/Kpna1/Fgf9/Apoe/Npr3/R bp4	7	0.263157894736842
GO:0006875	BP	GO:0006875	cellular metal ion homeostasis	9/180	425/28943	0.0014358294 614593	0.0154496918 29342	0.0108202518281868	Ccl2/Mt2/Abcb7/Sgk1/Mt1/Nucb2/Ap oe/Ccr5/Agtr2	9	0.211764705882353
GO:0006721	BP	GO:0006721	terpenoid metabolic process	4/180	78/28943	0.0014420344 8453177	0.0154496918 29342	0.0108202518281868	Fdft1/Fdps/Rbp4/Aldh1a1	4	0.512820512820513
GO:2000379	BP	GO:2000379	positive regulation of reactive oxygen species metabolic process	4/180	78/28943	0.0014420344 8453177	0.0154496918 29342	0.0108202518281868	Gadd45a/Tyrobp/Itgb2/Mapt	4	0.512820512820513
GO:0072594	BP	GO:0072594	establishment of protein localization to organelle	9/180	428/28943	0.0015066426 2262815	0.0160064492 729254	0.0112101790716123	Mmp12/Cd68/Kpna1/Fgf9/Laptm5/L mna/Mapt/Mdfic/Agtr2	9	0.210280373831776
GO:0045453	BP	GO:0045453	bone resorption	4/180	79/28943	0.0015116533 0749736	0.0160064492 729254	0.0112101790716123	Ctss/Adam8/Car2/Pdk4	4	0.506329113924051
GO:0045669	BP	GO:0045669	positive regulation of osteoblast differentiation	4/180	79/28943	0.0015116533 0749736	0.0160064492 729254	0.0112101790716123	Cebpd/Sox11/Lmna/Clic1	4	0.506329113924051
GO:0001933	BP	GO:0001933	negative regulation of protein phosphorylation	8/180	346/28943	0.0015185731 7326802	0.0160064492 729254	0.0112101790716123	Jun/Gadd45a/Sfrp1/Pkib/Adarb1/Ddit 4/Apoe/Chordc1	8	0.23121387283237
GO:0060249	BP	GO:0060249	anatomical structure homeostasis	8/180	346/28943	0.0015185731 7326802	0.0160064492 729254	0.0112101790716123	Ctss/Adam8/Car2/Col14a1/Rbp4/Pdk 4/Aldh1a1/Pla2g4a	8	0.23121387283237
GO:0035640	BP	GO:0035640	exploration behavior	3/180	37/28943	0.0015732961 1186214	0.0164362222 362241	0.0115111722398389	Apoe/Penk/Agtr2	3	0.810810810810811
GO:0071402	BP	GO:0071402	cellular response to lipoprotein particle stimulus	3/180	37/28943	0.0015732961 1186214	0.0164362222 362241	0.0115111722398389	Cd68/Itgb2/Apoe	3	0.810810810810811
GO:0072001	BP	GO:0072001	renal system development	8/180	348/28943	0.0015744861 33774	0.0164362222 362241	0.0115111722398389	Sfrp1/Mmp9/Nrp1/Bdnf/Rbp4/Anxa4/ Agtr2/Efnb2	8	0.229885057471264

GO:1990138	BP	GO:1990138	neuron projection extension	6/180	200/28943	0.00162274152502784	0.0168379052165684	0.0117924924791312	Sema7a/Nrp1/Bdnf/Mapt/Apoe/Ccr5	6	0.3
GO:0044403	BP	GO:0044403	biological process involved in symbiotic interaction	7/180	272/28943	0.00162968859723096	0.0168379052165684	0.0117924924791312	Jun/Nrp1/Apoe/Ctsl/Gbp3/Lgals1/Trim25	7	0.257352941176471
GO:0070371	BP	GO:0070371	ERK1 and ERK2 cascade	8/180	350/28943	0.00163201975860984	0.0168379052165684	0.0117924924791312	Atf3/Adcyap1/Jun/Ccl2/Npy/Sema7a/Nrp1/Apoe	8	0.228571428571429
GO:0007568	BP	GO:0007568	aging	4/180	81/28943	0.00165785127748668	0.0168379052165684	0.0117924924791312	C1qa/Mmp9/Cd68/Apoe	4	0.493827160493827
GO:0001906	BP	GO:0001906	cell killing	7/180	273/28943	0.00166397705162864	0.0168379052165684	0.0117924924791312	Ccl2/Tyrobp/Lgals3/B2m/Igf2/Lyz1/Lyz2	7	0.256410256410256
GO:0090092	BP	GO:0090092	regulation of transmembrane receptor protein serine/threonine kinase signaling pathway	7/180	273/28943	0.00166397705162864	0.0168379052165684	0.0117924924791312	Sox11/Sfrp1/Fgf9/Nrep/Sfrp4/Htra1/Inhbb	7	0.256410256410256
GO:0021612	BP	GO:0021612	facial nerve structural organization	2/180	10/28943	0.00167500193127669	0.0168379052165684	0.0117924924791312	Nrp1/Egr2	2	2
GO:0034380	BP	GO:0034380	high-density lipoprotein particle assembly	2/180	10/28943	0.00167500193127669	0.0168379052165684	0.0117924924791312	Abca1/Apoe	2	2
GO:0061687	BP	GO:0061687	detoxification of inorganic compound	2/180	10/28943	0.00167500193127669	0.0168379052165684	0.0117924924791312	Mt2/Mt1	2	2
GO:0097501	BP	GO:0097501	stress response to metal ion	2/180	10/28943	0.00167500193127669	0.0168379052165684	0.0117924924791312	Mt2/Mt1	2	2
GO:0098883	BP	GO:0098883	synapse pruning	2/180	10/28943	0.00167500193127669	0.0168379052165684	0.0117924924791312	C1qa/C1qc	2	2
GO:2000646	BP	GO:2000646	positive regulation of receptor catabolic process	2/180	10/28943	0.00167500193127669	0.0168379052165684	0.0117924924791312	Laptm5/Apoe	2	2

GO:0001953	BP	GO:0001953	negative regulation of cell-matrix adhesion	3/180	38/28943	0.0017003907026684	0.0168422612368208	0.0117955432348757	Mmp12/Hoxa7/Enpp2	3	0.789473684210526
GO:0034381	BP	GO:0034381	plasma lipoprotein particle clearance	3/180	38/28943	0.0017003907026684	0.0168422612368208	0.0117955432348757	Msr1/Apoe/Anxa2	3	0.789473684210526
GO:0050869	BP	GO:0050869	negative regulation of B cell activation	3/180	38/28943	0.0017003907026684	0.0168422612368208	0.0117955432348757	Sfrp1/Tyrobp/Laptm5	3	0.789473684210526
GO:0002695	BP	GO:0002695	negative regulation of leukocyte activation	6/180	202/28943	0.00170646183854801	0.0168422612368208	0.0117955432348757	Cst7/Sox11/Sfrp1/Tyrobp/Lgals3/Laptm5	6	0.297029702970297
GO:0006469	BP	GO:0006469	negative regulation of protein kinase activity	6/180	202/28943	0.00170646183854801	0.0168422612368208	0.0117955432348757	Gadd45a/Sfrp1/Pkib/Adarb1/Apoe/Chordc1	6	0.297029702970297
GO:0051495	BP	GO:0051495	positive regulation of cytoskeleton organization	6/180	202/28943	0.00170646183854801	0.0168422612368208	0.0117955432348757	Slain1/S100a10/Nes/Nrp1/Mapt/Lpar1	6	0.297029702970297
GO:0046578	BP	GO:0046578	regulation of Ras protein signal transduction	6/180	203/28943	0.00174953238000276	0.0171633342218945	0.0120204079501141	Abca1/Arhgef3/Rit2/Nrp1/Apoe/Lpar1	6	0.295566502463054
GO:0048771	BP	GO:0048771	tissue remodeling	6/180	203/28943	0.00174953238000276	0.0171633342218945	0.0120204079501141	Ctss/Adam8/Sfrp1/Mmp9/Car2/Pdk4	6	0.295566502463054
GO:0055021	BP	GO:0055021	regulation of cardiac muscle tissue growth	4/180	83/28943	0.00181359431850655	0.0177383684545821	0.0124231354139519	Fgf9/Fdps/Col14a1/Rbp4	4	0.481927710843373
GO:0060560	BP	GO:0060560	developmental growth involved in morphogenesis	7/180	278/28943	0.00184392838519824	0.017927387315196	0.0125555155089539	Sfrp1/Sema7a/Nrp1/Bdnf/Mapt/Apoe/Ccr5	7	0.251798561151079
GO:0071375	BP	GO:0071375	cellular response to peptide hormone stimulus	7/180	278/28943	0.00184392838519824	0.017927387315196	0.0125555155089539	Sgk1/Car2/Igf2/Bglap/Nucb2/Inhbb/Pdk4	7	0.251798561151079
GO:0031623	BP	GO:0031623	receptor internalization	5/180	140/28943	0.00186549707802686	0.0180831070926592	0.0126645744614167	Adm/Itgb2/Sfrp4/Cd63/Efnb2	5	0.357142857142857

GO:0050890	BP	GO:0050890	cognition	8/180	358/28943	0.00187902652765722	0.0181602059364378	0.0127185709368521	Jun/Specc1/B2m/Sgk1/Igf2/Bdnf/Mapt/Apoe	8	0.223463687150838
GO:0072507	BP	GO:0072507	divalent inorganic cation homeostasis	8/180	359/28943	0.00191187021882168	0.0184229624340302	0.0129025934730195	Ccl2/Mt2/Adm/Mt1/Nucb2/Apoe/Ank3/Ccr5	8	0.222841225626741
GO:0070663	BP	GO:0070663	regulation of leukocyte proliferation	7/180	280/28943	0.00191999674685604	0.0184466944085845	0.0129192142538024	Sox11/Igfbp2/Tyrobp/Lgals3/Tnfrsf1b/Laptm5/Igf2	7	0.25
GO:0034329	BP	GO:0034329	cell junction assembly	9/180	445/28943	0.00196285106734815	0.0187937293918114	0.013162261555534	Gap43/S100a10/Enpp2/Nrp1/Bdnf/Mapt/Cbln1/Pclo/Efnb2	9	0.202247191011236
GO:1904707	BP	GO:1904707	positive regulation of vascular associated smooth muscle cell proliferation	3/180	40/28943	0.00197340510538465	0.0187937293918114	0.013162261555534	Jun/Mmp9/Fgf9	3	0.75
GO:0034103	BP	GO:0034103	regulation of tissue remodeling	4/180	85/28943	0.00197919839772531	0.0187937293918114	0.013162261555534	Adam8/Sfrp1/Car2/Pdk4	4	0.470588235294118
GO:0045670	BP	GO:0045670	regulation of osteoclast differentiation	4/180	85/28943	0.00197919839772531	0.0187937293918114	0.013162261555534	Adam8/Sfrp1/Tyrobp/Car2	4	0.470588235294118
GO:0060900	BP	GO:0060900	embryonic camera-type eye formation	2/180	11/28943	0.00203886329320826	0.0191371116598827	0.0134027506639839	Sox11/Aldh1a1	2	1.81818181818182
GO:1900454	BP	GO:1900454	positive regulation of long-term synaptic depression	2/180	11/28943	0.00203886329320826	0.0191371116598827	0.0134027506639839	Mapt/Cbln1	2	1.81818181818182
GO:1902946	BP	GO:1902946	protein localization to early endosome	2/180	11/28943	0.00203886329320826	0.0191371116598827	0.0134027506639839	Msn/Nrp1	2	1.81818181818182
GO:1990440	BP	GO:1990440	positive regulation of transcription from RNA polymerase II promoter in response to endoplasmic reticulum stress	2/180	11/28943	0.00203886329320826	0.0191371116598827	0.0134027506639839	Atf3/Atf6	2	1.81818181818182
GO:0014015	BP	GO:0014015	positive regulation of gliogenesis	4/180	86/28943	0.00206579650823147	0.019334193181925	0.0135407774753103	Tnfrsf1b/Enpp2/Gfap/Egr2	4	0.465116279069767

GO:0006953	BP	GO:0006953	acute-phase response	3/180	41/28943	0.00211953679919423	0.019601775182339	0.0137281795711677	Serpina3n/Serpina1b/Ccr5	3	0.731707317073171
GO:0032365	BP	GO:0032365	intracellular lipid transport	3/180	41/28943	0.00211953679919423	0.019601775182339	0.0137281795711677	Abca1/Anxa2/Nus1	3	0.731707317073171
GO:0038179	BP	GO:0038179	neurotrophin signaling pathway	3/180	41/28943	0.00211953679919423	0.019601775182339	0.0137281795711677	Bdnf/Ddit4/Agtr2	3	0.731707317073171
GO:0016485	BP	GO:0016485	protein processing	7/180	285/28943	0.0021208319862712	0.019601775182339	0.0137281795711677	Cst7/Ctss/Ctsz/S100a10/Clec3b/Ctla2a/Ctsl	7	0.245614035087719
GO:0008361	BP	GO:0008361	regulation of cell size	6/180	211/28943	0.00212447855061887	0.019601775182339	0.0137281795711677	Sema7a/Nrp1/Bdnf/Mapt/Apoe/Ccr5	6	0.28436018957346
GO:0032869	BP	GO:0032869	cellular response to insulin stimulus	6/180	212/28943	0.00217529766087278	0.0200139674617589	0.014016860038987	Sgk1/Igf2/Bglap/Nucb2/Inhbb/Pdk4	6	0.283018867924528
GO:0010563	BP	GO:0010563	negative regulation of phosphorus metabolic process	9/180	453/28943	0.00221268861501043	0.0202436146603623	0.0141776943486919	Jun/Gadd45a/Sfrp1/Pkib/Adarb1/Ddit4/Mapt/Apoe/Chordc1	9	0.198675496688742
GO:0045936	BP	GO:0045936	negative regulation of phosphate metabolic process	9/180	453/28943	0.00221268861501043	0.0202436146603623	0.0141776943486919	Jun/Gadd45a/Sfrp1/Pkib/Adarb1/Ddit4/Mapt/Apoe/Chordc1	9	0.198675496688742
GO:0062012	BP	GO:0062012	regulation of small molecule metabolic process	8/180	368/28943	0.00222830896279803	0.0203294181844066	0.0142377871808442	Fdps/Ugt1a1/Igf2/Nucb2/Ddit4/Apoe/Pdk4/Pla2g4a	8	0.217391304347826
GO:0021675	BP	GO:0021675	nerve development	4/180	88/28943	0.00224677999159753	0.0204406771861262	0.0143157078559008	Adarb1/Nrp1/Bdnf/Egr2	4	0.454545454545455
GO:0038084	BP	GO:0038084	vascular endothelial growth factor signaling pathway	3/180	42/28943	0.00227222160061147	0.0206145564155754	0.0144374848512683	Nrp1/Cd63/Nus1	3	0.714285714285714
GO:0050729	BP	GO:0050729	positive regulation of inflammatory response	5/180	148/28943	0.00237646889796474	0.0215004422240865	0.0150579184265778	Ctss/Adam8/Lgals1/Ccr5/Pla2g4a	5	0.337837837837838

GO:1900026	BP	GO:1900026	positive regulation of substrate adhesion-dependent cell spreading	3/180	43/28943	0.00243155879128668	0.0218627096333899	0.0153116338172215	S100a10/Enpp2/Nrp1	3	0.697674418604651
GO:0042762	BP	GO:0042762	regulation of sulfur metabolic process	2/180	12/28943	0.0024366483257355	0.0218627096333899	0.0153116338172215	Abcb7/Pdk4	2	1.66666666666667
GO:1900272	BP	GO:1900272	negative regulation of long-term synaptic potentiation	2/180	12/28943	0.0024366483257355	0.0218627096333899	0.0153116338172215	Tyrobp/Apoe	2	1.66666666666667
GO:0035265	BP	GO:0035265	organ growth	6/180	218/28943	0.00249973578806523	0.0223671413783749	0.0156649145538964	Fgf9/Fdps/Col14a1/Igf2/Rbp4/Agtr2	6	0.275229357798165
GO:0055007	BP	GO:0055007	cardiac muscle cell differentiation	5/180	150/28943	0.00251867070309018	0.0224748232327801	0.0157403299599032	Fdps/Lmna/Col14a1/Agtr2/Efnb2	5	0.333333333333333
GO:0046888	BP	GO:0046888	negative regulation of hormone secretion	4/180	91/28943	0.00253830401491431	0.0225265835874003	0.0157765805257975	Sfrp1/Vsnl1/Nucb2/Inhbb	4	0.43956043956044
GO:0060420	BP	GO:0060420	regulation of heart growth	4/180	91/28943	0.00253830401491431	0.0225265835874003	0.0157765805257975	Fgf9/Fdps/Col14a1/Rbp4	4	0.43956043956044
GO:0003206	BP	GO:0003206	cardiac chamber morphogenesis	5/180	151/28943	0.00259204214150129	0.0228662971286292	0.0160145002271188	Dsp/Sox11/Fgf9/Nrp1/Rbp4	5	0.33112582781457
GO:0048147	BP	GO:0048147	negative regulation of fibroblast proliferation	3/180	44/28943	0.00259764505299135	0.0228662971286292	0.0160145002271188	Sfrp1/Mmp9/Agtr2	3	0.681818181818182
GO:0050918	BP	GO:0050918	positive chemotaxis	3/180	44/28943	0.00259764505299135	0.0228662971286292	0.0160145002271188	Scg2/Lgals3/Nrp1	3	0.681818181818182
GO:0015698	BP	GO:0015698	inorganic anion transport	5/180	152/28943	0.00266695198947603	0.0234131068186615	0.0163974605226119	Slc25a14/Car2/Sfrp4/Clic1/Clca1	5	0.328947368421053
GO:0045104	BP	GO:0045104	intermediate filament cytoskeleton organization	4/180	93/28943	0.00274646682905492	0.0237467398806753	0.0166311217366396	Dsp/Ina/Nes/Gfap	4	0.43010752688172

GO:0050878	BP	GO:0050878	regulation of body fluid levels	8/180	381/28943	0.0027565267 8607101	0.0237467398 806753	0.0166311217366396	Csn3/Adm/Procr/Papss2/Apoe/Anxa2/ Npr3/Pla2g4a	8	0.20997375328084
GO:0003298	BP	GO:0003298	physiological muscle hypertrophy	3/180	45/28943	0.0027705745 0250433	0.0237467398 806753	0.0166311217366396	Fdps/Col14a1/Agtr2	3	0.666666666666667
GO:0003301	BP	GO:0003301	physiological cardiac muscle hypertrophy	3/180	45/28943	0.0027705745 0250433	0.0237467398 806753	0.0166311217366396	Fdps/Col14a1/Agtr2	3	0.666666666666667
GO:0006509	BP	GO:0006509	membrane protein ectodomain proteolysis	3/180	45/28943	0.0027705745 0250433	0.0237467398 806753	0.0166311217366396	Adam8/Tnfrsf1b/Apoe	3	0.666666666666667
GO:0042554	BP	GO:0042554	superoxide anion generation	3/180	45/28943	0.0027705745 0250433	0.0237467398 806753	0.0166311217366396	Tyrobp/Itgb2/Mapt	3	0.666666666666667
GO:0045940	BP	GO:0045940	positive regulation of steroid metabolic process	3/180	45/28943	0.0027705745 0250433	0.0237467398 806753	0.0166311217366396	Fdps/Igf2/Apoe	3	0.666666666666667
GO:0046006	BP	GO:0046006	regulation of activated T cell proliferation	3/180	45/28943	0.0027705745 0250433	0.0237467398 806753	0.0166311217366396	Igfbp2/Laptm5/Igf2	3	0.666666666666667
GO:0061049	BP	GO:0061049	cell growth involved in cardiac muscle cell development	3/180	45/28943	0.0027705745 0250433	0.0237467398 806753	0.0166311217366396	Fdps/Col14a1/Agtr2	3	0.666666666666667
GO:0035914	BP	GO:0035914	skeletal muscle cell differentiation	4/180	94/28943	0.0028548062 6602442	0.0242619068 682854	0.0169919209422861	Atf3/Sox11/Nr1d2/Egr2	4	0.425531914893617
GO:0045103	BP	GO:0045103	intermediate filament-based process	4/180	94/28943	0.0028548062 6602442	0.0242619068 682854	0.0169919209422861	Dsp/Ina/Nes/Gfap	4	0.425531914893617
GO:0002674	BP	GO:0002674	negative regulation of acute inflammatory response	2/180	13/28943	0.0028679257 4279701	0.0242619068 682854	0.0169919209422861	Adcyap1/Npy	2	1.53846153846154
GO:0021604	BP	GO:0021604	cranial nerve structural organization	2/180	13/28943	0.0028679257 4279701	0.0242619068 682854	0.0169919209422861	Nrp1/Egr2	2	1.53846153846154

GO:0032060	BP	GO:0032060	bleb assembly	2/180	13/28943	0.00286792574279701	0.0242619068682854	0.0169919209422861	Prdx6/Lpar1	2	1.53846153846154
GO:0050900	BP	GO:0050900	leukocyte migration	8/180	385/28943	0.00293732105666438	0.0247032877403699	0.0173010437546278	Adam8/Ccl2/Hoxa7/Scg2/Mmp9/Lgals3/Msn/Itgb2	8	0.207792207792208
GO:0030517	BP	GO:0030517	negative regulation of axon extension	3/180	46/28943	0.00295043872612953	0.0247032877403699	0.0173010437546278	Sema7a/Nrp1/Ccr5	3	0.652173913043478
GO:0032373	BP	GO:0032373	positive regulation of sterol transport	3/180	46/28943	0.00295043872612953	0.0247032877403699	0.0173010437546278	Abca1/Apoe/Anxa2	3	0.652173913043478
GO:0032376	BP	GO:0032376	positive regulation of cholesterol transport	3/180	46/28943	0.00295043872612953	0.0247032877403699	0.0173010437546278	Abca1/Apoe/Anxa2	3	0.652173913043478
GO:0051341	BP	GO:0051341	regulation of oxidoreductase activity	4/180	95/28943	0.00296603459342577	0.0247701914635583	0.0173479000376617	Apoe/Npr3/Nus1/Agtr2	4	0.421052631578947
GO:0050708	BP	GO:0050708	regulation of protein secretion	7/180	305/28943	0.00309127646712215	0.0257500957887899	0.0180341798472303	Sfrp1/Cyp51/Vsnl1/Oxct1/Apoe/Rbp4/Inhbb	7	0.229508196721311
GO:1902903	BP	GO:1902903	regulation of supramolecular fiber organization	8/180	389/28943	0.00312723868367403	0.0259832050834855	0.0181974388494136	Slain1/S100a10/Sgk1/Tmsb10/Nrp1/Mapt/Apoe/Lpar1	8	0.205655526992288
GO:0001655	BP	GO:0001655	urogenital system development	8/180	391/28943	0.00322571480628735	0.0267332140561778	0.0187227105537021	Sfrp1/Mmp9/Nrp1/Bdnf/Rbp4/Anxa4/Agtr2/Efnb2	8	0.20460358056266
GO:0008360	BP	GO:0008360	regulation of cell shape	5/180	159/28943	0.00323595527000846	0.0267500160264405	0.0187344778789964	Sprr1b/Ccl2/Msn/Mark2/Lpar1	5	0.314465408805031
GO:0015908	BP	GO:0015908	fatty acid transport	4/180	98/28943	0.00331742578507762	0.02679801905867	0.0187680969895247	Slc27a2/Apoe/Agtr2/Pla2g4a	4	0.408163265306122
GO:0032091	BP	GO:0032091	negative regulation of protein binding	4/180	98/28943	0.00331742578507762	0.02679801905867	0.0187680969895247	Ctsz/B2m/Nes/Ckmt1	4	0.408163265306122

GO:0007613	BP	GO:0007613	memory	5/180	160/28943	0.0033238405 2661742	0.0267980190 5867	0.0187680969895247	Sgk1/Igf2/Bdnf/Mapt/Apoe	5	0.3125
GO:0050920	BP	GO:0050920	regulation of chemotaxis	6/180	231/28943	0.0033261903 9699498	0.0267980190 5867	0.0187680969895247	Ccl2/Scg2/Sema7a/Nrp1/Lpar1/Efnb2	6	0.25974025974026
GO:0043949	BP	GO:0043949	regulation of cAMP-mediated signaling	3/180	48/28943	0.0033313253 9311066	0.0267980190 5867	0.0187680969895247	Adcyap1/Nucb2/Lpar1	3	0.625
GO:0045197	BP	GO:0045197	establishment or maintenance of epithelial cell apical/basal polarity	3/180	48/28943	0.0033313253 9311066	0.0267980190 5867	0.0187680969895247	Lin7b/Msn/Mark2	3	0.625
GO:0048010	BP	GO:0048010	vascular endothelial growth factor receptor signaling pathway	3/180	48/28943	0.0033313253 9311066	0.0267980190 5867	0.0187680969895247	Ccl2/Fgf9/Nrp1	3	0.625
GO:0071385	BP	GO:0071385	cellular response to glucocorticoid stimulus	3/180	48/28943	0.0033313253 9311066	0.0267980190 5867	0.0187680969895247	Sgk1/Ddit4/Agtr2	3	0.625
GO:0071294	BP	GO:0071294	cellular response to zinc ion	2/180	14/28943	0.0033322682 5875387	0.0267980190 5867	0.0187680969895247	Mt2/Mt1	2	1.42857142857143
GO:1905809	BP	GO:1905809	negative regulation of synapse organization	2/180	14/28943	0.0033322682 5875387	0.0267980190 5867	0.0187680969895247	Apoe/Cbln1	2	1.42857142857143
GO:2000822	BP	GO:2000822	regulation of behavioral fear response	2/180	14/28943	0.0033322682 5875387	0.0267980190 5867	0.0187680969895247	Apoe/Penk	2	1.42857142857143
GO:0045667	BP	GO:0045667	regulation of osteoblast differentiation	5/180	161/28943	0.0034134312 0990251	0.0273158364 880896	0.0191307524424124	Cebpd/Sox11/Sfrp1/Lmna/Clic1	5	0.31055900621118
GO:2000377	BP	GO:2000377	regulation of reactive oxygen species metabolic process	5/180	161/28943	0.0034134312 0990251	0.0273158364 880896	0.0191307524424124	Gadd45a/Tyrobp/Abcb7/Itgb2/Mapt	5	0.31055900621118
GO:0019935	BP	GO:0019935	cyclic-nucleotide-mediated signaling	4/180	99/28943	0.0034405814 4620566	0.0273984688 760192	0.0191886243570394	Adcyap1/Nucb2/Apoe/Lpar1	4	0.404040404040404

GO:2001237	BP	GO:2001237	negative regulation of extrinsic apoptotic signaling pathway	4/180	99/28943	0.00344058144620566	0.0273984688760192	0.0191886243570394	Scg2/Lgals3/Lmna/Nrp1	4	0.404040404040404
GO:0060562	BP	GO:0060562	epithelial tube morphogenesis	8/180	397/28943	0.0035356791428693	0.0280069908889262	0.0196148051181433	Sox11/Adm/Sfrp1/Ctsz/Itgax/Nrp1/Agtr2/Efnb2	8	0.201511335012594
GO:0002685	BP	GO:0002685	regulation of leukocyte migration	6/180	234/28943	0.00354279405779478	0.0280069908889262	0.0196148051181433	Adam8/Ccl2/Hoxa7/Mmp9/Lgals3/Msn	6	0.256410256410256
GO:0097305	BP	GO:0097305	response to alcohol	6/180	234/28943	0.00354279405779478	0.0280069908889262	0.0196148051181433	Sgk1/Serpina1b/Inhbb/Aldh1a1/Ccr5/Serpina1c	6	0.256410256410256
GO:0007162	BP	GO:0007162	negative regulation of cell adhesion	7/180	313/28943	0.00356264413071502	0.0280957189678906	0.019676946173721	Mmp12/Adam8/Hoxa7/Lgals3/Enpp2/Laptm5/Lgals1	7	0.223642172523962
GO:0032535	BP	GO:0032535	regulation of cellular component size	8/180	398/28943	0.00358951122918176	0.0281683756605958	0.0197278315713559	Sema7a/Tmsb10/Nrp1/Bdnf/Mapt/Apoe/Pclo/Ccr5	8	0.201005025125628
GO:0051701	BP	GO:0051701	biological process involved in interaction with host	5/180	163/28943	0.00359780297046602	0.0281683756605958	0.0197278315713559	Nrp1/Ctsl/Gbp3/Lgals1/Trim25	5	0.306748466257669
GO:0097530	BP	GO:0097530	granulocyte migration	5/180	163/28943	0.00359780297046602	0.0281683756605958	0.0197278315713559	Adam8/Ccl2/Scg2/Lgals3/Itgb2	5	0.306748466257669
GO:0051056	BP	GO:0051056	regulation of small GTPase mediated signal transduction	6/180	236/28943	0.00369291341620947	0.0287999057527583	0.0201701261303516	Abca1/Arhgef3/Rit2/Nrp1/Apoe/Lpar1	6	0.254237288135593
GO:0031638	BP	GO:0031638	zymogen activation	4/180	101/28943	0.00369615001678016	0.0287999057527583	0.0201701261303516	Ctsz/S100a10/Clec3b/Ctsl	4	0.396039603960396
GO:0007157	BP	GO:0007157	heterophilic cell-cell adhesion via plasma membrane cell adhesion molecules	3/180	50/28943	0.003740988423645	0.0290104745138375	0.0203175987820519	Cbln1/Lgals1/Mcam	3	0.6
GO:0043618	BP	GO:0043618	regulation of transcription from RNA polymerase II promoter in response to stress	3/180	50/28943	0.003740988423645	0.0290104745138375	0.0203175987820519	Atf3/Jun/Atf6	3	0.6

GO:0007548	BP	GO:0007548	sex differentiation	7/180	317/28943	0.00381793297911346	0.0290719710352764	0.0203606681102868	Adcyap1/Sfrp1/Fgf9/Serpinb6a/Fndc3a/Rbp4/Pla2g4a	7	0.220820189274448
GO:0009410	BP	GO:0009410	response to xenobiotic stimulus	7/180	317/28943	0.00381793297911346	0.0290719710352764	0.0203606681102868	Jun/Sfrp1/Mmp9/Ugt1a1/Igf2/Bdnf/Aldh1a1	7	0.220820189274448
GO:0014033	BP	GO:0014033	neural crest cell differentiation	4/180	102/28943	0.00382863521344068	0.0290719710352764	0.0203606681102868	Sox11/Sfrp1/Sema7a/Nrp1	4	0.392156862745098
GO:0033700	BP	GO:0033700	phospholipid efflux	2/180	15/28943	0.00382925255576714	0.0290719710352764	0.0203606681102868	Abca1/Apoe	2	1.33333333333333
GO:0034638	BP	GO:0034638	phosphatidylcholine catabolic process	2/180	15/28943	0.00382925255576714	0.0290719710352764	0.0203606681102868	Enpp2/Pla2g4a	2	1.33333333333333
GO:0061029	BP	GO:0061029	eyelid development in camera-type eye	2/180	15/28943	0.00382925255576714	0.0290719710352764	0.0203606681102868	Jun/Sox11	2	1.33333333333333
GO:0110096	BP	GO:0110096	cellular response to aldehyde	2/180	15/28943	0.00382925255576714	0.0290719710352764	0.0203606681102868	Sgk1/Aldh1a1	2	1.33333333333333
GO:0150079	BP	GO:0150079	negative regulation of neuroinflammatory response	2/180	15/28943	0.00382925255576714	0.0290719710352764	0.0203606681102868	Cst7/Tnfrsf1b	2	1.33333333333333
GO:1903365	BP	GO:1903365	regulation of fear response	2/180	15/28943	0.00382925255576714	0.0290719710352764	0.0203606681102868	Apoe/Penk	2	1.33333333333333
GO:1903829	BP	GO:1903829	positive regulation of protein localization	9/180	493/28943	0.00386734377189942	0.0292928806164568	0.0205153829956084	Ccl2/Tyrobp/Lgals3/Msn/Vsnl1/Oxct1/Mapt/Rbp4/Ank3	9	0.182555780933063
GO:0030111	BP	GO:0030111	regulation of Wnt signaling pathway	7/180	319/28943	0.00395067739106851	0.0296821426358287	0.020788004166521	Sfrp1/Kpna1/Fgf9/Sfrp4/Apoe/Spin1/Mdfic	7	0.219435736677116
GO:0008038	BP	GO:0008038	neuron recognition	3/180	51/28943	0.00395681411627739	0.0296821426358287	0.020788004166521	Gap43/Nrp1/Bdnf	3	0.588235294117647

GO:1902041	BP	GO:1902041	regulation of extrinsic apoptotic signaling pathway via death domain receptors	3/180	51/28943	0.00395681411627739	0.0296821426358287	0.020788004166521	Atf3/Sfrp1/Lgals3	3	0.588235294117647
GO:0001938	BP	GO:0001938	positive regulation of endothelial cell proliferation	4/180	103/28943	0.00396430213281716	0.0296821426358287	0.020788004166521	Jun/Ccl2/Scg2/Igf2	4	0.388349514563107
GO:0010770	BP	GO:0010770	positive regulation of cell morphogenesis involved in differentiation	4/180	103/28943	0.00396430213281716	0.0296821426358287	0.020788004166521	S100a10/Enpp2/Sgk1/Nrp1	4	0.388349514563107
GO:0032675	BP	GO:0032675	regulation of interleukin-6 production	5/180	167/28943	0.0039878260067859	0.0297897919818846	0.0208633968051788	Adcyap1/Tyrobp/Laptm5/Ifih1/Ccr5	5	0.29940119760479
GO:0071241	BP	GO:0071241	cellular response to inorganic substance	6/180	241/28943	0.00408885736665513	0.0302354700610623	0.02117552918324	Jun/Mt2/Mmp9/B2m/Mt1/Ank3	6	0.24896265560166
GO:2001234	BP	GO:2001234	negative regulation of apoptotic signaling pathway	6/180	241/28943	0.00408885736665513	0.0302354700610623	0.02117552918324	Scg2/Mmp9/Lgals3/Lmna/Nrp1/Bdnf	6	0.24896265560166
GO:0006979	BP	GO:0006979	response to oxidative stress	8/180	407/28943	0.00410311002044984	0.0302354700610623	0.02117552918324	Jun/Mmp9/Cst3/Gpx3/Mapt/Apoe/Prdx6/Aldh1a1	8	0.196560196560197
GO:0030510	BP	GO:0030510	regulation of BMP signaling pathway	4/180	104/28943	0.00410318629628171	0.0302354700610623	0.02117552918324	Sox11/Sfrp1/Sfrp4/Htra1	4	0.384615384615385
GO:0046718	BP	GO:0046718	viral entry into host cell	4/180	104/28943	0.00410318629628171	0.0302354700610623	0.02117552918324	Nrp1/Ctsl/Lgals1/Trim25	4	0.384615384615385
GO:0055013	BP	GO:0055013	cardiac muscle cell development	4/180	104/28943	0.00410318629628171	0.0302354700610623	0.02117552918324	Fdps/Lmna/Col14a1/Agtr2	4	0.384615384615385
GO:0015849	BP	GO:0015849	organic acid transport	7/180	322/28943	0.00415633012059853	0.0304891153215978	0.0213531706290588	Slc27a2/Slc25a14/Bdnf/Apoe/Gfap/Agtr2/Pla2g4a	7	0.217391304347826
GO:1901653	BP	GO:1901653	cellular response to peptide	7/180	322/28943	0.00415633012059853	0.0304891153215978	0.0213531706290588	Sgk1/Car2/Igf2/Bglap/Nucb2/Inhbb/Pdk4	7	0.217391304347826

GO:0060251	BP	GO:0060251	regulation of glial cell proliferation	3/180	52/28943	0.00418007284827641	0.0305257786251934	0.0213788479164857	Adcyap1/Sox11/Gfap	3	0.576923076923077
GO:0071384	BP	GO:0071384	cellular response to corticosteroid stimulus	3/180	52/28943	0.00418007284827641	0.0305257786251934	0.0213788479164857	Sgk1/Ddit4/Agtr2	3	0.576923076923077
GO:0071383	BP	GO:0071383	cellular response to steroid hormone stimulus	5/180	169/28943	0.00419377254274949	0.030557309109027	0.0214009304136068	Sfrp1/Sgk1/Bdnf/Ddit4/Agtr2	5	0.29585798816568
GO:0048608	BP	GO:0048608	reproductive structure development	7/180	324/28943	0.00429787332984204	0.0309614310771985	0.0216839588075204	Adcyap1/Sfrp1/Fgf9/Serpinb6a/Fndc3a/Rbp4/Pla2g4a	7	0.216049382716049
GO:0045664	BP	GO:0045664	regulation of neuron differentiation	6/180	244/28943	0.00434101851648209	0.0309614310771985	0.0216839588075204	Sox11/Sfrp1/B2m/Nrep/Bdnf/Ccr5	6	0.245901639344262
GO:0001771	BP	GO:0001771	immunological synapse formation	2/180	16/28943	0.00435845925142279	0.0309614310771985	0.0216839588075204	Lgals3/Msn	2	1.25
GO:0034112	BP	GO:0034112	positive regulation of homotypic cell-cell adhesion	2/180	16/28943	0.00435845925142279	0.0309614310771985	0.0216839588075204	Ank3/Lgals1	2	1.25
GO:0042159	BP	GO:0042159	lipoprotein catabolic process	2/180	16/28943	0.00435845925142279	0.0309614310771985	0.0216839588075204	Ctsd/Apoe	2	1.25
GO:0043951	BP	GO:0043951	negative regulation of cAMP-mediated signaling	2/180	16/28943	0.00435845925142279	0.0309614310771985	0.0216839588075204	Nucb2/Lpar1	2	1.25
GO:0051151	BP	GO:0051151	negative regulation of smooth muscle cell differentiation	2/180	16/28943	0.00435845925142279	0.0309614310771985	0.0216839588075204	Ankrd17/Fgf9	2	1.25
GO:0060979	BP	GO:0060979	vasculogenesis involved in coronary vascular morphogenesis	2/180	16/28943	0.00435845925142279	0.0309614310771985	0.0216839588075204	Fgf9/Nrp1	2	1.25
GO:1902043	BP	GO:1902043	positive regulation of extrinsic apoptotic signaling pathway via death domain receptors	2/180	16/28943	0.00435845925142279	0.0309614310771985	0.0216839588075204	Atf3/Sfrp1	2	1.25

GO:0045921	BP	GO:0045921	positive regulation of exocytosis	4/180	106/28943	0.00439074714634315	0.0309614310771985	0.0216839588075204	S100a10/Itgb2/Vsnl1/Anxa2	4	0.377358490566038
GO:0051250	BP	GO:0051250	negative regulation of lymphocyte activation	5/180	171/28943	0.00440720528550109	0.0309614310771985	0.0216839588075204	Sox11/Sfrp1/Tyrobp/Lgals3/Laptm5	5	0.292397660818713
GO:0055067	BP	GO:0055067	monovalent inorganic cation homeostasis	5/180	171/28943	0.00440720528550109	0.0309614310771985	0.0216839588075204	Sgk1/Car2/Pdk4/Agtr2/Slc4a3	5	0.292397660818713
GO:0031103	BP	GO:0031103	axon regeneration	3/180	53/28943	0.00441083942886709	0.0309614310771985	0.0216839588075204	Jun/Gap43/Nrep	3	0.566037735849057
GO:0045124	BP	GO:0045124	regulation of bone resorption	3/180	53/28943	0.00441083942886709	0.0309614310771985	0.0216839588075204	Adam8/Car2/Pdk4	3	0.566037735849057
GO:0046676	BP	GO:0046676	negative regulation of insulin secretion	3/180	53/28943	0.00441083942886709	0.0309614310771985	0.0216839588075204	Sfrp1/Vsnl1/Inhbb	3	0.566037735849057
GO:0050819	BP	GO:0050819	negative regulation of coagulation	3/180	53/28943	0.00441083942886709	0.0309614310771985	0.0216839588075204	Procr/Apoe/Anxa2	3	0.566037735849057
GO:0099054	BP	GO:0099054	presynapse assembly	3/180	53/28943	0.00441083942886709	0.0309614310771985	0.0216839588075204	Cbln1/Pclo/Efnb2	3	0.566037735849057
GO:0031668	BP	GO:0031668	cellular response to extracellular stimulus	6/180	245/28943	0.00442757051974345	0.0310120369522675	0.0217194008291659	Atf3/Jun/Mmp9/Cd68/Inhbb/Pdk4	6	0.244897959183673
GO:0061458	BP	GO:0061458	reproductive system development	7/180	328/28943	0.0045918794562298	0.0320813561547663	0.0224683026961778	Adcyap1/Sfrp1/Fgf9/Serpinb6a/Fndc3a/Rbp4/Pla2g4a	7	0.213414634146341
GO:0035296	BP	GO:0035296	regulation of tube diameter	5/180	173/28943	0.00462827079838478	0.0320813561547663	0.0224683026961778	Adcyap1/Adm/Apoe/Npr3/Agtr2	5	0.289017341040462
GO:0097746	BP	GO:0097746	blood vessel diameter maintenance	5/180	173/28943	0.00462827079838478	0.0320813561547663	0.0224683026961778	Adcyap1/Adm/Apoe/Npr3/Agtr2	5	0.289017341040462

GO:0021545	BP	GO:0021545	cranial nerve development	3/180	54/28943	0.0046491864 0007667	0.0320813561 547663	0.0224683026961778	Adarb1/Nrp1/Egr2	3	0.555555555555556
GO:0035088	BP	GO:0035088	establishment or maintenance of apical/basal cell polarity	3/180	54/28943	0.0046491864 0007667	0.0320813561 547663	0.0224683026961778	Lin7b/Msn/Mark2	3	0.555555555555556
GO:0045687	BP	GO:0045687	positive regulation of glial cell differentiation	3/180	54/28943	0.0046491864 0007667	0.0320813561 547663	0.0224683026961778	Tnfrsf1b/Enpp2/Egr2	3	0.555555555555556
GO:0061245	BP	GO:0061245	establishment or maintenance of bipolar cell polarity	3/180	54/28943	0.0046491864 0007667	0.0320813561 547663	0.0224683026961778	Lin7b/Msn/Mark2	3	0.555555555555556
GO:0044409	BP	GO:0044409	entry into host	4/180	108/28943	0.0046915966 8962499	0.0323055611 376503	0.0226253255289137	Nrp1/Ctsl/Lgals1/Trim25	4	0.37037037037037
GO:0001936	BP	GO:0001936	regulation of endothelial cell proliferation	5/180	174/28943	0.0047417114 97678	0.0325131670 482889	0.0227707231367717	Jun/Ccl2/Scg2/Igf2/Apoe	5	0.28735632183908
GO:0035150	BP	GO:0035150	regulation of tube size	5/180	174/28943	0.0047417114 97678	0.0325131670 482889	0.0227707231367717	Adcyap1/Adm/Apoe/Npr3/Agtr2	5	0.28735632183908
GO:0033555	BP	GO:0033555	multicellular organismal response to stress	4/180	109/28943	0.0048470906 9973038	0.0330954474 545807	0.0231785254865072	Adcyap1/Bdnf/Apoe/Penk	4	0.36697247706422
GO:0034446	BP	GO:0034446	substrate adhesion-dependent cell spreading	4/180	109/28943	0.0048470906 9973038	0.0330954474 545807	0.0231785254865072	Sfrp1/S100a10/Enpp2/Nrp1	4	0.36697247706422
GO:0050792	BP	GO:0050792	regulation of viral process	5/180	175/28943	0.0048571151 0079508	0.0330954474 545807	0.0231785254865072	Adarb1/Ifih1/Lgals1/Mdfic/Trim25	5	0.285714285714286
GO:0031113	BP	GO:0031113	regulation of microtubule polymerization	3/180	55/28943	0.0048951840 6808576	0.0332158635 619902	0.0232628593761883	Slain1/Sgk1/Mapt	3	0.545454545454545
GO:0043620	BP	GO:0043620	regulation of DNA-templated transcription in response to stress	3/180	55/28943	0.0048951840 6808576	0.0332158635 619902	0.0232628593761883	Atf3/Jun/Atf6	3	0.545454545454545

GO:0045655	BP	GO:0045655	regulation of monocyte differentiation	2/180	17/28943	0.00491947286660229	0.0333112746913174	0.0233296809321089	Jun/Hoxa7	2	1.17647058823529
GO:0010594	BP	GO:0010594	regulation of endothelial cell migration	5/180	176/28943	0.00497449976475469	0.0335444011051885	0.0234929519237734	Gadd45a/Igf2/Nrp1/Apoe/Nus1	5	0.284090909090909
GO:0032635	BP	GO:0032635	interleukin-6 production	5/180	176/28943	0.00497449976475469	0.0335444011051885	0.0234929519237734	Adcyap1/Tyrobp/Laptm5/Ifih1/Ccr5	5	0.284090909090909
GO:0046889	BP	GO:0046889	positive regulation of lipid biosynthetic process	4/180	110/28943	0.00500600950111739	0.033617676175545	0.0235442704046042	Fdps/Igf2/Apoe/Pla2g4a	4	0.363636363636364
GO:0055006	BP	GO:0055006	cardiac cell development	4/180	110/28943	0.00500600950111739	0.033617676175545	0.0235442704046042	Fdps/Lmna/Col14a1/Agtr2	4	0.363636363636364
GO:0001822	BP	GO:0001822	kidney development	7/180	334/28943	0.00506106083018425	0.033917438526564	0.0237542101343064	Sfrp1/Mmp9/Nrp1/Bdnf/Anxa4/Agtr2/Efnb2	7	0.209580838323353
GO:0007596	BP	GO:0007596	blood coagulation	5/180	177/28943	0.0050938836047046	0.0340673078039484	0.0238591716662593	Procr/Papss2/Apoe/Anxa2/Pla2g4a	5	0.282485875706215
GO:0032760	BP	GO:0032760	positive regulation of tumor necrosis factor production	4/180	111/28943	0.0051683867129921	0.0344947449266706	0.0241585289194432	Ccl2/Tyrobp/Ifih1/Ccr5	4	0.36036036036036
GO:0043254	BP	GO:0043254	regulation of protein-containing complex assembly	8/180	425/28943	0.00529900490445613	0.0352941901304982	0.0247184234806941	Abca1/Lgals3/Msn/Slain1/Sgk1/Tmsb10/Mapt/Apoe	8	0.188235294117647
GO:0002762	BP	GO:0002762	negative regulation of myeloid leukocyte differentiation	3/180	57/28943	0.00541040172575095	0.0356194424637819	0.0249462152187241	Hoxa7/Sfrp1/C1qc	3	0.526315789473684
GO:0034113	BP	GO:0034113	heterotypic cell-cell adhesion	3/180	57/28943	0.00541040172575095	0.0356194424637819	0.0249462152187241	Dsp/Itgax/Itgb2	3	0.526315789473684
GO:0043536	BP	GO:0043536	positive regulation of blood vessel endothelial cell migration	3/180	57/28943	0.00541040172575095	0.0356194424637819	0.0249462152187241	Igf2/Nrp1/Nus1	3	0.526315789473684

GO:0001890	BP	GO:0001890	placenta development	5/180	180/28943	0.00546421066364658	0.0356194424637819	0.0249462152187241	Adm/Igf2/Htra1/Ctsl/Pla2g4a	5	0.277777777777778
GO:0007599	BP	GO:0007599	hemostasis	5/180	180/28943	0.00546421066364658	0.0356194424637819	0.0249462152187241	Procr/Papss2/Apoe/Anxa2/Pla2g4a	5	0.277777777777778
GO:0050817	BP	GO:0050817	coagulation	5/180	180/28943	0.00546421066364658	0.0356194424637819	0.0249462152187241	Procr/Papss2/Apoe/Anxa2/Pla2g4a	5	0.277777777777778
GO:1902905	BP	GO:1902905	positive regulation of supramolecular fiber organization	5/180	180/28943	0.00546421066364658	0.0356194424637819	0.0249462152187241	Slain1/S100a10/Nrp1/Mapt/Lpar1	5	0.277777777777778
GO:0030004	BP	GO:0030004	cellular monovalent inorganic cation homeostasis	4/180	113/28943	0.00550364939676854	0.0356194424637819	0.0249462152187241	Sgk1/Car2/Agtr2/Slc4a3	4	0.353982300884956
GO:0002862	BP	GO:0002862	negative regulation of inflammatory response to antigenic stimulus	2/180	18/28943	0.00551188179359719	0.0356194424637819	0.0249462152187241	Adcyap1/Npy	2	1.11111111111111
GO:0060712	BP	GO:0060712	spongiotrophoblast layer development	2/180	18/28943	0.00551188179359719	0.0356194424637819	0.0249462152187241	Adm/Igf2	2	1.11111111111111
GO:0070486	BP	GO:0070486	leukocyte aggregation	2/180	18/28943	0.00551188179359719	0.0356194424637819	0.0249462152187241	Adam8/Msn	2	1.11111111111111
GO:0071280	BP	GO:0071280	cellular response to copper ion	2/180	18/28943	0.00551188179359719	0.0356194424637819	0.0249462152187241	Mt2/Mt1	2	1.11111111111111
GO:0099150	BP	GO:0099150	regulation of postsynaptic specialization assembly	2/180	18/28943	0.00551188179359719	0.0356194424637819	0.0249462152187241	Gap43/Cbln1	2	1.11111111111111
GO:1900221	BP	GO:1900221	regulation of amyloid-beta clearance	2/180	18/28943	0.00551188179359719	0.0356194424637819	0.0249462152187241	Cyp51/Apoe	2	1.11111111111111
GO:1903729	BP	GO:1903729	regulation of plasma membrane organization	2/180	18/28943	0.00551188179359719	0.0356194424637819	0.0249462152187241	S100a10/Anxa2	2	1.11111111111111

GO:0051235	BP	GO:0051235	maintenance of location	7/180	341/28943	0.00565305401853956	0.036343713938165	0.0254534615658593	Msr1/Sqle/Rit2/Tmsb10/Apoe/Ank3/Ccr5	7	0.205278592375367
GO:0009395	BP	GO:0009395	phospholipid catabolic process	3/180	58/28943	0.00567975142601351	0.036343713938165	0.0254534615658593	Enpp2/Prdx6/Pla2g4a	3	0.517241379310345
GO:0033619	BP	GO:0033619	membrane protein proteolysis	3/180	58/28943	0.00567975142601351	0.036343713938165	0.0254534615658593	Adam8/Tnfrsf1b/Apoe	3	0.517241379310345
GO:0051496	BP	GO:0051496	positive regulation of stress fiber assembly	3/180	58/28943	0.00567975142601351	0.036343713938165	0.0254534615658593	S100a10/Nrp1/Lpar1	3	0.517241379310345
GO:0140353	BP	GO:0140353	lipid export from cell	3/180	58/28943	0.00567975142601351	0.036343713938165	0.0254534615658593	Nucb2/Agtr2/Pla2g4a	3	0.517241379310345
GO:0007265	BP	GO:0007265	Ras protein signal transduction	7/180	344/28943	0.00592206221874742	0.0378199149930595	0.0264873247224472	Jun/Abca1/Arhgef3/Rit2/Nrp1/Apoe/Lpar1	7	0.203488372093023
GO:1900024	BP	GO:1900024	regulation of substrate adhesion-dependent cell spreading	3/180	59/28943	0.00595701130466898	0.0378945035533337	0.0265395631110561	S100a10/Enpp2/Nrp1	3	0.508474576271186
GO:2001238	BP	GO:2001238	positive regulation of extrinsic apoptotic signaling pathway	3/180	59/28943	0.00595701130466898	0.0378945035533337	0.0265395631110561	Atf3/Sfrp1/Agtr2	3	0.508474576271186
GO:0007264	BP	GO:0007264	small GTPase mediated signal transduction	8/180	434/28943	0.00598883772155136	0.0379704007481284	0.0265927180069467	Jun/Abca1/Arhgef3/Rit2/Nrp1/Nucb2/Apoe/Lpar1	8	0.184331797235023
GO:0045926	BP	GO:0045926	negative regulation of growth	6/180	261/28943	0.00599225851536322	0.0379704007481284	0.0265927180069467	Sfrp1/H19/Sema7a/Nrp1/Rbp4/Ccr5	6	0.229885057471264
GO:0060840	BP	GO:0060840	artery development	4/180	116/28943	0.00603330516595621	0.0381562619912997	0.0267228866522782	Nrp1/Apoe/Egr2/Efnb2	4	0.344827586206897
GO:0048660	BP	GO:0048660	regulation of smooth muscle cell proliferation	5/180	185/28943	0.00612308315350107	0.0384280794372398	0.026913255058901	Jun/Mmp9/Fgf9/Apoe/Npr3	5	0.27027027027027

GO:0016114	BP	GO:0016114	terpenoid biosynthetic process	2/180	19/28943	0.00613527826446567	0.0384280794372398	0.026913255058901	Fdps/Aldh1a1	2	1.05263157894737
GO:0035641	BP	GO:0035641	locomotory exploration behavior	2/180	19/28943	0.00613527826446567	0.0384280794372398	0.026913255058901	Apoe/Penk	2	1.05263157894737
GO:0043691	BP	GO:0043691	reverse cholesterol transport	2/180	19/28943	0.00613527826446567	0.0384280794372398	0.026913255058901	Abca1/Apoe	2	1.05263157894737
GO:1900452	BP	GO:1900452	regulation of long-term synaptic depression	2/180	19/28943	0.00613527826446567	0.0384280794372398	0.026913255058901	Mapt/Cbln1	2	1.05263157894737
GO:0006913	BP	GO:0006913	nucleocytoplasmic transport	7/180	347/28943	0.00620053657050292	0.0385056416009979	0.026967576022288	Mmp12/Kpna1/Fgf9/Lmna/Mdfic/Egr2/Agtr2	7	0.201729106628242
GO:0051169	BP	GO:0051169	nuclear transport	7/180	347/28943	0.00620053657050292	0.0385056416009979	0.026967576022288	Mmp12/Kpna1/Fgf9/Lmna/Mdfic/Egr2/Agtr2	7	0.201729106628242
GO:0044070	BP	GO:0044070	regulation of anion transport	4/180	117/28943	0.00621712240120178	0.0385056416009979	0.026967576022288	Car2/Sfrp4/Agtr2/Pla2g4a	4	0.341880341880342
GO:0060078	BP	GO:0060078	regulation of postsynaptic membrane potential	4/180	117/28943	0.00621712240120178	0.0385056416009979	0.026967576022288	Adcyap1/Bdnf/Cbln1/Pclo	4	0.341880341880342
GO:0030199	BP	GO:0030199	collagen fibril organization	3/180	60/28943	0.00624224094729103	0.0385056416009979	0.026967576022288	Acan/Col14a1/Anxa2	3	0.5
GO:0035924	BP	GO:0035924	cellular response to vascular endothelial growth factor stimulus	3/180	60/28943	0.00624224094729103	0.0385056416009979	0.026967576022288	Nrp1/Cd63/Nus1	3	0.5
GO:0050922	BP	GO:0050922	negative regulation of chemotaxis	3/180	60/28943	0.00624224094729103	0.0385056416009979	0.026967576022288	Ccl2/Sema7a/Nrp1	3	0.5
GO:0099172	BP	GO:0099172	presynapse organization	3/180	60/28943	0.00624224094729103	0.0385056416009979	0.026967576022288	Cbln1/Pclo/Efnb2	3	0.5

GO:0035051	BP	GO:0035051	cardiocyte differentiation	5/180	186/28943	0.0062612669 1240067	0.0385499930 693553	0.0269986377458407	Fdps/Lmna/Col14a1/Agtr2/Efnb2	5	0.268817204301075
GO:0043271	BP	GO:0043271	negative regulation of ion transport	5/180	187/28943	0.0064016282 8158045	0.0391919235 208788	0.02744821623701	Adcyap1/Mmp9/Sfrp4/Ank3/Agtr2	5	0.267379679144385
GO:0071772	BP	GO:0071772	response to BMP	5/180	187/28943	0.0064016282 8158045	0.0391919235 208788	0.02744821623701	Sox11/Sfrp1/Sfrp4/Bglap/Htra1	5	0.267379679144385
GO:0071773	BP	GO:0071773	cellular response to BMP stimulus	5/180	187/28943	0.0064016282 8158045	0.0391919235 208788	0.02744821623701	Sox11/Sfrp1/Sfrp4/Bglap/Htra1	5	0.267379679144385
GO:0045444	BP	GO:0045444	fat cell differentiation	6/180	265/28943	0.0064394086 5839437	0.0393492570 363799	0.0275584053771909	Cebpd/Sfrp1/Selenbp1/Nucb2/Inhbb/E gr2	6	0.226415094339623
GO:0048524	BP	GO:0048524	positive regulation of viral process	3/180	61/28943	0.0065354978 8477274	0.0397656900 550939	0.0278500558632867	Adarb1/Lgals1/Mdfic	3	0.491803278688525
GO:0060760	BP	GO:0060760	positive regulation of response to cytokine stimulus	3/180	61/28943	0.0065354978 8477274	0.0397656900 550939	0.0278500558632867	Mmp12/Laptm5/Ifih1	3	0.491803278688525
GO:0045834	BP	GO:0045834	positive regulation of lipid metabolic process	5/180	188/28943	0.0065441847 9260986	0.0397656900 550939	0.0278500558632867	Fdps/Igf2/Nucb2/Apoe/Pla2g4a	5	0.265957446808511
GO:0001659	BP	GO:0001659	temperature homeostasis	5/180	189/28943	0.0066889539 1329613	0.0404942804 751032	0.0283603270008075	Adcyap1/Elovl6/Npr3/Aldh1a1/Ccr5	5	0.264550264550265
GO:1903555	BP	GO:1903555	regulation of tumor necrosis factor superfamily cytokine production	5/180	189/28943	0.0066889539 1329613	0.0404942804 751032	0.0283603270008075	Adam8/Ccl2/Tyrobp/Ifih1/Ccr5	5	0.264550264550265
GO:0009083	BP	GO:0009083	branched-chain amino acid catabolic process	2/180	20/28943	0.0067892583 1962963	0.0406574860 010887	0.0284746286263947	Bckdhb/Hibadh	2	1
GO:0019886	BP	GO:0019886	antigen processing and presentation of exogenous peptide antigen via MHC class II	2/180	20/28943	0.0067892583 1962963	0.0406574860 010887	0.0284746286263947	Ctss/B2m	2	1

GO:0043116	BP	GO:0043116	negative regulation of vascular permeability	2/180	20/28943	0.00678925831962963	0.0406574860010887	0.0284746286263947	Adm/Apoe	2	1
GO:0045540	BP	GO:0045540	regulation of cholesterol biosynthetic process	2/180	20/28943	0.00678925831962963	0.0406574860010887	0.0284746286263947	Fdps/Apoe	2	1
GO:0106118	BP	GO:0106118	regulation of sterol biosynthetic process	2/180	20/28943	0.00678925831962963	0.0406574860010887	0.0284746286263947	Fdps/Apoe	2	1
GO:0071675	BP	GO:0071675	regulation of mononuclear cell migration	4/180	120/28943	0.00679081129401051	0.0406574860010887	0.0284746286263947	Adam8/Ccl2/Lgals3/Msn	4	0.333333333333333
GO:0046622	BP	GO:0046622	positive regulation of organ growth	3/180	62/28943	0.00683683762239313	0.0408579452039164	0.0286150210096106	Fgf9/Fdps/Igf2	3	0.483870967741935
GO:0030099	BP	GO:0030099	myeloid cell differentiation	8/180	445/28943	0.00692249851459438	0.0412940982821133	0.0289204825161481	Jun/Adam8/Hoxa7/Sfrp1/C1qc/Tyrobp/B2m/Car2	8	0.179775280898876
GO:0001935	BP	GO:0001935	endothelial cell proliferation	5/180	191/28943	0.00698519952584684	0.0413239086476885	0.0289413603217494	Jun/Ccl2/Scg2/Igf2/Apoe	5	0.261780104712042
GO:0050796	BP	GO:0050796	regulation of insulin secretion	5/180	191/28943	0.00698519952584684	0.0413239086476885	0.0289413603217494	Sfrp1/Vsnl1/Oxct1/Rbp4/Inhbb	5	0.261780104712042
GO:0010769	BP	GO:0010769	regulation of cell morphogenesis involved in differentiation	4/180	121/28943	0.00698955628149154	0.0413239086476885	0.0289413603217494	S100a10/Enpp2/Sgk1/Nrp1	4	0.330578512396694
GO:0009100	BP	GO:0009100	glycoprotein metabolic process	7/180	355/28943	0.0069909344995015	0.0413239086476885	0.0289413603217494	Mmp12/St3gal5/Acan/Serpina1b/Ctsl/Serpina1a/Nus1	7	0.197183098591549
GO:0042176	BP	GO:0042176	regulation of protein catabolic process	7/180	355/28943	0.0069909344995015	0.0413239086476885	0.0289413603217494	Adam8/Cyp51/Msn/Tnfrsf1b/Apoe/Anxa2/Gfap	7	0.197183098591549
GO:0048659	BP	GO:0048659	smooth muscle cell proliferation	5/180	192/28943	0.00713671061926484	0.0420895906302529	0.0294776086795269	Jun/Mmp9/Fgf9/Apoe/Npr3	5	0.260416666666667

GO:0006487	BP	GO:0006487	protein N-linked glycosylation	3/180	63/28943	0.0071463136 6856919	0.0420895906 302529	0.0294776086795269	Serpina1b/Serpina1a/Nus1	3	0.476190476190476
GO:0009636	BP	GO:0009636	response to toxic substance	4/180	122/28943	0.0071921096 271224	0.0421753312 565296	0.0295376574609827	Mt2/Mt1/Prdx6/Aldh1a1	4	0.327868852459016
GO:0002253	BP	GO:0002253	activation of immune response	8/180	448/28943	0.0071952759 7424342	0.0421753312 565296	0.0295376574609827	C1qa/C4a/C1qc/Mog/Tyrobp/Lgals3/ Lat2/Laptm5	8	0.178571428571429
GO:0045862	BP	GO:0045862	positive regulation of proteolysis	7/180	357/28943	0.0071997188 144398	0.0421753312 565296	0.0295376574609827	Adam8/Tnfrsf1b/S100a10/Clec3b/Cts d/Laptm5/Apoe	7	0.196078431372549
GO:0030316	BP	GO:0030316	osteoclast differentiation	4/180	123/28943	0.0073985012 5258069	0.0430967545 923947	0.0301829798819447	Adam8/Sfrp1/Tyrobp/Car2	4	0.32520325203252
GO:0050777	BP	GO:0050777	negative regulation of immune response	5/180	194/28943	0.0074465953 582454	0.0430967545 923947	0.0301829798819447	Adcyap1/Mmp12/Npy/Lgals3/Igf2	5	0.257731958762887
GO:0001954	BP	GO:0001954	positive regulation of cell-matrix adhesion	3/180	64/28943	0.0074639775 6329675	0.0430967545 923947	0.0301829798819447	S100a10/Enpp2/Nrp1	3	0.46875
GO:0051893	BP	GO:0051893	regulation of focal adhesion assembly	3/180	64/28943	0.0074639775 6329675	0.0430967545 923947	0.0301829798819447	S100a10/Enpp2/Nrp1	3	0.46875
GO:0090109	BP	GO:0090109	regulation of cell-substrate junction assembly	3/180	64/28943	0.0074639775 6329675	0.0430967545 923947	0.0301829798819447	S100a10/Enpp2/Nrp1	3	0.46875
GO:0046697	BP	GO:0046697	decidualization	2/180	21/28943	0.0074734217 7671033	0.0430967545 923947	0.0301829798819447	Ctsl/Pla2g4a	2	0.952380952380952
GO:0090280	BP	GO:0090280	positive regulation of calcium ion import	2/180	21/28943	0.0074734217 7671033	0.0430967545 923947	0.0301829798819447	Ccl2/Lgals3	2	0.952380952380952
GO:0150146	BP	GO:0150146	cell junction disassembly	2/180	21/28943	0.0074734217 7671033	0.0430967545 923947	0.0301829798819447	C1qa/C1qc	2	0.952380952380952

GO:0016055	BP	GO:0016055	Wnt signaling pathway	8/180	451/28943	0.00747610265419189	0.0430967545923947	0.0301829798819447	Sfrp1/Kpna1/Fgf9/Sfrp4/Mark2/Apoe/Spin1/Mdfic	8	0.177383592017738
GO:1901654	BP	GO:1901654	response to ketone	5/180	195/28943	0.00760500317769807	0.043762359275199	0.0306491387131704	Msn/Sgk1/Bglap/Ddit4/Agtr2	5	0.256410256410256
GO:0198738	BP	GO:0198738	cell-cell signaling by wnt	8/180	453/28943	0.00766786402789294	0.0440462665588136	0.0308479742846821	Sfrp1/Kpna1/Fgf9/Sfrp4/Mark2/Apoe/Spin1/Mdfic	8	0.176600441501104
GO:0042129	BP	GO:0042129	regulation of T cell proliferation	5/180	196/28943	0.00776574395527973	0.0444336875617576	0.0311193060925711	Igfbp2/Lgals3/Tnfrsf1b/Laptm5/Igf2	5	0.255102040816327
GO:0071706	BP	GO:0071706	tumor necrosis factor superfamily cytokine production	5/180	196/28943	0.00776574395527973	0.0444336875617576	0.0311193060925711	Adam8/Ccl2/Tyrobp/Ifih1/Ccr5	5	0.255102040816327
GO:0001836	BP	GO:0001836	release of cytochrome c from mitochondria	3/180	65/28943	0.00778987890628296	0.0444336875617576	0.0311193060925711	Jun/Mmp9/Lmna	3	0.461538461538462
GO:2000401	BP	GO:2000401	regulation of lymphocyte migration	3/180	65/28943	0.00778987890628296	0.0444336875617576	0.0311193060925711	Adam8/Ccl2/Msn	3	0.461538461538462
GO:0001910	BP	GO:0001910	regulation of leukocyte mediated cytotoxicity	4/180	125/28943	0.00782291738758487	0.0444663908051726	0.0311422099364314	Ccl2/Tyrobp/B2m/Igf2	4	0.32
GO:0090277	BP	GO:0090277	positive regulation of peptide hormone secretion	4/180	125/28943	0.00782291738758487	0.0444663908051726	0.0311422099364314	Adcyap1/Vsnl1/Oxct1/Rbp4	4	0.32
GO:0002718	BP	GO:0002718	regulation of cytokine production involved in immune response	4/180	126/28943	0.00804100009086892	0.0456263715957492	0.031954606999657	Tnfrsf1b/B2m/Sema7a/Laptm5	4	0.317460317460317
GO:0015909	BP	GO:0015909	long-chain fatty acid transport	3/180	66/28943	0.00812406538477324	0.0459376405524417	0.0321726054254452	Slc27a2/Apoe/Pla2g4a	3	0.454545454545455
GO:0072678	BP	GO:0072678	T cell migration	3/180	66/28943	0.00812406538477324	0.0459376405524417	0.0321726054254452	Adam8/Ccl2/Msn	3	0.454545454545455

GO:0002283	BP	GO:0002283	neutrophil activation involved in immune response	2/180	22/28943	0.00818737219960101	0.0459763297484491	0.0321997015545107	Tyrobp/Itgb2	2	0.909090909090909
GO:0034377	BP	GO:0034377	plasma lipoprotein particle assembly	2/180	22/28943	0.00818737219960101	0.0459763297484491	0.0321997015545107	Abca1/Apoe	2	0.909090909090909
GO:0048532	BP	GO:0048532	anatomical structure arrangement	2/180	22/28943	0.00818737219960101	0.0459763297484491	0.0321997015545107	Nrp1/Egr2	2	0.909090909090909
GO:0060253	BP	GO:0060253	negative regulation of glial cell proliferation	2/180	22/28943	0.00818737219960101	0.0459763297484491	0.0321997015545107	Adcyap1/Sox11	2	0.909090909090909
GO:0003205	BP	GO:0003205	cardiac chamber development	5/180	199/28943	0.00826213262231389	0.0462417749079446	0.0323856071055107	Dsp/Sox11/Fgf9/Nrp1/Rbp4	5	0.251256281407035
GO:0044000	BP	GO:0044000	movement in host	4/180	127/28943	0.00826303745668522	0.0462417749079446	0.0323856071055107	Nrp1/Ctsl/Lgals1/Trim25	4	0.31496062992126
GO:0051604	BP	GO:0051604	protein maturation	7/180	367/28943	0.00831371490598046	0.0464455736685735	0.0325283383610358	Cst7/Ctss/Ctsz/S100a10/Clec3b/Ctla2a/Ctsl	7	0.190735694822888
GO:0019882	BP	GO:0019882	antigen processing and presentation	4/180	128/28943	0.00848905774277638	0.0473439401853128	0.0331575128489481	Ctss/Cd68/B2m/Ctsl	4	0.3125
GO:0002793	BP	GO:0002793	positive regulation of peptide secretion	4/180	129/28943	0.00871908886728532	0.0484292013490774	0.0339175797306048	Adcyap1/Vsnl1/Oxct1/Rbp4	4	0.310077519379845
GO:0071621	BP	GO:0071621	granulocyte chemotaxis	4/180	129/28943	0.00871908886728532	0.0484292013490774	0.0339175797306048	Ccl2/Scg2/Lgals3/Itgb2	4	0.310077519379845
GO:0003007	BP	GO:0003007	heart morphogenesis	6/180	283/28943	0.0087560858964818	0.0484292013490774	0.0339175797306048	Dsp/Jun/Sox11/Fgf9/Nrp1/Rbp4	6	0.212014134275618
GO:0002831	BP	GO:0002831	regulation of response to biotic stimulus	7/180	371/28943	0.00879311997552455	0.0484292013490774	0.0339175797306048	Mmp12/Adam8/Tyrobp/Ankrd17/Igf2/Apoe/Htra1	7	0.188679245283019

GO:0006801	BP	GO:0006801	superoxide metabolic process	3/180	68/28943	0.0088174750 9978596	0.0484292013 490774	0.0339175797306048	Tyrobp/Itgb2/Mapt	3	0.441176470588235
GO:0007584	BP	GO:0007584	response to nutrient	3/180	68/28943	0.0088174750 9978596	0.0484292013 490774	0.0339175797306048	Bckdhb/Bglap/Mapt	3	0.441176470588235
GO:0019933	BP	GO:0019933	cAMP-mediated signaling	3/180	68/28943	0.0088174750 9978596	0.0484292013 490774	0.0339175797306048	Adcyap1/Nucb2/Lpar1	3	0.441176470588235
GO:0032233	BP	GO:0032233	positive regulation of actin filament bundle assembly	3/180	68/28943	0.0088174750 9978596	0.0484292013 490774	0.0339175797306048	S100a10/Nrp1/Lpar1	3	0.441176470588235
GO:0050771	BP	GO:0050771	negative regulation of axonogenesis	3/180	68/28943	0.0088174750 9978596	0.0484292013 490774	0.0339175797306048	Sema7a/Nrp1/Ccr5	3	0.441176470588235
GO:0051251	BP	GO:0051251	positive regulation of lymphocyte activation	8/180	465/28943	0.0088973328 593755	0.0484789080 639032	0.033952392021873	Adam8/Ccl2/Igfbp2/Tyrobp/B2m/Igf2 /Lgals1/Efnb2	8	0.172043010752688
GO:0002693	BP	GO:0002693	positive regulation of cellular extravasation	2/180	23/28943	0.0089307168 6777462	0.0484789080 639032	0.033952392021873	Adam8/Ccl2	2	0.869565217391304
GO:0002864	BP	GO:0002864	regulation of acute inflammatory response to antigenic stimulus	2/180	23/28943	0.0089307168 6777462	0.0484789080 639032	0.033952392021873	Adcyap1/Npy	2	0.869565217391304
GO:0019835	BP	GO:0019835	cytolysis	2/180	23/28943	0.0089307168 6777462	0.0484789080 639032	0.033952392021873	Lyz1/Lyz2	2	0.869565217391304
GO:0030728	BP	GO:0030728	ovulation	2/180	23/28943	0.0089307168 6777462	0.0484789080 639032	0.033952392021873	Inhbb/Pla2g4a	2	0.869565217391304
GO:0032095	BP	GO:0032095	regulation of response to food	2/180	23/28943	0.0089307168 6777462	0.0484789080 639032	0.033952392021873	Npy/Nucb2	2	0.869565217391304
GO:0140354	BP	GO:0140354	lipid import into cell	2/180	23/28943	0.0089307168 6777462	0.0484789080 639032	0.033952392021873	Slc27a2/Rbp4	2	0.869565217391304

GO:0031334	BP	GO:0031334	positive regulation of protein-containing complex assembly	5/180	203/28943	0.00895766157771783	0.0485442658213427	0.0339981655817752	Abca1/Lgals3/Msn/Slain1/Mapt	5	0.246305418719212
GO:0150116	BP	GO:0150116	regulation of cell-substrate junction organization	3/180	69/28943	0.0091767843947146	0.0496491474644276	0.0347719737425188	S100a10/Enpp2/Nrp1	3	0.434782608695652
GO:0046890	BP	GO:0046890	regulation of lipid biosynthetic process	5/180	205/28943	0.00932015564533395	0.0503412055337524	0.035256659305994	Fdps/Igf2/Apoe/Pdk4/Pla2g4a	5	0.24390243902439
GO:0061351	BP	GO:0061351	neural precursor cell proliferation	5/180	206/28943	0.00950514195202759	0.0511292805796257	0.0358085907328503	Adcyap1/Ctsz/Nes/Bdnf/Ccr5	5	0.242718446601942
GO:0000768	BP	GO:0000768	syncytium formation by plasma membrane fusion	3/180	70/28943	0.00954455099552115	0.0511292805796257	0.0358085907328503	Adam8/Tyrobp/Cd53	3	0.428571428571429
GO:0032507	BP	GO:0032507	maintenance of protein location in cell	3/180	70/28943	0.00954455099552115	0.0511292805796257	0.0358085907328503	Rit2/Tmsb10/Ank3	3	0.428571428571429
GO:0071677	BP	GO:0071677	positive regulation of mononuclear cell migration	3/180	70/28943	0.00954455099552115	0.0511292805796257	0.0358085907328503	Adam8/Ccl2/Lgals3	3	0.428571428571429
GO:0140253	BP	GO:0140253	cell-cell fusion	3/180	70/28943	0.00954455099552115	0.0511292805796257	0.0358085907328503	Adam8/Tyrobp/Cd53	3	0.428571428571429
GO:1990266	BP	GO:1990266	neutrophil migration	4/180	133/28943	0.00967986811450719	0.0513868103921177	0.035988952739757	Adam8/Ccl2/Lgals3/Itgb2	4	0.300751879699248
GO:0048167	BP	GO:0048167	regulation of synaptic plasticity	7/180	378/28943	0.009680550383285	0.0513868103921177	0.035988952739757	Mmp9/Tyrobp/Bdnf/Mapt/Apoe/Cbln1/Gfap	7	0.185185185185185
GO:0002700	BP	GO:0002700	regulation of production of molecular mediator of immune response	5/180	207/28943	0.00969264253372971	0.0513868103921177	0.035988952739757	Tnfrsf1b/B2m/Sema7a/Laptm5/Rbp4	5	0.241545893719807
GO:0007519	BP	GO:0007519	skeletal muscle tissue development	5/180	207/28943	0.00969264253372971	0.0513868103921177	0.035988952739757	Atf3/Sox11/Nr1d2/Igf2/Egr2	5	0.241545893719807

GO:0002495	BP	GO:0002495	antigen processing and presentation of peptide antigen via MHC class II	2/180	24/28943	0.00970306674582511	0.0513868103921177	0.035988952739757	Ctss/B2m	2	0.833333333333333
GO:0009081	BP	GO:0009081	branched-chain amino acid metabolic process	2/180	24/28943	0.00970306674582511	0.0513868103921177	0.035988952739757	Bckdhb/Hibadh	2	0.833333333333333
GO:0048485	BP	GO:0048485	sympathetic nervous system development	2/180	24/28943	0.00970306674582511	0.0513868103921177	0.035988952739757	Sox11/Nrp1	2	0.833333333333333
GO:0014009	BP	GO:0014009	glial cell proliferation	3/180	71/28943	0.00992081343405632	0.052369674805059	0.0366773056582787	Adcyap1/Sox11/Gfap	3	0.422535211267606
GO:0046503	BP	GO:0046503	glycerolipid catabolic process	3/180	71/28943	0.00992081343405632	0.052369674805059	0.0366773056582787	Enpp2/Prdx6/Pla2g4a	3	0.422535211267606
GO:0072330	BP	GO:0072330	monocarboxylic acid biosynthetic process	5/180	209/28943	0.0100752502440189	0.0530988512051289	0.0371879871892165	Elovl6/Slc27a2/Pdk4/Aldh1a1/Pla2g4a	5	0.239234449760766
GO:0042157	BP	GO:0042157	lipoprotein metabolic process	4/180	135/28943	0.010185023351939	0.0535906640666645	0.0375324302417342	Abca1/Piga/Ctsd/Apoe	4	0.296296296296296
GO:0006949	BP	GO:0006949	syncytium formation	3/180	72/28943	0.0103056084904157	0.0540505102307312	0.0378544852932128	Adam8/Tyrobp/Cd53	3	0.416666666666667
GO:1904705	BP	GO:1904705	regulation of vascular associated smooth muscle cell proliferation	3/180	72/28943	0.0103056084904157	0.0540505102307312	0.0378544852932128	Jun/Mmp9/Fgf9	3	0.416666666666667
GO:0032303	BP	GO:0032303	regulation of icosanoid secretion	2/180	25/28943	0.0105040364532406	0.0548263569362252	0.0383978525542718	Agtr2/Pla2g4a	2	0.8
GO:0036010	BP	GO:0036010	protein localization to endosome	2/180	25/28943	0.0105040364532406	0.0548263569362252	0.0383978525542718	Msn/Nrp1	2	0.8
GO:0050860	BP	GO:0050860	negative regulation of T cell receptor signaling pathway	2/180	25/28943	0.0105040364532406	0.0548263569362252	0.0383978525542718	Lgals3/Laptm5	2	0.8

GO:0002763	BP	GO:0002763	positive regulation of myeloid leukocyte differentiation	3/180	73/28943	0.010698971218708	0.0555295919973915	0.0388903659675023	Jun/Tyrobp/Car2	3	0.410958904109589
GO:0043112	BP	GO:0043112	receptor metabolic process	3/180	73/28943	0.010698971218708	0.0555295919973915	0.0388903659675023	Laptm5/Apoe/Anxa2	3	0.410958904109589
GO:1905330	BP	GO:1905330	regulation of morphogenesis of an epithelium	3/180	73/28943	0.010698971218708	0.0555295919973915	0.0388903659675023	Sfrp1/Itgax/Agtr2	3	0.410958904109589
GO:0055076	BP	GO:0055076	transition metal ion homeostasis	4/180	137/28943	0.0107069646221559	0.0555295919973915	0.0388903659675023	Mt2/B2m/Abcb7/Mt1	4	0.291970802919708
GO:0031669	BP	GO:0031669	cellular response to nutrient levels	5/180	213/28943	0.0108713325539618	0.056292416738082	0.0394246132448083	Atf3/Jun/Cd68/Inhbb/Pdk4	5	0.234741784037559
GO:0010811	BP	GO:0010811	positive regulation of cell-substrate adhesion	4/180	138/28943	0.0109742926359628	0.0567353509767154	0.0397348239634608	Npy/S100a10/Enpp2/Nrp1	4	0.289855072463768
GO:0019216	BP	GO:0019216	regulation of lipid metabolic process	7/180	388/28943	0.0110596780184108	0.0570279892832184	0.0399397743408479	Fdps/Nr1d2/Igf2/Nucb2/Apoe/Pdk4/Pla2g4a	7	0.180412371134021
GO:0010633	BP	GO:0010633	negative regulation of epithelial cell migration	3/180	74/28943	0.0111009349725393	0.0570279892832184	0.0399397743408479	Gadd45a/Apoe/Cd63	3	0.405405405405405
GO:0045109	BP	GO:0045109	intermediate filament organization	3/180	74/28943	0.0111009349725393	0.0570279892832184	0.0399397743408479	Dsp/Ina/Gfap	3	0.405405405405405
GO:0098586	BP	GO:0098586	cellular response to virus	3/180	74/28943	0.0111009349725393	0.0570279892832184	0.0399397743408479	Mmp12/Ankrd17/Ifih1	3	0.405405405405405
GO:0034754	BP	GO:0034754	cellular hormone metabolic process	4/180	139/28943	0.0112458915871747	0.0573173547693498	0.0401424325857444	Adm/Ugt1a1/Rbp4/Aldh1a1	4	0.287769784172662
GO:0061844	BP	GO:0061844	antimicrobial humoral immune response mediated by antimicrobial peptide	4/180	139/28943	0.0112458915871747	0.0573173547693498	0.0401424325857444	Adm/Npy/Lgals3/B2m	4	0.287769784172662

GO:0002504	BP	GO:0002504	antigen processing and presentation of peptide or polysaccharide antigen via MHC class II	2/180	26/28943	0.0113332442344063	0.0573173547693498	0.0401424325857444	Ctss/B2m	2	0.769230769230769
GO:0006817	BP	GO:0006817	phosphate ion transport	2/180	26/28943	0.0113332442344063	0.0573173547693498	0.0401424325857444	Slc25a14/Sfrp4	2	0.769230769230769
GO:0006883	BP	GO:0006883	cellular sodium ion homeostasis	2/180	26/28943	0.0113332442344063	0.0573173547693498	0.0401424325857444	Sgk1/Agtr2	2	0.769230769230769
GO:0032098	BP	GO:0032098	regulation of appetite	2/180	26/28943	0.0113332442344063	0.0573173547693498	0.0401424325857444	Npy/Nucb2	2	0.769230769230769
GO:0032727	BP	GO:0032727	positive regulation of interferon-alpha production	2/180	26/28943	0.0113332442344063	0.0573173547693498	0.0401424325857444	Mmp12/Ifih1	2	0.769230769230769
GO:0065005	BP	GO:0065005	protein-lipid complex assembly	2/180	26/28943	0.0113332442344063	0.0573173547693498	0.0401424325857444	Abca1/Apoe	2	0.769230769230769
GO:0070723	BP	GO:0070723	response to cholesterol	2/180	26/28943	0.0113332442344063	0.0573173547693498	0.0401424325857444	Inhbb/Ccr5	2	0.769230769230769
GO:2000050	BP	GO:2000050	regulation of non-canonical Wnt signaling pathway	2/180	26/28943	0.0113332442344063	0.0573173547693498	0.0401424325857444	Sfrp1/Sfrp4	2	0.769230769230769
GO:0010611	BP	GO:0010611	regulation of cardiac muscle hypertrophy	3/180	75/28943	0.0115115314302182	0.0578219653537951	0.0404958385733316	Tnfrsf1b/Fdps/Lmna	3	0.4
GO:0050818	BP	GO:0050818	regulation of coagulation	3/180	75/28943	0.0115115314302182	0.0578219653537951	0.0404958385733316	Procr/Apoe/Anxa2	3	0.4
GO:1990874	BP	GO:1990874	vascular associated smooth muscle cell proliferation	3/180	75/28943	0.0115115314302182	0.0578219653537951	0.0404958385733316	Jun/Mmp9/Fgf9	3	0.4
GO:0030177	BP	GO:0030177	positive regulation of Wnt signaling pathway	4/180	140/28943	0.0115217855433261	0.0578219653537951	0.0404958385733316	Sfrp1/Fgf9/Sfrp4/Spin1	4	0.285714285714286

GO:0030278	BP	GO:0030278	regulation of ossification	4/180	140/28943	0.0115217855433261	0.0578219653537951	0.0404958385733316	Sox11/Sfrp1/Bglap/Egr2	4	0.285714285714286
GO:0044262	BP	GO:0044262	cellular carbohydrate metabolic process	6/180	302/28943	0.0117934298950014	0.0589556873956737	0.0412898452196645	Stbd1/Ugt1a1/Igf2/Ddit4/Pdk4/Pla2g4a	6	0.198675496688742
GO:0008584	BP	GO:0008584	male gonad development	4/180	141/28943	0.0118019982136872	0.0589556873956737	0.0412898452196645	Sfrp1/Fgf9/Fndc3a/Rbp4	4	0.283687943262411
GO:0055123	BP	GO:0055123	digestive system development	4/180	141/28943	0.0118019982136872	0.0589556873956737	0.0412898452196645	Sox11/Sfrp1/Fgf9/Igf2	4	0.283687943262411
GO:0002703	BP	GO:0002703	regulation of leukocyte mediated immunity	6/180	303/28943	0.0119714532234315	0.0597106020654157	0.0418185526479828	Ccl2/Tyrobp/Tnfrsf1b/B2m/Itgb2/Igf2	6	0.198019801980198
GO:0002702	BP	GO:0002702	positive regulation of production of molecular mediator of immune response	4/180	142/28943	0.0120865529482487	0.0597949487232235	0.0418776251581938	B2m/Sema7a/Laptm5/Rbp4	4	0.28169014084507
GO:0046546	BP	GO:0046546	development of primary male sexual characteristics	4/180	142/28943	0.0120865529482487	0.0597949487232235	0.0418776251581938	Sfrp1/Fgf9/Fndc3a/Rbp4	4	0.28169014084507
GO:0060538	BP	GO:0060538	skeletal muscle organ development	5/180	219/28943	0.0121444733019223	0.0597949487232235	0.0418776251581938	Atf3/Sox11/Nr1d2/Igf2/Egr2	5	0.228310502283105
GO:0010839	BP	GO:0010839	negative regulation of keratinocyte proliferation	2/180	27/28943	0.0121903119288365	0.0597949487232235	0.0418776251581938	Ctsl/Efnb2	2	0.740740740740741
GO:0015740	BP	GO:0015740	C4-dicarboxylate transport	2/180	27/28943	0.0121903119288365	0.0597949487232235	0.0418776251581938	Slc25a14/Gfap	2	0.740740740740741
GO:0035809	BP	GO:0035809	regulation of urine volume	2/180	27/28943	0.0121903119288365	0.0597949487232235	0.0418776251581938	Adm/Npr3	2	0.740740740740741
GO:0036003	BP	GO:0036003	positive regulation of transcription from RNA polymerase II promoter in response to stress	2/180	27/28943	0.0121903119288365	0.0597949487232235	0.0418776251581938	Atf3/Atf6	2	0.740740740740741

GO:0045780	BP	GO:0045780	positive regulation of bone resorption	2/180	27/28943	0.0121903119288365	0.0597949487232235	0.0418776251581938	Adam8/Car2	2	0.740740740740741
GO:0048843	BP	GO:0048843	negative regulation of axon extension involved in axon guidance	2/180	27/28943	0.0121903119288365	0.0597949487232235	0.0418776251581938	Sema7a/Nrp1	2	0.740740740740741
GO:0060143	BP	GO:0060143	positive regulation of syncytium formation by plasma membrane fusion	2/180	27/28943	0.0121903119288365	0.0597949487232235	0.0418776251581938	Tyrobp/Cd53	2	0.740740740740741
GO:0071549	BP	GO:0071549	cellular response to dexamethasone stimulus	2/180	27/28943	0.0121903119288365	0.0597949487232235	0.0418776251581938	Ddit4/Agtr2	2	0.740740740740741
GO:0002090	BP	GO:0002090	regulation of receptor internalization	3/180	77/28943	0.0123587409431478	0.060439067945694	0.0423287365636181	Sfrp4/Cd63/Efnb2	3	0.38961038961039
GO:0002437	BP	GO:0002437	inflammatory response to antigenic stimulus	3/180	77/28943	0.0123587409431478	0.060439067945694	0.0423287365636181	Adcyap1/Npy/Cd68	3	0.38961038961039
GO:0030258	BP	GO:0030258	lipid modification	5/180	221/28943	0.0125904066607605	0.0614796918951978	0.043057541598466	Slc27a2/Nucb2/Apoe/Sacm1l/Pdk4	5	0.226244343891403
GO:0014743	BP	GO:0014743	regulation of muscle hypertrophy	3/180	78/28943	0.0127954092014924	0.0623871972593723	0.043693116513763	Tnfrsf1b/Fdps/Lmna	3	0.384615384615385
GO:1901888	BP	GO:1901888	regulation of cell junction assembly	5/180	222/28943	0.0128174807508866	0.0624013973178442	0.0437030615800037	S100a10/Enpp2/Nrp1/Bdnf/Cbln1	5	0.225225225225225
GO:0071496	BP	GO:0071496	cellular response to external stimulus	6/180	308/28943	0.0128900767742932	0.0626611642595118	0.043884990369039	Atf3/Jun/Mmp9/Cd68/Inhbb/Pdk4	6	0.194805194805195
GO:0008654	BP	GO:0008654	phospholipid biosynthetic process	5/180	223/28943	0.0130473123654544	0.0629022675256985	0.0440538479802817	Idi1/Piga/Fdps/Nus1/Pla2g4a	5	0.224215246636771
GO:0001778	BP	GO:0001778	plasma membrane repair	2/180	28/28943	0.0130748649416328	0.0629022675256985	0.0440538479802817	S100a10/Anxa2	2	0.714285714285714

GO:0006516	BP	GO:0006516	glycoprotein catabolic process	2/180	28/28943	0.0130748649416328	0.0629022675256985	0.0440538479802817	Mmp12/Ctsl	2	0.714285714285714
GO:0006882	BP	GO:0006882	cellular zinc ion homeostasis	2/180	28/28943	0.0130748649416328	0.0629022675256985	0.0440538479802817	Mt2/Mt1	2	0.714285714285714
GO:0042104	BP	GO:0042104	positive regulation of activated T cell proliferation	2/180	28/28943	0.0130748649416328	0.0629022675256985	0.0440538479802817	Igfbp2/Igf2	2	0.714285714285714
GO:0046688	BP	GO:0046688	response to copper ion	2/180	28/28943	0.0130748649416328	0.0629022675256985	0.0440538479802817	Mt2/Mt1	2	0.714285714285714
GO:0098698	BP	GO:0098698	postsynaptic specialization assembly	2/180	28/28943	0.0130748649416328	0.0629022675256985	0.0440538479802817	Gap43/Cbln1	2	0.714285714285714
GO:0048002	BP	GO:0048002	antigen processing and presentation of peptide antigen	3/180	79/28943	0.0132408206183606	0.0636067149764019	0.0445472104952452	Ctss/B2m/Ctsl	3	0.379746835443038
GO:0031341	BP	GO:0031341	regulation of cell killing	4/180	146/28943	0.0132686469030302	0.0636465139369212	0.0445750838522077	Ccl2/Tyrobp/B2m/Igf2	4	0.273972602739726
GO:2000241	BP	GO:2000241	regulation of reproductive process	5/180	225/28943	0.0135153052474085	0.0647343370453079	0.0453369450017557	Mmp12/Sfrp1/Cyp51/Fgf9/Inhbb	5	0.222222222222222
GO:0048706	BP	GO:0048706	embryonic skeletal system development	4/180	147/28943	0.0135752495721048	0.0647358533767865	0.0453380069704107	Hoxa7/Sox11/Fgf9/Rbp4	4	0.272108843537415
GO:0120161	BP	GO:0120161	regulation of cold-induced thermogenesis	4/180	147/28943	0.0135752495721048	0.0647358533767865	0.0453380069704107	Adcyap1/Elovl6/Npr3/Aldh1a1	4	0.272108843537415
GO:1901655	BP	GO:1901655	cellular response to ketone	4/180	147/28943	0.0135752495721048	0.0647358533767865	0.0453380069704107	Msn/Sgk1/Ddit4/Agtr2	4	0.272108843537415
GO:0002367	BP	GO:0002367	cytokine production involved in immune response	4/180	148/28943	0.0138863268130531	0.0661224655411023	0.0463091261986551	Tnfrsf1b/B2m/Sema7a/Laptm5	4	0.27027027027027

GO:0002888	BP	GO:0002888	positive regulation of myeloid leukocyte mediated immunity	2/180	29/28943	0.0139865322141676	0.0662124061359651	0.0463721164444634	Tyrobp/Itgb2	2	0.689655172413793
GO:0010893	BP	GO:0010893	positive regulation of steroid biosynthetic process	2/180	29/28943	0.0139865322141676	0.0662124061359651	0.0463721164444634	Fdps/Igf2	2	0.689655172413793
GO:0010954	BP	GO:0010954	positive regulation of protein processing	2/180	29/28943	0.0139865322141676	0.0662124061359651	0.0463721164444634	S100a10/Clec3b	2	0.689655172413793
GO:0055069	BP	GO:0055069	zinc ion homeostasis	2/180	29/28943	0.0139865322141676	0.0662124061359651	0.0463721164444634	Mt2/Mt1	2	0.689655172413793
GO:0045123	BP	GO:0045123	cellular extravasation	3/180	81/28943	0.0141579660788733	0.0667329891735026	0.0467367088017646	Adam8/Ccl2/Itgb2	3	0.37037037037037
GO:0048844	BP	GO:0048844	artery morphogenesis	3/180	81/28943	0.0141579660788733	0.0667329891735026	0.0467367088017646	Nrp1/Apoe/Efnb2	3	0.37037037037037
GO:0051145	BP	GO:0051145	smooth muscle cell differentiation	3/180	81/28943	0.0141579660788733	0.0667329891735026	0.0467367088017646	Adm/Ankrd17/Fgf9	3	0.37037037037037
GO:0106106	BP	GO:0106106	cold-induced thermogenesis	4/180	149/28943	0.0142018994387335	0.066843333051958	0.04681398856668	Adcyap1/Elovl6/Npr3/Aldh1a1	4	0.268456375838926
GO:0030595	BP	GO:0030595	leukocyte chemotaxis	5/180	228/28943	0.014238329760111	0.0669180952794829	0.0468663485838241	Adam8/Ccl2/Scg2/Lgals3/Itgb2	5	0.219298245614035
GO:0055001	BP	GO:0055001	muscle cell development	5/180	229/28943	0.0144850027884185	0.0678595618096478	0.047525708333955	Adm/Fdps/Lmna/Col14a1/Agtr2	5	0.218340611353712
GO:0061138	BP	GO:0061138	morphogenesis of a branching epithelium	5/180	229/28943	0.0144850027884185	0.0678595618096478	0.047525708333955	Adm/Sfrp1/Ctsz/Nrp1/Agtr2	5	0.218340611353712
GO:0019233	BP	GO:0019233	sensory perception of pain	4/180	150/28943	0.0145219878972442	0.0678595618096478	0.047525708333955	Adcyap1/Ctss/Ccl2/Penk	4	0.266666666666667

GO:0046328	BP	GO:0046328	regulation of JNK cascade	4/180	150/28943	0.0145219878972442	0.0678595618096478	0.047525708333955	Gadd45a/Sfrp1/Sfrp4/Mdfic	4	0.266666666666667
GO:0003208	BP	GO:0003208	cardiac ventricle morphogenesis	3/180	82/28943	0.0146297428969019	0.0682651470131939	0.0478097615104604	Dsp/Sox11/Fgf9	3	0.365853658536585
GO:0050921	BP	GO:0050921	positive regulation of chemotaxis	4/180	151/28943	0.0148466122715502	0.0684655630381522	0.0479501236538874	Ccl2/Scg2/Nrp1/Lpar1	4	0.264900662251656
GO:1903900	BP	GO:1903900	regulation of viral life cycle	4/180	151/28943	0.0148466122715502	0.0684655630381522	0.0479501236538874	Adarb1/Ifih1/Lgals1/Trim25	4	0.264900662251656
GO:0006929	BP	GO:0006929	substrate-dependent cell migration	2/180	30/28943	0.0149249461949917	0.0684655630381522	0.0479501236538874	Adam8/Nrp1	2	0.666666666666667
GO:0039528	BP	GO:0039528	cytoplasmic pattern recognition receptor signaling pathway in response to virus	2/180	30/28943	0.0149249461949917	0.0684655630381522	0.0479501236538874	Ankrd17/Ifih1	2	0.666666666666667
GO:0042744	BP	GO:0042744	hydrogen peroxide catabolic process	2/180	30/28943	0.0149249461949917	0.0684655630381522	0.0479501236538874	Gpx3/Prdx6	2	0.666666666666667
GO:0048596	BP	GO:0048596	embryonic camera-type eye morphogenesis	2/180	30/28943	0.0149249461949917	0.0684655630381522	0.0479501236538874	Sox11/Aldh1a1	2	0.666666666666667
GO:0060343	BP	GO:0060343	trabecula formation	2/180	30/28943	0.0149249461949917	0.0684655630381522	0.0479501236538874	Sfrp1/Rbp4	2	0.666666666666667
GO:0060907	BP	GO:0060907	positive regulation of macrophage cytokine production	2/180	30/28943	0.0149249461949917	0.0684655630381522	0.0479501236538874	Sema7a/Laptm5	2	0.666666666666667
GO:0071548	BP	GO:0071548	response to dexamethasone	2/180	30/28943	0.0149249461949917	0.0684655630381522	0.0479501236538874	Ddit4/Agtr2	2	0.666666666666667
GO:0090314	BP	GO:0090314	positive regulation of protein targeting to membrane	2/180	30/28943	0.0149249461949917	0.0684655630381522	0.0479501236538874	Ccl2/Ank3	2	0.666666666666667

GO:0150077	BP	GO:0150077	regulation of neuroinflammatory response	2/180	30/28943	0.0149249461949917	0.0684655630381522	0.0479501236538874	Cst7/Tnfrsf1b	2	0.666666666666667
GO:1905562	BP	GO:1905562	regulation of vascular endothelial cell proliferation	2/180	30/28943	0.0149249461949917	0.0684655630381522	0.0479501236538874	Ccl2/Igf2	2	0.666666666666667
GO:0008625	BP	GO:0008625	extrinsic apoptotic signaling pathway via death domain receptors	3/180	83/28943	0.0151103484685917	0.0690244108866804	0.0483415149205554	Atf3/Sfrp1/Lgals3	3	0.36144578313253
GO:0046785	BP	GO:0046785	microtubule polymerization	3/180	83/28943	0.0151103484685917	0.0690244108866804	0.0483415149205554	Slain1/Sgk1/Mapt	3	0.36144578313253
GO:0050810	BP	GO:0050810	regulation of steroid biosynthetic process	3/180	83/28943	0.0151103484685917	0.0690244108866804	0.0483415149205554	Fdps/Igf2/Apoe	3	0.36144578313253
GO:0046660	BP	GO:0046660	female sex differentiation	4/180	152/28943	0.0151757922791738	0.0692262681418334	0.0484828864351609	Adcyap1/Sfrp1/Rbp4/Pla2g4a	4	0.263157894736842
GO:0048545	BP	GO:0048545	response to steroid hormone	5/180	232/28943	0.0152422069034051	0.0693350110117182	0.0485590449274901	Sfrp1/Sgk1/Bdnf/Ddit4/Agtr2	5	0.21551724137931
GO:0051651	BP	GO:0051651	maintenance of location in cell	5/180	232/28943	0.0152422069034051	0.0693350110117182	0.0485590449274901	Rit2/Tmsb10/Apoe/Ank3/Ccr5	5	0.21551724137931
GO:0034504	BP	GO:0034504	protein localization to nucleus	6/180	320/28943	0.0152945795762037	0.0693794508073752	0.0485901685114109	Mmp12/Kpna1/Fgf9/Lmna/Mdfic/Agtr2	6	0.1875
GO:0072503	BP	GO:0072503	cellular divalent inorganic cation homeostasis	6/180	320/28943	0.0152945795762037	0.0693794508073752	0.0485901685114109	Ccl2/Mt2/Mt1/Nucb2/Apoe/Ccr5	6	0.1875
GO:0034109	BP	GO:0034109	homotypic cell-cell adhesion	3/180	84/28943	0.0155998004837952	0.0705674307996125	0.0494221749245383	Dsp/Ank3/Lgals1	3	0.357142857142857
GO:1900076	BP	GO:1900076	regulation of cellular response to insulin stimulus	3/180	84/28943	0.0155998004837952	0.0705674307996125	0.0494221749245383	Igf2/Bglap/Nucb2	3	0.357142857142857

GO:0030947	BP	GO:0030947	regulation of vascular endothelial growth factor receptor signaling pathway	2/180	31/28943	0.0158897428109626	0.0707006725892146	0.0495154913306814	Fgf9/Nrp1	2	0.645161290322581
GO:0031116	BP	GO:0031116	positive regulation of microtubule polymerization	2/180	31/28943	0.0158897428109626	0.0707006725892146	0.0495154913306814	Slain1/Mapt	2	0.645161290322581
GO:0032647	BP	GO:0032647	regulation of interferon-alpha production	2/180	31/28943	0.0158897428109626	0.0707006725892146	0.0495154913306814	Mmp12/Ifih1	2	0.645161290322581
GO:0033137	BP	GO:0033137	negative regulation of peptidyl-serine phosphorylation	2/180	31/28943	0.0158897428109626	0.0707006725892146	0.0495154913306814	Gadd45a/Ddit4	2	0.645161290322581
GO:0033688	BP	GO:0033688	regulation of osteoblast proliferation	2/180	31/28943	0.0158897428109626	0.0707006725892146	0.0495154913306814	Sfrp1/Npr3	2	0.645161290322581
GO:0035094	BP	GO:0035094	response to nicotine	2/180	31/28943	0.0158897428109626	0.0707006725892146	0.0495154913306814	B2m/Igf2	2	0.645161290322581
GO:0036314	BP	GO:0036314	response to sterol	2/180	31/28943	0.0158897428109626	0.0707006725892146	0.0495154913306814	Inhbb/Ccr5	2	0.645161290322581
GO:0050858	BP	GO:0050858	negative regulation of antigen receptor-mediated signaling pathway	2/180	31/28943	0.0158897428109626	0.0707006725892146	0.0495154913306814	Lgals3/Laptm5	2	0.645161290322581
GO:0060384	BP	GO:0060384	innervation	2/180	31/28943	0.0158897428109626	0.0707006725892146	0.0495154913306814	Adarb1/Nrp1	2	0.645161290322581
GO:0101023	BP	GO:0101023	vascular endothelial cell proliferation	2/180	31/28943	0.0158897428109626	0.0707006725892146	0.0495154913306814	Ccl2/Igf2	2	0.645161290322581
GO:1903319	BP	GO:1903319	positive regulation of protein maturation	2/180	31/28943	0.0158897428109626	0.0707006725892146	0.0495154913306814	S100a10/Clec3b	2	0.645161290322581
GO:2000178	BP	GO:2000178	negative regulation of neural precursor cell proliferation	2/180	31/28943	0.0158897428109626	0.0707006725892146	0.0495154913306814	Bdnf/Ccr5	2	0.645161290322581

GO:0003018	BP	GO:0003018	vascular process in circulatory system	5/180	235/28943	0.0160254679685484	0.0712072976446962	0.0498703081609025	Adcyap1/Adm/Apoe/Npr3/Agtr2	5	0.212765957446809
GO:0002720	BP	GO:0002720	positive regulation of cytokine production involved in immune response	3/180	85/28943	0.0160981151942612	0.0713354573982431	0.0499600653433068	B2m/Sema7a/Laptm5	3	0.352941176470588
GO:0014855	BP	GO:0014855	striated muscle cell proliferation	3/180	85/28943	0.0160981151942612	0.0713354573982431	0.0499600653433068	Kpna1/Fgf9/Rbp4	3	0.352941176470588
GO:0072073	BP	GO:0072073	kidney epithelium development	4/180	155/28943	0.0161908577908406	0.0716489454140868	0.0501796178958118	Sfrp1/Bdnf/Agtr2/Efnb2	4	0.258064516129032
GO:0030324	BP	GO:0030324	lung development	5/180	236/28943	0.0162924063787723	0.072000498745809	0.0504258296405049	Mmp12/Sox11/Ctsz/Fgf9/Rbp4	5	0.211864406779661
GO:0010565	BP	GO:0010565	regulation of cellular ketone metabolic process	4/180	156/28943	0.0165384501908578	0.0727906949109117	0.0509792466014724	Ugt1a1/Nucb2/Pdk4/Pla2g4a	4	0.256410256410256
GO:0030216	BP	GO:0030216	keratinocyte differentiation	4/180	156/28943	0.0165384501908578	0.0727906949109117	0.0509792466014724	Sprr1a/Dsp/Sprr1b/Hoxa7	4	0.256410256410256
GO:2001235	BP	GO:2001235	positive regulation of apoptotic signaling pathway	4/180	156/28943	0.0165384501908578	0.0727906949109117	0.0509792466014724	Atf3/Sfrp1/Inhbb/Agtr2	4	0.256410256410256
GO:0003151	BP	GO:0003151	outflow tract morphogenesis	3/180	86/28943	0.0166053074359249	0.0727906949109117	0.0509792466014724	Jun/Sox11/Nrp1	3	0.348837209302326
GO:0048041	BP	GO:0048041	focal adhesion assembly	3/180	86/28943	0.0166053074359249	0.0727906949109117	0.0509792466014724	S100a10/Enpp2/Nrp1	3	0.348837209302326
GO:0051384	BP	GO:0051384	response to glucocorticoid	3/180	86/28943	0.0166053074359249	0.0727906949109117	0.0509792466014724	Sgk1/Ddit4/Agtr2	3	0.348837209302326
GO:0006887	BP	GO:0006887	exocytosis	7/180	422/28943	0.0168197241734259	0.073014593101595	0.0511360545710959	Lin7b/S100a10/Itgb2/Lat2/Vsnl1/Anxa2/Pclo	7	0.165876777251185

GO:0001893	BP	GO:0001893	maternal placenta development	2/180	32/28943	0.0168805614385941	0.073014593101595	0.0511360545710959	Ctsl/Pla2g4a	2	0.625
GO:0010875	BP	GO:0010875	positive regulation of cholesterol efflux	2/180	32/28943	0.0168805614385941	0.073014593101595	0.0511360545710959	Abca1/Apoe	2	0.625
GO:0030224	BP	GO:0030224	monocyte differentiation	2/180	32/28943	0.0168805614385941	0.073014593101595	0.0511360545710959	Jun/Hoxa7	2	0.625
GO:0031295	BP	GO:0031295	T cell costimulation	2/180	32/28943	0.0168805614385941	0.073014593101595	0.0511360545710959	Lgals1/Efnb2	2	0.625
GO:0048011	BP	GO:0048011	neurotrophin TRK receptor signaling pathway	2/180	32/28943	0.0168805614385941	0.073014593101595	0.0511360545710959	Ddit4/Agtr2	2	0.625
GO:0048714	BP	GO:0048714	positive regulation of oligodendrocyte differentiation	2/180	32/28943	0.0168805614385941	0.073014593101595	0.0511360545710959	Tnfrsf1b/Enpp2	2	0.625
GO:0051150	BP	GO:0051150	regulation of smooth muscle cell differentiation	2/180	32/28943	0.0168805614385941	0.073014593101595	0.0511360545710959	Ankrd17/Fgf9	2	0.625
GO:0060977	BP	GO:0060977	coronary vasculature morphogenesis	2/180	32/28943	0.0168805614385941	0.073014593101595	0.0511360545710959	Fgf9/Nrp1	2	0.625
GO:0061050	BP	GO:0061050	regulation of cell growth involved in cardiac muscle cell development	2/180	32/28943	0.0168805614385941	0.073014593101595	0.0511360545710959	Fdps/Col14a1	2	0.625
GO:0002366	BP	GO:0002366	leukocyte activation involved in immune response	6/180	328/28943	0.0170605908331356	0.0736954169012237	0.0516128721700473	Tyrobp/Lgals3/Itgb2/Lat2/Lgals1/Apbb1ip	6	0.182926829268293
GO:0030323	BP	GO:0030323	respiratory tube development	5/180	239/28943	0.0171109543199165	0.0737623933202902	0.0516597793659224	Mmp12/Sox11/Ctsz/Fgf9/Rbp4	5	0.209205020920502
GO:0048708	BP	GO:0048708	astrocyte differentiation	3/180	87/28943	0.0171213906509485	0.0737623933202902	0.0516597793659224	C1qa/Gap43/Gfap	3	0.344827586206897

GO:0045088	BP	GO:0045088	regulation of innate immune response	5/180	240/28943	0.0173897562130777	0.0748195983962932	0.052400197057275	Mmp12/Adam8/Tyrobp/Igf2/Apoe	5	0.208333333333333
GO:0006959	BP	GO:0006959	humoral immune response	7/180	425/28943	0.0174134581065678	0.0748227348985375	0.0524023937188342	C1qa/Adm/Npy/C4a/C1qc/Lgals3/B2m	7	0.164705882352941
GO:0043525	BP	GO:0043525	positive regulation of neuron apoptotic process	3/180	88/28943	0.0176463769095139	0.0756240126240615	0.0529635716937903	Jun/Ctsz/Tyrobp	3	0.340909090909091
GO:0051881	BP	GO:0051881	regulation of mitochondrial membrane potential	3/180	88/28943	0.0176463769095139	0.0756240126240615	0.0529635716937903	Clic1/Mapt/Ppa2	3	0.340909090909091
GO:0042180	BP	GO:0042180	cellular ketone metabolic process	5/180	241/28943	0.017671554917451	0.0756323973273823	0.0529694439534081	Ugt1a1/Oxct1/Nucb2/Pdk4/Pla2g4a	5	0.20746887966805
GO:0002438	BP	GO:0002438	acute inflammatory response to antigenic stimulus	2/180	33/28943	0.0178970448756245	0.0758006178932497	0.0530872578817033	Adcyap1/Npy	2	0.606060606060606
GO:0002478	BP	GO:0002478	antigen processing and presentation of exogenous peptide antigen	2/180	33/28943	0.0178970448756245	0.0758006178932497	0.0530872578817033	Ctss/B2m	2	0.606060606060606
GO:0002675	BP	GO:0002675	positive regulation of acute inflammatory response	2/180	33/28943	0.0178970448756245	0.0758006178932497	0.0530872578817033	Adam8/Ccr5	2	0.606060606060606
GO:0048566	BP	GO:0048566	embryonic digestive tract development	2/180	33/28943	0.0178970448756245	0.0758006178932497	0.0530872578817033	Sox11/Fgf9	2	0.606060606060606
GO:0048841	BP	GO:0048841	regulation of axon extension involved in axon guidance	2/180	33/28943	0.0178970448756245	0.0758006178932497	0.0530872578817033	Sema7a/Nrp1	2	0.606060606060606
GO:0060669	BP	GO:0060669	embryonic placenta morphogenesis	2/180	33/28943	0.0178970448756245	0.0758006178932497	0.0530872578817033	Adm/Igf2	2	0.606060606060606
GO:0099068	BP	GO:0099068	postsynapse assembly	2/180	33/28943	0.0178970448756245	0.0758006178932497	0.0530872578817033	Gap43/Cbln1	2	0.606060606060606

GO:1903792	BP	GO:1903792	negative regulation of anion transport	2/180	33/28943	0.0178970448756245	0.0758006178932497	0.0530872578817033	Sfrp4/Agtr2	2	0.606060606060606
GO:0009913	BP	GO:0009913	epidermal cell differentiation	5/180	242/28943	0.0179563625544845	0.0759161407168198	0.0531681647409947	Sprr1a/Dsp/Sprr1b/Hoxa7/Sfrp4	5	0.206611570247934
GO:0002687	BP	GO:0002687	positive regulation of leukocyte migration	4/180	160/28943	0.0179754805095395	0.0759161407168198	0.0531681647409947	Adam8/Ccl2/Mmp9/Lgals3	4	0.25
GO:0002263	BP	GO:0002263	cell activation involved in immune response	6/180	332/28943	0.0179942464333389	0.0759161407168198	0.0531681647409947	Tyrobp/Lgals3/Itgb2/Lat2/Lgals1/Apbb1ip	6	0.180722891566265
GO:0007218	BP	GO:0007218	neuropeptide signaling pathway	3/180	89/28943	0.0181802769313697	0.0764040799554468	0.0535098948866258	Adcyap1/Npy/Penk	3	0.337078651685393
GO:0014032	BP	GO:0014032	neural crest cell development	3/180	89/28943	0.0181802769313697	0.0764040799554468	0.0535098948866258	Sox11/Sema7a/Nrp1	3	0.337078651685393
GO:0034620	BP	GO:0034620	cellular response to unfolded protein	3/180	89/28943	0.0181802769313697	0.0764040799554468	0.0535098948866258	Atf3/Atf6/Hspb8	3	0.337078651685393
GO:0097529	BP	GO:0097529	myeloid leukocyte migration	5/180	243/28943	0.0182441911169935	0.0764753287877062	0.0535597942837158	Adam8/Ccl2/Scg2/Lgals3/Itgb2	5	0.205761316872428
GO:2000116	BP	GO:2000116	regulation of cysteine-type endopeptidase activity	5/180	243/28943	0.0182441911169935	0.0764753287877062	0.0535597942837158	Cst7/Mmp9/Cst3/Ctsd/Laptm5	5	0.205761316872428
GO:0070507	BP	GO:0070507	regulation of microtubule cytoskeleton organization	4/180	161/28943	0.0183464881350079	0.0768052851615949	0.0537908805148995	Slain1/Sgk1/Mapt/Mark2	4	0.248447204968944
GO:0050806	BP	GO:0050806	positive regulation of synaptic transmission	6/180	335/28943	0.0187171069738137	0.0779809936687272	0.0546142925456529	Adcyap1/Ccl2/Tyrobp/Car2/Apoe/Gfap	6	0.17910447761194
GO:0006835	BP	GO:0006835	dicarboxylic acid transport	3/180	90/28943	0.0187231001071368	0.0779809936687272	0.0546142925456529	Slc25a14/Bdnf/Gfap	3	0.333333333333333

GO:0019915	BP	GO:0019915	lipid storage	3/180	90/28943	0.0187231001071368	0.0779809936687272	0.0546142925456529	Msr1/Sqle/Apoe	3	0.333333333333333
GO:0050805	BP	GO:0050805	negative regulation of synaptic transmission	3/180	90/28943	0.0187231001071368	0.0779809936687272	0.0546142925456529	Bdnf/Mapt/Cbln1	3	0.333333333333333
GO:0000038	BP	GO:0000038	very long-chain fatty acid metabolic process	2/180	34/28943	0.0189388393128013	0.0782789335555761	0.0548229559054776	Elovl6/Slc27a2	2	0.588235294117647
GO:0002092	BP	GO:0002092	positive regulation of receptor internalization	2/180	34/28943	0.0189388393128013	0.0782789335555761	0.0548229559054776	Sfrp4/Cd63	2	0.588235294117647
GO:0010614	BP	GO:0010614	negative regulation of cardiac muscle hypertrophy	2/180	34/28943	0.0189388393128013	0.0782789335555761	0.0548229559054776	Tnfrsf1b/Lmna	2	0.588235294117647
GO:0031294	BP	GO:0031294	lymphocyte costimulation	2/180	34/28943	0.0189388393128013	0.0782789335555761	0.0548229559054776	Lgals1/Efnb2	2	0.588235294117647
GO:0032607	BP	GO:0032607	interferon-alpha production	2/180	34/28943	0.0189388393128013	0.0782789335555761	0.0548229559054776	Mmp12/Ifih1	2	0.588235294117647
GO:0051973	BP	GO:0051973	positive regulation of telomerase activity	2/180	34/28943	0.0189388393128013	0.0782789335555761	0.0548229559054776	Pkib/Parm1	2	0.588235294117647
GO:0009755	BP	GO:0009755	hormone-mediated signaling pathway	4/180	163/28943	0.0191027171371869	0.0788562100327219	0.0552272537339523	Sfrp1/Nr1d2/Sgk1/Bdnf	4	0.245398773006135
GO:0001912	BP	GO:0001912	positive regulation of leukocyte mediated cytotoxicity	3/180	91/28943	0.0192748545193717	0.0793656146265406	0.0555840172246702	Ccl2/Tyrobp/B2m	3	0.32967032967033
GO:0060079	BP	GO:0060079	excitatory postsynaptic potential	3/180	91/28943	0.0192748545193717	0.0793656146265406	0.0555840172246702	Bdnf/Cbln1/Pclo	3	0.32967032967033
GO:0016042	BP	GO:0016042	lipid catabolic process	6/180	338/28943	0.0194596102752722	0.0800251902355576	0.056045953570594	Slc27a2/Enpp2/Oxct1/Apoe/Prdx6/Pla2g4a	6	0.177514792899408

GO:0031110	BP	GO:0031110	regulation of microtubule polymerization or depolymerization	3/180	92/28943	0.0198355469633928	0.0809613306761537	0.0567015831731574	Slain1/Sgk1/Mapt	3	0.326086956521739
GO:0032024	BP	GO:0032024	positive regulation of insulin secretion	3/180	92/28943	0.0198355469633928	0.0809613306761537	0.0567015831731574	Vsnl1/Oxct1/Rbp4	3	0.326086956521739
GO:0045638	BP	GO:0045638	negative regulation of myeloid cell differentiation	3/180	92/28943	0.0198355469633928	0.0809613306761537	0.0567015831731574	Hoxa7/Sfrp1/C1qc	3	0.326086956521739
GO:2001259	BP	GO:2001259	positive regulation of cation channel activity	3/180	92/28943	0.0198355469633928	0.0809613306761537	0.0567015831731574	Ctss/Ccl2/Ank3	3	0.326086956521739
GO:1990845	BP	GO:1990845	adaptive thermogenesis	4/180	165/28943	0.0198780006172367	0.0809613306761537	0.0567015831731574	Adcyap1/Elovl6/Npr3/Aldh1a1	4	0.242424242424242
GO:0014002	BP	GO:0014002	astrocyte development	2/180	35/28943	0.020005594305882	0.0809613306761537	0.0567015831731574	C1qa/Gfap	2	0.571428571428571
GO:0031112	BP	GO:0031112	positive regulation of microtubule polymerization or depolymerization	2/180	35/28943	0.020005594305882	0.0809613306761537	0.0567015831731574	Slain1/Mapt	2	0.571428571428571
GO:0032104	BP	GO:0032104	regulation of response to extracellular stimulus	2/180	35/28943	0.020005594305882	0.0809613306761537	0.0567015831731574	Npy/Nucb2	2	0.571428571428571
GO:0032107	BP	GO:0032107	regulation of response to nutrient levels	2/180	35/28943	0.020005594305882	0.0809613306761537	0.0567015831731574	Npy/Nucb2	2	0.571428571428571
GO:0035025	BP	GO:0035025	positive regulation of Rho protein signal transduction	2/180	35/28943	0.020005594305882	0.0809613306761537	0.0567015831731574	Arhgef3/Lpar1	2	0.571428571428571
GO:0045672	BP	GO:0045672	positive regulation of osteoclast differentiation	2/180	35/28943	0.020005594305882	0.0809613306761537	0.0567015831731574	Tyrobp/Car2	2	0.571428571428571
GO:0060142	BP	GO:0060142	regulation of syncytium formation by plasma membrane fusion	2/180	35/28943	0.020005594305882	0.0809613306761537	0.0567015831731574	Tyrobp/Cd53	2	0.571428571428571

GO:0001763	BP	GO:0001763	morphogenesis of a branching structure	5/180	249/28943	0.0200352571461406	0.0809613306761537	0.0567015831731574	Adm/Sfrp1/Ctsz/Nrp1/Agtr2	5	0.200803212851406
GO:0060828	BP	GO:0060828	regulation of canonical Wnt signaling pathway	5/180	249/28943	0.0200352571461406	0.0809613306761537	0.0567015831731574	Sfrp1/Kpna1/Fgf9/Sfrp4/Apoe	5	0.200803212851406
GO:0010675	BP	GO:0010675	regulation of cellular carbohydrate metabolic process	4/180	167/28943	0.020672456851404	0.0834327037980458	0.0584324189617069	Ugt1a1/Igf2/Ddit4/Pdk4	4	0.239520958083832
GO:0043270	BP	GO:0043270	positive regulation of ion transport	6/180	343/28943	0.0207413745029098	0.083607248460368	0.058554661995578	Ctss/Ccl2/Lgals3/Sgk1/Ank3/Pla2g4a	6	0.174927113702624
GO:0031640	BP	GO:0031640	killing of cells of another organism	3/180	94/28943	0.0209837668151844	0.0841038037573335	0.0589024264312143	Lgals3/Lyz1/Lyz2	3	0.319148936170213
GO:0051899	BP	GO:0051899	membrane depolarization	3/180	94/28943	0.0209837668151844	0.0841038037573335	0.0589024264312143	Jun/B2m/Ank3	3	0.319148936170213
GO:0030178	BP	GO:0030178	negative regulation of Wnt signaling pathway	4/180	168/28943	0.021076910123911	0.0841038037573335	0.0589024264312143	Sfrp1/Fgf9/Sfrp4/Apoe	4	0.238095238095238
GO:0008045	BP	GO:0008045	motor neuron axon guidance	2/180	36/28943	0.0210969627478482	0.0841038037573335	0.0589024264312143	Nrp1/Egr2	2	0.555555555555556
GO:0010043	BP	GO:0010043	response to zinc ion	2/180	36/28943	0.0210969627478482	0.0841038037573335	0.0589024264312143	Mt2/Mt1	2	0.555555555555556
GO:0014741	BP	GO:0014741	negative regulation of muscle hypertrophy	2/180	36/28943	0.0210969627478482	0.0841038037573335	0.0589024264312143	Tnfrsf1b/Lmna	2	0.555555555555556
GO:0032369	BP	GO:0032369	negative regulation of lipid transport	2/180	36/28943	0.0210969627478482	0.0841038037573335	0.0589024264312143	Apoe/Agtr2	2	0.555555555555556
GO:0043537	BP	GO:0043537	negative regulation of blood vessel endothelial cell migration	2/180	36/28943	0.0210969627478482	0.0841038037573335	0.0589024264312143	Gadd45a/Apoe	2	0.555555555555556

GO:0048048	BP	GO:0048048	embryonic eye morphogenesis	2/180	36/28943	0.0210969627478482	0.0841038037573335	0.0589024264312143	Sox11/Aldh1a1	2	0.555555555555556
GO:0019318	BP	GO:0019318	hexose metabolic process	5/180	253/28943	0.0212911727558792	0.0847742660952303	0.059371987340098	Atf3/Igf2/Rbp4/Pdk4/Aldh1a1	5	0.197628458498024
GO:0001909	BP	GO:0001909	leukocyte mediated cytotoxicity	4/180	169/28943	0.0214861983076967	0.0854463344177878	0.0598426730065919	Ccl2/Tyrobp/B2m/Igf2	4	0.236686390532544
GO:0031960	BP	GO:0031960	response to corticosteroid	3/180	95/28943	0.0215713015615461	0.0854716900072452	0.0598604308923938	Sgk1/Ddit4/Agtr2	3	0.315789473684211
GO:0045685	BP	GO:0045685	regulation of glial cell differentiation	3/180	95/28943	0.0215713015615461	0.0854716900072452	0.0598604308923938	Tnfrsf1b/Enpp2/Egr2	3	0.315789473684211
GO:0050672	BP	GO:0050672	negative regulation of lymphocyte proliferation	3/180	95/28943	0.0215713015615461	0.0854716900072452	0.0598604308923938	Sox11/Tyrobp/Laptm5	3	0.315789473684211
GO:0017157	BP	GO:0017157	regulation of exocytosis	5/180	254/28943	0.0216129855768403	0.0855327995428541	0.0599032291935959	S100a10/Itgb2/Vsnl1/Anxa2/Pclo	5	0.196850393700787
GO:0071900	BP	GO:0071900	regulation of protein serine/threonine kinase activity	6/180	347/28943	0.0218072000016031	0.0861267154050259	0.0603191804801613	Gadd45a/Adam8/Sfrp1/Pkib/Apoe/Chordc1	6	0.172910662824208
GO:0007292	BP	GO:0007292	female gamete generation	4/180	170/28943	0.0219003345568925	0.0861267154050259	0.0603191804801613	Cyp51/Spin1/Inhbb/Pla2g4a	4	0.235294117647059
GO:0007044	BP	GO:0007044	cell-substrate junction assembly	3/180	96/28943	0.0221677890568977	0.0861267154050259	0.0603191804801613	S100a10/Enpp2/Nrp1	3	0.3125
GO:0032945	BP	GO:0032945	negative regulation of mononuclear cell proliferation	3/180	96/28943	0.0221677890568977	0.0861267154050259	0.0603191804801613	Sox11/Tyrobp/Laptm5	3	0.3125
GO:2000117	BP	GO:2000117	negative regulation of cysteine-type endopeptidase activity	3/180	96/28943	0.0221677890568977	0.0861267154050259	0.0603191804801613	Cst7/Mmp9/Cst3	3	0.3125

GO:0002724	BP	GO:0002724	regulation of T cell cytokine production	2/180	37/28943	0.0222126008413331	0.0861267154050259	0.0603191804801613	Tnfrsf1b/B2m	2	0.540540540540541
GO:0033687	BP	GO:0033687	osteoblast proliferation	2/180	37/28943	0.0222126008413331	0.0861267154050259	0.0603191804801613	Sfrp1/Npr3	2	0.540540540540541
GO:0033866	BP	GO:0033866	nucleoside bisphosphate biosynthetic process	2/180	37/28943	0.0222126008413331	0.0861267154050259	0.0603191804801613	Papss2/Pdk4	2	0.540540540540541
GO:0034030	BP	GO:0034030	ribonucleoside bisphosphate biosynthetic process	2/180	37/28943	0.0222126008413331	0.0861267154050259	0.0603191804801613	Papss2/Pdk4	2	0.540540540540541
GO:0034033	BP	GO:0034033	purine nucleoside bisphosphate biosynthetic process	2/180	37/28943	0.0222126008413331	0.0861267154050259	0.0603191804801613	Papss2/Pdk4	2	0.540540540540541
GO:0035329	BP	GO:0035329	hippo signaling	2/180	37/28943	0.0222126008413331	0.0861267154050259	0.0603191804801613	Sox11/Mob1b	2	0.540540540540541
GO:0046329	BP	GO:0046329	negative regulation of JNK cascade	2/180	37/28943	0.0222126008413331	0.0861267154050259	0.0603191804801613	Sfrp1/Sfrp4	2	0.540540540540541
GO:0048846	BP	GO:0048846	axon extension involved in axon guidance	2/180	37/28943	0.0222126008413331	0.0861267154050259	0.0603191804801613	Sema7a/Nrp1	2	0.540540540540541
GO:0090279	BP	GO:0090279	regulation of calcium ion import	2/180	37/28943	0.0222126008413331	0.0861267154050259	0.0603191804801613	Ccl2/Lgals3	2	0.540540540540541
GO:0090313	BP	GO:0090313	regulation of protein targeting to membrane	2/180	37/28943	0.0222126008413331	0.0861267154050259	0.0603191804801613	Ccl2/Ank3	2	0.540540540540541
GO:1902284	BP	GO:1902284	neuron projection extension involved in neuron projection guidance	2/180	37/28943	0.0222126008413331	0.0861267154050259	0.0603191804801613	Sema7a/Nrp1	2	0.540540540540541
GO:2000191	BP	GO:2000191	regulation of fatty acid transport	2/180	37/28943	0.0222126008413331	0.0861267154050259	0.0603191804801613	Agtr2/Pla2g4a	2	0.540540540540541

GO:0009267	BP	GO:0009267	cellular response to starvation	4/180	171/28943	0.0223193316651893	0.086437649504782	0.0605369443875903	Atf3/Jun/Inhbb/Pdk4	4	0.233918128654971
GO:0044282	BP	GO:0044282	small molecule catabolic process	6/180	350/28943	0.022630473617885	0.0875385422487547	0.0613079589073094	Bckdhb/Slc27a2/Hibadh/Oxct1/Apoe/Aldh1a1	6	0.171428571428571
GO:0007565	BP	GO:0007565	female pregnancy	4/180	172/28943	0.0227432020665994	0.0876622593265258	0.0613946046444359	Mmp12/Mmp9/Ctsl/Pla2g4a	4	0.232558139534884
GO:0046661	BP	GO:0046661	male sex differentiation	4/180	172/28943	0.0227432020665994	0.0876622593265258	0.0613946046444359	Sfrp1/Fgf9/Fndc3a/Rbp4	4	0.232558139534884
GO:0062013	BP	GO:0062013	positive regulation of small molecule metabolic process	4/180	172/28943	0.0227432020665994	0.0876622593265258	0.0613946046444359	Fdps/Igf2/Nucb2/Pla2g4a	4	0.232558139534884
GO:0001570	BP	GO:0001570	vasculogenesis	3/180	97/28943	0.0227732299645794	0.0876742434924764	0.0614029978017215	Adm/Fgf9/Nrp1	3	0.309278350515464
GO:0042632	BP	GO:0042632	cholesterol homeostasis	3/180	98/28943	0.0233876237807742	0.0899332829444882	0.0629851248778197	Abca1/Apoe/Nus1	3	0.306122448979592
GO:0048705	BP	GO:0048705	skeletal system morphogenesis	5/180	261/28943	0.0239545684856581	0.0918962697492326	0.0643599103296825	Hoxa7/Sox11/Sfrp1/Acan/Sfrp4	5	0.191570881226054
GO:0048864	BP	GO:0048864	stem cell development	3/180	99/28943	0.0240109688537295	0.0918962697492326	0.0643599103296825	Sox11/Sema7a/Nrp1	3	0.303030303030303
GO:0051492	BP	GO:0051492	regulation of stress fiber assembly	3/180	99/28943	0.0240109688537295	0.0918962697492326	0.0643599103296825	S100a10/Nrp1/Lpar1	3	0.303030303030303
GO:0055092	BP	GO:0055092	sterol homeostasis	3/180	99/28943	0.0240109688537295	0.0918962697492326	0.0643599103296825	Abca1/Apoe/Nus1	3	0.303030303030303
GO:0042552	BP	GO:0042552	myelination	4/180	175/28943	0.0240441719916492	0.0919153382356824	0.0643732650184134	Cst7/Itgax/Tnfrsf1b/Egr2	4	0.228571428571429

GO:0015711	BP	GO:0015711	organic anion transport	6/180	356/28943	0.0243393750810577	0.0929347533868756	0.0650872163887851	Slc25a14/Bdnf/Gfap/Agtr2/Slc4a3/Pla2g4a	6	0.168539325842697
GO:0030509	BP	GO:0030509	BMP signaling pathway	4/180	176/28943	0.0244876527411119	0.0933876264534697	0.065404387804298	Sox11/Sfrp1/Sfrp4/Htra1	4	0.227272727272727
GO:0060292	BP	GO:0060292	long-term synaptic depression	2/180	39/28943	0.0245153271776839	0.0933876264534697	0.065404387804298	Mapt/Cbln1	2	0.512820512820513
GO:0007254	BP	GO:0007254	JNK cascade	4/180	177/28943	0.0249360635881964	0.094598767484177	0.0662526151408987	Gadd45a/Sfrp1/Sfrp4/Mdfic	4	0.225988700564972
GO:0019730	BP	GO:0019730	antimicrobial humoral response	4/180	177/28943	0.0249360635881964	0.094598767484177	0.0662526151408987	Adm/Npy/Lgals3/B2m	4	0.225988700564972
GO:0055088	BP	GO:0055088	lipid homeostasis	4/180	177/28943	0.0249360635881964	0.094598767484177	0.0662526151408987	Abca1/Nr1d2/Apoe/Nus1	4	0.225988700564972
GO:0010959	BP	GO:0010959	regulation of metal ion transport	7/180	458/28943	0.0249494446634658	0.094598767484177	0.0662526151408987	Adcyap1/Ccl2/Lgals3/B2m/Sgk1/Ank3/Cd63	7	0.152838427947598
GO:0006885	BP	GO:0006885	regulation of pH	3/180	101/28943	0.025284500537023	0.095424818365103	0.0668311430916983	Car2/Pdk4/Slc4a3	3	0.297029702970297
GO:0007200	BP	GO:0007200	phospholipase C-activating G protein-coupled receptor signaling pathway	3/180	101/28943	0.025284500537023	0.095424818365103	0.0668311430916983	Gna14/Npr3/Lpar1	3	0.297029702970297
GO:0043502	BP	GO:0043502	regulation of muscle adaptation	3/180	101/28943	0.025284500537023	0.095424818365103	0.0668311430916983	Tnfrsf1b/Fdps/Lmna	3	0.297029702970297
GO:0045185	BP	GO:0045185	maintenance of protein location	3/180	101/28943	0.025284500537023	0.095424818365103	0.0668311430916983	Rit2/Tmsb10/Ank3	3	0.297029702970297
GO:0007272	BP	GO:0007272	ensheathment of neurons	4/180	178/28943	0.0253894148271895	0.0955992186036488	0.0669532849778152	Cst7/Itgax/Tnfrsf1b/Egr2	4	0.224719101123595

GO:0008366	BP	GO:0008366	axon ensheathment	4/180	178/28943	0.0253894148271895	0.0955992186036488	0.0669532849778152	Cst7/Itgax/Tnfrsf1b/Egr2	4	0.224719101123595
GO:0002861	BP	GO:0002861	regulation of inflammatory response to antigenic stimulus	2/180	40/28943	0.0257017441288576	0.0961085885507339	0.0673100242035584	Adcyap1/Npy	2	0.5
GO:0019884	BP	GO:0019884	antigen processing and presentation of exogenous antigen	2/180	40/28943	0.0257017441288576	0.0961085885507339	0.0673100242035584	Ctss/B2m	2	0.5
GO:0032094	BP	GO:0032094	response to food	2/180	40/28943	0.0257017441288576	0.0961085885507339	0.0673100242035584	Npy/Nucb2	2	0.5
GO:0042119	BP	GO:0042119	neutrophil activation	2/180	40/28943	0.0257017441288576	0.0961085885507339	0.0673100242035584	Tyrobp/Itgb2	2	0.5
GO:0043267	BP	GO:0043267	negative regulation of potassium ion transport	2/180	40/28943	0.0257017441288576	0.0961085885507339	0.0673100242035584	Adcyap1/Ank3	2	0.5
GO:1905332	BP	GO:1905332	positive regulation of morphogenesis of an epithelium	2/180	40/28943	0.0257017441288576	0.0961085885507339	0.0673100242035584	Itgax/Agtr2	2	0.5
GO:0009408	BP	GO:0009408	response to heat	3/180	102/28943	0.0259346782741013	0.0964260812086163	0.0675323814227078	Ccl2/Mapt/Chordc1	3	0.294117647058824
GO:0031343	BP	GO:0031343	positive regulation of cell killing	3/180	102/28943	0.0259346782741013	0.0964260812086163	0.0675323814227078	Ccl2/Tyrobp/B2m	3	0.294117647058824
GO:0042158	BP	GO:0042158	lipoprotein biosynthetic process	3/180	102/28943	0.0259346782741013	0.0964260812086163	0.0675323814227078	Abca1/Piga/Apoe	3	0.294117647058824
GO:0099565	BP	GO:0099565	chemical synaptic transmission, postsynaptic	3/180	102/28943	0.0259346782741013	0.0964260812086163	0.0675323814227078	Bdnf/Cbln1/Pclo	3	0.294117647058824
GO:0150115	BP	GO:0150115	cell-substrate junction organization	3/180	102/28943	0.0259346782741013	0.0964260812086163	0.0675323814227078	S100a10/Enpp2/Nrp1	3	0.294117647058824

GO:0060541	BP	GO:0060541	respiratory system development	5/180	267/28943	0.0260872164486214	0.0968826271073658	0.0678521251205215	Mmp12/Sox11/Ctsz/Fgf9/Rbp4	5	0.187265917602996
GO:0005996	BP	GO:0005996	monosaccharide metabolic process	5/180	268/28943	0.0264540770541859	0.0978711554706358	0.0685444448087093	Atf3/Igf2/Rbp4/Pdk4/Aldh1a1	5	0.186567164179104
GO:0032886	BP	GO:0032886	regulation of microtubule-based process	5/180	268/28943	0.0264540770541859	0.0978711554706358	0.0685444448087093	Slain1/Sgk1/Mapt/Mark2/Chordc1	5	0.186567164179104
GO:0050730	BP	GO:0050730	regulation of peptidyl-tyrosine phosphorylation	5/180	268/28943	0.0264540770541859	0.0978711554706358	0.0685444448087093	Sfrp1/Enpp2/Itgb2/Igf2/Nrp1	5	0.186567164179104
GO:0021510	BP	GO:0021510	spinal cord development	3/180	103/28943	0.0265937895583398	0.0978711554706358	0.0685444448087093	Sox11/Adarb1/Pbx3	3	0.29126213592233
GO:0030593	BP	GO:0030593	neutrophil chemotaxis	3/180	103/28943	0.0265937895583398	0.0978711554706358	0.0685444448087093	Ccl2/Lgals3/Itgb2	3	0.29126213592233
GO:0050795	BP	GO:0050795	regulation of behavior	3/180	103/28943	0.0265937895583398	0.0978711554706358	0.0685444448087093	Npy/Apoe/Penk	3	0.29126213592233
GO:0062014	BP	GO:0062014	negative regulation of small molecule metabolic process	3/180	103/28943	0.0265937895583398	0.0978711554706358	0.0685444448087093	Ugt1a1/Ddit4/Apoe	3	0.29126213592233
GO:0070664	BP	GO:0070664	negative regulation of leukocyte proliferation	3/180	103/28943	0.0265937895583398	0.0978711554706358	0.0685444448087093	Sox11/Tyrobp/Laptm5	3	0.29126213592233
GO:0034110	BP	GO:0034110	regulation of homotypic cell-cell adhesion	2/180	41/28943	0.0269110880944813	0.0982616748023831	0.0688179465432394	Ank3/Lgals1	2	0.48780487804878
GO:0042572	BP	GO:0042572	retinol metabolic process	2/180	41/28943	0.0269110880944813	0.0982616748023831	0.0688179465432394	Rbp4/Aldh1a1	2	0.48780487804878
GO:0048520	BP	GO:0048520	positive regulation of behavior	2/180	41/28943	0.0269110880944813	0.0982616748023831	0.0688179465432394	Npy/Penk	2	0.48780487804878

GO:0051968	BP	GO:0051968	positive regulation of synaptic transmission, glutamatergic	2/180	41/28943	0.0269110880944813	0.0982616748023831	0.0688179465432394	Adcyap1/Ccl2	2	0.48780487804878
GO:0071827	BP	GO:0071827	plasma lipoprotein particle organization	2/180	41/28943	0.0269110880944813	0.0982616748023831	0.0688179465432394	Abca1/Apoe	2	0.48780487804878
GO:0090181	BP	GO:0090181	regulation of cholesterol metabolic process	2/180	41/28943	0.0269110880944813	0.0982616748023831	0.0688179465432394	Fdps/Apoe	2	0.48780487804878
GO:1904037	BP	GO:1904037	positive regulation of epithelial cell apoptotic process	2/180	41/28943	0.0269110880944813	0.0982616748023831	0.0688179465432394	Ccl2/Sfrp4	2	0.48780487804878
GO:0051153	BP	GO:0051153	regulation of striated muscle cell differentiation	3/180	104/28943	0.027261827278995	0.0993196548631842	0.0695589069982128	Fdps/Bdnf/Efnb2	3	0.288461538461538
GO:0090288	BP	GO:0090288	negative regulation of cellular response to growth factor stimulus	3/180	104/28943	0.027261827278995	0.0993196548631842	0.0695589069982128	Sfrp1/Htra1/Agtr2	3	0.288461538461538
GO:0008544	BP	GO:0008544	epidermis development	6/180	367/28943	0.0276927692828179	0.100776926876132	0.0705795131265703	Sprr1a/Dsp/Sprr1b/Hoxa7/Sfrp4/Ctsl	6	0.163487738419619
GO:0071356	BP	GO:0071356	cellular response to tumor necrosis factor	4/180	183/28943	0.0277306125390529	0.100802014553231	0.0705970833789092	Ccl2/Sfrp1/Laptm5/Gbp3	4	0.218579234972678
GO:0019217	BP	GO:0019217	regulation of fatty acid metabolic process	3/180	105/28943	0.0279387832881641	0.101332535823553	0.0709686360162367	Nucb2/Pdk4/Pla2g4a	3	0.285714285714286
GO:0019731	BP	GO:0019731	antibacterial humoral response	3/180	105/28943	0.0279387832881641	0.101332535823553	0.0709686360162367	Adm/Npy/B2m	3	0.285714285714286
GO:0010464	BP	GO:0010464	regulation of mesenchymal cell proliferation	2/180	42/28943	0.0281430314191748	0.101620679969238	0.0711704388905962	Fgf9/Lmna	2	0.476190476190476
GO:0018149	BP	GO:0018149	peptide cross-linking	2/180	42/28943	0.0281430314191748	0.101620679969238	0.0711704388905962	Sprr1a/Dsp	2	0.476190476190476

GO:0031076	BP	GO:0031076	embryonic camera-type eye development	2/180	42/28943	0.0281430314191748	0.101620679969238	0.0711704388905962	Sox11/Aldh1a1	2	0.476190476190476
GO:0061081	BP	GO:0061081	positive regulation of myeloid leukocyte cytokine production involved in immune response	2/180	42/28943	0.0281430314191748	0.101620679969238	0.0711704388905962	Sema7a/Laptm5	2	0.476190476190476
GO:0071674	BP	GO:0071674	mononuclear cell migration	4/180	184/28943	0.0282138026085591	0.101763405422012	0.0712703972171679	Adam8/Ccl2/Lgals3/Msn	4	0.217391304347826
GO:0030098	BP	GO:0030098	lymphocyte differentiation	7/180	471/28943	0.0284528198430133	0.102511984766255	0.0717946676756054	Adam8/Sfrp1/B2m/Ctla2a/Laptm5/Ctsl/Lgals1	7	0.148619957537155
GO:0002705	BP	GO:0002705	positive regulation of leukocyte mediated immunity	4/180	185/28943	0.0287019953036985	0.103295468181377	0.0723433833359867	Ccl2/Tyrobp/B2m/Itgb2	4	0.216216216216216
GO:0032680	BP	GO:0032680	regulation of tumor necrosis factor production	4/180	186/28943	0.0291951981254852	0.104755844567444	0.0733661636241463	Ccl2/Tyrobp/Ifih1/Ccr5	4	0.21505376344086
GO:0001657	BP	GO:0001657	ureteric bud development	3/180	107/28943	0.0293194125007707	0.104755844567444	0.0733661636241463	Sfrp1/Bdnf/Agtr2	3	0.280373831775701
GO:0006821	BP	GO:0006821	chloride transport	3/180	107/28943	0.0293194125007707	0.104755844567444	0.0733661636241463	Car2/Clic1/Clca1	3	0.280373831775701
GO:0007616	BP	GO:0007616	long-term memory	2/180	43/28943	0.029397249596145	0.104755844567444	0.0733661636241463	Sgk1/Apoe	2	0.465116279069767
GO:0010874	BP	GO:0010874	regulation of cholesterol efflux	2/180	43/28943	0.029397249596145	0.104755844567444	0.0733661636241463	Abca1/Apoe	2	0.465116279069767
GO:0032228	BP	GO:0032228	regulation of synaptic transmission, GABAergic	2/180	43/28943	0.029397249596145	0.104755844567444	0.0733661636241463	Car2/Bdnf	2	0.465116279069767
GO:0032924	BP	GO:0032924	activin receptor signaling pathway	2/180	43/28943	0.029397249596145	0.104755844567444	0.0733661636241463	Fgf9/Inhbb	2	0.465116279069767

GO:0045880	BP	GO:0045880	positive regulation of smoothened signaling pathway	2/180	43/28943	0.029397249596145	0.104755844567444	0.0733661636241463	Sfrp1/Fgf9	2	0.465116279069767
GO:1990748	BP	GO:1990748	cellular detoxification	2/180	43/28943	0.029397249596145	0.104755844567444	0.0733661636241463	Prdx6/Aldh1a1	2	0.465116279069767
GO:0002833	BP	GO:0002833	positive regulation of response to biotic stimulus	4/180	187/28943	0.0296934182313629	0.105235542088737	0.0737021216509518	Mmp12/Adam8/Tyrobp/Ankrd17	4	0.213903743315508
GO:0007612	BP	GO:0007612	learning	4/180	187/28943	0.0296934182313629	0.105235542088737	0.0737021216509518	Jun/Specc1/Sgk1/Bdnf	4	0.213903743315508
GO:0032388	BP	GO:0032388	positive regulation of intracellular transport	4/180	187/28943	0.0296934182313629	0.105235542088737	0.0737021216509518	Ccl2/Msn/Anxa2/Ank3	4	0.213903743315508
GO:0043409	BP	GO:0043409	negative regulation of MAPK cascade	4/180	187/28943	0.0296934182313629	0.105235542088737	0.0737021216509518	Atf3/Sfrp1/Sfrp4/Apoe	4	0.213903743315508
GO:0048592	BP	GO:0048592	eye morphogenesis	4/180	187/28943	0.0296934182313629	0.105235542088737	0.0737021216509518	Sox11/Bdnf/Rbp4/Aldh1a1	4	0.213903743315508
GO:0006650	BP	GO:0006650	glycerophospholipid metabolic process	5/180	277/28943	0.0299046451871686	0.1058689449724	0.0741457278267669	Piga/Enpp2/Sacm1l/Prdx6/Pla2g4a	5	0.180505415162455
GO:0090263	BP	GO:0090263	positive regulation of canonical Wnt signaling pathway	3/180	108/28943	0.0300230643810891	0.106172769477967	0.0743585125022231	Sfrp1/Fgf9/Sfrp4	3	0.277777777777778
GO:0016331	BP	GO:0016331	morphogenesis of embryonic epithelium	4/180	188/28943	0.0301966624365532	0.106670856351251	0.0747073495832982	Sox11/Adm/Sfrp1/Aldh1a1	4	0.212765957446809
GO:0043010	BP	GO:0043010	camera-type eye development	6/180	376/28943	0.0306538403067327	0.107525051495618	0.0753055884785818	Jun/Sox11/Nrp1/Mab21l2/Rbp4/Aldh1a1	6	0.159574468085106
GO:0035886	BP	GO:0035886	vascular associated smooth muscle cell differentiation	2/180	44/28943	0.0306734212410571	0.107525051495618	0.0753055884785818	Adm/Fgf9	2	0.454545454545455

GO:0046320	BP	GO:0046320	regulation of fatty acid oxidation	2/180	44/28943	0.0306734212410571	0.107525051495618	0.0753055884785818	Nucb2/Pdk4	2	0.454545454545455
GO:0097028	BP	GO:0097028	dendritic cell differentiation	2/180	44/28943	0.0306734212410571	0.107525051495618	0.0753055884785818	Tmem176a/Tmem176b	2	0.454545454545455
GO:0099084	BP	GO:0099084	postsynaptic specialization organization	2/180	44/28943	0.0306734212410571	0.107525051495618	0.0753055884785818	Gap43/Cbln1	2	0.454545454545455
GO:0030534	BP	GO:0030534	adult behavior	4/180	189/28943	0.0307049372154344	0.107525051495618	0.0753055884785818	Npy/Bdnf/Pbx3/Mapt	4	0.211640211640212
GO:0035967	BP	GO:0035967	cellular response to topologically incorrect protein	3/180	109/28943	0.0307355919381087	0.107525051495618	0.0753055884785818	Atf3/Atf6/Hspb8	3	0.275229357798165
GO:0048661	BP	GO:0048661	positive regulation of smooth muscle cell proliferation	3/180	109/28943	0.0307355919381087	0.107525051495618	0.0753055884785818	Jun/Mmp9/Fgf9	3	0.275229357798165
GO:0072676	BP	GO:0072676	lymphocyte migration	3/180	109/28943	0.0307355919381087	0.107525051495618	0.0753055884785818	Adam8/Ccl2/Msn	3	0.275229357798165
GO:0044703	BP	GO:0044703	multi-organism reproductive process	4/180	190/28943	0.0312182487029496	0.109096390585308	0.0764060819282717	Mmp12/Mmp9/Ctsl/Pla2g4a	4	0.210526315789474
GO:0010595	BP	GO:0010595	positive regulation of endothelial cell migration	3/180	110/28943	0.0314569820998935	0.109344066914998	0.0765795430101323	Igf2/Nrp1/Nus1	3	0.272727272727273
GO:0046916	BP	GO:0046916	cellular transition metal ion homeostasis	3/180	110/28943	0.0314569820998935	0.109344066914998	0.0765795430101323	Mt2/Abcb7/Mt1	3	0.272727272727273
GO:0072163	BP	GO:0072163	mesonephric epithelium development	3/180	110/28943	0.0314569820998935	0.109344066914998	0.0765795430101323	Sfrp1/Bdnf/Agtr2	3	0.272727272727273
GO:0072164	BP	GO:0072164	mesonephric tubule development	3/180	110/28943	0.0314569820998935	0.109344066914998	0.0765795430101323	Sfrp1/Bdnf/Agtr2	3	0.272727272727273

GO:2000243	BP	GO:2000243	positive regulation of reproductive process	3/180	110/28943	0.0314569820998935	0.109344066914998	0.0765795430101323	Cyp51/Fgf9/Inhbb	3	0.272727272727273
GO:0042594	BP	GO:0042594	response to starvation	4/180	191/28943	0.0317366026960427	0.110198416824106	0.0771778902955051	Atf3/Jun/Inhbb/Pdk4	4	0.209424083769634
GO:0048863	BP	GO:0048863	stem cell differentiation	5/180	282/28943	0.0319387086268632	0.110307510393334	0.077254294401485	Hoxa7/Sox11/Sfrp1/Sema7a/Nrp1	5	0.177304964539007
GO:0010765	BP	GO:0010765	positive regulation of sodium ion transport	2/180	45/28943	0.031971228066106	0.110307510393334	0.077254294401485	Sgk1/Ank3	2	0.444444444444444
GO:0010935	BP	GO:0010935	regulation of macrophage cytokine production	2/180	45/28943	0.031971228066106	0.110307510393334	0.077254294401485	Sema7a/Laptm5	2	0.444444444444444
GO:0016925	BP	GO:0016925	protein sumoylation	2/180	45/28943	0.031971228066106	0.110307510393334	0.077254294401485	Ifih1/Egr2	2	0.444444444444444
GO:0031952	BP	GO:0031952	regulation of protein autophosphorylation	2/180	45/28943	0.031971228066106	0.110307510393334	0.077254294401485	Jun/Mob1b	2	0.444444444444444
GO:0071825	BP	GO:0071825	protein-lipid complex subunit organization	2/180	45/28943	0.031971228066106	0.110307510393334	0.077254294401485	Abca1/Apoe	2	0.444444444444444
GO:0110020	BP	GO:0110020	regulation of actomyosin structure organization	3/180	111/28943	0.0321872208606408	0.110935215177891	0.0776939098914049	S100a10/Nrp1/Lpar1	3	0.27027027027027
GO:0046486	BP	GO:0046486	glycerolipid metabolic process	6/180	382/28943	0.0327389794268759	0.112717606758282	0.0789422147747191	Piga/Enpp2/Apoe/Sacm1l/Prdx6/Pla2g4a	6	0.157068062827225
GO:0032640	BP	GO:0032640	tumor necrosis factor production	4/180	193/28943	0.0327884597055568	0.112768757403377	0.0789780383281738	Ccl2/Tyrobp/Ifih1/Ccr5	4	0.207253886010363
GO:0032606	BP	GO:0032606	type I interferon production	3/180	112/28943	0.0329262932971991	0.113004148860883	0.0791428956519667	Mmp12/Irf9/Ifih1	3	0.267857142857143

GO:1905477	BP	GO:1905477	positive regulation of protein localization to membrane	3/180	112/28943	0.0329262932971991	0.113004148860883	0.0791428956519667	Ccl2/Lgals3/Ank3	3	0.267857142857143
GO:0002691	BP	GO:0002691	regulation of cellular extravasation	2/180	46/28943	0.0332903548542868	0.113893577479424	0.0797658104480755	Adam8/Ccl2	2	0.434782608695652
GO:0036230	BP	GO:0036230	granulocyte activation	2/180	46/28943	0.0332903548542868	0.113893577479424	0.0797658104480755	Tyrobp/Itgb2	2	0.434782608695652
GO:0055023	BP	GO:0055023	positive regulation of cardiac muscle tissue growth	2/180	46/28943	0.0332903548542868	0.113893577479424	0.0797658104480755	Fgf9/Fdps	2	0.434782608695652
GO:0001823	BP	GO:0001823	mesonephros development	3/180	113/28943	0.0336741835853927	0.114844833442538	0.0804320262656886	Sfrp1/Bdnf/Agtr2	3	0.265486725663717
GO:0002027	BP	GO:0002027	regulation of heart rate	3/180	113/28943	0.0336741835853927	0.114844833442538	0.0804320262656886	Dsp/Adm/Agtr2	3	0.265486725663717
GO:0045639	BP	GO:0045639	positive regulation of myeloid cell differentiation	3/180	113/28943	0.0336741835853927	0.114844833442538	0.0804320262656886	Jun/Tyrobp/Car2	3	0.265486725663717
GO:0009101	BP	GO:0009101	glycoprotein biosynthetic process	5/180	287/28943	0.034057491611163	0.11603059642004	0.0812624799839134	St3gal5/Acan/Serpina1b/Serpina1a/Nus1	5	0.174216027874564
GO:0032496	BP	GO:0032496	response to lipopolysaccharide	6/180	386/28943	0.0341791584276795	0.116323426331194	0.0814675645523638	Abca1/Ccl2/Adm/Cd68/Tnfrsf1b/B2m	6	0.155440414507772
GO:0019722	BP	GO:0019722	calcium-mediated signaling	4/180	196/28943	0.0344041896650436	0.116399901017635	0.0815211238967775	Lat2/Rit2/Mapt/Ccr5	4	0.204081632653061
GO:2001257	BP	GO:2001257	regulation of cation channel activity	4/180	196/28943	0.0344041896650436	0.116399901017635	0.0815211238967775	Ctss/Ccl2/Mmp9/Ank3	4	0.204081632653061
GO:0043266	BP	GO:0043266	regulation of potassium ion transport	3/180	114/28943	0.0344308750161533	0.116399901017635	0.0815211238967775	Adcyap1/Ank3/Cd63	3	0.263157894736842

GO:0035774	BP	GO:0035774	positive regulation of insulin secretion involved in cellular response to glucose stimulus	2/180	47/28943	0.0346304894338618	0.116399901017635	0.0815211238967775	Vsnl1/Oxct1	2	0.425531914893617
GO:0042417	BP	GO:0042417	dopamine metabolic process	2/180	47/28943	0.0346304894338618	0.116399901017635	0.0815211238967775	Npy/Agtr2	2	0.425531914893617
GO:0042789	BP	GO:0042789	mRNA transcription by RNA polymerase II	2/180	47/28943	0.0346304894338618	0.116399901017635	0.0815211238967775	S100a10/Anxa2	2	0.425531914893617
GO:0043114	BP	GO:0043114	regulation of vascular permeability	2/180	47/28943	0.0346304894338618	0.116399901017635	0.0815211238967775	Adm/Apoe	2	0.425531914893617
GO:0043277	BP	GO:0043277	apoptotic cell clearance	2/180	47/28943	0.0346304894338618	0.116399901017635	0.0815211238967775	Ccl2/Tyrobp	2	0.425531914893617
GO:0048246	BP	GO:0048246	macrophage chemotaxis	2/180	47/28943	0.0346304894338618	0.116399901017635	0.0815211238967775	Ccl2/Lgals3	2	0.425531914893617
GO:0050685	BP	GO:0050685	positive regulation of mRNA processing	2/180	47/28943	0.0346304894338618	0.116399901017635	0.0815211238967775	Adarb1/Prdx6	2	0.425531914893617
GO:0060135	BP	GO:0060135	maternal process involved in female pregnancy	2/180	47/28943	0.0346304894338618	0.116399901017635	0.0815211238967775	Ctsl/Pla2g4a	2	0.425531914893617
GO:0120163	BP	GO:0120163	negative regulation of cold-induced thermogenesis	2/180	47/28943	0.0346304894338618	0.116399901017635	0.0815211238967775	Npr3/Aldh1a1	2	0.425531914893617
GO:0007033	BP	GO:0007033	vacuole organization	4/180	197/28943	0.0349529016873469	0.117362475047102	0.0821952663662299	Abca1/Stbd1/Ctsd/Laptm5	4	0.203045685279188
GO:0032231	BP	GO:0032231	regulation of actin filament bundle assembly	3/180	115/28943	0.0351963500114623	0.118058199780981	0.0826825198925024	S100a10/Nrp1/Lpar1	3	0.260869565217391
GO:0046942	BP	GO:0046942	carboxylic acid transport	5/180	290/28943	0.0353697437158431	0.118517752348252	0.0830043693223218	Slc25a14/Bdnf/Gfap/Agtr2/Pla2g4a	5	0.172413793103448

GO:0006986	BP	GO:0006986	response to unfolded protein	3/180	116/28943	0.0359705901401041	0.118888172293004	0.0832637943729102	Atf3/Atf6/Hspb8	3	0.258620689655172
GO:0007589	BP	GO:0007589	body fluid secretion	3/180	116/28943	0.0359705901401041	0.118888172293004	0.0832637943729102	Csn3/Anxa2/Npr3	3	0.258620689655172
GO:0030038	BP	GO:0030038	contractile actin filament bundle assembly	3/180	116/28943	0.0359705901401041	0.118888172293004	0.0832637943729102	S100a10/Nrp1/Lpar1	3	0.258620689655172
GO:0043149	BP	GO:0043149	stress fiber assembly	3/180	116/28943	0.0359705901401041	0.118888172293004	0.0832637943729102	S100a10/Nrp1/Lpar1	3	0.258620689655172
GO:1904035	BP	GO:1904035	regulation of epithelial cell apoptotic process	3/180	116/28943	0.0359705901401041	0.118888172293004	0.0832637943729102	Ccl2/Scg2/Sfrp4	3	0.258620689655172
GO:0002369	BP	GO:0002369	T cell cytokine production	2/180	48/28943	0.0359913226530249	0.118888172293004	0.0832637943729102	Tnfrsf1b/B2m	2	0.416666666666667
GO:0002686	BP	GO:0002686	negative regulation of leukocyte migration	2/180	48/28943	0.0359913226530249	0.118888172293004	0.0832637943729102	Ccl2/Hoxa7	2	0.416666666666667
GO:0032873	BP	GO:0032873	negative regulation of stress-activated MAPK cascade	2/180	48/28943	0.0359913226530249	0.118888172293004	0.0832637943729102	Sfrp1/Sfrp4	2	0.416666666666667
GO:0042304	BP	GO:0042304	regulation of fatty acid biosynthetic process	2/180	48/28943	0.0359913226530249	0.118888172293004	0.0832637943729102	Pdk4/Pla2g4a	2	0.416666666666667
GO:0045761	BP	GO:0045761	regulation of adenylate cyclase activity	2/180	48/28943	0.0359913226530249	0.118888172293004	0.0832637943729102	Adcyap1/Npr3	2	0.416666666666667
GO:0045923	BP	GO:0045923	positive regulation of fatty acid metabolic process	2/180	48/28943	0.0359913226530249	0.118888172293004	0.0832637943729102	Nucb2/Pla2g4a	2	0.416666666666667
GO:0050856	BP	GO:0050856	regulation of T cell receptor signaling pathway	2/180	48/28943	0.0359913226530249	0.118888172293004	0.0832637943729102	Lgals3/Laptm5	2	0.416666666666667

GO:0051972	BP	GO:0051972	regulation of telomerase activity	2/180	48/28943	0.0359913226530249	0.118888172293004	0.0832637943729102	Pkib/Parm1	2	0.416666666666667
GO:0070303	BP	GO:0070303	negative regulation of stress-activated protein kinase signaling cascade	2/180	48/28943	0.0359913226530249	0.118888172293004	0.0832637943729102	Sfrp1/Sfrp4	2	0.416666666666667
GO:0006109	BP	GO:0006109	regulation of carbohydrate metabolic process	4/180	199/28943	0.036065550119457	0.118892203177198	0.0832666174183501	Ugt1a1/Igf2/Ddit4/Pdk4	4	0.201005025125628
GO:0008016	BP	GO:0008016	regulation of heart contraction	4/180	199/28943	0.036065550119457	0.118892203177198	0.0832666174183501	Dsp/Adm/Agtr2/Slc4a3	4	0.201005025125628
GO:0001764	BP	GO:0001764	neuron migration	4/180	200/28943	0.0366294925010851	0.120143259895301	0.0841427998620358	Nrp1/Ddit4/Mapt/Mark2	4	0.2
GO:0006486	BP	GO:0006486	protein glycosylation	4/180	200/28943	0.0366294925010851	0.120143259895301	0.0841427998620358	St3gal5/Serpina1b/Serpina1a/Nus1	4	0.2
GO:0030308	BP	GO:0030308	negative regulation of cell growth	4/180	200/28943	0.0366294925010851	0.120143259895301	0.0841427998620358	Sfrp1/Sema7a/Nrp1/Ccr5	4	0.2
GO:0043413	BP	GO:0043413	macromolecule glycosylation	4/180	200/28943	0.0366294925010851	0.120143259895301	0.0841427998620358	St3gal5/Serpina1b/Serpina1a/Nus1	4	0.2
GO:0045732	BP	GO:0045732	positive regulation of protein catabolic process	4/180	200/28943	0.0366294925010851	0.120143259895301	0.0841427998620358	Adam8/Msn/Tnfrsf1b/Apoe	4	0.2
GO:0019218	BP	GO:0019218	regulation of steroid metabolic process	3/180	117/28943	0.0367535761332331	0.120428971293702	0.0843428989523626	Fdps/Igf2/Apoe	3	0.256410256410256
GO:0010543	BP	GO:0010543	regulation of platelet activation	2/180	49/28943	0.0373725483547593	0.121722389991451	0.0852487497734351	Apoe/Pla2g4a	2	0.408163265306122
GO:0010677	BP	GO:0010677	negative regulation of cellular carbohydrate metabolic process	2/180	49/28943	0.0373725483547593	0.121722389991451	0.0852487497734351	Ugt1a1/Ddit4	2	0.408163265306122

GO:0010837	BP	GO:0010837	regulation of keratinocyte proliferation	2/180	49/28943	0.0373725483547593	0.121722389991451	0.0852487497734351	Ctsl/Efnb2	2	0.408163265306122
GO:0060421	BP	GO:0060421	positive regulation of heart growth	2/180	49/28943	0.0373725483547593	0.121722389991451	0.0852487497734351	Fgf9/Fdps	2	0.408163265306122
GO:0070232	BP	GO:0070232	regulation of T cell apoptotic process	2/180	49/28943	0.0373725483547593	0.121722389991451	0.0852487497734351	Adam8/Lgals3	2	0.408163265306122
GO:0071526	BP	GO:0071526	semaphorin-plexin signaling pathway	2/180	49/28943	0.0373725483547593	0.121722389991451	0.0852487497734351	Sema7a/Nrp1	2	0.408163265306122
GO:0002832	BP	GO:0002832	negative regulation of response to biotic stimulus	3/180	118/28943	0.0375452878997563	0.121797811443731	0.0853015714812035	Mmp12/Igf2/Htra1	3	0.254237288135593
GO:0019395	BP	GO:0019395	fatty acid oxidation	3/180	118/28943	0.0375452878997563	0.121797811443731	0.0853015714812035	Slc27a2/Nucb2/Pdk4	3	0.254237288135593
GO:0035282	BP	GO:0035282	segmentation	3/180	118/28943	0.0375452878997563	0.121797811443731	0.0853015714812035	Sfrp1/Nrp1/Egr2	3	0.254237288135593
GO:0043401	BP	GO:0043401	steroid hormone mediated signaling pathway	3/180	118/28943	0.0375452878997563	0.121797811443731	0.0853015714812035	Sfrp1/Sgk1/Bdnf	3	0.254237288135593
GO:0007179	BP	GO:0007179	transforming growth factor beta receptor signaling pathway	4/180	202/28943	0.0377726261059214	0.122291693068575	0.0856474634001587	Jun/Ccl2/Nrep/Htra1	4	0.198019801980198
GO:0034612	BP	GO:0034612	response to tumor necrosis factor	4/180	202/28943	0.0377726261059214	0.122291693068575	0.0856474634001587	Ccl2/Sfrp1/Laptm5/Gbp3	4	0.198019801980198
GO:0008585	BP	GO:0008585	female gonad development	3/180	119/28943	0.0383457045415328	0.123552801395919	0.0865306855275139	Adcyap1/Sfrp1/Pla2g4a	3	0.252100840336134
GO:0008637	BP	GO:0008637	apoptotic mitochondrial changes	3/180	119/28943	0.0383457045415328	0.123552801395919	0.0865306855275139	Jun/Mmp9/Lmna	3	0.252100840336134

GO:0045666	BP	GO:0045666	positive regulation of neuron differentiation	3/180	119/28943	0.0383457045415328	0.123552801395919	0.0865306855275139	Sox11/Bdnf/Ccr5	3	0.252100840336134
GO:2000106	BP	GO:2000106	regulation of leukocyte apoptotic process	3/180	119/28943	0.0383457045415328	0.123552801395919	0.0865306855275139	Adam8/Lgals3/Ccr5	3	0.252100840336134
GO:0006575	BP	GO:0006575	cellular modified amino acid metabolic process	4/180	203/28943	0.0383518213728199	0.123552801395919	0.0865306855275139	Ckmt1/Clic1/Gpx3/Ctsl	4	0.197044334975369
GO:0002753	BP	GO:0002753	cytoplasmic pattern recognition receptor signaling pathway	2/180	50/28943	0.0387738633518891	0.124297709583763	0.0870523848772728	Ankrd17/Ifih1	2	0.4
GO:0003254	BP	GO:0003254	regulation of membrane depolarization	2/180	50/28943	0.0387738633518891	0.124297709583763	0.0870523848772728	B2m/Ank3	2	0.4
GO:0034332	BP	GO:0034332	adherens junction organization	2/180	50/28943	0.0387738633518891	0.124297709583763	0.0870523848772728	Dsp/Efnb2	2	0.4
GO:0097237	BP	GO:0097237	cellular response to toxic substance	2/180	50/28943	0.0387738633518891	0.124297709583763	0.0870523848772728	Prdx6/Aldh1a1	2	0.4
GO:1902930	BP	GO:1902930	regulation of alcohol biosynthetic process	2/180	50/28943	0.0387738633518891	0.124297709583763	0.0870523848772728	Fdps/Apoe	2	0.4
GO:0032872	BP	GO:0032872	regulation of stress-activated MAPK cascade	4/180	204/28943	0.0389361044489419	0.12457258564853	0.0872448953995006	Gadd45a/Sfrp1/Sfrp4/Mdfic	4	0.196078431372549
GO:0044706	BP	GO:0044706	multi-multicellular organism process	4/180	204/28943	0.0389361044489419	0.12457258564853	0.0872448953995006	Mmp12/Mmp9/Ctsl/Pla2g4a	4	0.196078431372549
GO:0046394	BP	GO:0046394	carboxylic acid biosynthetic process	5/180	298/28943	0.0390206039976229	0.124720419254424	0.0873484312409988	Elovl6/Slc27a2/Pdk4/Aldh1a1/Pla2g4a	5	0.167785234899329
GO:0002275	BP	GO:0002275	myeloid cell activation involved in immune response	3/180	120/28943	0.0391548043683924	0.124904209429828	0.0874771494059553	Tyrobp/Itgb2/Lat2	3	0.25

GO:0008306	BP	GO:0008306	associative learning	3/180	120/28943	0.0391548043683924	0.124904209429828	0.0874771494059553	Specc1/Sgk1/Bdnf	3	0.25
GO:0016053	BP	GO:0016053	organic acid biosynthetic process	5/180	299/28943	0.0394925431213476	0.125858329692983	0.0881453712472553	Elovl6/Slc27a2/Pdk4/Aldh1a1/Pla2g4a	5	0.167224080267559
GO:0060070	BP	GO:0060070	canonical Wnt signaling pathway	5/180	300/28943	0.0399679590204482	0.127139300704647	0.0890425042829112	Sfrp1/Kpna1/Fgf9/Sfrp4/Apoe	5	0.166666666666667
GO:0090100	BP	GO:0090100	positive regulation of transmembrane receptor protein serine/threonine kinase signaling pathway	3/180	121/28943	0.0399725649129746	0.127139300704647	0.0890425042829112	Sox11/Fgf9/Inhbb	3	0.247933884297521
GO:0032271	BP	GO:0032271	regulation of protein polymerization	4/180	206/28943	0.0401199386359694	0.127225470193745	0.0891028534201137	Slain1/Sgk1/Tmsb10/Mapt	4	0.194174757281553
GO:0021879	BP	GO:0021879	forebrain neuron differentiation	2/180	51/28943	0.0401949674023222	0.127225470193745	0.0891028534201137	B2m/Nrp1	2	0.392156862745098
GO:0030195	BP	GO:0030195	negative regulation of blood coagulation	2/180	51/28943	0.0401949674023222	0.127225470193745	0.0891028534201137	Apoe/Anxa2	2	0.392156862745098
GO:0032309	BP	GO:0032309	icosanoid secretion	2/180	51/28943	0.0401949674023222	0.127225470193745	0.0891028534201137	Agtr2/Pla2g4a	2	0.392156862745098
GO:0045740	BP	GO:0045740	positive regulation of DNA replication	2/180	51/28943	0.0401949674023222	0.127225470193745	0.0891028534201137	Jun/Cst3	2	0.392156862745098
GO:0070302	BP	GO:0070302	regulation of stress-activated protein kinase signaling cascade	4/180	207/28943	0.0407194912685259	0.128760566079212	0.0901779638006086	Gadd45a/Sfrp1/Sfrp4/Mdfic	4	0.193236714975845
GO:0021782	BP	GO:0021782	glial cell development	3/180	122/28943	0.040798962945391	0.128761843326685	0.090178858325849	C1qa/Sox11/Gfap	3	0.245901639344262
GO:0046545	BP	GO:0046545	development of primary female sexual characteristics	3/180	122/28943	0.040798962945391	0.128761843326685	0.090178858325849	Adcyap1/Sfrp1/Pla2g4a	3	0.245901639344262

GO:0008037	BP	GO:0008037	cell recognition	5/180	302/28943	0.0409292364639661	0.129047941106619	0.0903792280199873	Lgals3/Msn/Gap43/Nrp1/Bdnf	5	0.165562913907285
GO:0099173	BP	GO:0099173	postsynapse organization	4/180	208/28943	0.0413241347572329	0.130016327221347	0.0910574410059816	Gap43/Nrp1/Apoe/Cbln1	4	0.192307692307692
GO:0001676	BP	GO:0001676	long-chain fatty acid metabolic process	3/180	123/28943	0.0416339744877113	0.130016327221347	0.0910574410059816	Elovl6/Slc27a2/Pla2g4a	3	0.24390243902439
GO:1902414	BP	GO:1902414	protein localization to cell junction	3/180	123/28943	0.0416339744877113	0.130016327221347	0.0910574410059816	Dsp/Mapt/Pclo	3	0.24390243902439
GO:0010934	BP	GO:0010934	macrophage cytokine production	2/180	52/28943	0.041635563184484	0.130016327221347	0.0910574410059816	Sema7a/Laptm5	2	0.384615384615385
GO:0019083	BP	GO:0019083	viral transcription	2/180	52/28943	0.041635563184484	0.130016327221347	0.0910574410059816	Jun/Mdfic	2	0.384615384615385
GO:0046596	BP	GO:0046596	regulation of viral entry into host cell	2/180	52/28943	0.041635563184484	0.130016327221347	0.0910574410059816	Lgals1/Trim25	2	0.384615384615385
GO:0060043	BP	GO:0060043	regulation of cardiac muscle cell proliferation	2/180	52/28943	0.041635563184484	0.130016327221347	0.0910574410059816	Fgf9/Rbp4	2	0.384615384615385
GO:1900047	BP	GO:1900047	negative regulation of hemostasis	2/180	52/28943	0.041635563184484	0.130016327221347	0.0910574410059816	Apoe/Anxa2	2	0.384615384615385
GO:1904407	BP	GO:1904407	positive regulation of nitric oxide metabolic process	2/180	52/28943	0.041635563184484	0.130016327221347	0.0910574410059816	Npy/Itgb2	2	0.384615384615385
GO:2001258	BP	GO:2001258	negative regulation of cation channel activity	2/180	52/28943	0.041635563184484	0.130016327221347	0.0910574410059816	Mmp9/Ank3	2	0.384615384615385
GO:0060485	BP	GO:0060485	mesenchyme development	5/180	304/28943	0.0419044655360901	0.130605592584733	0.091470135297615	Sox11/Sfrp1/Fgf9/Sema7a/Nrp1	5	0.164473684210526

GO:0071222	BP	GO:0071222	cellular response to lipopolysaccharide	5/180	304/28943	0.0419044655360901	0.130605592584733	0.091470135297615	Abca1/Ccl2/Cd68/Tnfrsf1b/B2m	5	0.164473684210526
GO:0002237	BP	GO:0002237	response to molecule of bacterial origin	6/180	407/28943	0.0424107511399865	0.132057185911029	0.0924867643356655	Abca1/Ccl2/Adm/Cd68/Tnfrsf1b/B2m	6	0.147420147420147
GO:0021953	BP	GO:0021953	central nervous system neuron differentiation	4/180	210/28943	0.042548693945365	0.132107813327029	0.0925222214435864	B2m/Adarb1/Nrp1/Cbln1	4	0.19047619047619
GO:1902107	BP	GO:1902107	positive regulation of leukocyte differentiation	4/180	210/28943	0.042548693945365	0.132107813327029	0.0925222214435864	Jun/Adam8/Tyrobp/Car2	4	0.19047619047619
GO:1903708	BP	GO:1903708	positive regulation of hemopoiesis	4/180	210/28943	0.042548693945365	0.132107813327029	0.0925222214435864	Jun/Adam8/Tyrobp/Car2	4	0.19047619047619
GO:0002042	BP	GO:0002042	cell migration involved in sprouting angiogenesis	2/180	53/28943	0.0430953562729396	0.133170375124255	0.0932663907355039	Nrp1/Efnb2	2	0.377358490566038
GO:0009299	BP	GO:0009299	mRNA transcription	2/180	53/28943	0.0430953562729396	0.133170375124255	0.0932663907355039	S100a10/Anxa2	2	0.377358490566038
GO:0034605	BP	GO:0034605	cellular response to heat	2/180	53/28943	0.0430953562729396	0.133170375124255	0.0932663907355039	Mapt/Chordc1	2	0.377358490566038
GO:0043392	BP	GO:0043392	negative regulation of DNA binding	2/180	53/28943	0.0430953562729396	0.133170375124255	0.0932663907355039	Jun/Sox11	2	0.377358490566038
GO:0090199	BP	GO:0090199	regulation of release of cytochrome c from mitochondria	2/180	53/28943	0.0430953562729396	0.133170375124255	0.0932663907355039	Mmp9/Lmna	2	0.377358490566038
GO:0031109	BP	GO:0031109	microtubule polymerization or depolymerization	3/180	125/28943	0.0433297385358332	0.133767733091193	0.0936847527135451	Slain1/Sgk1/Mapt	3	0.24
GO:0034440	BP	GO:0034440	lipid oxidation	3/180	126/28943	0.0441904394735154	0.136295702050416	0.0954552256171329	Slc27a2/Nucb2/Pdk4	3	0.238095238095238

GO:0035272	BP	GO:0035272	exocrine system development	2/180	54/28943	0.0445740551142035	0.136831006132857	0.0958301279155504	Igf2/Nrp1	2	0.37037037037037
GO:0045912	BP	GO:0045912	negative regulation of carbohydrate metabolic process	2/180	54/28943	0.0445740551142035	0.136831006132857	0.0958301279155504	Ugt1a1/Ddit4	2	0.37037037037037
GO:0048247	BP	GO:0048247	lymphocyte chemotaxis	2/180	54/28943	0.0445740551142035	0.136831006132857	0.0958301279155504	Adam8/Ccl2	2	0.37037037037037
GO:0071709	BP	GO:0071709	membrane assembly	2/180	54/28943	0.0445740551142035	0.136831006132857	0.0958301279155504	S100a10/Ank3	2	0.37037037037037
GO:2000351	BP	GO:2000351	regulation of endothelial cell apoptotic process	2/180	54/28943	0.0445740551142035	0.136831006132857	0.0958301279155504	Ccl2/Scg2	2	0.37037037037037
GO:0018958	BP	GO:0018958	phenol-containing compound metabolic process	3/180	127/28943	0.0450596508126322	0.138061413637576	0.0966918486022418	Npy/Ctsl/Agtr2	3	0.236220472440945
GO:0051101	BP	GO:0051101	regulation of DNA binding	3/180	127/28943	0.0450596508126322	0.138061413637576	0.0966918486022418	Jun/Sox11/Mmp9	3	0.236220472440945
GO:0043500	BP	GO:0043500	muscle adaptation	3/180	128/28943	0.0459373450463087	0.140486321892796	0.0983901425405002	Tnfrsf1b/Fdps/Lmna	3	0.234375
GO:0048565	BP	GO:0048565	digestive tract development	3/180	128/28943	0.0459373450463087	0.140486321892796	0.0983901425405002	Sox11/Sfrp1/Fgf9	3	0.234375
GO:0001961	BP	GO:0001961	positive regulation of cytokine-mediated signaling pathway	2/180	55/28943	0.0460713710027363	0.140500426363214	0.098400020661275	Mmp12/Laptm5	2	0.363636363636364
GO:0010823	BP	GO:0010823	negative regulation of mitochondrion organization	2/180	55/28943	0.0460713710027363	0.140500426363214	0.098400020661275	Lmna/Mapt	2	0.363636363636364
GO:0048546	BP	GO:0048546	digestive tract morphogenesis	2/180	55/28943	0.0460713710027363	0.140500426363214	0.098400020661275	Sox11/Sfrp1	2	0.363636363636364

GO:0003014	BP	GO:0003014	renal system process	3/180	129/28943	0.0468234940029521	0.142527214923005	0.0998194899207056	Adm/Npr3/Agtr2	3	0.232558139534884
GO:0008286	BP	GO:0008286	insulin receptor signaling pathway	3/180	129/28943	0.0468234940029521	0.142527214923005	0.0998194899207056	Igf2/Nucb2/Pdk4	3	0.232558139534884
GO:0071219	BP	GO:0071219	cellular response to molecule of bacterial origin	5/180	314/28943	0.0469907479804362	0.142782117223669	0.0999980117394734	Abca1/Ccl2/Cd68/Tnfrsf1b/B2m	5	0.159235668789809
GO:0006006	BP	GO:0006006	glucose metabolic process	4/180	217/28943	0.0469949123929302	0.142782117223669	0.0999980117394734	Atf3/Igf2/Rbp4/Pdk4	4	0.184331797235023
GO:0016358	BP	GO:0016358	dendrite development	5/180	315/28943	0.0475186995432887	0.143643111966687	0.100601012760148	Sgk1/Nrp1/Bdnf/Apoe/Lpar1	5	0.158730158730159
GO:0010463	BP	GO:0010463	mesenchymal cell proliferation	2/180	56/28943	0.0475870180571249	0.143643111966687	0.100601012760148	Fgf9/Lmna	2	0.357142857142857
GO:0010596	BP	GO:0010596	negative regulation of endothelial cell migration	2/180	56/28943	0.0475870180571249	0.143643111966687	0.100601012760148	Gadd45a/Apoe	2	0.357142857142857
GO:0045604	BP	GO:0045604	regulation of epidermal cell differentiation	2/180	56/28943	0.0475870180571249	0.143643111966687	0.100601012760148	Hoxa7/Sfrp4	2	0.357142857142857
GO:0048713	BP	GO:0048713	regulation of oligodendrocyte differentiation	2/180	56/28943	0.0475870180571249	0.143643111966687	0.100601012760148	Tnfrsf1b/Enpp2	2	0.357142857142857
GO:1900271	BP	GO:1900271	regulation of long-term synaptic potentiation	2/180	56/28943	0.0475870180571249	0.143643111966687	0.100601012760148	Tyrobp/Apoe	2	0.357142857142857
GO:1901861	BP	GO:1901861	regulation of muscle tissue development	2/180	56/28943	0.0475870180571249	0.143643111966687	0.100601012760148	Igf2/Bdnf	2	0.357142857142857
GO:0030522	BP	GO:0030522	intracellular receptor signaling pathway	4/180	218/28943	0.0476504138417553	0.143701294335738	0.100641761008854	Sfrp1/Ankrd17/Bdnf/Ifih1	4	0.18348623853211

GO:0030514	BP	GO:0030514	negative regulation of BMP signaling pathway	2/180	57/28943	0.0491207131964476	0.146776296220945	0.102795350552102	Sfrp1/Htra1	2	0.350877192982456
GO:0030857	BP	GO:0030857	negative regulation of epithelial cell differentiation	2/180	57/28943	0.0491207131964476	0.146776296220945	0.102795350552102	Hoxa7/Mmp9	2	0.350877192982456
GO:0035458	BP	GO:0035458	cellular response to interferon-beta	2/180	57/28943	0.0491207131964476	0.146776296220945	0.102795350552102	Ifit1/Gbp3	2	0.350877192982456
GO:0035567	BP	GO:0035567	non-canonical Wnt signaling pathway	2/180	57/28943	0.0491207131964476	0.146776296220945	0.102795350552102	Sfrp1/Sfrp4	2	0.350877192982456
GO:0044060	BP	GO:0044060	regulation of endocrine process	2/180	57/28943	0.0491207131964476	0.146776296220945	0.102795350552102	Inhbb/Agtr2	2	0.350877192982456
GO:0051489	BP	GO:0051489	regulation of filopodium assembly	2/180	57/28943	0.0491207131964476	0.146776296220945	0.102795350552102	Gap43/Nrp1	2	0.350877192982456
GO:0052372	BP	GO:0052372	modulation by symbiont of entry into host	2/180	57/28943	0.0491207131964476	0.146776296220945	0.102795350552102	Lgals1/Trim25	2	0.350877192982456
GO:0061383	BP	GO:0061383	trabecula morphogenesis	2/180	57/28943	0.0491207131964476	0.146776296220945	0.102795350552102	Sfrp1/Rbp4	2	0.350877192982456
GO:0072577	BP	GO:0072577	endothelial cell apoptotic process	2/180	57/28943	0.0491207131964476	0.146776296220945	0.102795350552102	Ccl2/Scg2	2	0.350877192982456
GO:1903533	BP	GO:1903533	regulation of protein targeting	2/180	57/28943	0.0491207131964476	0.146776296220945	0.102795350552102	Ccl2/Ank3	2	0.350877192982456
GO:0062023	CC	GO:0062023	collagen-containing extracellular matrix	18/177	396/28804	5.30860615365132e-11	1.43863226763951e-08	1.08407325664037e-08	Sfrp1/Ctsz/Acan/Mmp9/Cst3/Lgals3/Fgf9/S100a10/Ctsd/Col14a1/Igf2/Serpinb6a/Anxa2/Htra1/Ctsl/Lgals1/Serpina1a/Anxa4	18	0.454545454545455
GO:0043204	CC	GO:0043204	perikaryon	8/177	143/28804	3.04512856050865e-06	0.000331667371285376	0.000249926082848564	Adcyap1/Ccl2/Npy/Ckmt1/Bglap/Bdnf/Penk/Ctsl	8	0.559440559440559

GO:0030141	CC	GO:0030141	secretory granule	13/177	440/28804	3.67159451607427e-06	0.000331667371285376	0.000249926082848564	Adam8/Npy/Scg2/Cyp51/Msn/Bdnf/Fndc3a/Penk/Lyz1/Ctsl/Clca1/Lyz2/Pla2g4a	13	0.295454545454545
GO:0005770	CC	GO:0005770	late endosome	9/177	247/28804	2.3736544506251e-05	0.00160815089029851	0.00121181306163492	Cst7/Ctss/Cst3/Cd68/Parm1/Apoe/Anxa2/Ctsl/Cd63	9	0.364372469635628
GO:0001533	CC	GO:0001533	cornified envelope	5/177	61/28804	3.72823675399343e-05	0.00202070432066444	0.00152269037952574	Sprr1a/Dsp/Sprr1b/Lgals3/Anxa2	5	0.819672131147541
GO:0005902	CC	GO:0005902	microvillus	6/177	105/28804	4.8114997509066e-05	0.00217319405415948	0.00163759816083488	Msn/Car2/Lyz1/Ctsl/Clca1/Lyz2	6	0.571428571428571
GO:0031045	CC	GO:0031045	dense core granule	4/177	35/28804	6.21825622630749e-05	0.00240735348189904	0.00181404768105812	Adam8/Npy/Scg2/Penk	4	1.14285714285714
GO:0005635	CC	GO:0005635	nuclear envelope	11/177	435/28804	8.51188433536493e-05	0.00273687077086794	0.00206235358146584	Hoxa7/Cst3/Ankrd17/Lmna/Clic1/Nucb2/Apoe/Spin1/Anxa4/Pla2g4a/Lmo7	11	0.252873563218391
GO:0098858	CC	GO:0098858	actin-based cell projection	8/177	229/28804	9.08923872243964e-05	0.00273687077086794	0.00206235358146584	Msn/Gap43/Car2/Pvalb/Lyz1/Ctsl/Clca1/Lyz2	8	0.349344978165939
GO:0098992	CC	GO:0098992	neuronal dense core vesicle	3/177	16/28804	0.000120448568126816	0.0032641561962367	0.00245968654911602	Npy/Scg2/Penk	3	1.875
GO:0045121	CC	GO:0045121	membrane raft	10/177	394/28804	0.000173783797329816	0.00400549440570813	0.00301831778872549	Abca1/Mog/Tnfrsf1b/S100a10/Itgb2/Ctsd/Lat2/Rit2/Mapt/Anxa2	10	0.253807106598985
GO:0098857	CC	GO:0098857	membrane microdomain	10/177	395/28804	0.000177365065935415	0.00400549440570813	0.00301831778872549	Abca1/Mog/Tnfrsf1b/S100a10/Itgb2/Ctsd/Lat2/Rit2/Mapt/Anxa2	10	0.253164556962025
GO:0005882	CC	GO:0005882	intermediate filament	6/177	140/28804	0.000235019238468847	0.0048992472019275	0.00369180018323533	Dsp/Ina/Nes/Lmna/Nrp1/Gfap	6	0.428571428571429
GO:0030139	CC	GO:0030139	endocytic vesicle	7/177	211/28804	0.000343184553695104	0.00664307243224093	0.00500584988096617	Abca1/Ctss/Adam8/Ccl2/Mpeg1/B2m/Lpar1	7	0.33175355450237

GO:0043209	CC	GO:0043209	myelin sheath	7/177	215/28804	0.000384199726113732	0.00694120838512142	0.00523050855200449	Mog/Ina/Msn/Ckmt1/Car2/Anxa2/Gfap	7	0.325581395348837
GO:0045178	CC	GO:0045178	basal part of cell	8/177	300/28804	0.000562525453741542	0.00943740161856949	0.00711150092834524	Dsp/Abca1/Lin7b/Msn/Car2/Mark2/Anxa2/Ank3	8	0.266666666666667
GO:0150034	CC	GO:0150034	distal axon	9/177	379/28804	0.000592014123674101	0.00943740161856949	0.00711150092834524	Adcyap1/Ccl2/Npy/Ctsz/Nrp1/Bdnf/Mapt/Penk/Pclo	9	0.237467018469657
GO:0045335	CC	GO:0045335	phagocytic vesicle	5/177	113/28804	0.000680352702392347	0.010045326623804	0.00756959939801116	Abca1/Ctss/Adam8/Mpeg1/B2m	5	0.442477876106195
GO:0005771	CC	GO:0005771	multivesicular body	4/177	66/28804	0.000738097484020188	0.010045326623804	0.00756959939801116	Cst7/Cst3/Ctsl/Cd63	4	0.606060606060606
GO:0090575	CC	GO:0090575	RNA polymerase II transcription regulator complex	7/177	242/28804	0.000774682603664527	0.010045326623804	0.00756959939801116	Atf3/Jun/Cebpd/Irf9/Atf6/S100a10/Anxa2	7	0.289256198347107
GO:0045111	CC	GO:0045111	intermediate filament cytoskeleton	6/177	178/28804	0.000839304234205427	0.010045326623804	0.00756959939801116	Dsp/Ina/Nes/Lmna/Nrp1/Gfap	6	0.337078651685393
GO:0016323	CC	GO:0016323	basolateral plasma membrane	7/177	246/28804	0.000852555396116205	0.010045326623804	0.00756959939801116	Dsp/Abca1/Lin7b/Msn/Car2/Anxa2/Ank3	7	0.284552845528455
GO:0031965	CC	GO:0031965	nuclear membrane	7/177	246/28804	0.000852555396116205	0.010045326623804	0.00756959939801116	Hoxa7/Cst3/Ankrd17/Lmna/Nucb2/Spin1/Anxa4	7	0.284552845528455
GO:0043679	CC	GO:0043679	axon terminus	6/177	194/28804	0.00130734214846368	0.0147620717597358	0.0111238761755243	Adcyap1/Ccl2/Npy/Bdnf/Penk/Pclo	6	0.309278350515464
GO:0098802	CC	GO:0098802	plasma membrane signaling receptor complex	6/177	199/28804	0.00148820428397542	0.0161321344382935	0.0121562792038413	Itgax/B2m/Itgb2/Osmr/Sacm1l/Apbb1ip	6	0.301507537688442
GO:0005581	CC	GO:0005581	collagen trimer	4/177	81/28804	0.00158651255606042	0.0161639810944185	0.012180277072508	C1qa/C1qc/Msr1/Col14a1	4	0.493827160493827

GO:0009925	CC	GO:0009925	basal plasma membrane	7/177	275/28804	0.00161921504986237	0.0161639810944185	0.012180277072508	Dsp/Abca1/Lin7b/Msn/Car2/Anxa2/Ank3	7	0.254545454545455
GO:0005765	CC	GO:0005765	lysosomal membrane	6/177	205/28804	0.00172972491416287	0.0161639810944185	0.012180277072508	Cd68/Ctsd/Laptm5/Anxa2/Gfap/Cd63	6	0.292682926829268
GO:0098852	CC	GO:0098852	lytic vacuole membrane	6/177	205/28804	0.00172972491416287	0.0161639810944185	0.012180277072508	Cd68/Ctsd/Laptm5/Anxa2/Gfap/Cd63	6	0.292682926829268
GO:0005788	CC	GO:0005788	endoplasmic reticulum lumen	4/177	85/28804	0.00189447264761103	0.017113402916753	0.0128957085486505	Slc27a2/Bdnf/Lyz1/Lyz2	4	0.470588235294118
GO:0043195	CC	GO:0043195	terminal bouton	4/177	88/28804	0.0021509743503613	0.018407792884897	0.0138710888315013	Adcyap1/Npy/Bdnf/Pclo	4	0.454545454545455
GO:0044306	CC	GO:0044306	neuron projection terminus	6/177	215/28804	0.00219700120564911	0.018407792884897	0.0138710888315013	Adcyap1/Ccl2/Npy/Bdnf/Penk/Pclo	6	0.27906976744186
GO:0005791	CC	GO:0005791	rough endoplasmic reticulum	4/177	89/28804	0.00224153935498745	0.018407792884897	0.0138710888315013	Ccl2/Bglap/Lyz1/Lyz2	4	0.449438202247191
GO:0097386	CC	GO:0097386	glial cell projection	3/177	43/28804	0.00235017960375371	0.0184268704089996	0.0138854645925264	Lgals3/Mapt/Gfap	3	0.697674418604651
GO:0005883	CC	GO:0005883	neurofilament	2/177	12/28804	0.00237985411186342	0.0184268704089996	0.0138854645925264	Ina/Nrp1	2	1.66666666666667
GO:0000137	CC	GO:0000137	Golgi cis cisterna	2/177	13/28804	0.00280121739969441	0.021086942092144	0.0158899466532373	Lyz1/Lyz2	2	1.53846153846154
GO:0030176	CC	GO:0030176	integral component of endoplasmic reticulum membrane	5/177	157/28804	0.00291110606870799	0.0213218849897261	0.0160669865527554	Elovl6/Atf6/Slc27a2/B2m/Sacm1l	5	0.318471337579618
GO:0034362	CC	GO:0034362	low-density lipoprotein particle	2/177	14/28804	0.00325491969664899	0.0232127167839967	0.0174918122202189	Msr1/Apoe	2	1.42857142857143

GO:0031227	CC	GO:0031227	intrinsic component of endoplasmic reticulum membrane	5/177	165/28804	0.0036004166 2867426	0.0250182796 505314	0.0188523839666075	Elovl6/Atf6/Slc27a2/B2m/Sacm1l	5	0.303030303030303
GO:0005774	CC	GO:0005774	vacuolar membrane	6/177	252/28804	0.0047842922 8256367	0.0317664306 723686	0.0239374152279647	Cd68/Ctsd/Laptm5/Anxa2/Gfap/Cd63	6	0.238095238095238
GO:0031527	CC	GO:0031527	filopodium membrane	2/177	17/28804	0.0048059913 563362	0.0317664306 723686	0.0239374152279647	Msn/Gap43	2	1.17647058823529
GO:0098636	CC	GO:0098636	protein complex involved in cell adhesion	3/177	56/28804	0.0049801137 1070664	0.0321335908 476548	0.0242140867137115	Itgax/Itgb2/Lgals1	3	0.535714285714286
GO:0030133	CC	GO:0030133	transport vesicle	7/177	346/28804	0.0057269554 0910063	0.0360931375 782854	0.0271977808902209	Lin7b/Laptm5/Bdnf/Penk/Lyz1/Lyz2/ Pclo	7	0.202312138728324
GO:0043083	CC	GO:0043083	synaptic cleft	2/177	19/28804	0.0059943389 7579656	0.0369196786 918379	0.0278206162991515	Apoe/Cbln1	2	1.05263157894737
GO:0031985	CC	GO:0031985	Golgi cisterna	3/177	61/28804	0.0063229736 3127127	0.0380783523 12767	0.028693728291617	Nucb2/Lyz1/Lyz2	3	0.491803278688525
GO:0005604	CC	GO:0005604	basement membrane	4/177	120/28804	0.0065132371 9042571	0.0383714625 783775	0.0289145998842697	Acan/Cst3/Fgf9/Anxa2	4	0.333333333333333
GO:0043230	CC	GO:0043230	extracellular organelle	4/177	126/28804	0.0077149660 0046655	0.0435574122 109674	0.0328224430721603	Clic1/Anxa2/Gbp3/Cd63	4	0.317460317460317
GO:0065010	CC	GO:0065010	extracellular membrane-bounded organelle	4/177	126/28804	0.0077149660 0046655	0.0435574122 109674	0.0328224430721603	Clic1/Anxa2/Gbp3/Cd63	4	0.317460317460317
GO:0140534	CC	GO:0140534	endoplasmic reticulum protein-containing complex	4/177	131/28804	0.0088211290 9555371	0.0487862445 89695	0.0367626003122969	Elovl6/Piga/B2m/Ugt1a1	4	0.305343511450382
GO:0034399	CC	GO:0034399	nuclear periphery	4/177	132/28804	0.0090541160 0738345	0.0490733087 600183	0.0369789159038398	S100a10/Lmna/Mapt/Anxa2	4	0.303030303030303

GO:0030315	CC	GO:0030315	T-tubule	3/177	70/28804	0.0092386703370184	0.0490917580653331	0.0369928182741294	Msn/Stbd1/Ank3	3	0.428571428571429
GO:0042588	CC	GO:0042588	zymogen granule	2/177	25/28804	0.010265747292382	0.0535003368506831	0.0403148780308119	Clca1/Pla2g4a	2	0.8
GO:0045177	CC	GO:0045177	apical part of cell	8/177	488/28804	0.0108985487534193	0.0557265417391815	0.041992422207812	Igfbp2/Msn/Specc1/Osmr/Car2/Ctsl/Anxa4/Lmo7	8	0.163934426229508
GO:0031902	CC	GO:0031902	late endosome membrane	3/177	84/28804	0.0151112033236658	0.0744570200129716	0.056106668799831	Cd68/Anxa2/Cd63	3	0.357142857142857
GO:0044304	CC	GO:0044304	main axon	3/177	84/28804	0.0151112033236658	0.0744570200129716	0.056106668799831	Tnfrsf1b/Mapt/Ank3	3	0.357142857142857
GO:0005795	CC	GO:0005795	Golgi stack	3/177	86/28804	0.016086932504582	0.0778492626561021	0.0586628741708441	Nucb2/Lyz1/Lyz2	3	0.348837209302326
GO:0098688	CC	GO:0098688	parallel fiber to Purkinje cell synapse	2/177	32/28804	0.0165032221213948	0.0784626876297891	0.0591251171108141	Cbln1/Pclo	2	0.625
GO:0008305	CC	GO:0008305	integrin complex	2/177	34/28804	0.0185172785116725	0.0857926733372742	0.0646485866282043	Itgax/Itgb2	2	0.588235294117647
GO:0030667	CC	GO:0030667	secretory granule membrane	3/177	91/28804	0.0186781096933549	0.0857926733372742	0.0646485866282043	Adam8/Msn/Clca1	3	0.32967032967033
GO:0042383	CC	GO:0042383	sarcolemma	4/177	166/28804	0.0194947660086829	0.0869031761251614	0.0654853997602693	Msn/Stbd1/Anxa2/Ank3	4	0.240963855421687
GO:0030140	CC	GO:0030140	trans-Golgi network transport vesicle	2/177	35/28804	0.0195612315263279	0.0869031761251614	0.0654853997602693	Lyz1/Lyz2	2	0.571428571428571
GO:0005798	CC	GO:0005798	Golgi-associated vesicle	3/177	94/28804	0.0203373498818201	0.088893900289891	0.0669854987618522	Lyz1/Lyz2/Pclo	3	0.319148936170213

GO:0070062	CC	GO:0070062	extracellular exosome	3/177	104/28804	0.0264359606268081	0.11371659253754	0.0856904989406979	Clic1/Anxa2/Cd63	3	0.288461538461538
GO:0044232	CC	GO:0044232	organelle membrane contact site	2/177	42/28804	0.0275271675892796	0.115739571848164	0.087214903626117	Ckmt1/Sacm1l	2	0.476190476190476
GO:0016363	CC	GO:0016363	nuclear matrix	3/177	106/28804	0.0277604139119213	0.115739571848164	0.087214903626117	S100a10/Lmna/Anxa2	3	0.283018867924528
GO:0098982	CC	GO:0098982	GABA-ergic synapse	3/177	109/28804	0.0298123143578884	0.121586284801829	0.0916206612994946	Acan/Gap43/Pclo	3	0.275229357798165
GO:0019897	CC	GO:0019897	extrinsic component of plasma membrane	4/177	190/28804	0.0300600777923342	0.121586284801829	0.0916206612994946	S100a10/Gna14/Apoe/Anxa2	4	0.210526315789474
GO:0001772	CC	GO:0001772	immunological synapse	2/177	47/28804	0.0338807344988636	0.129319423228057	0.0974479242813868	Lgals3/Cd53	2	0.425531914893617
GO:0034358	CC	GO:0034358	plasma lipoprotein particle	2/177	47/28804	0.0338807344988636	0.129319423228057	0.0974479242813868	Msr1/Apoe	2	0.425531914893617
GO:0099738	CC	GO:0099738	cell cortex region	2/177	47/28804	0.0338807344988636	0.129319423228057	0.0974479242813868	Mark2/Pclo	2	0.425531914893617
GO:1990777	CC	GO:1990777	lipoprotein particle	2/177	47/28804	0.0338807344988636	0.129319423228057	0.0974479242813868	Msr1/Apoe	2	0.425531914893617
GO:1903561	CC	GO:1903561	extracellular vesicle	3/177	117/28804	0.035664421313215	0.134236919109462	0.10115347565444	Clic1/Anxa2/Cd63	3	0.256410256410256
GO:0030426	CC	GO:0030426	growth cone	4/177	202/28804	0.0363952498358993	0.135111132952448	0.101812234580598	Ctsz/Nrp1/Mapt/Pclo	4	0.198019801980198
GO:0032994	CC	GO:0032994	protein-lipid complex	2/177	50/28804	0.0379398060942472	0.138941722318122	0.1046987536598	Msr1/Apoe	2	0.4

GO:0098562	CC	GO:0098562	cytoplasmic side of membrane	4/177	207/28804	0.0392453700461821	0.141806603766871	0.10685756896785	Msn/Gna14/Mapt/Gfap	4	0.193236714975845
GO:0030427	CC	GO:0030427	site of polarized growth	4/177	209/28804	0.0404201895435561	0.144129886398733	0.108608265532547	Ctsz/Nrp1/Mapt/Pclo	4	0.191387559808612
GO:0005938	CC	GO:0005938	cell cortex	5/177	306/28804	0.0410911514177415	0.144619506937766	0.108977216336867	Ctsz/Pvalb/Mark2/Anxa2/Pclo	5	0.163398692810458
GO:0005200	MF	GO:0005200	structural constituent of cytoskeleton	6/171	69/28404	3.81834339621135e-06	0.00123150819835183	0.000996428471565111	Tubb6/Sprr1b/Ina/Lmna/Ank3/Gfap	6	0.869565217391304
GO:0004866	MF	GO:0004866	endopeptidase inhibitor activity	9/171	216/28404	6.90901392409571e-06	0.00123150819835183	0.000996428471565111	Cst7/Cst3/Ctla2b/Serpina3n/Serpinb6a/Serpina1b/Anxa2/Serpina1a/Serpina1c	9	0.416666666666667
GO:0030414	MF	GO:0030414	peptidase inhibitor activity	9/171	224/28404	9.25610806734664e-06	0.00123150819835183	0.000996428471565111	Cst7/Cst3/Ctla2b/Serpina3n/Serpinb6a/Serpina1b/Anxa2/Serpina1a/Serpina1c	9	0.401785714285714
GO:0061135	MF	GO:0061135	endopeptidase regulator activity	9/171	232/28404	1.22538129189237e-05	0.00123150819835183	0.000996428471565111	Cst7/Cst3/Ctla2b/Serpina3n/Serpinb6a/Serpina1b/Anxa2/Serpina1a/Serpina1c	9	0.387931034482759
GO:0004175	MF	GO:0004175	endopeptidase activity	12/171	453/28404	2.09767524547034e-05	0.00133203130394056	0.0010777629560556	Ctss/Mmp12/Adam8/Sfrp1/Ctsz/Mmp9/Ctla2a/Prss23/Ctla2b/Ctsd/Htra1/Ctsl	12	0.264900662251656
GO:0071813	MF	GO:0071813	lipoprotein particle binding	4/171	28/28404	2.31945749442385e-05	0.00133203130394056	0.0010777629560556	Abca1/Msr1/Mapt/Apoe	4	1.42857142857143
GO:0071814	MF	GO:0071814	protein-lipid complex binding	4/171	28/28404	2.31945749442385e-05	0.00133203130394056	0.0010777629560556	Abca1/Msr1/Mapt/Apoe	4	1.42857142857143
GO:0061134	MF	GO:0061134	peptidase regulator activity	9/171	266/28404	3.5982779313805e-05	0.0018081346605187	0.0014629840536797	Cst7/Cst3/Ctla2b/Serpina3n/Serpinb6a/Serpina1b/Anxa2/Serpina1a/Serpina1c	9	0.338345864661654
GO:0004197	MF	GO:0004197	cysteine-type endopeptidase activity	6/171	105/28404	4.28765743721979e-05	0.00186359943833441	0.00150786128946146	Ctss/Sfrp1/Ctsz/Ctla2a/Ctla2b/Ctsl	6	0.571428571428571

GO:0004857	MF	GO:0004857	enzyme inhibitor activity	11/171	415/28404	4.6358194983443e-05	0.00186359943833441	0.00150786128946146	Cst7/Cst3/Pkib/Ctla2b/Serpina3n/Ugt1a1/Serpinb6a/Serpina1b/Anxa2/Serpina1a/Serpina1c	11	0.265060240963855
GO:0004659	MF	GO:0004659	prenyltransferase activity	3/171	15/28404	9.2493831287085e-05	0.00338022910703711	0.00273498505911093	Fdft1/Fdps/Nus1	3	2
GO:0050839	MF	GO:0050839	cell adhesion molecule binding	9/171	308/28404	0.000110689835831615	0.00370810950035911	0.0030002771291201	Dsp/Adam8/Msn/Itgax/Itgb2/Sema7a/Igf2/Ank3/Gfap	9	0.292207792207792
GO:0004867	MF	GO:0004867	serine-type endopeptidase inhibitor activity	6/171	130/28404	0.00014039146569564	0.00425539177910038	0.00344308997052112	Serpina3n/Serpinb6a/Serpina1b/Anxa2/Serpina1a/Serpina1c	6	0.461538461538462
GO:0038024	MF	GO:0038024	cargo receptor activity	5/171	83/28404	0.000148197723650262	0.00425539177910038	0.00344308997052112	Abca1/Msr1/Enpp2/Itgb2/Stbd1	5	0.602409638554217
GO:0048156	MF	GO:0048156	tau protein binding	3/171	22/28404	0.000303500983995635	0.00813382637108301	0.00658117923190534	Sgk1/Mark2/Apoe	3	1.36363636363636
GO:0005544	MF	GO:0005544	calcium-dependent phospholipid binding	4/171	55/28404	0.000340445692876126	0.00855369803351266	0.0069209025722844	Anxa2/Pclo/Anxa4/Pla2g4a	4	0.727272727272727
GO:0005178	MF	GO:0005178	integrin binding	6/171	155/28404	0.000363382090160949	0.00859291766145302	0.00695263565694942	Adam8/Itgax/Itgb2/Sema7a/Igf2/Gfap	6	0.387096774193548
GO:0050840	MF	GO:0050840	extracellular matrix binding	4/171	57/28404	0.00039063621406164	0.00872420878070996	0.00705886492076297	Ctss/Lgals3/Anxa2/Lgals1	4	0.701754385964912
GO:0033218	MF	GO:0033218	amide binding	10/171	452/28404	0.000436647823188031	0.00923854868008361	0.00747502367673694	Cst3/Msr1/Kpna1/Itgb2/Ctsd/Fkbp5/Apoe/Npr3/Ctsl/Pla2g4a	10	0.221238938053097
GO:0005539	MF	GO:0005539	glycosaminoglycan binding	7/171	233/28404	0.00054833728447216	0.0110215794178904	0.00891769583694198	Ccl2/Sfrp1/Acan/Fgf9/Clec3b/Nrp1/Apoe	7	0.300429184549356
GO:0008201	MF	GO:0008201	heparin binding	6/171	170/28404	0.000592594014399665	0.011343942561365	0.00917852383205497	Ccl2/Sfrp1/Fgf9/Clec3b/Nrp1/Apoe	6	0.352941176470588

GO:0001540	MF	GO:0001540	amyloid-beta binding	4/171	66/28404	0.000683413802824118	0.0119031086999628	0.00963095205103041	Cst3/Msr1/Itgb2/Apoe	4	0.606060606060606
GO:0043236	MF	GO:0043236	laminin binding	3/171	29/28404	0.000698187407042938	0.0119031086999628	0.00963095205103041	Ctss/Lgals3/Lgals1	3	1.03448275862069
GO:0008234	MF	GO:0008234	cysteine-type peptidase activity	6/171	176/28404	0.000710633355221662	0.0119031086999628	0.00963095205103041	Ctss/Sfrp1/Ctsz/Ctla2a/Ctla2b/Ctsl	6	0.340909090909091
GO:0001664	MF	GO:0001664	G protein-coupled receptor binding	8/171	320/28404	0.000747590308086291	0.0120212521540276	0.00972654337678584	Adcyap1/Ccl2/Adm/Npy/Sfrp1/Nes/Gna14/Penk	8	0.25
GO:0005179	MF	GO:0005179	hormone activity	5/171	132/28404	0.00124646046463756	0.0192721964147807	0.0155933718045751	Adcyap1/Adm/Npy/Igf2/Inhbb	5	0.378787878787879
GO:0047499	MF	GO:0047499	calcium-independent phospholipase A2 activity	2/171	10/28404	0.00157082217923154	0.0233877968907808	0.0189233549077016	Prdx6/Pla2g4a	2	2
GO:0003796	MF	GO:0003796	lysozyme activity	2/171	11/28404	0.00191230665396104	0.0274552598175835	0.0222143893260888	Lyz1/Lyz2	2	1.81818181818182
GO:0042277	MF	GO:0042277	peptide binding	8/171	377/28404	0.00211008656321189	0.0292501654624545	0.0236666696200535	Cst3/Msr1/Kpna1/Itgb2/Ctsd/Apoe/Npr3/Ctsl	8	0.212201591511936
GO:0001968	MF	GO:0001968	fibronectin binding	3/171	48/28404	0.00303957903985958	0.0407303591341184	0.032955435905846	Ctss/Mmp9/Ctsl	3	0.625
GO:0061783	MF	GO:0061783	peptidoglycan muralytic activity	2/171	15/28404	0.00359346744222151	0.0465991584442919	0.0377039538080288	Lyz1/Lyz2	2	1.33333333333333
GO:0043394	MF	GO:0043394	proteoglycan binding	3/171	55/28404	0.00447107260111322	0.0561678495514849	0.0454460997942101	Ctss/Apoe/Ctsl	3	0.545454545454545
GO:0035497	MF	GO:0035497	cAMP response element binding	2/171	18/28404	0.00517453919721532	0.0630352956751685	0.0510026351495864	Jun/Atf6	2	1.11111111111111

GO:0002020	MF	GO:0002020	protease binding	5/171	185/28404	0.0053455937 3052504	0.0632037846 962078	0.0511389616944965	Cst3/Serpinb6a/Serpina1b/Anxa2/Serpina1c	5	0.27027027027027
GO:0070492	MF	GO:0070492	oligosaccharide binding	2/171	19/28404	0.0057605412 6968168	0.0661639311 546296	0.0535340527017035	Lgals3/Lgals1	2	1.05263157894737
GO:0034185	MF	GO:0034185	apolipoprotein binding	2/171	20/28404	0.0063754162 1352428	0.0688184712 909309	0.0556818738646181	Abca1/Mapt	2	1
GO:0016765	MF	GO:0016765	transferase activity, transferring alkyl or aryl (other than methyl) groups	3/171	63/28404	0.0065347999 1561979	0.0688184712 909309	0.0556818738646181	Fdft1/Fdps/Nus1	3	0.476190476190476
GO:0030246	MF	GO:0030246	carbohydrate binding	6/171	277/28404	0.0067980028 1537044	0.0688184712 909309	0.0556818738646181	Acan/Lgals3/Enpp2/Clec3b/Stbd1/Lgals1	6	0.216606498194946
GO:0005543	MF	GO:0005543	phospholipid binding	8/171	460/28404	0.0069293706 4737122	0.0688184712 909309	0.0556818738646181	Abca1/Gap43/Apoe/Anxa2/Lpar1/Pclo/Anxa4/Pla2g4a	8	0.173913043478261
GO:0004622	MF	GO:0004622	lysophospholipase activity	2/171	21/28404	0.0070187993 1076658	0.0688184712 909309	0.0556818738646181	Enpp2/Pla2g4a	2	0.952380952380952
GO:0120020	MF	GO:0120020	cholesterol transfer activity	2/171	21/28404	0.0070187993 1076658	0.0688184712 909309	0.0556818738646181	Abca1/Apoe	2	0.952380952380952
GO:1901681	MF	GO:1901681	sulfur compound binding	6/171	284/28404	0.0076418472 335567	0.0702616435 378264	0.0568495623283277	Ccl2/Sfrp1/Fgf9/Clec3b/Nrp1/Apoe	6	0.211267605633803
GO:0030215	MF	GO:0030215	semaphorin receptor binding	2/171	22/28404	0.0076903291 4344368	0.0702616435 378264	0.0568495623283277	Sema7a/Rit2	2	0.909090909090909
GO:0120015	MF	GO:0120015	sterol transfer activity	2/171	22/28404	0.0076903291 4344368	0.0702616435 378264	0.0568495623283277	Abca1/Apoe	2	0.909090909090909
GO:0071837	MF	GO:0071837	HMG box domain binding	2/171	23/28404	0.0083896475 6746999	0.0749475182 693986	0.0606409613648708	Jun/Egr2	2	0.869565217391304

GO:0031072	MF	GO:0031072	heat shock protein binding	4/171	143/28404	0.0110875281208168	0.0943943986807223	0.0763756721454391	Itgb2/Fkbp5/Mapt/Chordc1	4	0.27972027972028
GO:0004602	MF	GO:0004602	glutathione peroxidase activity	2/171	27/28404	0.0114577574338122	0.0943943986807223	0.0763756721454391	Gpx3/Prdx6	2	0.740740740740741
GO:0004869	MF	GO:0004869	cysteine-type endopeptidase inhibitor activity	3/171	78/28404	0.0117259648257638	0.0943943986807223	0.0763756721454391	Cst7/Cst3/Ctla2b	3	0.384615384615385
GO:0005184	MF	GO:0005184	neuropeptide hormone activity	2/171	28/28404	0.0122907594339093	0.0943943986807223	0.0763756721454391	Adcyap1/Npy	2	0.714285714285714
GO:0030247	MF	GO:0030247	polysaccharide binding	2/171	28/28404	0.0122907594339093	0.0943943986807223	0.0763756721454391	Enpp2/Stbd1	2	0.714285714285714
GO:0031625	MF	GO:0031625	ubiquitin protein ligase binding	6/171	315/28404	0.0123034709798946	0.0943943986807223	0.0763756721454391	Jun/Atf6/Tnfrsf1b/Laptm5/Prdx6/Egr2	6	0.19047619047619
GO:0005518	MF	GO:0005518	collagen binding	3/171	80/28404	0.0125539836718766	0.0943943986807223	0.0763756721454391	Ctss/Mmp12/Ctsl	3	0.375
GO:0008083	MF	GO:0008083	growth factor activity	4/171	149/28404	0.0127354771415785	0.0943943986807223	0.0763756721454391	Fgf9/Igf2/Bdnf/Inhbb	4	0.268456375838926
GO:0015103	MF	GO:0015103	inorganic anion transmembrane transporter activity	4/171	149/28404	0.0127354771415785	0.0943943986807223	0.0763756721454391	Slc25a14/Clic1/Clca1/Slc4a3	4	0.268456375838926
GO:0005520	MF	GO:0005520	insulin-like growth factor binding	2/171	29/28404	0.0131494684729364	0.0943943986807223	0.0763756721454391	Igfbp2/Htra1	2	0.689655172413793
GO:0017147	MF	GO:0017147	Wnt-protein binding	2/171	29/28404	0.0131494684729364	0.0943943986807223	0.0763756721454391	Sfrp1/Sfrp4	2	0.689655172413793
GO:0019838	MF	GO:0019838	growth factor binding	4/171	152/28404	0.0136149992225776	0.0960215734644947	0.0776922393310523	Igfbp2/Osmr/Nrp1/Htra1	4	0.263157894736842

GO:0098918	MF	GO:0098918	structural constituent of synapse	2/171	30/28404	0.014033548608228	0.0972670093190978	0.0786999368410611	Ina/Pclo	2	0.666666666666667
GO:0003725	MF	GO:0003725	double-stranded RNA binding	3/171	85/28404	0.0147674903891752	0.10061917180421	0.0814122128502252	Msn/Adarb1/Ifih1	3	0.352941176470588
GO:0016209	MF	GO:0016209	antioxidant activity	3/171	86/28404	0.0152349444707852	0.102074127954261	0.082589435815309	Gpx3/Apoe/Prdx6	3	0.348837209302326
GO:0044389	MF	GO:0044389	ubiquitin-like protein ligase binding	6/171	332/28404	0.0155702092294975	0.102610231315705	0.0830232036568548	Jun/Atf6/Tnfrsf1b/Laptm5/Prdx6/Egr2	6	0.180722891566265
GO:0015248	MF	GO:0015248	sterol transporter activity	2/171	32/28404	0.015876493730618	0.102941136769491	0.0832909433405934	Abca1/Apoe	2	0.625
GO:0004623	MF	GO:0004623	phospholipase A2 activity	2/171	34/28404	0.0178169682534051	0.111912831841701	0.0905500524720751	Prdx6/Pla2g4a	2	0.588235294117647
GO:0005501	MF	GO:0005501	retinoid binding	2/171	34/28404	0.0178169682534051	0.111912831841701	0.0905500524720751	Ugt1a1/Rbp4	2	0.588235294117647
GO:0005319	MF	GO:0005319	lipid transporter activity	4/171	166/28404	0.0182262719729235	0.112722482047927	0.0912051504394068	Abca1/Slc27a2/Apoe/Rbp4	4	0.240963855421687
GO:0048306	MF	GO:0048306	calcium-dependent protein binding	3/171	94/28404	0.0192741003126186	0.115955159514339	0.0938207496463229	S100a10/Anxa2/Anxa4	3	0.319148936170213
GO:0052689	MF	GO:0052689	carboxylic ester hydrolase activity	4/171	169/28404	0.0193258599190565	0.115955159514339	0.0938207496463229	Enpp2/Car2/Prdx6/Pla2g4a	4	0.236686390532544
GO:0019840	MF	GO:0019840	isoprenoid binding	2/171	37/28404	0.0209049199841688	0.118486406089919	0.0958688124686694	Ugt1a1/Rbp4	2	0.540540540540541
GO:0030280	MF	GO:0030280	structural constituent of skin epidermis	2/171	37/28404	0.0209049199841688	0.118486406089919	0.0958688124686694	Sprr1a/Sprr1b	2	0.540540540540541

GO:0071855	MF	GO:0071855	neuropeptide receptor binding	2/171	37/28404	0.0209049199841688	0.118486406089919	0.0958688124686694	Adcyap1/Npy	2	0.540540540540541
GO:0004896	MF	GO:0004896	cytokine receptor activity	3/171	97/28404	0.0209267035631448	0.118486406089919	0.0958688124686694	Osmr/Il10rb/Ccr5	3	0.309278350515464
GO:0004620	MF	GO:0004620	phospholipase activity	3/171	98/28404	0.0214943364255677	0.120010045042753	0.097101607536556	Enpp2/Prdx6/Pla2g4a	3	0.306122448979592
GO:0001965	MF	GO:0001965	G-protein alpha-subunit binding	2/171	40/28404	0.0241980179203326	0.133254838410598	0.107818133199463	Nucb2/Lpar1	2	0.5
GO:0004180	MF	GO:0004180	carboxypeptidase activity	2/171	41/28404	0.025339869130981	0.135821698542058	0.109895011389096	Ctsz/Ctsl	2	0.48780487804878
GO:0042056	MF	GO:0042056	chemoattractant activity	2/171	41/28404	0.025339869130981	0.135821698542058	0.109895011389096	Scg2/Lgals3	2	0.48780487804878
GO:0008237	MF	GO:0008237	metallopeptidase activity	4/171	185/28404	0.0258779266570824	0.136593893904024	0.110519804180003	Mmp12/Adam8/Mmp9/Clca1	4	0.216216216216216
GO:0004222	MF	GO:0004222	metalloendopeptidase activity	3/171	106/28404	0.0263372540761044	0.136593893904024	0.110519804180003	Mmp12/Adam8/Mmp9	3	0.283018867924528
GO:0004712	MF	GO:0004712	protein serine/threonine/tyrosine kinase activity	2/171	42/28404	0.0265032928470493	0.136593893904024	0.110519804180003	Sgk1/Pdk4	2	0.476190476190476
GO:0005044	MF	GO:0005044	scavenger receptor activity	2/171	43/28404	0.0276879912005134	0.140893322311473	0.113998524729629	Msr1/Enpp2	2	0.465116279069767
GO:0120013	MF	GO:0120013	lipid transfer activity	2/171	46/28404	0.031366796784096	0.157618153840082	0.127530792187969	Abca1/Apoe	2	0.434782608695652
GO:0032813	MF	GO:0032813	tumor necrosis factor receptor superfamily binding	2/171	48/28404	0.033920369632346	0.168345538175347	0.13621045115523	Bdnf/Nucb2	2	0.416666666666667

GO:0030295	MF	GO:0030295	protein kinase activator activity	3/171	120/28404	0.0360969098693828	0.17696289960356	0.143182864565331	Mob1b/Igf2/Cks2	3	0.25
GO:0051879	MF	GO:0051879	Hsp90 protein binding	2/171	50/28404	0.0365521242166663	0.177035589579517	0.143241678921368	Mapt/Chordc1	2	0.4
GO:0008374	MF	GO:0008374	O-acyltransferase activity	2/171	51/28404	0.0378966238959434	0.181362414359157	0.146742566213615	Prdx6/Pla2g4a	2	0.392156862745098
GO:0016298	MF	GO:0016298	lipase activity	3/171	123/28404	0.0383985472695455	0.181602541204204	0.146936855805444	Enpp2/Prdx6/Pla2g4a	3	0.24390243902439
GO:0000287	MF	GO:0000287	magnesium ion binding	4/171	213/28404	0.0402185435654917	0.187998308294508	0.152111749837661	Idi1/Snrk/Mark2/Ppa2	4	0.187793427230047
GO:0019887	MF	GO:0019887	protein kinase regulator activity	4/171	214/28404	0.0407995635439332	0.188522121203002	0.152535573322146	Mob1b/Pkib/Igf2/Cks2	4	0.186915887850467
GO:0019209	MF	GO:0019209	kinase activator activity	3/171	127/28404	0.0415811692568927	0.189950341378078	0.153691163880142	Mob1b/Igf2/Cks2	3	0.236220472440945
GO:0005126	MF	GO:0005126	cytokine receptor binding	5/171	315/28404	0.0423315093184908	0.191205244337453	0.15470652134138	Ccl2/Nes/Osmr/Bdnf/Nucb2	5	0.158730158730159
GO:0048020	MF	GO:0048020	CCR chemokine receptor binding	2/171	56/28404	0.0448944729729344	0.200528645945774	0.162250200568851	Ccl2/Nes	2	0.357142857142857
GO:0004601	MF	GO:0004601	peroxidase activity	2/171	58/28404	0.0478171539884236	0.211236218718091	0.170913829756193	Gpx3/Prdx6	2	0.344827586206897
GO:0140375	MF	GO:0140375	immune receptor activity	3/171	136/28404	0.0492105195380254	0.213120205749031	0.172438186898273	Osmr/Il10rb/Ccr5	3	0.220588235294118
GO:0043539	MF	GO:0043539	Protein serine/threonine kinase activator activity	2/171	59/28404	0.0493039281956713	0.213120205749031	0.172438186898273	Igf2/Cks2	2	0.338983050847458
