# Supplementary material for: Integrative bulk and single-cell transcriptome analyses reveal RNA modification–related biomarkers of spinal cord injury
Source: Neural Regen Res. 2025 Nov 25;21(7):3249–66. doi: 10.4103/NRR.NRR-D-25-00080 (PMC13379046; doi:10.4103/NRR.NRR-D-25-00080)
Supplement: Supplementary file 4 [file NRR-21-3249_Suppl3.pdf]

Additional Table 5 Kyoto Encyclopedia of Genes and Genomes (KEGG) enrichment analysis of DEGs

	ID	Description	GeneRatio	BgRatio	P value	Padj	q value	geneID	Count
mmu04610	mmu04610	Complement and coagulation cascades - Mus musculus (house mouse)	9/114	94/9784	1.33895961815296e-06	0.000311977591029641	0.000293161684816649	C1qa/C4a/C1qc/Procr/Itgax/Itgb2/Serpina1b/Serpina1a/Serpina1c	9
mmu00100	mmu00100	Steroid biosynthesis - Mus musculus (house mouse)	4/114	20/9784	7.33419109274707e-05	0.00854433262305034	0.00802900919627048	Cyp51/Fdft1/Sqle/Msmo1	4
mmu05133	mmu05133	Pertussis - Mus musculus (house mouse)	6/114	77/9784	0.000265310706791095	0.0206057982274417	0.0193630270219466	Jun/C1qa/C4a/C1qc/Itgb2/Calml4	6
mmu04142	mmu04142	Lysosome - Mus musculus (house mouse)	7/114	135/9784	0.000984120341920271	0.0458600079334846	0.043094111814614	Ctss/Ctsz/Cd68/Ctsd/Laptm5/Ctsl/Cd63	7
mmu04210	mmu04210	Apoptosis - Mus musculus (house mouse)	7/114	135/9784	0.000984120341920271	0.0458600079334846	0.043094111814614	Jun/Gadd45a/Ctss/Ctsz/Ctsd/Lmna/Ctsl	7
mmu00900	mmu00900	Terpenoid backbone biosynthesis - Mus musculus (house mouse)	3/114	23/9784	0.00230203225334948	0.089395585838405	0.0840039839818759	Idi1/Fdps/Nus1	3
mmu04970	mmu04970	Salivary secretion - Mus musculus (house mouse)	5/114	87/9784	0.00340193393983874	0.113235801140347	0.106406354809994	Adcy9/Cst3/Calml4/Lyz1/Lyz2	5

mmu04915	mmu04915	Estrogen signaling pathway - Mus musculus (house mouse)	6/114	134/9784	0.00470411242693933	0.137007274434608	0.128744129579392	Jun/Adcy9/Mmp9/Ctsd/Calml4/Fkbp5	6
mmu00920	mmu00920	Sulfur metabolism - Mus musculus (house mouse)	2/114	11/9784	0.00691058778345589	0.162201547091587	0.152418892229727	Selenbp1/Papss2	2
mmu05142	mmu05142	Chagas disease - Mus musculus (house mouse)	5/114	103/9784	0.00696143978933851	0.162201547091587	0.152418892229727	Jun/Ccl2/C1qa/C1qc/Gna14	5
mmu05164	mmu05164	Influenza A - Mus musculus (house mouse)	6/114	174/9784	0.0159552595053254	0.332733258970691	0.312665542651474	Ccl2/Irf9/Kpna1/Fdps/Ifih1/Trim25	6
mmu05152	mmu05152	Tuberculosis - Mus musculus (house mouse)	6/114	180/9784	0.0185511921734348	0.332733258970691	0.312665542651474	Ctss/Itgax/Itgb2/Ctsd/Il10rb/Calml4	6
mmu05323	mmu05323	Rheumatoid arthritis - Mus musculus (house mouse)	4/114	87/9784	0.0185645165949313	0.332733258970691	0.312665542651474	Jun/Ccl2/Itgb2/Ctsl	4
mmu04912	mmu04912	GnRH signaling pathway - Mus musculus (house mouse)	4/114	90/9784	0.0207565570074235	0.345448413052119	0.324613823875495	Jun/Adcy9/Calml4/Pla2g4a	4
mmu04061	mmu04061	Viral protein interaction with cytokine and cytokine receptor - Mus musculus (house mouse)	4/114	95/9784	0.0247511005374114	0.366905977744936	0.344777245859258	Ccl2/Tnfrsf1b/Il10rb/Ccr5	4

mmu05166	mmu05166	Human T-cell leukemia virus 1 infection - Mus musculus (house mouse)	7/114	247/9784	0.0251952602743304	0.366905977744936	0.344777245859258	Jun/Adcy9/Fdps/B2m/Itgb2/Nrp1/Egr2	7
mmu00280	mmu00280	Valine, leucine and isoleucine degradation - Mus musculus (house mouse)	3/114	57/9784	0.0285980467272848	0.378016879137766	0.355218029639283	Bckdhb/Hibadh/Oxct1	3
mmu05418	mmu05418	Fluid shear stress and atherosclerosis - Mus musculus (house mouse)	5/114	148/9784	0.0292030207059218	0.378016879137766	0.355218029639283	Jun/Ccl2/Mmp9/Calml4/Ctsl	5
mmu05205	mmu05205	Proteoglycans in cancer - Mus musculus (house mouse)	6/114	204/9784	0.0318110369275025	0.390103768637268	0.366575938001137	Mmp9/Msn/Igf2/Ctsl/Ank3/Cd63	6
mmu04015	mmu04015	Rap1 signaling pathway - Mus musculus (house mouse)	6/114	214/9784	0.0387998545586566	0.421674378637584	0.396242470099921	Adcy9/Fgf9/Itgb2/Calml4/Lpar1/Apbb1ip	6
mmu05171	mmu05171	Coronavirus disease - COVID-19 - Mus musculus (house mouse)	8/114	334/9784	0.0410456250253099	0.421674378637584	0.396242470099921	Jun/Ccl2/C1qa/C4a/Irf9/C1qc/Nrp1/Ifih1	8
mmu05417	mmu05417	Lipid and atherosclerosis - Mus musculus (house mouse)	6/114	217/9784	0.0410734532758976	0.421674378637584	0.396242470099921	Jun/Abca1/Ccl2/Mmp9/Atf6/Calml4	6

mmu04625	mmu04625	C-type lectin receptor signaling pathway - Mus musculus (house mouse)	4/114	112/9784	0.0416245094792465	0.421674378637584	0.396242470099921	Jun/Irf9/Calml4/Egr2	4
mmu04024	mmu04024	cAMP signaling pathway - Mus musculus (house mouse)	6/114	224/9784	0.0467025766607412	0.453404181748029	0.42605859409799	Adcyap1/Jun/Npy/Adcy9/Calml4/Bdnf	6
mmu04668	mmu04668	TNF signaling pathway - Mus musculus (house mouse)	4/114	118/9784	0.0488157968892979	0.454963227008257	0.427523610651536	Jun/Ccl2/Mmp9/Tnfrsf1b	4
