# Supplementary material for: Integrative bulk and single-cell transcriptome analyses reveal RNA modification–related biomarkers of spinal cord injury
Source: Neural Regen Res. 2025 Nov 25;21(7):3249–66. doi: 10.4103/NRR.NRR-D-25-00080 (PMC13379046; doi:10.4103/NRR.NRR-D-25-00080)
Supplement: Supplementary file 5 [file NRR-21-3249_Suppl4.pdf]

Additional Table 6 GO enrichment analysis of the candidate genes

	Ontology	ID	Description	GeneRatio	BgRatio	P value	p.adjust	qvalue	geneID	Count	richFactor
GO:0016126	BP	GO:0016126	Sterol biosynthetic process	3/23	65/28891	1.86392244509522e-05	0.0172642525239714	0.00835687982335919	Idi1/Sqle/Msmo1	3	0.461538461538462
GO:0034446	BP	GO:0034446	Substrate adhesion-dependent cell spreading	3/23	102/28891	7.18744355870364e-05	0.0172642525239714	0.00835687982335919	Sfrp1/S100a10/Nrp1	3	0.294117647058824
GO:0060979	BP	GO:0060979	Vasculogenesis involved in coronary vascular morphogenesis	2/23	16/28891	7.22561949668712e-05	0.0172642525239714	0.00835687982335919	Fgf9/Nrp1	2	1.25
GO:1903729	BP	GO:1903729	Regulation of plasma membrane organization	2/23	18/28891	9.2037423525223e-05	0.0172642525239714	0.00835687982335919	S100a10/Anxa2	2	1.11111111111111
GO:0009083	BP	GO:0009083	Branched-chain amino acid catabolic process	2/23	20/28891	0.000114184152338398	0.0172642525239714	0.00835687982335919	Bckdhb/Hibadh	2	1
GO:0045927	BP	GO:0045927	Positive regulation of growth	4/23	324/28891	0.000116175754034102	0.0172642525239714	0.00835687982335919	Sfrp1/Fgf9/Nrp1/Bdnf	4	0.123456790123457

GO:1901890	BP	GO:1901890	Positive regulation of cell junction assembly	3/23	129/28891	0.000144268353034821	0.0172642525239714	0.00835687982335919	S100a10/Nrp1/Bdnf	3	0.232558139534884
GO:1900120	BP	GO:1900120	Regulation of receptor binding	2/23	23/28891	0.000151824416825575	0.0172642525239714	0.00835687982335919	Bdnf/Anxa2	2	0.869565217391304
GO:0009081	BP	GO:0009081	Branched-chain amino acid metabolic process	2/23	24/28891	0.000165546430236507	0.0172642525239714	0.00835687982335919	Bckdhb/Hibadh	2	0.833333333333333
GO:0008203	BP	GO:0008203	Cholesterol metabolic process	3/23	150/28891	0.000225099666666486	0.0172642525239714	0.00835687982335919	Idi1/Sqle/Msmo1	3	0.2
GO:0001778	BP	GO:0001778	Plasma membrane repair	2/23	30/28891	0.000260158473779562	0.0172642525239714	0.00835687982335919	S100a10/Anxa2	2	0.666666666666667
GO:0051894	BP	GO:0051894	Positive regulation of focal adhesion assembly	2/23	30/28891	0.000260158473779562	0.0172642525239714	0.00835687982335919	S100a10/Nrp1	2	0.666666666666667

GO:1902652	BP	GO:1902652	Secondary alcohol metabolic process	3/23	162/28891	0.000282223258987577	0.0172642525239714	0.00835687982335919	Idi1/Sqle/Msmo1	3	0.185185185185185
GO:0016125	BP	GO:0016125	Sterol metabolic process	3/23	163/28891	0.000287365832393042	0.0172642525239714	0.00835687982335919	Idi1/Sqle/Msmo1	3	0.184049079754601
GO:0060977	BP	GO:0060977	Coronary vasculature morphogenesis	2/23	32/28891	0.000296353309497939	0.0172642525239714	0.00835687982335919	Fgf9/Nrp1	2	0.625
GO:0009755	BP	GO:0009755	Hormone-mediated signaling pathway	3/23	168/28891	0.000313988707399446	0.0172642525239714	0.00835687982335919	Sfrp1/Nr1d2/Bdnf	3	0.178571428571429
GO:0030947	BP	GO:0030947	Regulation of vascular endothelial growth factor receptor signaling pathway	2/23	33/28891	0.000315320155149836	0.0172642525239714	0.00835687982335919	Fgf9/Nrp1	2	0.606060606060606
GO:2001236	BP	GO:2001236	Regulation of extrinsic apoptotic signaling pathway	3/23	171/28891	0.000330701434330537	0.0172642525239714	0.00835687982335919	Sfrp1/Lmna/Nrp1	3	0.175438596491228

GO:0150117	BP	GO:0150117	Positive regulation of cell-substrate junction organization	2/23	35/28891	0.000354988435217643	0.0172642525239714	0.00835687982335919	S100a10/Nrp1	2	0.571428571428571
GO:2001233	BP	GO:2001233	Regulation of apoptotic signaling pathway	4/23	437/28891	0.000363969395426976	0.0172642525239714	0.00835687982335919	Sfrp1/Lmna/Nrp1/Bdnf	4	0.091533180778032
GO:0006694	BP	GO:0006694	Steroid biosynthetic process	3/23	179/28891	0.00037804932534244	0.0172642525239714	0.00835687982335919	Idi1/Sqle/Msmo1	3	0.167597765363128
GO:0006606	BP	GO:0006606	Protein import into nucleus	3/23	182/28891	0.000396870529709417	0.0172999471814241	0.00837415806707431	Kpna1/Fgf9/Lmna	3	0.164835164835165
GO:0051170	BP	GO:0051170	Import into nucleus	3/23	187/28891	0.00042956252963717	0.0179108898226976	0.00866988904210489	Kpna1/Fgf9/Lmna	3	0.160427807486631
GO:0071229	BP	GO:0071229	Cellular response to acid chemical	3/23	195/28891	0.000485391119445252	0.0181837508222285	0.0088019692800645	S100a10/Bdnf/Anxa2	3	0.153846153846154

GO:1900026	BP	GO:1900026	Positive regulation of substrate adhesion-dependent cell spreading	2/23	41/28891	0.000487808719477419	0.0181837508222285	0.0088019692800645	S100a10/Nrp1	2	0.48780487804878
GO:0051099	BP	GO:0051099	Positive regulation of binding	3/23	197/28891	0.000500039854043828	0.0181837508222285	0.0088019692800645	S100a10/Bdnf/Anxa2	3	0.152284263959391
GO:0010464	BP	GO:0010464	Regulation of mesenchymal cell proliferation	2/23	42/28891	0.000511951274452731	0.0181837508222285	0.0088019692800645	Fgf9/Lmna	2	0.476190476190476
GO:0050767	BP	GO:0050767	Regulation of neurogenesis	4/23	494/28891	0.000577661742092929	0.0192162554315295	0.00930176021656827	S100a10/Nrp1/Bdnf/Anxa2	4	0.0809716599190283
GO:0045880	BP	GO:0045880	Positive regulation of smoothened signaling pathway	2/23	45/28891	0.000587800603030062	0.0192162554315295	0.00930176021656827	Sfrp1/Fgf9	2	0.444444444444444
GO:0030307	BP	GO:0030307	Positive regulation of cell growth	3/23	210/28891	0.000602211143256179	0.0192162554315295	0.00930176021656827	Sfrp1/Nrp1/Bdnf	3	0.142857142857143

GO:0008038	BP	GO:0008038	Neuron recognition	2/23	47/28891	0.000641209774566157	0.0192162554315295	0.00930176021656827	Nrp1/Bdnf	2	0.425531914893617
GO:0042789	BP	GO:0042789	mRNA transcription by RNA polymerase II	2/23	47/28891	0.000641209774566157	0.0192162554315295	0.00930176021656827	S100a10/Anxa2	2	0.425531914893617
GO:0001101	BP	GO:0001101	Response to acid chemical	3/23	218/28891	0.000671256127313496	0.0195071098816255	0.00944255030766353	S100a10/Bdnf/Anxa2	3	0.137614678899083
GO:0048639	BP	GO:0048639	Positive regulation of developmental growth	3/23	221/28891	0.000698396075457189	0.0196988775401013	0.00953537675779011	Fgf9/Nrp1/Bdnf	3	0.135746606334842
GO:0048010	BP	GO:0048010	Vascular endothelial growth factor receptor signaling pathway	2/23	51/28891	0.000754820920897921	0.0203368645276417	0.00984419873408702	Fgf9/Nrp1	2	0.392156862745098
GO:0001649	BP	GO:0001649	Osteoblast differentiation	3/23	230/28891	0.000783996817854794	0.0203368645276417	0.00984419873408702	Sfrp1/Fgf9/Lmna	3	0.130434782608696

GO:0009299	BP	GO:0009299	mRNA transcription	2/23	52/28891	0.000784633980732786	0.0203368645276417	0.00984419873408702	S100a10/Anxa2	2	0.384615384615385
GO:0097191	BP	GO:0097191	Extrinsic apoptotic signaling pathway	3/23	242/28891	0.000908150684711012	0.0210360160173607	0.0101826278070974	Sfrp1/Lmna/Nrp1	3	0.12396694214876
GO:0010463	BP	GO:0010463	Mesenchymal cell proliferation	2/23	56/28891	0.000909502215583259	0.0210360160173607	0.0101826278070974	Fgf9/Lmna	2	0.357142857142857
GO:1901888	BP	GO:1901888	Regulation of cell junction assembly	3/23	246/28891	0.000952148574502162	0.0210360160173607	0.0101826278070974	S100a10/Nrp1/Bdnf	3	0.121951219512195
GO:1901617	BP	GO:1901617	Organic hydroxy compound biosynthetic process	3/23	248/28891	0.000974646609579658	0.0210360160173607	0.0101826278070974	Idi1/Sqle/Msmo1	3	0.120967741935484
GO:0051496	BP	GO:0051496	Positive regulation of stress fiber assembly	2/23	58/28891	0.000975294252488989	0.0210360160173607	0.0101826278070974	S100a10/Nrp1	2	0.344827586206897

GO:0060828	BP	GO:0060828	Regulation of canonical Wnt signaling pathway	3/23	251/28891	0.00100902313621759	0.0210360160173607	0.0101826278070974	Sfrp1/Kpna1/Fgf9	3		0.119521912350598
GO:0006695	BP	GO:0006695	Cholesterol biosynthetic process	2/23	59/28891	0.00100902683712053	0.0210360160173607	0.0101826278070974	Idi1/Msmo1	2		0.338983050847458
GO:1900024	BP	GO:1900024	Regulation of substrate adhesion-dependent cell spreading	2/23	59/28891	0.00100902683712053	0.0210360160173607	0.0101826278070974	S100a10/Nrp1	2		0.338983050847458
GO:1902653	BP	GO:1902653	Secondary alcohol biosynthetic process	2/23	59/28891	0.00100902683712053	0.0210360160173607	0.0101826278070974	Idi1/Msmo1	2		0.338983050847458
GO:2001234	BP	GO:2001234	Negative regulation of apoptotic signaling pathway	3/23	253/28891	0.00103236335856169	0.0210646055502268	0.0101964667665331	Lmna/Nrp1/Bdnf	3		0.118577075098814
GO:0051893	BP	GO:0051893	Regulation of focal adhesion assembly	2/23	64/28891	0.00118602223104808	0.0232121493790838	0.0112360000836134	S100a10/Nrp1	2		0.3125

GO:0090109	BP	GO:0090109	Regulation of cell-substrate junction assembly	2/23	64/28891	0.00118602223104808	0.0232121493790838	0.0112360000836134	S100a10/Nrp1	2	0.3125
GO:0001954	BP	GO:0001954	Positive regulation of cell-matrix adhesion	2/23	66/28891	0.0012606934258226	0.0241800999072775	0.0117045431744793	S100a10/Nrp1	2	0.303030303030303
GO:0032233	BP	GO:0032233	Positive regulation of actin filament bundle assembly	2/23	68/28891	0.00133756787913706	0.0249128164046565	0.0120592196196186	S100a10/Nrp1	2	0.294117647058824
GO:0051148	BP	GO:0051148	Negative regulation of muscle cell differentiation	2/23	69/28891	0.00137682926949614	0.0249128164046565	0.0120592196196186	Fgf9/Bdnf	2	0.289855072463768
GO:0150116	BP	GO:0150116	Regulation of cell-substrate junction organization	2/23	69/28891	0.00137682926949614	0.0249128164046565	0.0120592196196186	S100a10/Nrp1	2	0.289855072463768
GO:0060560	BP	GO:0060560	Developmental growth involved in morphogenesis	3/23	292/28891	0.00155787779226223	0.0276667556070274	0.0133922827755876	Sfrp1/Nrp1/Bdnf	3	0.102739726027397

GO:0014706	BP	GO:0014706	Striated muscle tissue development	3/23	301/28891	0.0016989992040577	0.0296243679398425	0.0143398784495588	Fgf9/Lmna/Bdnf	3	0.0996677740863787
GO:0060070	BP	GO:0060070	Canonical Wnt signaling pathway	3/23	305/28891	0.00176420969128959	0.0302120909633342	0.0146243698093742	Sfrp1/Kpna1/Fgf9	3	0.0983606557377049
GO:0033143	BP	GO:0033143	Regulation of intracellular steroid hormone receptor signaling pathway	2/23	80/28891	0.00184471525253764	0.0305014125376482	0.0147644179014355	Sfrp1/Bdnf	2	0.25
GO:0060976	BP	GO:0060976	Coronary vasculature development	2/23	80/28891	0.00184471525253764	0.0305014125376482	0.0147644179014355	Fgf9/Nrp1	2	0.25
GO:0060485	BP	GO:0060485	Mesenchyme development	3/23	316/28891	0.00195158007060665	0.0317214455544369	0.0153549832495546	Sfrp1/Fgf9/Nrp1	3	0.0949367088607595
GO:0010721	BP	GO:0010721	Negative regulation of cell development	3/23	323/28891	0.00207705314324299	0.0321273220059682	0.0155514505292047	Sfrp1/Nrp1/Bdnf	3	0.0928792569659443

GO:0030111	BP	GO:0030111	Regulation of Wnt signaling pathway	3/23	323/28891	0.00207705314324299	0.0321273220059682	0.0155514505292047	Sfrp1/Kpna1/Fgf9	3	0.0928792569659443
GO:0090287	BP	GO:0090287	Regulation of cellular response to growth factor stimulus	3/23	323/28891	0.00207705314324299	0.0321273220059682	0.0155514505292047	Sfrp1/Fgf9/Nrp1	3	0.0928792569659443
GO:0048041	BP	GO:0048041	Focal adhesion assembly	2/23	87/28891	0.00217650939710999	0.0331313097115632	0.0160374376629157	S100a10/Nrp1	2	0.229885057471264
GO:0014855	BP	GO:0014855	Striated muscle cell proliferation	2/23	89/28891	0.00227612314739569	0.0337570781827194	0.0163403451825688	Kpna1/Fgf9	2	0.224719101123595
GO:0034504	BP	GO:0034504	Protein localization to nucleus	3/23	335/28891	0.00230366904781745	0.0337570781827194	0.0163403451825688	Kpna1/Fgf9/Lmna	3	0.0895522388059702
GO:0001822	BP	GO:0001822	Kidney development	3/23	336/28891	0.00232321914500467	0.0337570781827194	0.0163403451825688	Sfrp1/Nrp1/Bdnf	3	0.0892857142857143

GO:0072001	BP	GO:0072001	Renal system development	3/23	352/28891	0.00265019903485524	0.0338831426290634	0.016401367542305	Sfrp1/Nrp1/Bdnf	3	0.0852272727272727
GO:0044282	BP	GO:0044282	Small molecule catabolic process	3/23	353/28891	0.00267153110912311	0.0338831426290634	0.016401367542305	Bckdhb/Hibadh/Oxct1	3	0.084985835694051
GO:0006066	BP	GO:0006066	Alcohol metabolic process	3/23	354/28891	0.00269296969854341	0.0338831426290634	0.016401367542305	Idi1/Sqle/Msmo1	3	0.0847457627118644
GO:0009063	BP	GO:0009063	Amino acid catabolic process	2/23	97/28891	0.0026957975881048	0.0338831426290634	0.016401367542305	Bckdhb/Hibadh	2	0.206185567010309
GO:0044344	BP	GO:0044344	Cellular response to fibroblast growth factor stimulus	2/23	97/28891	0.0026957975881048	0.0338831426290634	0.016401367542305	Sfrp1/Fgf9	2	0.206185567010309
GO:0007044	BP	GO:0007044	Cell-substrate junction assembly	2/23	98/28891	0.00275063086234751	0.0338831426290634	0.016401367542305	S100a10/Nrp1	2	0.204081632653061

GO:0008202	BP	GO:0008202	Steroid metabolic process	3/23	358/28891	0.00277979288987222	0.0338831426290634	0.016401367542305	Idi1/Sqle/Msmo1	3	0.0837988826815642
GO:0006913	BP	GO:0006913	Nucleocytoplasmic transport	3/23	359/28891	0.00280176681174459	0.0338831426290634	0.016401367542305	Kpna1/Fgf9/Lmna	3	0.0835654596100279
GO:0051169	BP	GO:0051169	Nuclear transport	3/23	359/28891	0.00280176681174459	0.0338831426290634	0.016401367542305	Kpna1/Fgf9/Lmna	3	0.0835654596100279
GO:0001570	BP	GO:0001570	Vasculogenesis	2/23	99/28891	0.00280598902346919	0.0338831426290634	0.016401367542305	Fgf9/Nrp1	2	0.202020202020202
GO:0021675	BP	GO:0021675	Nerve development	2/23	99/28891	0.00280598902346919	0.0338831426290634	0.016401367542305	Nrp1/Bdnf	2	0.202020202020202
GO:0051492	BP	GO:0051492	Regulation of stress fiber assembly	2/23	99/28891	0.00280598902346919	0.0338831426290634	0.016401367542305	S100a10/Nrp1	2	0.202020202020202

GO:0008589	BP	GO:0008589	Regulation of smoothened signaling pathway	2/23	100/28891	0.00286187127523893	0.0338831426290634	0.016401367542305	Sfrp1/Fgf9	2	0.2
GO:0014033	BP	GO:0014033	Neural crest cell differentiation	2/23	100/28891	0.00286187127523893	0.0338831426290634	0.016401367542305	Sfrp1/Nrp1	2	0.2
GO:0071774	BP	GO:0071774	Response to fibroblast growth factor	2/23	100/28891	0.00286187127523893	0.0338831426290634	0.016401367542305	Sfrp1/Fgf9	2	0.2
GO:0050772	BP	GO:0050772	Positive regulation of axonogenesis	2/23	101/28891	0.00291827682226613	0.0341296033238198	0.0165206685316992	Nrp1/Bdnf	2	0.198019801980198
GO:2001237	BP	GO:2001237	Negative regulation of extrinsic apoptotic signaling pathway	2/23	102/28891	0.00297520486999971	0.0342154798248655	0.0165622376409261	Lmna/Nrp1	2	0.196078431372549
GO:0031589	BP	GO:0031589	Cell-substrate adhesion	3/23	368/28891	0.00300439635855589	0.0342154798248655	0.0165622376409261	Sfrp1/S100a10/Nrp1	3	0.0815217391304348

GO:0045921	BP	GO:0045921	Positive regulation of exocytosis	2/23	103/28891	0.00303265462472739	0.0342154798248655	0.0165622376409261	S100a10/Anxa2	2	0.194174757281553
GO:0032092	BP	GO:0032092	Positive regulation of protein binding	2/23	104/28891	0.00309062529357493	0.0344640657737019	0.0166825673741315	Bdnf/Anxa2	2	0.192307692307692
GO:0150115	BP	GO:0150115	Cell-substrate junction organization	2/23	105/28891	0.00314911608450541	0.0347126704027665	0.0168029061496296	S100a10/Nrp1	2	0.19047619047619
GO:0001657	BP	GO:0001657	Ureteric bud development	2/23	107/28891	0.00326765486864955	0.0356100115799422	0.0172372702999336	Sfrp1/Bdnf	2	0.186915887850467
GO:0007517	BP	GO:0007517	Muscle organ development	3/23	382/28891	0.00333722926866931	0.0359556640773346	0.0174045857615988	Nr1d2/Lmna/Bdnf	3	0.0785340314136126
GO:0090263	BP	GO:0090263	Positive regulation of canonical Wnt signaling pathway	2/23	109/28891	0.00338826465758238	0.0359556640773346	0.0174045857615988	Sfrp1/Fgf9	2	0.18348623853211

GO:0072163	BP	GO:0072163	Mesonephric epithelium development	2/23	110/28891	0.00344934420762751	0.0359556640773346	0.0174045857615988	Sfrp1/Bdnf	2	0.181818181818182
GO:0072164	BP	GO:0072164	Mesonephric tubule development	2/23	110/28891	0.00344934420762751	0.0359556640773346	0.0174045857615988	Sfrp1/Bdnf	2	0.181818181818182
GO:1903532	BP	GO:1903532	Positive regulation of secretion by cell	3/23	390/28891	0.0035372136593482	0.0363188487985511	0.0175803878164328	S100a10/Oxct1/Anxa2	3	0.0769230769230769
GO:0001823	BP	GO:0001823	Mesonephros development	2/23	113/28891	0.00363567203822827	0.0363188487985511	0.0175803878164328	Sfrp1/Bdnf	2	0.176991150442478
GO:0030516	BP	GO:0030516	Regulation of axon extension	2/23	113/28891	0.00363567203822827	0.0363188487985511	0.0175803878164328	Nrp1/Bdnf	2	0.176991150442478
GO:0110020	BP	GO:0110020	Regulation of actomyosin structure organization	2/23	113/28891	0.00363567203822827	0.0363188487985511	0.0175803878164328	S100a10/Nrp1	2	0.176991150442478

GO:0032231	BP	GO:0032231	Regulation of actin filament bundle assembly	2/23	115/28891	0.00376245705743944	0.0368374117019314	0.0178314017458446	S100a10/Nrp1	2	0.173913043478261
GO:0030038	BP	GO:0030038	Contractile actin filament bundle assembly	2/23	117/28891	0.00389128793638834	0.0368374117019314	0.0178314017458446	S100a10/Nrp1	2	0.170940170940171
GO:0043149	BP	GO:0043149	Stress fiber assembly	2/23	117/28891	0.00389128793638834	0.0368374117019314	0.0178314017458446	S100a10/Nrp1	2	0.170940170940171
GO:0048638	BP	GO:0048638	Regulation of developmental growth	3/23	405/28891	0.00393173757160012	0.0368374117019314	0.0178314017458446	Fgf9/Nrp1/Bdnf	3	0.0740740740740741
GO:0051098	BP	GO:0051098	Regulation of binding	3/23	405/28891	0.00393173757160012	0.0368374117019314	0.0178314017458446	S100a10/Bdnf/Anxa2	3	0.0740740740740741
GO:0030518	BP	GO:0030518	Intracellular steroid hormone receptor signaling pathway	2/23	118/28891	0.00395646861866417	0.0368374117019314	0.0178314017458446	Sfrp1/Bdnf	2	0.169491525423729

GO:0120034	BP	GO:0120034	Positive regulation of plasma membrane bounded cell projection assembly	2/23	118/28891	0.00395646861866417	0.0368374117019314	0.0178314017458446	Nrp1/Fam98a	2	0.169491525423729
GO:0035282	BP	GO:0035282	Segmentation	2/23	119/28891	0.00402215842200892	0.0370889416029477	0.0179531565192908	Sfrp1/Nrp1	2	0.168067226890756
GO:0043401	BP	GO:0043401	Steroid hormone mediated signaling pathway	2/23	123/28891	0.00428999326713569	0.039181938506506	0.0189662860231262	Sfrp1/Bdnf	2	0.16260162601626
GO:0048145	BP	GO:0048145	Regulation of fibroblast proliferation	2/23	126/28891	0.00449617705573082	0.04030921435034	0.0195119516255968	Sfrp1/Anxa2	2	0.158730158730159
GO:0001654	BP	GO:0001654	Eye development	3/23	428/28891	0.00458727206449468	0.04030921435034	0.0195119516255968	Fgf9/Nrp1/Bdnf	3	0.0700934579439252
GO:0001952	BP	GO:0001952	Regulation of cell-matrix adhesion	2/23	128/28891	0.00463614874390465	0.04030921435034	0.0195119516255968	S100a10/Nrp1	2	0.15625

GO:0150063	BP	GO:0150063	Visual system development	3/23	431/28891	0.00467737333712834	0.04030921435034	0.0195119516255968	Fgf9/Nrp1/Bdnf	3	0.0696055684454756
GO:0048565	BP	GO:0048565	Digestive tract development	2/23	129/28891	0.00470688701777661	0.04030921435034	0.0195119516255968	Sfrp1/Fgf9	2	0.155038759689922
GO:0061387	BP	GO:0061387	Regulation of extent of cell growth	2/23	129/28891	0.00470688701777661	0.04030921435034	0.0195119516255968	Nrp1/Bdnf	2	0.155038759689922
GO:0042886	BP	GO:0042886	Amide transport	3/23	432/28891	0.00470764547157256	0.04030921435034	0.0195119516255968	Sfrp1/Oxct1/Bdnf	3	0.0694444444444444
GO:0048880	BP	GO:0048880	Sensory system development	3/23	435/28891	0.00479917900985419	0.0407293156677006	0.0197153045490982	Fgf9/Nrp1/Bdnf	3	0.0689655172413793
GO:0051047	BP	GO:0051047	Positive regulation of secretion	3/23	437/28891	0.00486080040154459	0.0408904174129935	0.019793286953658	S100a10/Oxct1/Anxa2	3	0.068649885583524

GO:0072594	BP	GO:0072594	Establishment of protein localization to organelle	3/23	452/28891	0.00533835469788355	0.0445172361327854	0.0215488734257816	Kpna1/Fgf9/Lmna	3		0.0663716814159292
GO:0010811	BP	GO:0010811	Positive regulation of cell-substrate adhesion	2/23	140/28891	0.00551787793309167	0.0448268991175514	0.0216987679170629	S100a10/Nrp1	2		0.142857142857143
GO:0001558	BP	GO:0001558	Regulation of cell growth	3/23	459/28891	0.00557059390339292	0.0448268991175514	0.0216987679170629	Sfrp1/Nrp1/Bdnf	3		0.065359477124183
GO:0016055	BP	GO:0016055	Wnt signaling pathway	3/23	460/28891	0.00560426231424456	0.0448268991175514	0.0216987679170629	Sfrp1/Kpna1/Fgf9	3		0.0652173913043478
GO:0030177	BP	GO:0030177	Positive regulation of Wnt signaling pathway	2/23	142/28891	0.00567175871605425	0.0448268991175514	0.0216987679170629	Sfrp1/Fgf9	2		0.140845070422535
GO:0055123	BP	GO:0055123	Digestive system development	2/23	142/28891	0.00567175871605425	0.0448268991175514	0.0216987679170629	Sfrp1/Fgf9	2		0.140845070422535

GO:0198738	BP	GO:0198738	Cell-cell signaling by wnt	3/23	462/28891	0.00567196897264934	0.0448268991175514	0.0216987679170629	Sfrp1/Kpna1/Fgf9	3	0.0649350649350649
GO:0031346	BP	GO:0031346	Positive regulation of cell projection organization	3/23	463/28891	0.00570600747768323	0.0448268991175514	0.0216987679170629	Nrp1/Bdnf/Fam98a	3	0.0647948164146868
GO:0048675	BP	GO:0048675	Axon extension	2/23	143/28891	0.00574943544469115	0.0448268991175514	0.0216987679170629	Nrp1/Bdnf	2	0.13986013986014
GO:0046165	BP	GO:0046165	Alcohol biosynthetic process	2/23	144/28891	0.0058276020512225	0.0450699223155031	0.0218164049625562	Idi1/Msmo1	2	0.138888888888889
GO:0042692	BP	GO:0042692	Muscle cell differentiation	3/23	468/28891	0.00587805686786677	0.0450964522902739	0.0218292469787726	Fgf9/Lmna/Bdnf	3	0.0641025641025641
GO:0007015	BP	GO:0007015	Actin filament organization	3/23	471/28891	0.00598277654298366	0.0455355770215978	0.0220418083162556	Tagln2/S100a10/Nrp1	3	0.0636942675159236

GO:0048144	BP	GO:0048144	Fibroblast proliferation	2/23	150/28891	0.00630684667045546	0.0471243337334718	0.0228108568974931	Sfrp1/Anxa2	2	0.133333333333333
GO:0034329	BP	GO:0034329	Cell junction assembly	3/23	481/28891	0.0063399615885098	0.0471243337334718	0.0228108568974931	S100a10/Nrp1/Bdnf	3	0.0623700623700624
GO:0001503	BP	GO:0001503	Ossification	3/23	484/28891	0.00644956559308296	0.0471243337334718	0.0228108568974931	Sfrp1/Fgf9/Lmna	3	0.0619834710743802
GO:0006091	BP	GO:0006091	Generation of precursor metabolites and energy	3/23	484/28891	0.00644956559308296	0.0471243337334718	0.0228108568974931	Stbd1/Oxct1/Bdnf	3	0.0619834710743802
GO:0003206	BP	GO:0003206	Cardiac chamber morphogenesis	2/23	152/28891	0.00647047957943829	0.0471243337334718	0.0228108568974931	Fgf9/Nrp1	2	0.131578947368421
GO:0032102	BP	GO:0032102	Negative regulation of response to external stimulus	3/23	485/28891	0.00648635250554565	0.0471243337334718	0.0228108568974931	Nr1d2/Nrp1/Anxa2	3	0.0618556701030928

GO:0072073	BP	GO:0072073	Kidney epithelium development	2/23	155/28891	0.00671955012449566	0.0484514930029424	0.0234532774428381	Sfrp1/Bdnf	2	0.129032258064516
GO:0008584	BP	GO:0008584	Male gonad development	2/23	156/28891	0.00680353628271409	0.0486909798143493	0.0235692026761737	Sfrp1/Fgf9	2	0.128205128205128
GO:0046546	BP	GO:0046546	Development of primary male sexual characteristics	2/23	157/28891	0.00688800251268991	0.0489303289605157	0.0236850612717056	Sfrp1/Fgf9	2	0.127388535031847
GO:0022612	BP	GO:0022612	Gland morphogenesis	2/23	158/28891	0.00697294806571638	0.0491695381986912	0.0238008521438152	Sfrp1/Nrp1	2	0.126582278481013
GO:0008037	BP	GO:0008037	Cell recognition	2/23	160/28891	0.00714427415008406	0.0494368929937234	0.0239302670657286	Nrp1/Bdnf	2	0.125
GO:0045667	BP	GO:0045667	Regulation of osteoblast differentiation	2/23	162/28891	0.00731750856211198	0.0494368929937234	0.0239302670657286	Sfrp1/Lmna	2	0.123456790123457

GO:0051147	BP	GO:0051147	Regulation of muscle cell differentiation	2/23	163/28891	0.00740483952769855	0.0494368929937234	0.0239302670657286	Fgf9/Bdnf	2	0.122699386503067
GO:0031099	BP	GO:0031099	Regeneration	2/23	167/28891	0.00775890443810858	0.0494368929937234	0.0239302670657286	Kpna1/Bdnf	2	0.119760479041916
GO:0007224	BP	GO:0007224	Smoothened signaling pathway	2/23	168/28891	0.00784860221799663	0.0494368929937234	0.0239302670657286	Sfrp1/Fgf9	2	0.119047619047619
GO:0001765	BP	GO:0001765	Membrane raft assembly	1/23	10/28891	0.00793372898039035	0.0494368929937234	0.0239302670657286	S100a10	1	1
GO:0021562	BP	GO:0021562	Vestibulocochlear nerve development	1/23	10/28891	0.00793372898039035	0.0494368929937234	0.0239302670657286	Nrp1	1	1
GO:0021612	BP	GO:0021612	Facial nerve structural organization	1/23	10/28891	0.00793372898039035	0.0494368929937234	0.0239302670657286	Nrp1	1	1

GO:0032927	BP	GO:0032927	Positive regulation of activin receptor signaling pathway	1/23	10/28891	0.00793372898039035	0.0494368929937234	0.0239302670657286	Fgf9	1	1
GO:0046666	BP	GO:0046666	Retinal cell programmed cell death	1/23	10/28891	0.00793372898039035	0.0494368929937234	0.0239302670657286	Bdnf	1	1
GO:0060484	BP	GO:0060484	Lung-associated mesenchyme development	1/23	10/28891	0.00793372898039035	0.0494368929937234	0.0239302670657286	Fgf9	1	1
GO:0060982	BP	GO:0060982	Coronary artery morphogenesis	1/23	10/28891	0.00793372898039035	0.0494368929937234	0.0239302670657286	Nrp1	1	1
GO:0071481	BP	GO:0071481	Cellular response to X-ray	1/23	10/28891	0.00793372898039035	0.0494368929937234	0.0239302670657286	Sfrp1	1	1
GO:0099538	BP	GO:0099538	Synaptic signaling via neuropeptide	1/23	10/28891	0.00793372898039035	0.0494368929937234	0.0239302670657286	Bdnf	1	1

GO:1902224	BP	GO:1902224	Ketone body metabolic process	1/23	10/28891	0.00793372898039035	0.0494368929937234	0.0239302670657286	Oxct1	1	1
GO:1903242	BP	GO:1903242	Regulation of cardiac muscle hypertrophy in response to stress	1/23	10/28891	0.00793372898039035	0.0494368929937234	0.0239302670657286	Lmna	1	1
GO:0007009	BP	GO:0007009	Plasma membrane organization	2/23	169/28891	0.00793877113767821	0.0494368929937234	0.0239302670657286	S100a10/Anxa2	2	0.118343195266272
GO:0030178	BP	GO:0030178	Negative regulation of Wnt signaling pathway	2/23	169/28891	0.00793877113767821	0.0494368929937234	0.0239302670657286	Sfrp1/Fgf9	2	0.118343195266272
GO:0051017	BP	GO:0051017	Actin filament bundle assembly	2/23	174/28891	0.00839665698942807	0.0510128477850981	0.0246931187895596	S100a10/Nrp1	2	0.114942528735632
GO:0050768	BP	GO:0050768	Negative regulation of neurogenesis	2/23	175/28891	0.00848963725277268	0.0510128477850981	0.0246931187895596	Nrp1/Bdnf	2	0.114285714285714

GO:0061572	BP	GO:0061572	Actin filament bundle organization	2/23	177/28891	0.00867699499213923	0.0510128477850981	0.0246931187895596	S100a10/Nrp1	2	0.112994350282486
GO:0010612	BP	GO:0010612	Regulation of cardiac muscle adaptation	1/23	11/28891	0.00872378210297819	0.0510128477850981	0.0246931187895596	Lmna	1	0.909090909090909
GO:0021559	BP	GO:0021559	Trigeminal nerve development	1/23	11/28891	0.00872378210297819	0.0510128477850981	0.0246931187895596	Nrp1	1	0.909090909090909
GO:0042182	BP	GO:0042182	Ketone catabolic process	1/23	11/28891	0.00872378210297819	0.0510128477850981	0.0246931187895596	Oxct1	1	0.909090909090909
GO:0055015	BP	GO:0055015	Ventricular cardiac muscle cell development	1/23	11/28891	0.00872378210297819	0.0510128477850981	0.0246931187895596	Lmna	1	0.909090909090909
GO:0060385	BP	GO:0060385	Axonogenesis involved in innervation	1/23	11/28891	0.00872378210297819	0.0510128477850981	0.0246931187895596	Nrp1	1	0.909090909090909

GO:0060600	BP	GO:0060600	Dichotomous subdivision of an epithelial terminal unit	1/23	11/28891	0.00872378210297819	0.0510128477850981	0.0246931187895596	Nrp1	1	0.909090909090909
GO:0061299	BP	GO:0061299	Retina vasculature morphogenesis in camera-type eye	1/23	11/28891	0.00872378210297819	0.0510128477850981	0.0246931187895596	Nrp1	1	0.909090909090909
GO:0051961	BP	GO:0051961	Negative regulation of nervous system development	2/23	183/28891	0.0092501931866075	0.0517048044000349	0.025028065134093	Nrp1/Bdnf	2	0.109289617486339
GO:1902905	BP	GO:1902905	Positive regulation of supramolecular fiber organization	2/23	183/28891	0.0092501931866075	0.0517048044000349	0.025028065134093	S100a10/Nrp1	2	0.109289617486339
GO:0006607	BP	GO:0006607	NLS-bearing protein import into nucleus	1/23	12/28891	0.00951323338454413	0.0517048044000349	0.025028065134093	Kpna1	1	0.833333333333333
GO:0021561	BP	GO:0021561	Facial nerve development	1/23	12/28891	0.00951323338454413	0.0517048044000349	0.025028065134093	Nrp1	1	0.833333333333333

GO:0021610	BP	GO:0021610	Facial nerve morphogenesis	1/23	12/28891	0.00951323338454413	0.0517048044000349	0.025028065134093	Nrp1	1	0.833333333333333
GO:0038180	BP	GO:0038180	Nerve growth factor signaling pathway	1/23	12/28891	0.00951323338454413	0.0517048044000349	0.025028065134093	Bdnf	1	0.833333333333333
GO:0045843	BP	GO:0045843	Negative regulation of striated muscle tissue development	1/23	12/28891	0.00951323338454413	0.0517048044000349	0.025028065134093	Bdnf	1	0.833333333333333
GO:0048012	BP	GO:0048012	Hepatocyte growth factor receptor signaling pathway	1/23	12/28891	0.00951323338454413	0.0517048044000349	0.025028065134093	Nrp1	1	0.833333333333333
GO:0060525	BP	GO:0060525	Prostate glandular acinus development	1/23	12/28891	0.00951323338454413	0.0517048044000349	0.025028065134093	Sfrp1	1	0.833333333333333
GO:2000322	BP	GO:2000322	Regulation of glucocorticoid receptor signaling pathway	1/23	12/28891	0.00951323338454413	0.0517048044000349	0.025028065134093	Bdnf	1	0.833333333333333

GO:0046661	BP	GO:0046661	Male sex differentiation	2/23	186/28891	0.00954301395078851	0.0517048044000349	0.025028065134093	Sfrp1/Fgf9	2		0.10752688172043
GO:0050770	BP	GO:0050770	Regulation of axonogenesis	2/23	186/28891	0.00954301395078851	0.0517048044000349	0.025028065134093	Nrp1/Bdnf	2		0.10752688172043
GO:0071383	BP	GO:0071383	Cellular response to steroid hormone stimulus	2/23	186/28891	0.00954301395078851	0.0517048044000349	0.025028065134093	Sfrp1/Bdnf	2		0.10752688172043
GO:0009950	BP	GO:0009950	Dorsal/ventral axis specification	1/23	13/28891	0.010302083262731	0.0517261667484764	0.0250384057253478	Sfrp1	1		0.769230769230769
GO:0014029	BP	GO:0014029	Neural crest formation	1/23	13/28891	0.010302083262731	0.0517261667484764	0.0250384057253478	Sfrp1	1		0.769230769230769
GO:0014745	BP	GO:0014745	Negative regulation of muscle adaptation	1/23	13/28891	0.010302083262731	0.0517261667484764	0.0250384057253478	Lmna	1		0.769230769230769

GO:0021604	BP	GO:0021604	Cranial nerve structural organization	1/23	13/28891	0.010302083262731	0.0517261667484764	0.0250384057253478	Nrp1	1	0.769230769230769
GO:0033689	BP	GO:0033689	Negative regulation of osteoblast proliferation	1/23	13/28891	0.010302083262731	0.0517261667484764	0.0250384057253478	Sfrp1	1	0.769230769230769
GO:0042761	BP	GO:0042761	Very long-chain fatty acid biosynthetic process	1/23	13/28891	0.010302083262731	0.0517261667484764	0.0250384057253478	Elovl6	1	0.769230769230769
GO:0048672	BP	GO:0048672	Positive regulation of collateral sprouting	1/23	13/28891	0.010302083262731	0.0517261667484764	0.0250384057253478	Bdnf	1	0.769230769230769
GO:0071679	BP	GO:0071679	Commissural neuron axon guidance	1/23	13/28891	0.010302083262731	0.0517261667484764	0.0250384057253478	Nrp1	1	0.769230769230769
GO:0140059	BP	GO:0140059	Dendrite arborization	1/23	13/28891	0.010302083262731	0.0517261667484764	0.0250384057253478	Nrp1	1	0.769230769230769

GO:1902667	BP	GO:1902667	Regulation of axon guidance	1/23	13/28891	0.010302083262731	0.0517261667484764	0.0250384057253478	Nrp1	1	0.769230769230769
GO:1902946	BP	GO:1902946	Protein localization to early endosome	1/23	13/28891	0.010302083262731	0.0517261667484764	0.0250384057253478	Nrp1	1	0.769230769230769
GO:1904338	BP	GO:1904338	Regulation of dopaminergic neuron differentiation	1/23	13/28891	0.010302083262731	0.0517261667484764	0.0250384057253478	Sfrp1	1	0.769230769230769
GO:1905063	BP	GO:1905063	Regulation of vascular associated smooth muscle cell differentiation	1/23	13/28891	0.010302083262731	0.0517261667484764	0.0250384057253478	Fgf9	1	0.769230769230769
GO:2000271	BP	GO:2000271	Positive regulation of fibroblast apoptotic process	1/23	13/28891	0.010302083262731	0.0517261667484764	0.0250384057253478	Sfrp1	1	0.769230769230769
GO:0051495	BP	GO:0051495	Positive regulation of cytoskeleton organization	2/23	195/28891	0.0104461276720639	0.05188111490589	0.0251134094434965	S100a10/Nrp1	2	0.102564102564103

GO:0003205	BP	GO:0003205	Cardiac chamber development	2/23	200/28891	0.0109636921810314	0.05188111490589	0.0251134094434965	Fgf9/Nrp1	2	0.1
GO:0021979	BP	GO:0021979	Hypothalamus cell differentiation	1/23	14/28891	0.0110903321748774	0.05188111490589	0.0251134094434965	Nrp1	1	0.714285714285714
GO:0030497	BP	GO:0030497	Fatty acid elongation	1/23	14/28891	0.0110903321748774	0.05188111490589	0.0251134094434965	Elovl6	1	0.714285714285714
GO:0030949	BP	GO:0030949	Positive regulation of vascular endothelial growth factor receptor signaling pathway	1/23	14/28891	0.0110903321748774	0.05188111490589	0.0251134094434965	Fgf9	1	0.714285714285714
GO:0048505	BP	GO:0048505	Regulation of timing of cell differentiation	1/23	14/28891	0.0110903321748774	0.05188111490589	0.0251134094434965	Fgf9	1	0.714285714285714
GO:0048635	BP	GO:0048635	Negative regulation of muscle organ development	1/23	14/28891	0.0110903321748774	0.05188111490589	0.0251134094434965	Bdnf	1	0.714285714285714

GO:0060346	BP	GO:0060346	Bone trabecula formation	1/23	14/28891	0.0110903321748774	0.05188111490589	0.0251134094434965	Sfrp1	1	0.714285714285714
GO:0090179	BP	GO:0090179	Planar cell polarity pathway involved in neural tube closure	1/23	14/28891	0.0110903321748774	0.05188111490589	0.0251134094434965	Sfrp1	1	0.714285714285714
GO:0098917	BP	GO:0098917	Retrograde trans-synaptic signaling	1/23	14/28891	0.0110903321748774	0.05188111490589	0.0251134094434965	Bdnf	1	0.714285714285714
GO:0140042	BP	GO:0140042	Lipid droplet formation	1/23	14/28891	0.0110903321748774	0.05188111490589	0.0251134094434965	Sqle	1	0.714285714285714
GO:1904948	BP	GO:1904948	Midbrain dopaminergic neuron differentiation	1/23	14/28891	0.0110903321748774	0.05188111490589	0.0251134094434965	Sfrp1	1	0.714285714285714
GO:2000052	BP	GO:2000052	Positive regulation of non-canonical Wnt signaling pathway	1/23	14/28891	0.0110903321748774	0.05188111490589	0.0251134094434965	Sfrp1	1	0.714285714285714

GO:2000644	BP	GO:2000644	Regulation of receptor catabolic process	1/23	14/28891	0.0110903321748774	0.05188111490589	0.0251134094434965	Anxa2	1		0.714285714285714
GO:0030308	BP	GO:0030308	Negative regulation of cell growth	2/23	205/28891	0.011492453585958	0.0523524252848476	0.0253415504644287	Sfrp1/Nrp1	2		0.0975609756097561
GO:0010988	BP	GO:0010988	Regulation of low-density lipoprotein particle clearance	1/23	15/28891	0.0118779805580185	0.0523524252848476	0.0253415504644287	Anxa2	1		0.666666666666667
GO:0035729	BP	GO:0035729	Cellular response to hepatocyte growth factor stimulus	1/23	15/28891	0.0118779805580185	0.0523524252848476	0.0253415504644287	Nrp1	1		0.666666666666667
GO:0060442	BP	GO:0060442	Branching involved in prostate gland morphogenesis	1/23	15/28891	0.0118779805580185	0.0523524252848476	0.0253415504644287	Sfrp1	1		0.666666666666667
GO:0090178	BP	GO:0090178	Regulation of establishment of planar polarity involved in neural tube closure	1/23	15/28891	0.0118779805580185	0.0523524252848476	0.0253415504644287	Sfrp1	1		0.666666666666667

GO:0090435	BP	GO:0090435	Protein localization to nuclear envelope	1/23	15/28891	0.0118779805580185	0.0523524252848476	0.0253415504644287	Lmna	1	0.666666666666667
GO:0097475	BP	GO:0097475	Motor neuron migration	1/23	15/28891	0.0118779805580185	0.0523524252848476	0.0253415504644287	Nrp1	1	0.666666666666667
GO:0099550	BP	GO:0099550	Trans-synaptic signaling, modulating synaptic transmission	1/23	15/28891	0.0118779805580185	0.0523524252848476	0.0253415504644287	Bdnf	1	0.666666666666667
GO:1900029	BP	GO:1900029	Positive regulation of ruffle assembly	1/23	15/28891	0.0118779805580185	0.0523524252848476	0.0253415504644287	Fam98a	1	0.666666666666667
GO:2000095	BP	GO:2000095	Regulation of Wnt signaling pathway, planar cell polarity pathway	1/23	15/28891	0.0118779805580185	0.0523524252848476	0.0253415504644287	Sfrp1	1	0.666666666666667
GO:0043524	BP	GO:0043524	Negative regulation of neuron apoptotic process	2/23	210/28891	0.01203232279627	0.0523524252848476	0.0253415504644287	Nrp1/Bdnf	2	0.0952380952380952

GO:1990138	BP	GO:1990138	Neuron projection extension	2/23	211/28891	0.0121416217686739	0.0523524252848476	0.0253415504644287	Nrp1/Bdnf	2	0.0947867298578199
GO:0120032	BP	GO:0120032	Regulation of plasma membrane bounded cell projection assembly	2/23	212/28891	0.0122513608006483	0.0523524252848476	0.0253415504644287	Nrp1/Fam98a	2	0.0943396226415094
GO:0060491	BP	GO:0060491	Regulation of cell projection assembly	2/23	215/28891	0.0125832111992576	0.0523524252848476	0.0253415504644287	Nrp1/Fam98a	2	0.0930232558139535
GO:0007406	BP	GO:0007406	Negative regulation of neuroblast proliferation	1/23	16/28891	0.0126650288488891	0.0523524252848476	0.0253415504644287	Bdnf	1	0.625
GO:0030238	BP	GO:0030238	Male sex determination	1/23	16/28891	0.0126650288488891	0.0523524252848476	0.0253415504644287	Fgf9	1	0.625
GO:0032488	BP	GO:0032488	Cdc42 protein signal transduction	1/23	16/28891	0.0126650288488891	0.0523524252848476	0.0253415504644287	Nrp1	1	0.625

GO:0040034	BP	GO:0040034	Regulation of development, heterochronic	1/23	16/28891	0.0126650288488891	0.0523524252848476	0.0253415504644287	Fgf9	1	0.625
GO:0042921	BP	GO:0042921	Glucocorticoid receptor signaling pathway	1/23	16/28891	0.0126650288488891	0.0523524252848476	0.0253415504644287	Bdnf	1	0.625
GO:0043508	BP	GO:0043508	Negative regulation of JUN kinase activity	1/23	16/28891	0.0126650288488891	0.0523524252848476	0.0253415504644287	Sfrp1	1	0.625
GO:0051151	BP	GO:0051151	Negative regulation of smooth muscle cell differentiation	1/23	16/28891	0.0126650288488891	0.0523524252848476	0.0253415504644287	Fgf9	1	0.625
GO:0060026	BP	GO:0060026	Convergent extension	1/23	16/28891	0.0126650288488891	0.0523524252848476	0.0253415504644287	Sfrp1	1	0.625
GO:0061548	BP	GO:0061548	Ganglion development	1/23	16/28891	0.0126650288488891	0.0523524252848476	0.0253415504644287	Nrp1	1	0.625

GO:0090177	BP	GO:0090177	Establishment of planar polarity involved in neural tube closure	1/23	16/28891	0.0126650288488891	0.0523524252848476	0.0253415504644287	Sfrp1	1	0.625
GO:0099527	BP	GO:0099527	Postsynapse to nucleus signaling pathway	1/23	16/28891	0.0126650288488891	0.0523524252848476	0.0253415504644287	Kpna1	1	0.625
GO:1902043	BP	GO:1902043	Positive regulation of extrinsic apoptotic signaling pathway via death domain receptors	1/23	16/28891	0.0126650288488891	0.0523524252848476	0.0253415504644287	Sfrp1	1	0.625
GO:1902287	BP	GO:1902287	Semaphorin-plexin signaling pathway involved in axon guidance	1/23	16/28891	0.0126650288488891	0.0523524252848476	0.0253415504644287	Nrp1	1	0.625
GO:0031032	BP	GO:0031032	Actomyosin structure organization	2/23	218/28891	0.0129189967688233	0.0531730382030111	0.025738773774796	S100a10/Nrp1	2	0.0917431192660551
GO:0050796	BP	GO:0050796	Regulation of insulin secretion	2/23	221/28891	0.0132586985536691	0.0533056483763543	0.0258029646385733	Sfrp1/Oxct1	2	0.0904977375565611

GO:0008361	BP	GO:0008361	Regulation of cell size	2/23	222/28891	0.0133727994845434	0.0533056483763543	0.0258029646385733	Nrp1/Bdnf	2	0.0900900900900901
GO:0001921	BP	GO:0001921	Positive regulation of receptor recycling	1/23	17/28891	0.0134514774839184	0.0533056483763543	0.0258029646385733	Anxa2	1	0.588235294117647
GO:0010755	BP	GO:0010755	Regulation of plasminogen activation	1/23	17/28891	0.0134514774839184	0.0533056483763543	0.0258029646385733	S100a10	1	0.588235294117647
GO:0010832	BP	GO:0010832	Negative regulation of myotube differentiation	1/23	17/28891	0.0134514774839184	0.0533056483763543	0.0258029646385733	Bdnf	1	0.588235294117647
GO:0031958	BP	GO:0031958	Corticosteroid receptor signaling pathway	1/23	17/28891	0.0134514774839184	0.0533056483763543	0.0258029646385733	Bdnf	1	0.588235294117647
GO:0035728	BP	GO:0035728	Response to hepatocyte growth factor	1/23	17/28891	0.0134514774839184	0.0533056483763543	0.0258029646385733	Nrp1	1	0.588235294117647

GO:0060766	BP	GO:0060766	Negative regulation of androgen receptor signaling pathway	1/23	17/28891	0.0134514774839184	0.0533056483763543	0.0258029646385733	Sfrp1	1	0.588235294117647
GO:1902285	BP	GO:1902285	Semaphorin-plexin signaling pathway involved in neuron projection guidance	1/23	17/28891	0.0134514774839184	0.0533056483763543	0.0258029646385733	Nrp1	1	0.588235294117647
GO:0048839	BP	GO:0048839	Inner ear development	2/23	224/28891	0.0136022976593319	0.0536814957008202	0.0259848961133436	Fgf9/Bdnf	2	0.0892857142857143
GO:2000241	BP	GO:2000241	Regulation of reproductive process	2/23	225/28891	0.0137176935089322	0.0539150330945325	0.0260979414902463	Sfrp1/Fgf9	2	0.0888888888888889
GO:0017157	BP	GO:0017157	Regulation of exocytosis	2/23	226/28891	0.013833519605047	0.0539667845706196	0.0261229921471305	S100a10/Anxa2	2	0.0884955752212389
GO:0007623	BP	GO:0007623	Circadian rhythm	2/23	228/28891	0.0140664597564578	0.0539667845706196	0.0261229921471305	Nr1d2/Bdnf	2	0.087719298245614

GO:0010810	BP	GO:0010810	Regulation of cell-substrate adhesion	2/23	228/28891	0.0140664597564578	0.0539667845706196	0.0261229921471305	S100a10/Nrp1	2	0.087719298245614
GO:0043393	BP	GO:0043393	Regulation of protein binding	2/23	228/28891	0.0140664597564578	0.0539667845706196	0.0261229921471305	Bdnf/Anxa2	2	0.087719298245614
GO:0061138	BP	GO:0061138	Morphogenesis of a branching epithelium	2/23	229/28891	0.0141835724234479	0.0539667845706196	0.0261229921471305	Sfrp1/Nrp1	2	0.0873362445414847
GO:0042249	BP	GO:0042249	Establishment of planar polarity of embryonic epithelium	1/23	18/28891	0.0142373268992354	0.0539667845706196	0.0261229921471305	Sfrp1	1	0.555555555555556
GO:0055012	BP	GO:0055012	Ventricular cardiac muscle cell differentiation	1/23	18/28891	0.0142373268992354	0.0539667845706196	0.0261229921471305	Lmna	1	0.555555555555556
GO:0061430	BP	GO:0061430	Bone trabecula morphogenesis	1/23	18/28891	0.0142373268992354	0.0539667845706196	0.0261229921471305	Sfrp1	1	0.555555555555556

GO:0071696	BP	GO:0071696	Ectodermal placode development	1/23	18/28891	0.0142373268992354	0.0539667845706196	0.0261229921471305	Nrp1	1	0.555555555555556
GO:0007160	BP	GO:0007160	Cell-matrix adhesion	2/23	235/28891	0.0148952010896322	0.0554101994304219	0.026821687008195	S100a10/Nrp1	2	0.0851063829787234
GO:0050679	BP	GO:0050679	Positive regulation of epithelial cell proliferation	2/23	235/28891	0.0148952010896322	0.0554101994304219	0.026821687008195	Sfrp1/Fgf9	2	0.0851063829787234
GO:0014841	BP	GO:0014841	Skeletal muscle satellite cell proliferation	1/23	19/28891	0.015022577530667	0.0554101994304219	0.026821687008195	Kpna1	1	0.526315789473684
GO:0021783	BP	GO:0021783	Preganglionic parasympathetic fiber development	1/23	19/28891	0.015022577530667	0.0554101994304219	0.026821687008195	Nrp1	1	0.526315789473684
GO:0033145	BP	GO:0033145	Positive regulation of intracellular steroid hormone receptor signaling pathway	1/23	19/28891	0.015022577530667	0.0554101994304219	0.026821687008195	Bdnf	1	0.526315789473684

GO:1901569	BP	GO:1901569	Fatty acid derivative catabolic process	1/23	19/28891	0.015022577530667	0.0554101994304219	0.026821687008195	Oxct1	1	0.526315789473684
GO:1901862	BP	GO:1901862	Negative regulation of muscle tissue development	1/23	19/28891	0.015022577530667	0.0554101994304219	0.026821687008195	Bdnf	1	0.526315789473684
GO:0010976	BP	GO:0010976	Positive regulation of neuron projection development	2/23	239/28891	0.0153780959579653	0.0565041916616424	0.0273512414497386	Nrp1/Bdnf	2	0.0836820083682008
GO:0016054	BP	GO:0016054	Organic acid catabolic process	2/23	241/28891	0.0156220696209636	0.0565639305648258	0.0273801584754823	Bckdhb/Hibadh	2	0.0829875518672199
GO:0046395	BP	GO:0046395	Carboxylic acid catabolic process	2/23	241/28891	0.0156220696209636	0.0565639305648258	0.0273801584754823	Bckdhb/Hibadh	2	0.0829875518672199
GO:0046851	BP	GO:0046851	Negative regulation of bone remodeling	1/23	20/28891	0.0158072298137365	0.0565639305648258	0.0273801584754823	Sfrp1	1	0.5

GO:0048148	BP	GO:0048148	Behavioral response to cocaine	1/23	20/28891	0.0158072298137365	0.0565639305648258	0.0273801584754823	Bdnf	1	0.5
GO:0060973	BP	GO:0060973	Cell migration involved in heart development	1/23	20/28891	0.0158072298137365	0.0565639305648258	0.0273801584754823	Nrp1	1	0.5
GO:0061469	BP	GO:0061469	Regulation of type B pancreatic cell proliferation	1/23	20/28891	0.0158072298137365	0.0565639305648258	0.0273801584754823	Sfrp1	1	0.5
GO:1904177	BP	GO:1904177	Regulation of adipose tissue development	1/23	20/28891	0.0158072298137365	0.0565639305648258	0.0273801584754823	Lmna	1	0.5
GO:0045664	BP	GO:0045664	Regulation of neuron differentiation	2/23	245/28891	0.0161150420260247	0.0574510234310696	0.0278095618606023	Sfrp1/Bdnf	2	0.0816326530612245
GO:0003214	BP	GO:0003214	Cardiac left ventricle morphogenesis	1/23	21/28891	0.0165912841836677	0.0581513735942702	0.0281485711597312	Fgf9	1	0.476190476190476

GO:0048172	BP	GO:0048172	Regulation of short-term neuronal synaptic plasticity	1/23	21/28891	0.0165912841836677	0.0581513735942702	0.0281485711597312	Bdnf	1	0.476190476190476
GO:0090050	BP	GO:0090050	Positive regulation of cell migration involved in sprouting angiogenesis	1/23	21/28891	0.0165912841836677	0.0581513735942702	0.0281485711597312	Nrp1	1	0.476190476190476
GO:1904754	BP	GO:1904754	Positive regulation of vascular associated smooth muscle cell migration	1/23	21/28891	0.0165912841836677	0.0581513735942702	0.0281485711597312	Fgf9	1	0.476190476190476
GO:0001763	BP	GO:0001763	Morphogenesis of a branching structure	2/23	249/28891	0.0166146781697915	0.0581513735942702	0.0281485711597312	Sfrp1/Nrp1	2	0.0803212851405622
GO:0007411	BP	GO:0007411	Axon guidance	2/23	252/28891	0.0169937521961991	0.0588776561529705	0.0285001332127325	Nrp1/Bdnf	2	0.0793650793650794
GO:0097485	BP	GO:0097485	Neuron projection guidance	2/23	252/28891	0.0169937521961991	0.0588776561529705	0.0285001332127325	Nrp1/Bdnf	2	0.0793650793650794

GO:0048545	BP	GO:0048545	Response to steroid hormone	2/23	253/28891	0.0171209345773755	0.0588776561529705	0.0285001332127325	Sfrp1/Bdnf	2	0.0790513833992095
GO:0048762	BP	GO:0048762	Mesenchymal cell differentiation	2/23	254/28891	0.0172485280187041	0.0588776561529705	0.0285001332127325	Sfrp1/Nrp1	2	0.078740157480315
GO:0031290	BP	GO:0031290	Retinal ganglion cell axon guidance	1/23	22/28891	0.0173747410753813	0.0588776561529705	0.0285001332127325	Nrp1	1	0.454545454545455
GO:0042730	BP	GO:0042730	Fibrinolysis	1/23	22/28891	0.0173747410753813	0.0588776561529705	0.0285001332127325	Anxa2	1	0.454545454545455
GO:0048486	BP	GO:0048486	Parasympathetic nervous system development	1/23	22/28891	0.0173747410753813	0.0588776561529705	0.0285001332127325	Nrp1	1	0.454545454545455
GO:0048532	BP	GO:0048532	Anatomical structure arrangement	1/23	22/28891	0.0173747410753813	0.0588776561529705	0.0285001332127325	Nrp1	1	0.454545454545455

GO:0090201	BP	GO:0090201	Negative regulation of release of cytochrome c from mitochondria	1/23	22/28891	0.0173747410753813	0.0588776561529705	0.0285001332127325	Lmna	1	0.454545454545455
GO:0043583	BP	GO:0043583	Ear development	2/23	257/28891	0.0176337679620413	0.0595450122380199	0.0288231715020765	Fgf9/Bdnf	2	0.0778210116731518
GO:0008406	BP	GO:0008406	Gonad development	2/23	259/28891	0.0178926375449567	0.0595519007596043	0.0288265059381873	Sfrp1/Fgf9	2	0.0772200772200772
GO:0090092	BP	GO:0090092	Regulation of transmembrane receptor protein serine/threonine kinase signaling pathway	2/23	261/28891	0.0181531352245302	0.0595519007596043	0.0288265059381873	Sfrp1/Fgf9	2	0.0766283524904215
GO:0010984	BP	GO:0010984	Regulation of lipoprotein particle clearance	1/23	23/28891	0.0181576009234976	0.0595519007596043	0.0288265059381873	Anxa2	1	0.434782608695652
GO:0031579	BP	GO:0031579	Membrane raft organization	1/23	23/28891	0.0181576009234976	0.0595519007596043	0.0288265059381873	S100a10	1	0.434782608695652

GO:0048670	BP	GO:0048670	Regulation of collateral sprouting	1/23	23/28891	0.0181576009234976	0.0595519007596043	0.0288265059381873	Bdnf	1	0.434782608695652
GO:0045137	BP	GO:0045137	Development of primary sexual characteristics	2/23	263/28891	0.0184152556425371	0.0595519007596043	0.0288265059381873	Sfrp1/Fgf9	2	0.0760456273764259
GO:0045926	BP	GO:0045926	Negative regulation of growth	2/23	263/28891	0.0184152556425371	0.0595519007596043	0.0288265059381873	Sfrp1/Nrp1	2	0.0760456273764259
GO:0090276	BP	GO:0090276	Regulation of peptide hormone secretion	2/23	264/28891	0.0185469227071895	0.0595519007596043	0.0288265059381873	Sfrp1/Oxct1	2	0.0757575757575758
GO:0030073	BP	GO:0030073	Insulin secretion	2/23	266/28891	0.0188114672116173	0.0595519007596043	0.0288265059381873	Sfrp1/Oxct1	2	0.075187969924812
GO:0050730	BP	GO:0050730	Regulation of peptidyl-tyrosine phosphorylation	2/23	266/28891	0.0188114672116173	0.0595519007596043	0.0288265059381873	Sfrp1/Nrp1	2	0.075187969924812

GO:0007413	BP	GO:0007413	Axonal fasciculation	1/23	24/28891	0.0189398641623351	0.0595519007596043	0.0288265059381873	Nrp1	1	0.416666666666667
GO:0014856	BP	GO:0014856	Skeletal muscle cell proliferation	1/23	24/28891	0.0189398641623351	0.0595519007596043	0.0288265059381873	Kpna1	1	0.416666666666667
GO:0016202	BP	GO:0016202	Regulation of striated muscle tissue development	1/23	24/28891	0.0189398641623351	0.0595519007596043	0.0288265059381873	Bdnf	1	0.416666666666667
GO:0034383	BP	GO:0034383	Low-density lipoprotein particle clearance	1/23	24/28891	0.0189398641623351	0.0595519007596043	0.0288265059381873	Anxa2	1	0.416666666666667
GO:0035634	BP	GO:0035634	Response to stilbenoid	1/23	24/28891	0.0189398641623351	0.0595519007596043	0.0288265059381873	Idi1	1	0.416666666666667
GO:0042759	BP	GO:0042759	Long-chain fatty acid biosynthetic process	1/23	24/28891	0.0189398641623351	0.0595519007596043	0.0288265059381873	Elovl6	1	0.416666666666667

GO:0043586	BP	GO:0043586	Tongue development	1/23	24/28891	0.0189398641623351	0.0595519007596043	0.0288265059381873	Bdnf	1	0.416666666666667
GO:0048485	BP	GO:0048485	Sympathetic nervous system development	1/23	24/28891	0.0189398641623351	0.0595519007596043	0.0288265059381873	Nrp1	1	0.416666666666667
GO:0060080	BP	GO:0060080	Inhibitory postsynaptic potential	1/23	24/28891	0.0189398641623351	0.0595519007596043	0.0288265059381873	Bdnf	1	0.416666666666667
GO:0106030	BP	GO:0106030	Neuron projection fasciculation	1/23	24/28891	0.0189398641623351	0.0595519007596043	0.0288265059381873	Nrp1	1	0.416666666666667
GO:2000269	BP	GO:2000269	Regulation of fibroblast apoptotic process	1/23	24/28891	0.0189398641623351	0.0595519007596043	0.0288265059381873	Sfrp1	1	0.416666666666667
GO:0002791	BP	GO:0002791	Regulation of peptide secretion	2/23	269/28891	0.0192112999192844	0.0602079628189339	0.029144077276933	Sfrp1/Oxct1	2	0.0743494423791821

GO:0090087	BP	GO:0090087	Regulation of peptide transport	2/23	271/28891	0.0194798579411283	0.0608507614512771	0.0294552283628925	Sfrp1/Oxct1	2	0.0738007380073801
GO:0006520	BP	GO:0006520	Amino acid metabolic process	2/23	272/28891	0.0196147358283875	0.0610731547383884	0.0295628793585745	Bckdhb/Hibadh	2	0.0735294117647059
GO:0021854	BP	GO:0021854	Hypothalamus development	1/23	25/28891	0.019721531225912	0.0612069528985425	0.0296276452755142	Nrp1	1	0.4
GO:0005980	BP	GO:0005980	Glycogen catabolic process	1/23	26/28891	0.0205026025479447	0.0630192174470479	0.0305048843577719	Stbd1	1	0.384615384615385
GO:0010165	BP	GO:0010165	Response to X-ray	1/23	26/28891	0.0205026025479447	0.0630192174470479	0.0305048843577719	Sfrp1	1	0.384615384615385
GO:2000050	BP	GO:2000050	Regulation of non-canonical Wnt signaling pathway	1/23	26/28891	0.0205026025479447	0.0630192174470479	0.0305048843577719	Sfrp1	1	0.384615384615385

GO:0033002	BP	GO:0033002	Muscle cell proliferation	2/23	280/28891	0.0207080447892359	0.0630444919138959	0.0305171186367687	Kpna1/Fgf9	2	0.0714285714285714
GO:0048738	BP	GO:0048738	Cardiac muscle tissue development	2/23	280/28891	0.0207080447892359	0.0630444919138959	0.0305171186367687	Fgf9/Lmna	2	0.0714285714285714
GO:0110053	BP	GO:0110053	Regulation of actin filament organization	2/23	280/28891	0.0207080447892359	0.0630444919138959	0.0305171186367687	S100a10/Nrp1	2	0.0714285714285714
GO:0048863	BP	GO:0048863	Stem cell differentiation	2/23	284/28891	0.0212641547792726	0.0631903168446287	0.0305877061944803	Sfrp1/Nrp1	2	0.0704225352112676
GO:0003148	BP	GO:0003148	Outflow tract septum morphogenesis	1/23	27/28891	0.021283078561851	0.0631903168446287	0.0305877061944803	Nrp1	1	0.37037037037037
GO:0009251	BP	GO:0009251	Glucan catabolic process	1/23	27/28891	0.021283078561851	0.0631903168446287	0.0305877061944803	Stbd1	1	0.37037037037037

GO:0010954	BP	GO:0010954	Positive regulation of protein processing	1/23	27/28891	0.021283078561851	0.0631903168446287	0.0305877061944803	S100a10	1	0.37037037037037
GO:0031639	BP	GO:0031639	Plasminogen activation	1/23	27/28891	0.021283078561851	0.0631903168446287	0.0305877061944803	S100a10	1	0.37037037037037
GO:0048843	BP	GO:0048843	Negative regulation of axon extension involved in axon guidance	1/23	27/28891	0.021283078561851	0.0631903168446287	0.0305877061944803	Nrp1	1	0.37037037037037
GO:0060765	BP	GO:0060765	Regulation of androgen receptor signaling pathway	1/23	27/28891	0.021283078561851	0.0631903168446287	0.0305877061944803	Sfrp1	1	0.37037037037037
GO:1900407	BP	GO:1900407	Regulation of cellular response to oxidative stress	1/23	27/28891	0.021283078561851	0.0631903168446287	0.0305877061944803	Slc25a14	1	0.37037037037037
GO:0007530	BP	GO:0007530	Sex determination	1/23	28/28891	0.0220629597000746	0.0643111804043022	0.0311302678868309	Fgf9	1	0.357142857142857

GO:0032925	BP	GO:0032925	Regulation of activin receptor signaling pathway	1/23	28/28891	0.022062959700746	0.0643111804043022	0.0311302678868309	Fgf9	1	0.357142857142857
GO:0036010	BP	GO:0036010	Protein localization to endosome	1/23	28/28891	0.022062959700746	0.0643111804043022	0.0311302678868309	Nrp1	1	0.357142857142857
GO:0060445	BP	GO:0060445	Branching involved in salivary gland morphogenesis	1/23	28/28891	0.022062959700746	0.0643111804043022	0.0311302678868309	Nrp1	1	0.357142857142857
GO:0061437	BP	GO:0061437	Renal system vasculature development	1/23	28/28891	0.022062959700746	0.0643111804043022	0.0311302678868309	Nrp1	1	0.357142857142857
GO:0061440	BP	GO:0061440	Kidney vasculature development	1/23	28/28891	0.022062959700746	0.0643111804043022	0.0311302678868309	Nrp1	1	0.357142857142857
GO:0048588	BP	GO:0048588	Developmental cell growth	2/23	291/28891	0.0222523559821174	0.0646666951116684	0.0313023572188637	Nrp1/Bdnf	2	0.0687285223367698

GO:0003007	BP	GO:0003007	Heart morphogenesis	2/23	294/28891	0.02268167282334	0.0651955782593807	0.0315583667333153	Fgf9/Nrp1	2	0.0680272108843537
GO:0006120	BP	GO:0006120	Mitochondrial electron transport, NADH to ubiquinone	1/23	29/28891	0.0228422463974473	0.0651955782593807	0.0315583667333153	Bdnf	1	0.344827586206897
GO:0034104	BP	GO:0034104	Negative regulation of tissue remodeling	1/23	29/28891	0.0228422463974473	0.0651955782593807	0.0315583667333153	Sfrp1	1	0.344827586206897
GO:0048634	BP	GO:0048634	Regulation of muscle organ development	1/23	29/28891	0.0228422463974473	0.0651955782593807	0.0315583667333153	Bdnf	1	0.344827586206897
GO:1900027	BP	GO:1900027	Regulation of ruffle assembly	1/23	29/28891	0.0228422463974473	0.0651955782593807	0.0315583667333153	Fam98a	1	0.344827586206897
GO:1903319	BP	GO:1903319	Positive regulation of protein maturation	1/23	29/28891	0.0228422463974473	0.0651955782593807	0.0315583667333153	S100a10	1	0.344827586206897

GO:0000272	BP	GO:0000272	Polysaccharide catabolic process	1/23	30/28891	0.0236209390844702	0.0656593640058171	0.0317828654042756	Stbd1	1	0.333333333333333
GO:0001919	BP	GO:0001919	Pegulation of receptor recycling	1/23	30/28891	0.0236209390844702	0.0656593640058171	0.0317828654042756	Anxa2	1	0.333333333333333
GO:0006929	BP	GO:0006929	Substrate-dependent cell migration	1/23	30/28891	0.0236209390844702	0.0656593640058171	0.0317828654042756	Nrp1	1	0.333333333333333
GO:0007350	BP	GO:0007350	Blastoderm segmentation	1/23	30/28891	0.0236209390844702	0.0656593640058171	0.0317828654042756	Nrp1	1	0.333333333333333
GO:0021884	BP	GO:0021884	Forebrain neuron development	1/23	30/28891	0.0236209390844702	0.0656593640058171	0.0317828654042756	Nrp1	1	0.333333333333333
GO:0044346	BP	GO:0044346	Fibroblast apoptotic process	1/23	30/28891	0.0236209390844702	0.0656593640058171	0.0317828654042756	Sfrp1	1	0.333333333333333

GO:0060045	BP	GO:0060045	Positive regulation of cardiac muscle cell proliferation	1/23	30/28891	0.0236209390844702	0.0656593640058171	0.0317828654042756	Fgf9	1	0.333333333333333
GO:0060343	BP	GO:0060343	Trabecula formation	1/23	30/28891	0.0236209390844702	0.0656593640058171	0.0317828654042756	Sfrp1	1	0.333333333333333
GO:0061298	BP	GO:0061298	Retina vasculature development in camera-type eye	1/23	30/28891	0.0236209390844702	0.0656593640058171	0.0317828654042756	Nrp1	1	0.333333333333333
GO:0007214	BP	GO:0007214	Gamma-aminobutyric acid signaling pathway	1/23	31/28891	0.0243990381940318	0.0666628992252891	0.0322686335089759	Bdnf	1	0.32258064516129
GO:0021602	BP	GO:0021602	Cranial nerve morphogenesis	1/23	31/28891	0.0243990381940318	0.0666628992252891	0.0322686335089759	Nrp1	1	0.32258064516129
GO:0032367	BP	GO:0032367	Intracellular cholesterol transport	1/23	31/28891	0.0243990381940318	0.0666628992252891	0.0322686335089759	Anxa2	1	0.32258064516129

GO:0032801	BP	GO:0032801	Receptor catabolic process	1/23	31/28891	0.0243990381940318	0.0666628992252891	0.0322686335089759	Anxa2	1	0.32258064516129
GO:0033688	BP	GO:0033688	Regulation of osteoblast proliferation	1/23	31/28891	0.0243990381940318	0.0666628992252891	0.0322686335089759	Sfrp1	1	0.32258064516129
GO:0035767	BP	GO:0035767	Endothelial cell chemotaxis	1/23	31/28891	0.0243990381940318	0.0666628992252891	0.0322686335089759	Nrp1	1	0.32258064516129
GO:0043523	BP	GO:0043523	Regulation of neuron apoptotic process	2/23	310/28891	0.0250293071172436	0.0676341458000165	0.032738772073769	Nrp1/Bdnf	2	0.0645161290322581
GO:0003299	BP	GO:0003299	Muscle hypertrophy in response to stress	1/23	32/28891	0.0251765441580493	0.0676341458000165	0.032738772073769	Lmna	1	0.3125
GO:0010623	BP	GO:0010623	Programmed cell death involved in cell development	1/23	32/28891	0.0251765441580493	0.0676341458000165	0.032738772073769	Bdnf	1	0.3125

GO:0014898	BP	GO:0014898	Cardiac muscle hypertrophy in response to stress	1/23	32/28891	0.0251765441580493	0.0676341458000165	0.032738772073769	Lmna	1	0.3125
GO:0060384	BP	GO:0060384	Innervation	1/23	32/28891	0.0251765441580493	0.0676341458000165	0.032738772073769	Nrp1	1	0.3125
GO:0072659	BP	GO:0072659	Protein localization to plasma membrane	2/23	315/28891	0.0257826879851106	0.0676341458000165	0.032738772073769	S100a10/Anxa2	2	0.0634920634920635
GO:0061448	BP	GO:0061448	Connective tissue development	2/23	316/28891	0.0259344784813813	0.0676341458000165	0.032738772073769	Fgf9/Lmna	2	0.0632911392405063
GO:0002053	BP	GO:0002053	Positive regulation of mesenchymal cell proliferation	1/23	33/28891	0.0259534574081398	0.0676341458000165	0.032738772073769	Fgf9	1	0.303030303030303
GO:0008045	BP	GO:0008045	Motor neuron axon guidance	1/23	33/28891	0.0259534574081398	0.0676341458000165	0.032738772073769	Nrp1	1	0.303030303030303

GO:0014887	BP	GO:0014887	Cardiac muscle adaptation	1/23	33/28891	0.0259534574081398	0.0676341458000165	0.032738772073769	Lmna	1	0.303030303030303
GO:0048566	BP	GO:0048566	Embryonic digestive tract development	1/23	33/28891	0.0259534574081398	0.0676341458000165	0.032738772073769	Fgf9	1	0.303030303030303
GO:0048668	BP	GO:0048668	Collateral sprouting	1/23	33/28891	0.0259534574081398	0.0676341458000165	0.032738772073769	Bdnf	1	0.303030303030303
GO:0048841	BP	GO:0048841	Regulation of axon extension involved in axon guidance	1/23	33/28891	0.0259534574081398	0.0676341458000165	0.032738772073769	Nrp1	1	0.303030303030303
GO:0051150	BP	GO:0051150	Regulation of smooth muscle cell differentiation	1/23	33/28891	0.0259534574081398	0.0676341458000165	0.032738772073769	Fgf9	1	0.303030303030303
GO:0060740	BP	GO:0060740	Prostate gland epithelium morphogenesis	1/23	33/28891	0.0259534574081398	0.0676341458000165	0.032738772073769	Sfrp1	1	0.303030303030303

GO:0140058	BP	GO:0140058	Neuron projection arborization	1/23	33/28891	0.0259534574081398	0.0676341458000165	0.032738772073769	Nrp1	1	0.303030303030303
GO:2001014	BP	GO:2001014	Regulation of skeletal muscle cell differentiation	1/23	33/28891	0.0259534574081398	0.0676341458000165	0.032738772073769	Nr1d2	1	0.303030303030303
GO:0048511	BP	GO:0048511	Rhythmic process	2/23	317/28891	0.026086638946039	0.067796983060302	0.0328175945662622	Nr1d2/Bdnf	2	0.0630914826498423
GO:0030072	BP	GO:0030072	Peptide hormone secretion	2/23	321/28891	0.0266989679050655	0.0683076845095753	0.0330648031049039	Sfrp1/Oxct1	2	0.0623052959501558
GO:0007097	BP	GO:0007097	Nuclear migration	1/23	34/28891	0.026729778375622	0.0683076845095753	0.0330648031049039	Lmna	1	0.294117647058824
GO:0032366	BP	GO:0032366	Intracellular sterol transport	1/23	34/28891	0.026729778375622	0.0683076845095753	0.0330648031049039	Anxa2	1	0.294117647058824

GO:0060512	BP	GO:0060512	Prostate gland morphogenesis	1/23	34/28891	0.026729778375622	0.0683076845095753	0.0330648031049039	Sfrp1	1	0.294117647058824
GO:1904752	BP	GO:1904752	Regulation of vascular associated smooth muscle cell migration	1/23	34/28891	0.026729778375622	0.0683076845095753	0.0330648031049039	Fgf9	1	0.294117647058824
GO:2000178	BP	GO:2000178	Negative regulation of neural precursor cell proliferation	1/23	34/28891	0.026729778375622	0.0683076845095753	0.0330648031049039	Bdnf	1	0.294117647058824
GO:0018108	BP	GO:0018108	Peptidyl-tyrosine phosphorylation	2/23	322/28891	0.0268529687800937	0.0683076845095753	0.0330648031049039	Sfrp1/Nrp1	2	0.062111801242236
GO:0090596	BP	GO:0090596	Sensory organ morphogenesis	2/23	322/28891	0.0268529687800937	0.0683076845095753	0.0330648031049039	Fgf9/Bdnf	2	0.062111801242236
GO:0018212	BP	GO:0018212	Peptidyl-tyrosine modification	2/23	324/28891	0.0271620685063684	0.0689111738031939	0.033356926235891	Sfrp1/Nrp1	2	0.0617283950617284

GO:0000038	BP	GO:0000038	Very long-chain fatty acid metabolic process	1/23	35/28891	0.0275055074915166	0.0695983685603282	0.0336895675705008	Elovl6	1	0.285714285714286
GO:0016358	BP	GO:0016358	Dendrite development	2/23	327/28891	0.0276284536633219	0.0697254922713835	0.0337511026745844	Nrp1/Bdnf	2	0.0611620795107034
GO:0030522	BP	GO:0030522	Intracellular receptor signaling pathway	2/23	328/28891	0.0277846423723224	0.0699356221392578	0.0338528174780887	Sfrp1/Bdnf	2	0.0609756097560976
GO:0002790	BP	GO:0002790	Peptide secretion	2/23	329/28891	0.0279411935388882	0.0699624141091221	0.0338657863148267	Sfrp1/Oxct1	2	0.060790273556231
GO:0050769	BP	GO:0050769	Positive regulation of neurogenesis	2/23	329/28891	0.0279411935388882	0.0699624141091221	0.0338657863148267	Nrp1/Bdnf	2	0.060790273556231
GO:0050708	BP	GO:0050708	Regulation of protein secretion	2/23	331/28891	0.0282553807596441	0.0700098719185662	0.0338887585929287	Sfrp1/Oxct1	2	0.0604229607250755

GO:0010614	BP	GO:0010614	Negative regulation of cardiac muscle hypertrophy	1/23	36/28891	0.0282806451865433	0.0700098719185662	0.0338887585929287	Lmna	1	0.277777777777778
GO:0010719	BP	GO:0010719	Negative regulation of epithelial to mesenchymal transition	1/23	36/28891	0.0282806451865433	0.0700098719185662	0.0338887585929287	Sfrp1	1	0.277777777777778
GO:0007548	BP	GO:0007548	Sex differentiation	2/23	335/28891	0.0288880773944068	0.0700098719185662	0.0338887585929287	Sfrp1/Fgf9	2	0.0597014925373134
GO:0003401	BP	GO:0003401	Axis elongation	1/23	37/28891	0.0290551918911255	0.0700098719185662	0.0338887585929287	Sfrp1	1	0.27027027027027
GO:0007435	BP	GO:0007435	Salivary gland morphogenesis	1/23	37/28891	0.0290551918911255	0.0700098719185662	0.0338887585929287	Nrp1	1	0.27027027027027
GO:0014047	BP	GO:0014047	Glutamate secretion	1/23	37/28891	0.0290551918911255	0.0700098719185662	0.0338887585929287	Bdnf	1	0.27027027027027

GO:0033687	BP	GO:0033687	Osteoblast proliferation	1/23	37/28891	0.0290551918911255	0.0700098719185662	0.0338887585929287	Sfrp1	1	0.27027027027027
GO:0034389	BP	GO:0034389	Lipid droplet organization	1/23	37/28891	0.0290551918911255	0.0700098719185662	0.0338887585929287	Sqle	1	0.27027027027027
GO:0048169	BP	GO:0048169	Regulation of long-term neuronal synaptic plasticity	1/23	37/28891	0.0290551918911255	0.0700098719185662	0.0338887585929287	Bdnf	1	0.27027027027027
GO:0048846	BP	GO:0048846	Axon extension involved in axon guidance	1/23	37/28891	0.0290551918911255	0.0700098719185662	0.0338887585929287	Nrp1	1	0.27027027027027
GO:0050869	BP	GO:0050869	Negative regulation of B cell activation	1/23	37/28891	0.0290551918911255	0.0700098719185662	0.0338887585929287	Sfrp1	1	0.27027027027027
GO:0051491	BP	GO:0051491	Positive regulation of filopodium assembly	1/23	37/28891	0.0290551918911255	0.0700098719185662	0.0338887585929287	Nrp1	1	0.27027027027027

GO:1902284	BP	GO:1902284	Neuron projection extension involved in neuron projection guidance	1/23	37/28891	0.0290551918911255	0.0700098719185662	0.0338887585929287	Nrp1	1	0.27027027027027
GO:1902882	BP	GO:1902882	Regulation of response to oxidative stress	1/23	37/28891	0.0290551918911255	0.0700098719185662	0.0338887585929287	Slc25a14	1	0.27027027027027
GO:0046883	BP	GO:0046883	Regulation of hormone secretion	2/23	337/28891	0.0292065769145341	0.0701982638121258	0.0339799509808984	Sfrp1/Oxct1	2	0.0593471810089021
GO:0014741	BP	GO:0014741	Negative regulation of muscle hypertrophy	1/23	38/28891	0.0298291480353862	0.0711595844923765	0.0344452848484035	Lmna	1	0.263157894736842
GO:0045577	BP	GO:0045577	Regulation of B cell differentiation	1/23	38/28891	0.0298291480353862	0.0711595844923765	0.0344452848484035	Sfrp1	1	0.263157894736842
GO:0060071	BP	GO:0060071	Wnt signaling pathway, planar cell polarity pathway	1/23	38/28891	0.0298291480353862	0.0711595844923765	0.0344452848484035	Sfrp1	1	0.263157894736842

GO:0015833	BP	GO:0015833	Peptide transport	2/23	343/28891	0.0301706211511043	0.071533979642307	0.0346265133881317	Sfrp1/Oxct1	2	0.0583090379008746
GO:0048608	BP	GO:0048608	Reproductive structure development	2/23	343/28891	0.0301706211511043	0.071533979642307	0.0346265133881317	Sfrp1/Fgf9	2	0.0583090379008746
GO:0034381	BP	GO:0034381	Plasma lipoprotein particle clearance	1/23	39/28891	0.0306025140491518	0.071533979642307	0.0346265133881317	Anxa2	1	0.256410256410256
GO:0044342	BP	GO:0044342	Type B pancreatic cell proliferation	1/23	39/28891	0.0306025140491518	0.071533979642307	0.0346265133881317	Sfrp1	1	0.256410256410256
GO:0046329	BP	GO:0046329	Negative regulation of JNK cascade	1/23	39/28891	0.0306025140491518	0.071533979642307	0.0346265133881317	Sfrp1	1	0.256410256410256
GO:0071391	BP	GO:0071391	Cellular response to estrogen stimulus	1/23	39/28891	0.0306025140491518	0.071533979642307	0.0346265133881317	Sfrp1	1	0.256410256410256

GO:0090049	BP	GO:0090049	Regulation of cell migration involved in sprouting angiogenesis	1/23	39/28891	0.0306025140491518	0.071533979642307	0.0346265133881317	Nrp1	1	0.256410256410256
GO:1904738	BP	GO:1904738	Vascular associated smooth muscle cell migration	1/23	39/28891	0.0306025140491518	0.071533979642307	0.0346265133881317	Fgf9	1	0.256410256410256
GO:0009410	BP	GO:0009410	Response to xenobiotic stimulus	2/23	346/28891	0.030657419846703	0.071533979642307	0.0346265133881317	Sfrp1/Bdnf	2	0.0578034682080925
GO:0051146	BP	GO:0051146	Striated muscle cell differentiation	2/23	347/28891	0.0308203896435362	0.071565989511262	0.0346420079847062	Lmna/Bdnf	2	0.0576368876080692
GO:0061458	BP	GO:0061458	Reproductive system development	2/23	347/28891	0.0308203896435362	0.071565989511262	0.0346420079847062	Sfrp1/Fgf9	2	0.0576368876080692
GO:0015980	BP	GO:0015980	Energy derivation by oxidation of organic compounds	2/23	350/28891	0.0313114011997638	0.0725033818243638	0.0350957591620048	Stbd1/Bdnf	2	0.0571428571428571

GO:0033144	BP	GO:0033144	Negative regulation of intracellular steroid hormone receptor signaling pathway	1/23	40/28891	0.031375290361951	0.0725033818243638	0.0350957591620048	Sfrp1	1	0.25
GO:0032956	BP	GO:0032956	Regulation of actin cytoskeleton organization	2/23	351/28891	0.0314757704141427	0.0725607303537567	0.0351235191109234	S100a10/Nrp1	2	0.056980056980057
GO:0007431	BP	GO:0007431	Salivary gland development	1/23	41/28891	0.0321474774030128	0.0737546192093045	0.0357014291985109	Nrp1	1	0.24390243902439
GO:0008299	BP	GO:0008299	Isoprenoid biosynthetic process	1/23	41/28891	0.0321474774030128	0.0737546192093045	0.0357014291985109	Idi1	1	0.24390243902439
GO:0040013	BP	GO:0040013	Negative regulation of locomotion	2/23	358/28891	0.0326360744510275	0.0746968863926859	0.0361575400901975	Sfrp1/Nrp1	2	0.0558659217877095
GO:0038179	BP	GO:0038179	Neurotrophin signaling pathway	1/23	42/28891	0.0329190756012702	0.0749866829017057	0.0362978180776601	Bdnf	1	0.238095238095238

GO:0060218	BP	GO:0060218	Hematopoietic stem cell differentiation	1/23	42/28891	0.0329190756012702	0.0749866829017057	0.0362978180776601	Sfrp1	1	0.238095238095238
GO:0038084	BP	GO:0038084	Vascular endothelial growth factor signaling pathway	1/23	43/28891	0.0336900853853591	0.0760206867871986	0.0367983347490857	Nrp1	1	0.232558139534884
GO:0090175	BP	GO:0090175	Regulation of establishment of planar polarity	1/23	43/28891	0.0336900853853591	0.0760206867871986	0.0367983347490857	Sfrp1	1	0.232558139534884
GO:0097178	BP	GO:0097178	Ruffle assembly	1/23	43/28891	0.0336900853853591	0.0760206867871986	0.0367983347490857	Fam98a	1	0.232558139534884
GO:1904707	BP	GO:1904707	Positive regulation of vascular associated smooth muscle cell proliferation	1/23	43/28891	0.0336900853853591	0.0760206867871986	0.0367983347490857	Fgf9	1	0.232558139534884
GO:0030521	BP	GO:0030521	Androgen receptor signaling pathway	1/23	44/28891	0.0344605071836167	0.0762192811115642	0.03689446569365	Sfrp1	1	0.227272727272727

GO:0032228	BP	GO:0032228	Regulation of synaptic transmission, GABAergic	1/23	44/28891	0.0344605071836167	0.0762192811115642	0.03689446569365	Bdnf	1	0.227272727272727
GO:0035886	BP	GO:0035886	Vascular associated smooth muscle cell differentiation	1/23	44/28891	0.0344605071836167	0.0762192811115642	0.03689446569365	Fgf9	1	0.227272727272727
GO:0042220	BP	GO:0042220	Response to cocaine	1/23	44/28891	0.0344605071836167	0.0762192811115642	0.03689446569365	Bdnf	1	0.227272727272727
GO:0045671	BP	GO:0045671	Negative regulation of osteoclast differentiation	1/23	44/28891	0.0344605071836167	0.0762192811115642	0.03689446569365	Sfrp1	1	0.227272727272727
GO:0051154	BP	GO:0051154	Negative regulation of striated muscle cell differentiation	1/23	44/28891	0.0344605071836167	0.0762192811115642	0.03689446569365	Bdnf	1	0.227272727272727
GO:0051647	BP	GO:0051647	Nucleus localization	1/23	44/28891	0.0344605071836167	0.0762192811115642	0.03689446569365	Lmna	1	0.227272727272727

GO:0097484	BP	GO:0097484	Dendrite extension	1/23	44/28891	0.0344605071836167	0.0762192811115642	0.03689446569365	Bdnf	1	0.227272727272727
GO:0051402	BP	GO:0051402	Neuron apoptotic process	2/23	369/28891	0.0344933972913648	0.0762192811115642	0.03689446569365	Nrp1/Bdnf	2	0.0542005420054201
GO:0042060	BP	GO:0042060	Wound healing	2/23	370/28891	0.0346642819393968	0.0764207962755898	0.0369920104906812	S100a10/Anxa2	2	0.0540540540540541
GO:0001569	BP	GO:0001569	Branching involved in blood vessel morphogenesis	1/23	45/28891	0.0352303414240848	0.0766120123031685	0.0370845699200892	Nrp1	1	0.222222222222222
GO:0032365	BP	GO:0032365	Intracellular lipid transport	1/23	45/28891	0.0352303414240848	0.0766120123031685	0.0370845699200892	Anxa2	1	0.222222222222222
GO:0032924	BP	GO:0032924	Activin receptor signaling pathway	1/23	45/28891	0.0352303414240848	0.0766120123031685	0.0370845699200892	Fgf9	1	0.222222222222222

GO:0048147	BP	GO:0048147	Negative regulation of fibroblast proliferation	1/23	45/28891	0.0352303414240848	0.0766120123031685	0.0370845699200892	Sfrp1	1	0.222222222222222
GO:0050918	BP	GO:0050918	Positive chemotaxis	1/23	45/28891	0.0352303414240848	0.0766120123031685	0.0370845699200892	Nrp1	1	0.222222222222222
GO:1901568	BP	GO:1901568	Fatty acid derivative metabolic process	1/23	45/28891	0.0352303414240848	0.0766120123031685	0.0370845699200892	Oxct1	1	0.222222222222222
GO:0010830	BP	GO:0010830	Regulation of myotube differentiation	1/23	46/28891	0.0359995885345067	0.0774071870058115	0.037469479687792	Bdnf	1	0.217391304347826
GO:0030195	BP	GO:0030195	Negative regulation of blood coagulation	1/23	46/28891	0.0359995885345067	0.0774071870058115	0.037469479687792	Anxa2	1	0.217391304347826
GO:0030279	BP	GO:0030279	Negative regulation of ossification	1/23	46/28891	0.0359995885345067	0.0774071870058115	0.037469479687792	Sfrp1	1	0.217391304347826

GO:0032373	BP	GO:0032373	Positive regulation of sterol transport	1/23	46/28891	0.0359995885345067	0.0774071870058115	0.037469479687792	Anxa2	1	0.217391304347826
GO:0032376	BP	GO:0032376	Positive regulation of cholesterol transport	1/23	46/28891	0.0359995885345067	0.0774071870058115	0.037469479687792	Anxa2	1	0.217391304347826
GO:0071542	BP	GO:0071542	Dopaminergic neuron differentiation	1/23	47/28891	0.0367682489423319	0.0787070328921792	0.0380986790027452	Sfrp1	1	0.212765957446809
GO:1900047	BP	GO:1900047	Negative regulation of hemostasis	1/23	47/28891	0.0367682489423319	0.0787070328921792	0.0380986790027452	Anxa2	1	0.212765957446809
GO:0032873	BP	GO:0032873	Negative regulation of stress-activated MAPK cascade	1/23	48/28891	0.0375363230747103	0.0799940751747716	0.0387216806454907	Sfrp1	1	0.208333333333333
GO:0055023	BP	GO:0055023	Positive regulation of cardiac muscle tissue growth	1/23	48/28891	0.0375363230747103	0.0799940751747716	0.0387216806454907	Fgf9	1	0.208333333333333

GO:0006998	BP	GO:0006998	Nuclear envelope organization	1/23	49/28891	0.0383038113584975	0.0800833604887936	0.0387648998140146	Lmna	1	0.204081632653061
GO:0030517	BP	GO:0030517	Negative regulation of axon extension	1/23	49/28891	0.0383038113584975	0.0800833604887936	0.0387648998140146	Nrp1	1	0.204081632653061
GO:0030901	BP	GO:0030901	Midbrain development	1/23	49/28891	0.0383038113584975	0.0800833604887936	0.0387648998140146	Sfrp1	1	0.204081632653061
GO:0035774	BP	GO:0035774	Positive regulation of insulin secretion involved in cellular response to glucose stimulus	1/23	49/28891	0.0383038113584975	0.0800833604887936	0.0387648998140146	Oxct1	1	0.204081632653061
GO:0050819	BP	GO:0050819	Negative regulation of coagulation	1/23	49/28891	0.0383038113584975	0.0800833604887936	0.0387648998140146	Anxa2	1	0.204081632653061
GO:0070303	BP	GO:0070303	Negative regulation of stress-activated protein kinase signaling cascade	1/23	49/28891	0.0383038113584975	0.0800833604887936	0.0387648998140146	Sfrp1	1	0.204081632653061

GO:1990089	BP	GO:1990089	Response to nerve growth factor	1/23	49/28891	0.0383038113584975	0.0800833604887936	0.0387648998140146	Bdnf	1		0.204081632653061
GO:1990090	BP	GO:1990090	Cellular response to nerve growth factor stimulus	1/23	49/28891	0.0383038113584975	0.0800833604887936	0.0387648998140146	Bdnf	1		0.204081632653061
GO:0051962	BP	GO:0051962	Positive regulation of nervous system development	2/23	391/28891	0.0383297835916124	0.0800833604887936	0.0387648998140146	Nrp1/Bdnf	2		0.051150895140665
GO:1902903	BP	GO:1902903	Regulation of supramolecular fiber organization	2/23	393/28891	0.0386864301673444	0.0806527968053984	0.0390405393679608	S100a10/Nrp1	2		0.0508905852417303
GO:0006887	BP	GO:0006887	Exocytosis	2/23	395/28891	0.0390443705813988	0.0807517563302209	0.0390884414045633	S100a10/Anxa2	2		0.0506329113924051
GO:0060562	BP	GO:0060562	Epithelial tube morphogenesis	2/23	395/28891	0.0390443705813988	0.0807517563302209	0.0390884414045633	Sfrp1/Nrp1	2		0.0506329113924051

GO:0048483	BP	GO:0048483	Autonomic nervous system development	1/23	50/28891	0.0390707142202529	0.0807517563302209	0.0390884414045633	Nrp1	1	0.2
GO:0071526	BP	GO:0071526	Semaphorin-plexin signaling pathway	1/23	50/28891	0.0390707142202529	0.0807517563302209	0.0390884414045633	Nrp1	1	0.2
GO:0060421	BP	GO:0060421	Positive regulation of heart growth	1/23	51/28891	0.0398370320862402	0.0818066676032214	0.0395990784402839	Fgf9	1	0.196078431372549
GO:0090199	BP	GO:0090199	Regulation of release of cytochrome c from mitochondria	1/23	51/28891	0.0398370320862402	0.0818066676032214	0.0395990784402839	Lmna	1	0.196078431372549
GO:1902041	BP	GO:1902041	Regulation of extrinsic apoptotic signaling pathway via death domain receptors	1/23	51/28891	0.0398370320862402	0.0818066676032214	0.0395990784402839	Sfrp1	1	0.196078431372549
GO:1990778	BP	GO:1990778	Protein localization to cell periphery	2/23	400/28891	0.0399448518424973	0.0818528053781089	0.0396214117466067	S100a10/Anxa2	2	0.05

GO:0032970	BP	GO:0032970	Regulation of actin filament-based process	2/23	402/28891	0.040307284347783	0.0824193724723324	0.0398956624337836	S100a10/Nrp1	2	0.0497512437810945
GO:0046879	BP	GO:0046879	Hormone secretion	2/23	403/28891	0.040488978304255	0.0824958728850576	0.0399326929831627	Sfrp1/Oxct1	2	0.0496277915632754
GO:0043403	BP	GO:0043403	Skeletal muscle tissue regeneration	1/23	52/28891	0.0406027653824267	0.0824958728850576	0.0399326929831627	Kpna1	1	0.192307692307692
GO:0060043	BP	GO:0060043	Regulation of cardiac muscle cell proliferation	1/23	52/28891	0.0406027653824267	0.0824958728850576	0.0399326929831627	Fgf9	1	0.192307692307692
GO:0007178	BP	GO:0007178	Transmembrane receptor protein serine/threonine kinase signaling pathway	2/23	405/28891	0.0408533187624211	0.0828294560109129	0.04009416618277	Sfrp1/Fgf9	2	0.0493827160493827
GO:0001881	BP	GO:0001881	Receptor recycling	1/23	53/28891	0.0413679145344847	0.0833441807533001	0.0403433222240331	Anxa2	1	0.188679245283019

GO:0021879	BP	GO:0021879	Forebrain neuron differentiation	1/23	53/28891	0.0413679145344847	0.0833441807533001	0.0403433222240331	Nrp1	1		0.188679245283019
GO:0030850	BP	GO:0030850	Prostate gland development	1/23	53/28891	0.0413679145344847	0.0833441807533001	0.0403433222240331	Sfrp1	1		0.188679245283019
GO:0009914	BP	GO:0009914	Hormone transport	2/23	411/28891	0.0419539194060231	0.0834815047295709	0.0404097948364423	Sfrp1/Oxct1	2		0.048661800486618
GO:0032535	BP	GO:0032535	Regulation of cellular component size	2/23	411/28891	0.0419539194060231	0.0834815047295709	0.0404097948364423	Nrp1/Bdnf	2		0.048661800486618
GO:0002042	BP	GO:0002042	Cell migration involved in sprouting angiogenesis	1/23	54/28891	0.0421324799677918	0.0834815047295709	0.0404097948364423	Nrp1	1		0.185185185185185
GO:0010823	BP	GO:0010823	Negative regulation of mitochondrion organization	1/23	54/28891	0.0421324799677918	0.0834815047295709	0.0404097948364423	Lmna	1		0.185185185185185

GO:0035272	BP	GO:0035272	Exocrine system development	1/23	54/28891	0.0421324799677918	0.0834815047295709	0.0404097948364423	Nrp1	1	0.185185185185185
GO:0045773	BP	GO:0045773	Positive regulation of axon extension	1/23	54/28891	0.0421324799677918	0.0834815047295709	0.0404097948364423	Nrp1	1	0.185185185185185
GO:0048546	BP	GO:0048546	Digestive tract morphogenesis	1/23	54/28891	0.0421324799677918	0.0834815047295709	0.0404097948364423	Sfrp1	1	0.185185185185185
GO:0071709	BP	GO:0071709	Membrane assembly	1/23	54/28891	0.0421324799677918	0.0834815047295709	0.0404097948364423	S100a10	1	0.185185185185185
GO:0014888	BP	GO:0014888	Striated muscle adaptation	1/23	55/28891	0.0428964621074298	0.0848200147650004	0.0410577097978872	Lmna	1	0.181818181818182
GO:0031529	BP	GO:0031529	Ruffle organization	1/23	56/28891	0.0436598613781861	0.0861518663820586	0.0417024017062596	Fam98a	1	0.178571428571429

GO:0001662	BP	GO:0001662	Behavioral fear response	1/23	57/28891	0.04442267820<br>45536	0.08676445702<br>27432	0.04199893040<br>67063	Bdnf	1	0.17543859649<br>1228
GO:0021545	BP	GO:0021545	Cranial nerve development	1/23	57/28891	0.04442267820<br>45536	0.08676445702<br>27432	0.04199893040<br>67063	Nrp1	1	0.17543859649<br>1228
GO:0050732	BP	GO:0050732	Negative regulation of peptidyl-tyrosine phosphorylation	1/23	57/28891	0.04442267820<br>45536	0.08676445702<br>27432	0.04199893040<br>67063	Sfrp1	1	0.17543859649<br>1228
GO:0061383	BP	GO:0061383	Trabecula morphogenesis	1/23	57/28891	0.04442267820<br>45536	0.08676445702<br>27432	0.04199893040<br>67063	Sfrp1	1	0.17543859649<br>1228
GO:1901861	BP	GO:1901861	Regulation of muscle tissue development	1/23	57/28891	0.04442267820<br>45536	0.08676445702<br>27432	0.04199893040<br>67063	Bdnf	1	0.17543859649<br>1228
GO:0050678	BP	GO:0050678	Regulation of epithelial cell proliferation	2/23	426/28891	0.04475447541<br>36789	0.08723484130<br>43049	0.04222662314<br>38433	Sfrp1/Fgf9	2	0.04694835680<br>75117

GO:0002209	BP	GO:0002209	Behavioral defense response	1/23	58/28891	0.0451849130107301	0.0873635717284077	0.0422889359883956	Bdnf	1	0.172413793103448
GO:0043536	BP	GO:0043536	Positive regulation of blood vessel endothelial cell migration	1/23	58/28891	0.0451849130107301	0.0873635717284077	0.0422889359883956	Nrp1	1	0.172413793103448
GO:0050922	BP	GO:0050922	Negative regulation of chemotaxis	1/23	58/28891	0.0451849130107301	0.0873635717284077	0.0422889359883956	Nrp1	1	0.172413793103448
GO:0051489	BP	GO:0051489	Regulation of filopodium assembly	1/23	58/28891	0.0451849130107301	0.0873635717284077	0.0422889359883956	Nrp1	1	0.172413793103448
GO:0030514	BP	GO:0030514	Negative regulation of BMP signaling pathway	1/23	59/28891	0.0459465662206199	0.0884794317381013	0.0428290756780667	Sfrp1	1	0.169491525423729
GO:0035567	BP	GO:0035567	Non-canonical Wnt signaling pathway	1/23	59/28891	0.0459465662206199	0.0884794317381013	0.0428290756780667	Sfrp1	1	0.169491525423729

GO:0009306	BP	GO:0009306	Protein secretion	2/23	434/28891	0.0462762594561571	0.0889357371111317	0.0430499534229834	Sfrp1/Oxct1	2	0.0460829493087558
GO:0035592	BP	GO:0035592	Establishment of protein localization to extracellular region	2/23	435/28891	0.0464678395044079	0.0891253161694544	0.0431417204661977	Sfrp1/Oxct1	2	0.0459770114942529
GO:0030199	BP	GO:0030199	Collagen fibril organization	1/23	61/28891	0.0474681295456851	0.0899643008583242	0.0435478367581592	Anxa2	1	0.163934426229508
GO:0035924	BP	GO:0035924	Cellular response to vascular endothelial growth factor stimulus	1/23	61/28891	0.0474681295456851	0.0899643008583242	0.0435478367581592	Nrp1	1	0.163934426229508
GO:0043407	BP	GO:0043407	Negative regulation of MAP kinase activity	1/23	61/28891	0.0474681295456851	0.0899643008583242	0.0435478367581592	Sfrp1	1	0.163934426229508
GO:0044091	BP	GO:0044091	Membrane biogenesis	1/23	61/28891	0.0474681295456851	0.0899643008583242	0.0435478367581592	S100a10	1	0.163934426229508

GO:0046676	BP	GO:0046676	Negative regulation of insulin secretion	1/23	61/28891	0.0474681295456851	0.0899643008583242	0.0435478367581592	Sfrp1	1	0.163934426229508
GO:0098926	BP	GO:0098926	Postsynaptic signal transduction	1/23	61/28891	0.0474681295456851	0.0899643008583242	0.0435478367581592	Kpna1	1	0.163934426229508
GO:0071692	BP	GO:0071692	Protein localization to extracellular region	2/23	441/28891	0.0476235946343517	0.0900809216061997	0.0436042878308919	Sfrp1/Oxct1	2	0.0453514739229025
GO:0001755	BP	GO:0001755	Neural crest cell migration	1/23	62/28891	0.0482280405071993	0.0910446670204806	0.0440707954075319	Nrp1	1	0.161290322580645
GO:0042596	BP	GO:0042596	Fear response	1/23	63/28891	0.0489873715651038	0.0917556432244815	0.0444149483145781	Bdnf	1	0.158730158730159
GO:0045620	BP	GO:0045620	Negative regulation of lymphocyte differentiation	1/23	63/28891	0.0489873715651038	0.0917556432244815	0.0444149483145781	Sfrp1	1	0.158730158730159

GO:2000648	BP	GO:2000648	Positive regulation of stem cell proliferation	1/23	63/28891	0.0489873715651038	0.0917556432244815	0.0444149483145781	Fgf9	1	0.158730158730159
GO:2001238	BP	GO:2001238	Positive regulation of extrinsic apoptotic signaling pathway	1/23	63/28891	0.0489873715651038	0.0917556432244815	0.0444149483145781	Sfrp1	1	0.158730158730159
GO:0001736	BP	GO:0001736	Establishment of planar polarity	1/23	64/28891	0.0497461231418349	0.0920975523031268	0.0445804517487283	Sfrp1	1	0.15625
GO:0019646	BP	GO:0019646	Aerobic electron transport chain	1/23	64/28891	0.0497461231418349	0.0920975523031268	0.0445804517487283	Bdnf	1	0.15625
GO:0046622	BP	GO:0046622	Positive regulation of organ growth	1/23	64/28891	0.0497461231418349	0.0920975523031268	0.0445804517487283	Fgf9	1	0.15625
GO:0046850	BP	GO:0046850	Regulation of bone remodeling	1/23	64/28891	0.0497461231418349	0.0920975523031268	0.0445804517487283	Sfrp1	1	0.15625

GO:0048260	BP	GO:0048260	Positive regulation of receptor-mediated endocytosis	1/23	64/28891	0.0497461231418349	0.0920975523031268	0.0445804517487283	Anxa2	1	0.15625
GO:0090497	BP	GO:0090497	Mesenchymal cell migration	1/23	64/28891	0.0497461231418349	0.0920975523031268	0.0445804517487283	Nrp1	1	0.15625
GO:0016363	CC	GO:0016363	Nuclear matrix	3/23	107/28573	8.5617900882434e-05	0.00676381416971228	0.0047765776281779	S100a10/Lmna/Anxa2	3	0.280373831775701
GO:0034399	CC	GO:0034399	Nuclear periphery	3/23	136/28573	0.000174202639653102	0.00687745782002492	0.00485683230461453	S100a10/Lmna/Anxa2	3	0.220588235294118
GO:0062023	CC	GO:0062023	Collagen-containing extracellular matrix	4/23	396/28573	0.000261169284304744	0.00687745782002492	0.00485683230461453	Sfrp1/Fgf9/S100a10/Anxa2	4	0.101010101010101
GO:0005604	CC	GO:0005604	Basement membrane	2/23	115/28573	0.00384433660460623	0.0674405606977449	0.0476262453961423	Fgf9/Anxa2	2	0.173913043478261

GO:0005882	CC	GO:0005882	Intermediate filament	2/23	139/28573	0.00555938490942547	0.0674405606977449	0.0476262453961423	Lmna/Nrp1	2	0.143884892086331
GO:0042564	CC	GO:0042564	NLS-dependent protein nuclear import complex	1/23	10/28573	0.00802172071678986	0.0674405606977449	0.0476262453961423	Kpna1	1	1
GO:0042383	CC	GO:0042383	Sarcolemma	2/23	170/28573	0.00820173850019588	0.0674405606977449	0.0476262453961423	Stbd1/Anxa2	2	0.117647058823529
GO:0045111	CC	GO:0045111	Intermediate filament cytoskeleton	2/23	175/28573	0.00867161164402702	0.0674405606977449	0.0476262453961423	Lmna/Nrp1	2	0.114285714285714
GO:0030062	CC	GO:0030062	Mitochondrial tricarboxylic acid cycle enzyme complex	1/23	11/28573	0.00882049887116831	0.0674405606977449	0.0476262453961423	Bckdhb	1	0.909090909090909
GO:0005883	CC	GO:0005883	Neurofilament	1/23	12/28573	0.0096186617633317	0.0674405606977449	0.0476262453961423	Nrp1	1	0.833333333333333

GO:0031074	CC	GO:0031074	Nucleocytoplasmic transport complex	1/23	12/28573	0.0096186617633317	0.0674405606977449	0.0476262453961423	Kpna1	1	0.833333333333333
GO:0045240	CC	GO:0045240	Dihydrolipoyl dehydrogenase complex	1/23	14/28573	0.0112131435702262	0.0674405606977449	0.0476262453961423	Bckdhb	1	0.714285714285714
GO:0005652	CC	GO:0005652	Nuclear lamina	1/23	15/28573	0.0120094633887735	0.0674405606977449	0.0476262453961423	Lmna	1	0.666666666666667
GO:0030061	CC	GO:0030061	Mitochondrial crista	1/23	16/28573	0.0128051697527364	0.0674405606977449	0.0476262453961423	Bdnf	1	0.625
GO:0043220	CC	GO:0043220	Schmidt-Lanterman incisure	1/23	16/28573	0.0128051697527364	0.0674405606977449	0.0476262453961423	Anxa2	1	0.625
GO:0043218	CC	GO:0043218	Compact myelin	1/23	21/28573	0.0167745155318317	0.074453728095378	0.0525789152439045	Anxa2	1	0.476190476190476

GO:0045239	CC	GO:0045239	Tricarboxylic acid cycle enzyme complex	1/23	21/28573	0.0167745155318317	0.074453728095378	0.0525789152439045	Bckdhb	1	0.476190476190476
GO:0090575	CC	GO:0090575	RNA polymerase II transcription regulator complex	2/23	249/28573	0.0169641405786937	0.074453728095378	0.0525789152439045	S100a10/Anxa2	2	0.0803212851405622
GO:0045121	CC	GO:0045121	Membrane raft	2/23	301/28573	0.0241885952404212	0.096135307297497	0.0678903569189519	S100a10/Anxa2	2	0.0664451827242525
GO:0098857	CC	GO:0098857	Membrane microdomain	2/23	302/28573	0.024338052480379	0.096135307297497	0.0678903569189519	S100a10/Anxa2	2	0.0662251655629139
GO:0098992	CC	GO:0098992	Neuronal dense core vesicle	1/23	35/28573	0.0278076323959037	0.104609664727447	0.0738749131319747	Bdnf	1	0.285714285714286
GO:0005769	CC	GO:0005769	Early endosome	2/23	353/28573	0.0324567192157879	0.116549128093057	0.0823065128438641	Nrp1/Anxa2	2	0.0566572237960034

GO:0150034	CC	GO:0150034	Distal axon	2/23	397/28573	0.0402010492474844	0.138081864806577	0.0975128425682689	Nrp1/Bdnf	2	0.0503778337531486
GO:0031045	CC	GO:0031045	Dense core granule	1/23	54/28573	0.0425918700348675	0.140198238864772	0.0990074171863149	Bdnf	1	0.185185185185185
GO:0098686	CC	GO:0098686	Hippocampal mossy fiber to CA3 synapse	1/23	61/28573	0.0479842894639739	0.147134346447637	0.103905667711189	Bdnf	1	0.163934426229508
GO:0098984	CC	GO:0098984	Neuron to neuron synapse	2/23	448/28573	0.0499635153895093	0.147134346447637	0.103905667711189	Kpna1/Bdnf	2	0.0446428571428571
GO:0008201	MF	GO:0008201	Heparin binding	3/23	179/28407	0.000397086481465961	0.0340881811975759	0.0230188313945895	Sfrp1/Fgf9/Nrp1	3	0.167597765363128
GO:0000287	MF	GO:0000287	Magnesium ion binding	3/23	214/28407	0.000667960520397278	0.0340881811975759	0.0230188313945895	Idi1/Snrk/Ppa2	3	0.14018691588785

GO:0005539	MF	GO:0005539	Glycosaminoglycan binding	3/23	243/28407	0.000964759845214413	0.0340881811975759	0.0230188313945895	Sfrp1/Fgf9/Nrp1	3	0.123456790123457
GO:1901681	MF	GO:1901681	Sulfur compound binding	3/23	301/28407	0.0017826358056984	0.0443639378609233	0.0299577733321031	Sfrp1/Fgf9/Nrp1	3	0.0996677740863787
GO:0004722	MF	GO:0004722	Protein serine/threonine phosphatase activity	2/23	84/28407	0.0020994774983271	0.0443639378609233	0.0299577733321031	Ublcp1/Ppa2	2	0.238095238095238
GO:0048306	MF	GO:0048306	Calcium-dependent protein binding	2/23	92/28407	0.00251116629401453	0.0443639378609233	0.0299577733321031	S100a10/Anxa2	2	0.217391304347826
GO:0008083	MF	GO:0008083	Growth factor activity	2/23	149/28407	0.00643194237098829	0.0890287899081175	0.0601187459161071	Fgf9/Bdnf	2	0.134228187919463
GO:0004497	MF	GO:0004497	Monooxygenase activity	2/23	153/28407	0.00676982377210556	0.0890287899081175	0.0601187459161071	Sqle/Msmo1	2	0.130718954248366

GO:0004721	MF	GO:0004721	Phosphoprotein phosphatase activity	2/23	162/28407	0.00755904819974582	0.0890287899081175	0.0601187459161071	Ublcp1/Ppa2	2	0.123456790123457
GO:0017154	MF	GO:0017154	Semaphorin receptor activity	1/23	12/28407	0.00967463039262173	0.10255108216179	0.0692499859682398	Nrp1	1	0.833333333333333
GO:0055102	MF	GO:0055102	Lipase inhibitor activity	1/23	14/28407	0.0112783394521025	0.105017985359471	0.0709158193092753	Anxa2	1	0.714285714285714
GO:0044548	MF	GO:0044548	S100 protein binding	1/23	15/28407	0.0120792621510988	0.105017985359471	0.0709158193092753	Anxa2	1	0.666666666666667
GO:0005165	MF	GO:0005165	Neurotrophin receptor binding	1/23	16/28407	0.0128795642421993	0.105017985359471	0.0709158193092753	Bdnf	1	0.625
GO:0016705	MF	GO:0016705	Oxidoreductase activity, acting on paired donors, with incorporation or reduction of molecular oxygen	2/23	229/28407	0.0146437574140897	0.106494064037064	0.0719125755165873	Sqle/Msmo1	2	0.0873362445414847

GO:0005123	MF	GO:0005123	Death receptor binding	1/23	20/28407	0.0160745757037077	0.106494064037064	0.0719125755165873	Bdnf	1	0.5
GO:0061608	MF	GO:0061608	Nuclear import signal receptor activity	1/23	20/28407	0.0160745757037077	0.106494064037064	0.0719125755165873	Kpna1	1	0.5
GO:0016791	MF	GO:0016791	Phosphatase activity	2/23	263/28407	0.0190074942842251	0.108095661864615	0.0729940914279424	Ublcp1/Ppa2	2	0.0760456273764259
GO:0008139	MF	GO:0008139	Nuclear localization sequence binding	1/23	24/28407	0.0192596941043789	0.108095661864615	0.0729940914279424	Kpna1	1	0.416666666666667
GO:0016863	MF	GO:0016863	Intramolecular oxidoreductase activity, transposing C=C bonds	1/23	25/28407	0.0200544313427112	0.108095661864615	0.0729940914279424	Idi1	1	0.4
GO:0005104	MF	GO:0005104	Fibroblast growth factor receptor binding	1/23	27/28407	0.0216420581793024	0.108095661864615	0.0729940914279424	Fgf9	1	0.37037037037037

GO:0005452	MF	GO:0005452	Solute:inorganic anion antiporter activity	1/23	27/28407	0.0216420581793024	0.108095661864615	0.0729940914279424	Slc25a14	1	0.37037037037037
GO:0030247	MF	GO:0030247	Polysaccharide binding	1/23	28/28407	0.0224349486888823	0.108095661864615	0.0729940914279424	Stbd1	1	0.357142857142857
GO:0017147	MF	GO:0017147	Wnt-protein binding	1/23	31/28407	0.0248099340439496	0.109577208694111	0.0739945401310778	Sfrp1	1	0.32258064516129
GO:0140142	MF	GO:0140142	Nucleocytoplasmic carrier activity	1/23	31/28407	0.0248099340439496	0.109577208694111	0.0739945401310778	Kpna1	1	0.32258064516129
GO:0008157	MF	GO:0008157	Protein phosphatase 1 binding	1/23	38/28407	0.0303301221301061	0.12859971783165	0.0868399286251459	Lmna	1	0.263157894736842
GO:0042578	MF	GO:0042578	Phosphoric ester hydrolase activity	2/23	350/28407	0.0322956954557903	0.130301685606465	0.0879892216607709	Ublcp1/Ppa2	2	0.0571428571428571

GO:0005109	MF	GO:0005109	Frizzled binding	1/23	42/28407	0.0334710824729209	0.130301685606465	0.0879892216607709	Sfrp1	1	0.238095238095238
GO:0071949	MF	GO:0071949	FAD binding	1/23	45/28407	0.0358204137211022	0.130301685606465	0.0879892216607709	Sqle	1	0.222222222222222
GO:0005048	MF	GO:0005048	Signal sequence binding	1/23	46/28407	0.0366023095847365	0.130301685606465	0.0879892216607709	Kpna1	1	0.217391304347826
GO:0050661	MF	GO:0050661	NADP binding	1/23	47/28407	0.0373835989214865	0.130301685606465	0.0879892216607709	Hibadh	1	0.212765957446809
GO:0032813	MF	GO:0032813	Tumor necrosis factor receptor superfamily binding	1/23	48/28407	0.0381642821804712	0.130301685606465	0.0879892216607709	Bdnf	1	0.208333333333333
GO:0004879	MF	GO:0004879	Nuclear receptor activity	1/23	53/28407	0.0420586230059895	0.130301685606465	0.0879892216607709	Nr1d2	1	0.188679245283019

GO:0005544	MF	GO:0005544	Calcium-dependent phospholipid binding	1/23	53/28407	0.0420586230059895	0.130301685606465	0.0879892216607709	Anxa2	1	0.188679245283019
GO:0098531	MF	GO:0098531	Ligand-activated transcription factor activity	1/23	53/28407	0.0420586230059895	0.130301685606465	0.0879892216607709	Nr1d2	1	0.188679245283019
GO:0016709	MF	GO:0016709	Oxidoreductase activity, acting on paired donors, with incorporation or reduction of molecular oxygen, NAD(P)H as one donor, and incorporation of one atom of oxygen	1/23	55/28407	0.043612132475632	0.130301685606465	0.0879892216607709	Sqle	1	0.181818181818182
GO:0016860	MF	GO:0016860	Intramolecular oxidoreductase activity	1/23	58/28407	0.0459378790736262	0.130301685606465	0.0879892216607709	Idi1	1	0.172413793103448
GO:0050840	MF	GO:0050840	Extracellular matrix binding	1/23	58/28407	0.0459378790736262	0.130301685606465	0.0879892216607709	Anxa2	1	0.172413793103448
GO:0004714	MF	GO:0004714	Transmembrane receptor protein tyrosine kinase activity	1/23	59/28407	0.0467119250287328	0.130301685606465	0.0879892216607709	Nrp1	1	0.169491525423729
